# PSFM-DBT: Identifying DNA-Binding Proteins by Combing Position Specific Frequency Matrix and Distance-Bigram Transformation

**DOI:** 10.3390/ijms18091856

**Published:** 2017-08-25

**Authors:** Jun Zhang, Bin Liu

**Affiliations:** School of Computer Science and Technology, Harbin Institute of Technology Shenzhen Graduate School, Shenzhen 518055, China; junzhangcs@foxmail.com

**Keywords:** PSFM-DBT, DNA binding protein, distance bigram transformation, PSFM

## Abstract

DNA-binding proteins play crucial roles in various biological processes, such as DNA replication and repair, transcriptional regulation and many other biological activities associated with DNA. Experimental recognition techniques for DNA-binding proteins identification are both time consuming and expensive. Effective methods for identifying these proteins only based on protein sequences are highly required. The key for sequence-based methods is to effectively represent protein sequences. It has been reported by various previous studies that evolutionary information is crucial for DNA-binding protein identification. In this study, we employed four methods to extract the evolutionary information from Position Specific Frequency Matrix (PSFM), including Residue Probing Transformation (RPT), Evolutionary Difference Transformation (EDT), Distance-Bigram Transformation (DBT), and Trigram Transformation (TT). The PSFMs were converted into fixed length feature vectors by these four methods, and then respectively combined with Support Vector Machines (SVMs); four predictors for identifying these proteins were constructed, including PSFM-RPT, PSFM-EDT, PSFM-DBT, and PSFM-TT. Experimental results on a widely used benchmark dataset PDB1075 and an independent dataset PDB186 showed that these four methods achieved state-of-the-art-performance, and PSFM-DBT outperformed other existing methods in this field. For practical applications, a user-friendly webserver of PSFM-DBT was established, which is available at http://bioinformatics.hitsz.edu.cn/PSFM-DBT/.

## 1. Introduction

DNA-binding proteins play crucial roles in various biological processes, such as DNA replication and repair, transcriptional regulation, the combination and separation of single-stranded DNA and other biological activities associated with DNA. Therefore, effective methods for identifying DNA-binding proteins are highly required.

There are some experimental recognition techniques for DNA-binding protein identification, such as filter binding assays, genetic analysis, chromatin immune precipitation on microarrays, and X-ray crystallography. However, these methods are both time consuming and expensive [1]. With the development of genomic and proteomic sequencing techniques, the number of protein sequences is growing rapidly. It is highly desired to develop fast and effective computational methods to identify the DNA binding proteins based on the protein sequences. In this regard, some computational methods based on machine learning algorithms have been proposed. These methods can be roughly divided into two groups: structure-based methods [2,3,4,5,6,7,8] and sequence-based methods. Stawiski et al. [7] analyzed the positive electrostatic patches in protein surface, and represented proteins with 12 features including the patch size, percent helix in patch, average surface area, hydrogen-bonding potential, three conserved special residues, and other features of the protein. These features were then inputted into a Neural Network (NN) for identifying DNA-binding proteins.

A webserver for the identification of DNA binding proteins (iDBPs) [9] recently was constructed for DNA binding protein identification, in which a random forest (RF) classifier was trained based on multiple structural features, such as electrostatic potential, cluster-based amino acid conservation patterns, secondary structure content of the patches, dipole moment and hydrogen-bonding potential. Song et al developed nDNA-Prot, which employed an imbalanced classifier [10]. Bhardwaj et al. [11] examined the sizes of positively charged patches on the surface of proteins, and used generated structural features to train a support vector machine (SVM) classifier. These structure-based methods achieved state-of-the-art performance. However, they require the structure information of proteins, which is not always available. In contrast, the sequence-based methods identify the DNA binding proteins only based on the sequence information of proteins, for example, Cai and Lin [12] proposed a method representing proteins employing pseudo amino acid composition (PseAAC) [13], in which amino acid composition, limited range correlation of hydrophobicity and solvent accessible surface area were taken into account. In method DNA-Prot [14], proteins was represented by various sequence properties, including frequency of amino acid, physical chemical properties, secondary structure, neutral amino acids, etc. Fang et al. [15] extracted protein features by using autocross-covariance (ACC) transform, pseudo amino acid composition, and dipeptide composition. Evolutionary profiles were introduced into this field by Kumar et al. [16]; they also developed a SVM-based predictor based on generated features. Recently, evolutionary profile was widely used in this field. Position specific score matrix distance transformation (PSSM-DT) [17] combined PSSM distance transformation with SVM. An improved DNA-binding protein prediction method (Local-DPP) [18] extracted local evolutionary information from some equally sized sub-PSSMs to represent proteins. Zhang et al. [19] proposed a new method in which feature vectors were extracted from PSSM, secondary structure, and physicochemical properties. They further improved the performance by using an improved Binary Firefly Algorithm (BFA) to filter noisy features and select optimal parameters for the classifier. Waris et al. [20] combined three different protein representations (dipeptide composition, split amino acid composition, and PSSM), and three machine learning algorithms (*k* Nearest Neighbor (KNN), SVM, and RF).

All these aforementioned methods have made great contributions to the development of this important field; the profile-based methods especially achieved state-of-the-art performance by incorporating evolutionary information into the predictors. Almost all of the machine-learning-based classifiers require fixed length feature vectors as inputs [21]. However, it is not an easy task to convert the profiles into feature vectors because a profile such as PSSM is a matrix with different dimensions. In this study, we employed four methods to extract the evolutionary information from Position Specific Frequency Matrix (PSFM), including Residue Probing Transformation (RPT) [22], Evolutionary Difference Transformation (EDT) [3], Distance-Bigram Transformation (DBT) [17,23,24], and Trigram Transformation (TT) [25]. The PSFMs were converted into fixed length feature vectors by these four methods, and then respectively combined with SVMs; four predictors for DNA binding protein identification were constructed, including PSFM-RPT, PSFM-EDT, PSFM-DBT and PSFM-TT. Experimental results on a widely used benchmark dataset and an independent dataset showed that these four methods achieved state-of-the-art-performance, and outperformed other existing methods in this field.

## 2. Result and Discussion

### 2.1. Impact of the Maximum Distance D

**Figure 1:**
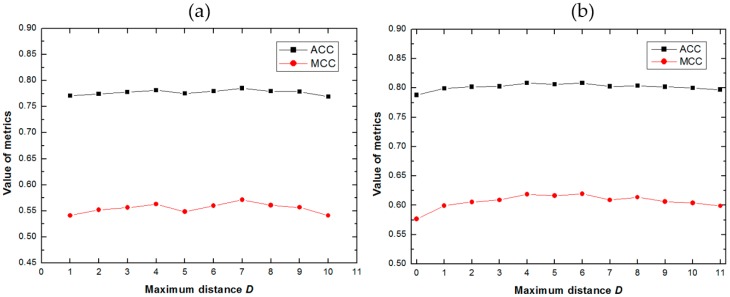
(**a**) The performance of Position Specific Frequency Matrix-Evolutionary Difference Transformation (PSFM-EDT) with different D on the benchmark dataset via five-cross validation. (**b**) The performance of Position Specific Frequency Matrix-Distance-Bigram Transformation (PSFM-DBT) with different *D* on the benchmark dataset via five-cross validation.

In order to evaluate the performance of the proposed methods, and select the optimized parameter, we explored the effect of the parameter *D* (see Equations (9) and (12)) in methods PSFM-EDT and PSFM-DBT. Taking into account the time cost, the predictive results were obtained by using 5-fold cross validation on benchmark dataset. The results of PSFM-EDT and PSFM-DBT with different values of *D* are shown in Figure 1a,b, respectively, from which we can see that PSFM-EDT and PSFM-DBT can achieve stable performance with different *D* values, and they achieved best performance when *D* = 7 and *D* = 4 respectively. Therefore, the parameter *D* of PSFM-EDT was set as 7 and the parameter *D* of PSFM-DBT was set as 4.

### 2.2. Comparison of the Four PSFM-Based Methods

**Figure 2:**
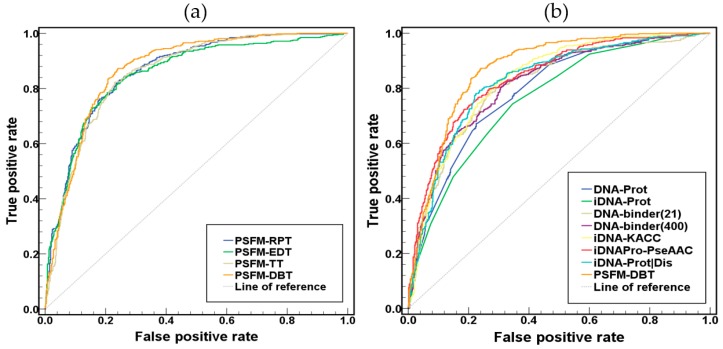
(**a**) The Receiver Operating Characteristic (ROC) curves of the four PSFM-based methods on the benchmark dataset using the jackknife tests. (**b**) The ROC curves of various methods on the benchmark dataset using the jackknife tests.

The performance of the four proposed PSFM-based methods was shown in Table 1 by using jackknife test on benchmark dataset, and the corresponding ROC curves of these methods were shown in Figure 2a. From Table 1 and Figure 2a we can see that the PSFM-DBT is better than all the other methods. The reason is that PSFM-DBT incorporates more sequence-order effects by considering bigrams separated by different distances, which is more efficient than the other three approaches. Furthermore, a recent study showed that these sequence-order effects are critical for DNA binding protein identification [23].

### 2.3. Comparison with Existing Methods

The performance of PSFM-DBT was compared with other existing methods on the benchmark dataset, including DNAbinder [16], DNA-Prot [14], iDNA-Prot [26], iDNA-KACC [27], PseDNA-Pro [17], iDNA-Prot|dis [23], iDNAPro-PseAAC [28], PSSM-DT [17] and Local-DPP [18]. Among these nine methods, DNAbinder, iDNAPro-PseAAC, PSSM-DT and Local-DPP are profile-based methods, and the other five methods are sequence-based methods. The performance of various methods was shown in Table 2 and Figure 2b, from which we can see that the profile-based methods achieved higher performance than other sequence-based methods, and PSFM-DBT obviously outperformed other methods, indicating that evolutionary information is critical for DNA binding protein identification, and PSFM-DBT is an efficient method. ACC represents the percentage of the samples which are correctly predicted among all samples; MCC explains the reliability of models; Sensitivity (SN) is an important measure, it presents the accuracy of predicting positive samples; Specificity (SP) denotes the percentage of true negative samples among negative samples; AUC is the area under ROC curve which gives a measure of the quality of binary classification methods, the larger AUC is, the better its predictive quality is.

### 2.4. Independent Test

**Figure 3:**
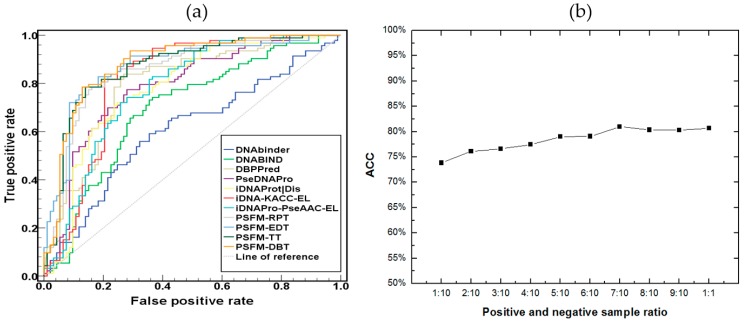
(**a**) The ROC curves of various methods on the independent dataset PDB186. (**b**) The performance of PSFM-DBT on the independent dataset with different ratios of positive samples.

In this study, the four proposed PSFM-based methods were further evaluated on an independent dataset PDB186 constructed by Lou et al. [1]. It contains 93 DNA-binding proteins and 93 non-DNA-binding proteins selected from PDB. Because there are some proteins in benchmark dataset share more than 25% sequence identity with some proteins in independent dataset, this will lead to homology bias. In order to avoid this problem, the NCBI’s BLASTCLUST [29] was employed to filter those proteins from the benchmark dataset which have more than 25% sequence identity to any protein in a same subset of the PDB186 dataset. Then we retrained the four proposed PSFM-based methods on such a reduced benchmark dataset, based on which the proteins in the independent dataset were predicted, and the results were shown in Table 3 and Figure 3a. PSFM-DBT achieved the top performance, which further demonstrates that it is a useful predictor for DNA binding protein identification.

The number of DNA-binding proteins is much lower than that of the non DNA-binding proteins in the real world. In order to simulate real world applications, we evaluated the performance of PSFM-DBT on this independent dataset with different ratios of positive and negative samples, and the results were shown in Figure 3b, from which we can see that the ACC increases slightly as the ratio of positive samples increases, indicating that the PSFM-DBT can achieve stable performance and it is suitable for DNA binding protein prediction.

### 2.5. Feature Analysis

To further investigate the importance of the features and to reveal the biological meaning of the features in proposed PSFM-DBT, we followed some previous studies [30,31] to calculate the discriminant weight vector in the feature space. The sequence-specific weight obtained from the SVM training process can be used to calculate the discriminant weight of each feature to measure the importance of the features. Given the weight vectors of the training set with *N* samples obtained from the kernel-based training **A** = [*a*_1_, *a*_2_, *a*_3_, …, *a_N_*], the feature discriminant weight vector **W** in the feature space can be calculated by the following equation:

(1) $$W=A\cdot M={\left[\begin{array}{c} a_{1}\\ a_{2}\\ \vdots \\ a_{N} \end{array}\right]}^{T}\left[\begin{array}{cccc} m_{11} & m_{12} & \cdots & m_{1j}\\ m_{21} & m_{22} & \cdots & m_{2j}\\ \vdots & \vdots & \ddots & \vdots \\ m_{N1} & m_{N2} & \cdots & m_{Nj} \end{array}\right]$$

where **M** is the matrix of sequence representatives; **A** is the weight vectors of the training samples; *N* is the number of training samples; *j* is the dimension of the feature vector. The element in **W** represents the discriminative power of the corresponding feature.

**Figure 4:**
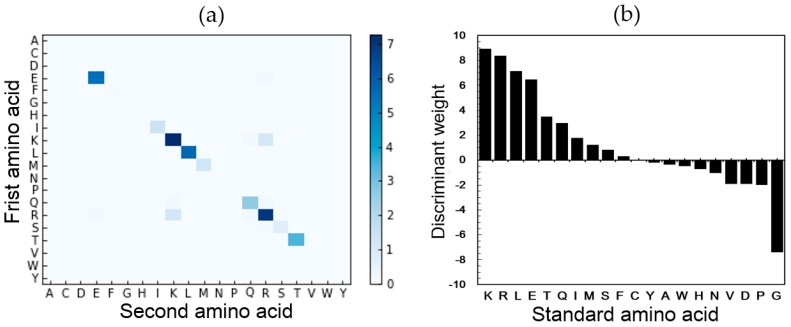

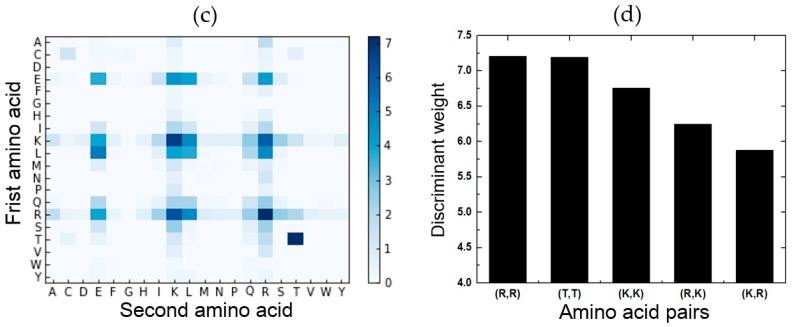
Feature analysis based on the features generated by PSFM-DBT. (**a**) The discriminant weights of the 400 features with *d* = 0. Each element in the figure represents the discriminant weight of the corresponding feature. The diagonal elements represent 20 features of amino acid composition. (**b**) The discriminant weights of the 20 amino acids according to amino acid composition. (**c**) The discriminant weights of the 400 standard amino acid pairs (*d* = 1, 2, 3, 4). Each element in the figure represents the sum of the discriminant weights of the corresponding bigrams, for example, the discriminant weight of bigrams (R, R) is W_(R, R)_ = W_(RR)_ + W_(R*R)_ + W_(R**R)_ + W_(R***R)_, where * represents mismatch. The x-axis and y-axis represent the second amino acid and first amino acid in a bigram, respectively. (**d**) The discriminant weights of the top five most discriminant bigrams, including (R, R), (T, T), (K, K), (R, K) and (K, R).

In this study, the feature analysis was based on the predictor PSFM-DBT (*D* = 4). The discriminative weights of the 2000 features were calculated by Equation (1). Then we analyzed the features of amino acid composition and the features of amino acid bigrams respectively. The discriminant weights of the 400 features with *d* = 0 were visualized by a heatmap shown in Figure 4a. The 20 elements in the diagonal represent the 20 features of amino acids composition, from which we can see that the amino acid K (Lys) has the highest weight value among all the 20 features, indicating that amino acid K is critical for predicting the DNA binding proteins. For further exploration, all the discriminant weights of all the 20 features of amino acid composition were shown in Figure 4b. We can see that 10 amino acids show positive discriminative weights, while the other 10 amino acids show negative discriminative weights. The top five most discriminative amino acids are K (Lys), R (Arg), L (Leu), E (Glu) and T (Thr). It has been reported that the positively charged amino acids (such as Arg and Lys) and the polar amino acids (such as Thr and Ser) are important for a protein binding with a DNA sequence, and the acidic amino acids, such as D (Asp) and E (Glu), show low propensity for the interaction of protein and DNA [32,33]. However, amino acid Glu show positive discriminative weights in Figure 4b indicating that the bigram composition is more accurate than the amino acid composition.

**Figure 5:**
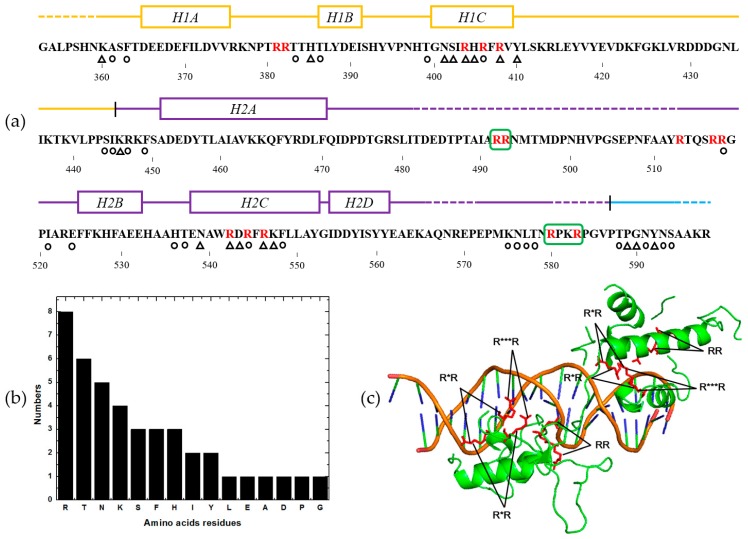
(**a**) The distributions of bigrams (R, R) in protein 1IGNB. The structural domains of this protein are color coded (orange represents domain 1, purple represents domain 2, and C-terminal tail is shown in blue). The open rectangles indicate the positions of helices, and broken lines mark regions of structural disorder. Residues interacting with DNA bases are indicated by triangles, and those contacting the phosphate backbone are indicated by circles. The two (R, R) bigrams shown in green rectangles are the two bigrams occurring in non-DNA-binding regions. (**b**) Histogram of the number of amino acid residues which binding with DNA in protein 1IGNB. (**c**) The distributions of bigrams (R, R) with different distances in the 3D structure of protein 1IGNB. The 3D structures of protein and DNA are shown in green and brown, respectively.

Then we analyzed the rest of the 1600 features of amino acid bigrams obtained by PSFM-DBT with *d* = 1, 2, 3, 4. The weight values of the same kinds of bigrams with different *d* values were summed, and the results are shown in Figure 4c. We can see from this figure, the top five most discriminative amino acid bigrams are (R, R), (T, T), (K, K), (R, K) and (K, R), whose discriminant weights were shown in Figure 4d. These results further confirmed that the importance of amino acid R (Arg), T (Thr) and K (Lys). Interestingly, this conclusion is fully consistent with previous studies [32,33,34,35]. A specific DNA-binding protein 1IGN chain B was selected as an example to further explore the importance of the features in PSFM-DBT. 1IGNB is known as the yeast RAP1, a multifunctional protein binding with the telomeric DNA in the yeast *S. cerevisiae* via a sequence-specific manner, it is also involved in transcriptional regulation [36]. As shown in Figure 4d, bigrams (R, R) have the highest weight values among all the four bigrams. There are four kinds of (R, R) bigrams, including RR, R*R, R**R and R***R (* represents mismatch) with distance *d* = 1, 2, 3, 4 respectively. The distributions of these bigrams in the protein sequence 1IGNB and its 3D structure were shown in Figure 5a,c, respectively, from which we can see that most of the (R, R) bigrams were located in the DNA binding regions, except that two occurred in the structural disordered regions, and all (R, R) bigrams occurred in the area close to DNA major grooves. Previous studies reported [23,34] that the arginine rich region is indeed critical for the protein helix, and DNA major groove interaction by a mechanism known as ‘phosphate bridging by an arginine-rich helix’. Moreover, we counted the numbers of these amino acid residues interacting with DNA in protein 1IGNB, the corresponding histogram is shown in Figure 5b, from which we can see that the positively charged amino acids (Arg, Lys and His) and the polar amino acids (Thr, Ser and Asn) are more likely to bind to DNA. This proved the correctness of the above conclusion, and explained the reason why the proposed PSFM-DBT predictor works well for DNA binding protein identification.

### 2.6. Web-Server Guide

We established an accessible web-server for the proposed PSFM-DBT predictor. Furthermore, for the convenience of the vast majority of experimental scientists, a step-by-step guide about how to use the web-server without the need to carefully understand the mathematical details was stated as follows.

**Figure 6:**
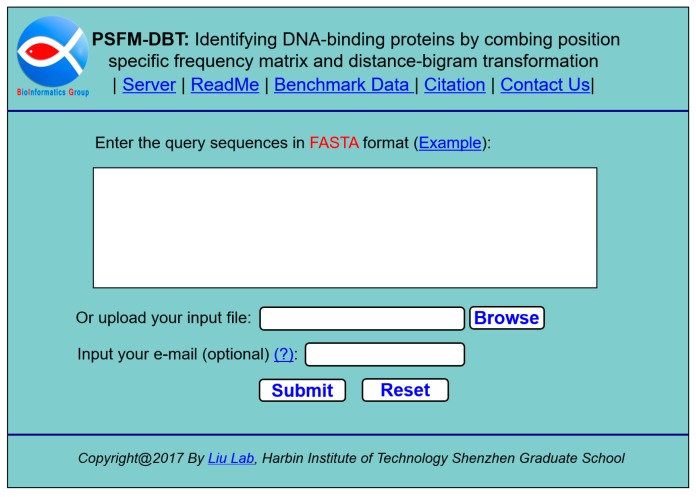
A semi-screenshot to show the home page of the web-server PSFM-DBT, which is available at http://bioinformatics.hitsz.edu.cn/PSFM-DBT/.

Step 1. Open the web-server at http://bioinformatics.hitsz.edu.cn/PSFM-DBT/ and you will see the home page of PSFM-DBT, as shown in Figure 6. Click on the “ReadMe” button to see a brief introduction of the server and the caveat when using it.

Step 2. You can input the query sequences into the input box or directly upload your input data via the “Browse” button. The input sequence should be in the FASTA format. The examples of sequences in the FASTA format could be shown in the input box by clicking the Example button right above the input box.

Step 3. Click on the “Submit” button to execute the recognition program, then the predicted results will be shown in a new page. For example, if you use the four example protein sequences as the input, you will see on your computer screen that the first and second query sequences are DNA-binding proteins. The third and fourth are non-DNA-binding proteins.

## 3. Methods and Materials

### 3.1. Dataset

The quality of the data set determines the quality of the research results. In the current study, we selected a widely used dataset PDB1075 [23] as the benchmark dataset. PDB1075 was constructed by Liu et al., which can be formulated as

(2) $$S=S^{+}\cup S^{-}$$

where $S^{+}$ is the subset of positive samples, $S^{-}$ is the subset of negative samples and the symbol ∪ represents the “union” in the set theory. These proteins were all extracted from Protein Data Bank (PDB) released at December 2013, where DNA-binding proteins were obtained by searching the mmCIF keyword of ‘DNA binding protein’ through the advanced search interface and non-DNA-binding proteins were obtained by randomly extracting from PDB. To construct a high quality and non-redundant benchmark dataset, these proteins were filtered strictly according to the following criteria. (1) Remove all the sequences which have less than 50 amino acids or contain character of ‘X’. (2) Using PISCES [37] to filter those sequences that have ≥25% pairwise sequence similarity to any other in the same subset. Finally, the subset $S^{+}$ consist of 525 DNA-binding proteins and the subset $S^{-}$ consists of 550 non-DNA-binding proteins.

### 3.2. Protein Representation

One of the most challenging problems in machine learning-based methods for computational biology is how to effectively represent a biological sequence with a discrete model [38,39,40], because all the existing machine learning algorithms [41], such as NN, SVM, RF, and KNN can only handle vector rather than protein sequences with different lengths. To solve this problem, many researchers have proposed various methods. Previous experimental results showed that evolutionary information can obviously improve the performance of predictors for identifying DNA-binding proteins. In order to incorporate the evolutionary information into the predictors, we employed four feature extraction methods to extract the evolutionary information from the Position Specific Frequency Matrix (PSFM) [42]. PSFM and the four methods will be introduced in more detail in the following sections.

#### 3.2.1. Position Specific Frequency Matrix

PSFM has been widely used in the field of predicting the structure and function of proteins [42,43]. Therefore, in this study, we employed the PSFM, which was generated by using PSI-BLAST [29] to search the target proteins against the non-redundant database NRDB90 [44] with default parameters, except the iteration and *e*-value were set as 10 and 0.001, respectively.

Given a protein sequence **P** with *L* amino acids, it can be formulated as:

(3) $$P=R_{1}R_{2}R_{3}R_{4}R_{5}\cdots R_{L}$$

where R_1_ represents the 1st residue, R_2_ the 2nd residue, and so forth.

The PSFM profile can be represented as a matrix with dimensions of 20 × *L* as follows:

(4) $$\mathrm{PSFM}=\left[\begin{array}{cccc} P_{1,1} & P_{1,2} & \cdots & P_{1,20}\\ P_{2,1} & P_{2,2} & \cdots & P_{2,20}\\ \vdots & \vdots & \ddots & \vdots \\ P_{L,1} & P_{L,2} & \cdots & P_{L,20} \end{array}\right]$$

where 20 represents the number of standard amino acids, and *L* is the length of the query protein sequence. The element *P_i,j_* represents the occurrence probability of amino acid *j* at position *i* of the protein sequence, the rows of matrix represent the positions of the sequence, and the columns of the matrix represent the 20 standard amino acids. The sum of elements in each row is 1.

#### 3.2.2. Residue Probing Transformation

RPT, first proposed by Jeong et al. [22], focuses on domains with similar conservation rates by grouping domain families based on their conservation scores in PSSM profiles. Because the idea is similar to the probe concept used in microarray technologies, it was called RPT. Each probe is a standard amino acid, and corresponds to a particular column in the PSFM profiles.

Given a PSFM (Equation (4)), it was divided into 20 groups according to 20 different standard amino acids, and for each group, we calculated the sum of the PSFM values in every column, leading to a feature vector of 20 dimension. Iteratively, for the 20 groups, the PSFM was translated into a Matrix **M** with 20 × 20 dimension, as follows:

(5) $$M=\left[\begin{array}{cccc} e_{1,1} & e_{1,2} & \cdots & e_{1,20}\\ e_{2,1} & e_{2,2} & \cdots & e_{2,20}\\ \vdots & \vdots & \ddots & \vdots \\ e_{20,1} & e_{20,2} & \cdots & e_{20,20} \end{array}\right]$$

The **M** was then transferred into a feature vector of 400 dimension, as follows:

(6) $$P=[f(e_{1,1})\;f(e_{1,2})\;\cdots \;f(e_{i,j})\;\cdots \;f(e_{20,20})]$$

where *f*(*e_i,j_*) was calculated by the following equation:

(7) $$f(e_{i,j})=\frac{e_{i,j}}{L}\;\left(i,j=1,2,\cdots ,20\right)$$

In this study, the amino acid composition of the 20 standard amino acids in PSFM was also incorporated into the RPT approach. As a result, the dimension of the corresponding feature vector is 400 + 20 = 420.

#### 3.2.3. Evolutionary Difference Transformation

EDT [3] is able to extract the information of the non-co-occurrence probability of two amino acids separated by a certain distance *d* in protein during the evolutionary process of the protein. The *d* is the distance between these two amino acids (*d* = 1, 2, …, *L*_min_ − 1, where *L*_min_ is the length of the shortest proteins in the benchmark dataset (Equation (2)). For example, *d* = 1 means the two amino acids are adjacent; *d* = 2 means there is one amino acid between the two amino acids; *d* = 3 means there are two amino acids between the two amino acids, and so forth.

For a given PSFM (Equation (4)), it can be transferred into a feature vector, as follows:

(8) $$P=[\psi_{1}\;\psi_{2}\;\cdots \;\psi_{k}\;\cdots \;\psi_{\Omega }]$$

where Ω is an integer reflecting the vector’s dimension, its value is *D* × 400; where *D* is the maximum value of *d*. The non-co-occurrence probability of two amino acids separated by distance *d* can be calculated by:

(9) $$f(A_{x},A_{y}|d)=\frac{1}{L-d}\sum_{i=1}^{L-d}{\left(P_{i,x}-P_{i+d,y}\right)}^{2}$$

where *P_i,x_* (*P_i+d,y_*) is the element in PSFM; A*_x_* and A*_y_* can be any of the 20 standard amino acids in the protein (Equation (3)).

Thus, each element in feature vector (Equation (8)) is obtained by

(10) $$\left\{ \begin{array}{l} \psi_{1}=f(A_{1},A_{1}|1)\\ \psi_{2}=f(A_{1},A_{2}|1)\\ \;\cdots \\ \psi_{400}=f(A_{20},A_{20}|1)\\ \;\cdots \\ \psi_{k}=f(A_{x},A_{y}|d)\\ \;\cdots \\ \psi_{\Omega }=f(A_{20},A_{20}|D) \end{array}\right.,\;(1\leq d\leq D)$$

#### 3.2.4. Distance-Bigram Transformation

DBT [17,23,24] calculate the occurrence frequency of a combination of two amino acids separated by a certain distance along the protein sequence. The distance *d* is determined by the number of amino acids between the two amino acids of bigram. Some previous studies [17,23,24] have reported that the occurrence frequencies of amino acid pairs can well capture characteristics of proteins and they worked well for protein functionality annotation. To capture the characteristics of DNA-binding proteins, we represented proteins by combining PSFM with distance-bigram transformation, which can transform PSFM into fixed length feature vector.

For a given PSFM (Equation (4)), it can be transferred into a feature vector, as follows:

(11) $$P=[\psi_{1}\;\psi_{2}\;\cdots \;\psi_{k}\;\cdots \;\psi_{\Omega }]$$

where Ω is an integer to reflect the vector’s dimension, its value is determined by *D* the maximum value of *d*. In order to incorporate the amino acid composition of the 20 standard amino acids in PSFM into the DBT approach, in this method, *d* = 0 was taken into account, therefore, Ω = 400 × *D* + 400.

The detail of DBT can be summarized mathematically as in the below equation.

(12) $$f(A_{x},A_{y}|d)=\frac{1}{L-d}\sum_{i=1}^{L-d}P_{i,x}P_{i+d,y}$$

where *P_i,x_* (*P_i+d,y_*) is the element of the PSFM matrix; *f*(A*_x_*,A*_y_*|*d*) represents the occurrence frequency of a bigram (standard amino acids A*_x_* and A*_y_* separated by a certain distance *d*) in evolutionary process.

Accordingly, each element in the feature vector (Equation (11)) is obtained by

(13) $$\left\{ \begin{array}{l} \psi_{1}=f(A_{1},A_{1}|0)\\ \psi_{2}=f(A_{1},A_{2}|0)\\ \;\cdots \\ \psi_{400}=f(A_{20},A_{20}|0)\\ \;\cdots \\ \psi_{k}=f(A_{x},A_{y}|d)\\ \;\cdots \\ \psi_{\Omega }=f(A_{20},A_{20}|D) \end{array}\right.,\;(0\leq d\leq D)$$

#### 3.2.5. Trigram Transformation

TT [25] is able to consider the local and global sequence-order effects by considering the trigrams along the protein sequences, the resulting feature vectors can be represented as:

(14) $$P=[\psi_{1}\;\psi_{2}\;\cdots \;\psi_{k}\;\cdots \;\psi_{8000}]$$

This technique can be summarized mathematically as shown in the below equation.

(15) $$f(A_{x},A_{y},A_{z})=\sum_{i=1}^{L-2}P_{i,x}P_{i+1,y}P_{i+2,z}$$

where *P_i,x_*, *P_i+1,y_* and *P_i+2,z_* represent the corresponding elements in PSFM (Equation (4)); A*_x_*, A*_y_* and A*_z_* can be any of the 20 standard amino acids in the protein (Equation (3)); *f*(A*_x_*, A*_y_*, A*_z_*) represents the occurrence frequency of trigram (A*_x_*A*_y_*A*_z_*) in evolutionary process.

Accordingly, each element in the feature vector (Equation (14)) is obtained by

(16) $$\left\{ \begin{array}{l} \psi_{1}=f(A_{1},A_{1},A_{1})\\ \psi_{2}=f(A_{1},A_{1},A_{2})\\ \;\cdots \\ \psi_{k}=f(A_{x},A_{y},A_{z})\\ \;\cdots \\ \psi_{8000}=f(A_{20},A_{20},A_{20}) \end{array}\right.,\;(x,y,x=1,2,\cdots ,20)$$

### 3.3. Support Vector Machine

SVM is a machine learning algorithm based on the structural-risk minimization principle of statistical learning theory. It was first presented by Vapnik [45] and has been widely used in bioinformatics. SVM is not only suitable for linear data, but also suitable for non-linear data. For linear data, SVM seek for an optimal hyper-plane to maximize the separation boundary between the positive instance and the negative instance, thereby separating the two instances. The nearest two points to the hyper-plane are called support vectors. For a non-linear model, SVM uses a non-linear transformation to map the input feature space to a high dimensional feature space where the samples can be well separated by an optimal hyper-plane. Kernel function is the most vital part for SVM; it determines the final performance of the SVM algorithm. There are some commonly used kernel functions for SVM, including Linear Function, Polynomial Function, Gaussian Function, Laplacian Function, Sigmoid Function and Radial Basis Function (RBF). SVM also can be used in the hierarchical classification [46]. Ensemble SVM may improve performance, too [47,48,49]. In the current study, an available SVM algorithm package called LIBSVM [50] was used to implement SVM algorithm, in which the RBF was chosen as the kernel function and the two parameters *c* and *g* were optimized by 5-fold cross validation on the benchmark.

### 3.4. Evaluation of Performance

In the current study, three commonly used methods were used to evaluate the performance of the proposed methods, including *k*-fold cross-validation, jackknife test and independent test. Moreover, sensitivity (SN), specificity (SP), accuracy (ACC), Matthew’s correlation coefficient (MCC), the Receiver Operating Characteristic (ROC) curve [51] and the area under ROC curve (AUC) were selected as evaluation criteria. These criteria have been widely used in various studies for biological sequence annotation. They can be mathematically defined as follows:

(17) $$\left\{ \begin{array}{l} \mathrm{SN}=\frac{\mathrm{TP}}{\mathrm{TP}+\mathrm{FN}}\\ \mathrm{SP}=\frac{\mathrm{TN}}{\mathrm{TN}+\mathrm{FP}}\\ \mathrm{ACC}=\frac{\mathrm{TP}+\mathrm{TN}}{\mathrm{TP}+\mathrm{FP}+\mathrm{TN}+\mathrm{FN}}\\ \mathrm{MCC}=\frac{\mathrm{TP}\times \mathrm{TN}-\mathrm{FP}\times \mathrm{FN}}{\sqrt{\left(\mathrm{TP}+\mathrm{FN})\times (\mathrm{TP}+\mathrm{FP})\times (\mathrm{TN}+\mathrm{FP})\times (\mathrm{TN}+\mathrm{FN}\right)}} \end{array}\right.$$

where TP is the number of true positive samples; TN is the number of true negative samples; FP is the number of false positive samples; and FN is the number of false negative samples. SN denote percentage of true positive samples among positive samples and SP denote percentage of true negative samples among negative samples. ACC represent the percentage of the samples which were correctly predicted among all samples. MCC explains the reliability of models, and its values range from −1 to 1, when MCC = −1 if all predictions are incorrect and when MCC = 1 if all predictions are correct. For MCC = 0, the prediction is no better than random. The ROC curve is a plot which is usually used to evaluate the performance of predictors. The AUC is the area under ROC curve which gives a measure of the quality of binary classification methods; the larger AUC, the better the predictive quality is.

## 4. Conclusions

To further improve the prediction accuracy and understand the binding regular patterns of DNA binding proteins, we explored and compared the performance of four feature extraction methods, including Residue Probing Transformation (RPT), Evolutionary Difference Transformation (EDT), Distance-Bigram Transformation (DBT), and Trigram Transformation (TT). Experimental results showed that PSFM-DBT achieved the best performance, and outperformed other existing methods in this field. This method was further evaluated on an independent dataset. Furthermore, some interesting patterns were discovered by analyzing the features generated PSFM-DBT, fully consistent with previous studies. Finally, a web server of the proposed PSFM-DBT predictor was established in order to help the users to use this method, which would be a useful tool for protein sequence analysis, especially for studying the structure and function of proteins. Future studies will focus on exploring advanced machine learning techniques to improve the performance of DNA binding protein prediction [52,53].

## Figures and Tables

**Table 1:** The results of the four Position Specific Frequency Matrix (PSFM)-based methods on the benchmark dataset.

Method	ACC (%)	MCC	AUC (%)	SN (%)	SP (%)
PSFM-RPT ^a^	78.88	0.5785	86.35	80.76	77.09
PSFM-EDT ^b^	79.35	0.5868	84.49	78.86	**79.82**
PSFM-DBT ^c^	**81.02**	**0.6224**	**87.12**	**84.19**	78.00
PSFM-TT ^d^	79.16	0.5840	85.54	80.95	77.45

The results were obtained by jackknife test on benchmark dataset with SVM algorithm. The bold numbers represent the best values of the corresponding evaluation criteria in this table. ^a^ The parameters were: *c* = 2^4^, *g* = 2^6^; ^b^ The parameters were: *D* = 7, *c* = 2^9^, *g* = 2^−2^; ^c^ The parameters were: *D* = 4, *c* = 2^3^, *g* = 2^5^; ^d^ The parameters were: *c* = 2^5^, *g* = 2^−9^.

**Table 2:** The performance of various methods on benchmark dataset.

Method	ACC (%)	MCC	AUC (%)	SN (%)	SP (%)
DNA-Prot	72.55	0.44	78.90	82.67	59.75
iDNA-Prot	75.40	0.50	76.10	83.81	64.73
DNAbinder (dimension 400)	73.58	0.47	81.50	66.47	80.36
DNAbinder (dimension 21)	73.95	0.48	81.40	68.57	79.09
PseDNA-Pro	76.55	0.53	N/A	79.61	73.63
iDNA-Prot|dis	77.30	0.54	82.60	79.40	75.27
iDNAPro-PseAAC	76.56	0.53	83.92	75.62	77.45
iDNA-KACC	75.16	0.50	83.00	77.52	72.90
PSSM-DT	79.96	0.62	86.50	78.00	**81.91**
Local-DPP	79.10	0.59	N/A	**84.80**	73.60
PSFM-DBT ^a^	**81.02**	**0.62**	**87.12**	84.19	78.00

The results of all methods in the table were obtained by jackknife validation on benchmark dataset. The bold numbers represent the best values of the corresponding evaluation criteria in this table. ^a^ See the footnote of Table 1.

**Table 3:** Performance of various methods on the independent dataset.

Method	ACC (%)	MCC	AUC (%)	SN (%)	SP (%)
DNA-Prot	61.80	0.240	N/A	69.90	53.80
iDNA-Prot	67.20	0.344	N/A	67.70	66.70
DNAbinder	60.80	0.216	60.70	57.00	64.50
DNABIND	67.70	0.355	69.40	66.70	68.80
DBPPred	76.90	0.538	79.10	79.60	74.20
iDNA-Prot|dis	72.00	0.445	78.60	79.50	64.50
iDNAPro-PseAAC-EL	71.50	0.442	77.80	82.80	60.2
iDNA-KACC-EL	79.03	0.611	81.40	**94.62**	63.44
PSSM-DT	80.00	**0.647**	87.40	87.09	72.83
Local-DPP	79.00	0.625	N/A	92.50	65.60
PSFM-TT	78.49	0.580	86.63	88.17	68.82
PSFM-RPT	79.57	0.594	85.67	84.95	**74.19**
PSFM-EDT	79.57	0.600	86.88	88.17	70.97
PSFM-DBT	**80.65**	0.624	**88.03**	90.32	70.97

The bold numbers represent the best values of the corresponding evaluation criteria in this table.

## Supplementary Materials

Supplementary materials can be found at www.mdpi.com/1422-0067/18/9/1856/s1. The benchmark dataset PDB1075 contains 525 DNA-binding proteins (positive samples) and 550 non-DNA-binding proteins (negative samples) (See Equation (2)), which is available at http://bioinformatics.hitsz.edu.cn/PSFM-DBT/data/.
