# Supplementary material for: PSFM-DBT: Identifying DNA-Binding Proteins by Combing Position Specific Frequency Matrix and Distance-Bigram Transformation
Source: Int J Mol Sci. 2017 Aug 25;18(9):1856. doi: 10.3390/ijms18091856 (PMC5618505; doi:10.3390/ijms18091856)
Supplement: Supplementary file 1 [file ijms-18-01856-s001.zip › Supp-S1/benchmark dataset.doc]

The benchmark dataset contains 1075 protein sequences, which are classified into subset with 525 DNA-binding proteins (positive samples) and subset with 550 non DNA-binding proteins (negative samples).

(1).525 DNA-binding proteins

>1AKHA

KKEKSPKGKSSISPQARAFLEEVFRRKQSLNSKEKEEVAKKCGITPLQVRVWFINKRMRSK

>1AOII

ATCAATATCCACCTGCAGATTCTACCAAAAGTGTATTTGGAAACTGCTCCATCAAAAGGCATGTTCAGCTGAATTCAGCTGAACATGCCTTTTGATGGAGCAGTTTCCAAATACACTTTTGGTAGAATCTGCAGGTGGATATTGAT

>1B6WA

MELPIAPIGRIIKDAGAERVSDDARITLAKILEEMGRDIASEAIKLARHAGRKTIKAEDIELAVRRFKK

>1C1KA

MIKLRMPAGGERYIDGKSVYKLYLMIKQHMNGKYDVIKYNWCMRVSDAAYQKRRDKYFFQKLSEKYKLKELALIFISNLVANQDAWIGDISDADALVFYREYIGRLKQIKFKFEEDIRNIYYFSKKVEVSAFKEIFEYNPKVQSSYIFKLLQSNIISFETFILLDSFLNIIDKHDEQTDNLVWNNYSIKLKAYRKILNIDSQKAKNVFIETVKSCKY

>1C6VX

QQSKNSKFKNFRVYYREGRDQLWKGPGELLWKGEGAVLLKVGTDIKVVPRRKAKIIKDYGGGKEVDSSSHMEDTGEAREVA

>1C6VD

IHGQVNSDLGTWQMDCTHLEGKIVIVAVHVASGFIEAEVIPQETGRQTALFLLKLAGRWPITHLHTDNGANFASQEVKMVAWWAGIEHTFGVPYNPQSQGVVEAMNHHLKNQIDRIREQANSVETIVLMAVHCMNHKRRGGIGDMTPAERLINMITTEQEIQFQ

>1CI4B

MTTSQKHRDFVAEPMGEKPVGSLAGIGEVLGKKLEERGFDKAYVVLGQFLVLKKDEDLFREWLKDTCGANAKQSRDCFGCLREWCDAFL

>1D4UA

MEFDYVICEECGKEFMDSYLMDHFDLPTCDDCRDADDKHKLITKTEAKQEYLLKDCDLEKREPPLKFIVKKNPHHSQWGDMKLYLKLQIVKRSLEVWGSQEALEEAKEVRQ

>1D8BA

ELNNLRMTYERLRELSLNLGNRMVPPVGNFMPDSILKKMAAILPMNDSAFATLGTVEDKYRRRFKYFKATIADLSKKRSSE

>1DMLG

MTDSPGGVAPASPVEDASDASLGQPEEGAPCQVVLQGAELNGILQAFAPLRTSLLDSLLVMGDRGILIHNTIFGEQVFLPLEHSQFSRYRWRGPTAAFLSLVDQKRSLLSVFRANQYPDLRRVELAITGQAPFRTLVQRIWTTTSDGEAVELASETLMKRELTSFVVLVPQGTPDVQLRLTRPQLTKVLNATGADSATPTTFELGVNGKFSVFTTSTCVTFAAREEGVSSSTSTQVQILSNALTKAGQAAANAKTVYGENTHRTFSVVVDDCSMRAVLRRLQVGGGTLKFFLTTPVPSLCVTATGPNAVSAVFLLKPQK

>1EE8B

PELPEVETTRRRLRPLVLGQTLRQVVHRDPARYRNTALAEGRRILEVDRRGKFLLFALEGGVELVAHLGMTGGFRLEPTPHTRAALVLEGRTLYFHDPRRFGRLFGVRRGDYREIPLLLRLGPEPLSEAFAFPGFFRGLKESARPLKALLLDQRLAAGVGNIYADEALFRARLSPFRPARSLTEEEARRLYRALREVLAEAVELGGSTLSDQSYRQPDGLPGGFQTRHAVYGREGLPCPACGRPVERRVVAGRGTHFCPTCQGEGP

>1EIJA

MRQQLEMQKKQIMMQILTPEARSRLANLRLTRPDFVEQIELQLIQLAQMGRVRSKITDEQLKELLKRVAGKKREIKISRK

>1F1EA

MAVELPKAAIERIFRQGIGERRLSQDAKDTIYDFVPTMAEYVANAAKSVLDASGKKTLMEEHLKALADVLMVEGVEDYDGELFGRATVRRILKRAGIERASSDAVDLYNKLICRATEELGEKAAEYADEDGRKTVQGEDVEKAITYSMPKGGEL

>1F2RI

MELSRGASAPDPDDVRPLKPCLLRRNHSRDQHGVAASSLEELRSKACELLAIDKSLTPITLVLAEDGTIVDDDDYFLCLPSNTKFVALACNEKWTYNDSD

>1F6VA

GSRIAKRTAINKTKKADVKAIADAWQINGEKELELLQQIAQKPGALRILNHSLRLAAMTAHGKGERVNEDYLRQAFRELDLDVDISTLLRN

>1G5HA

WLSGYAGPADGTQQPDAPEHAVAREALVDLCRRRHFLSGTPQQLSTAALLSGCHARFGPLGVELRKNLASQWWSSMVVFREQVFAVDSLHQEPGSSQPRDSAFRLVSPESIREILQDREPSKEQLVAFLENLLKTSGKLRATLLHGALEHYVNCLDLVNRKLPFGLAQIGVCFHPVSNSNQTPSSVTRVGEKTEASLVWFTPTRTSSQWLDFWLRHRLLWWRKFAMSPSNFSSADCQDELGRKGSKLYYSFPWGKEPIETLWNLGDQELLHTYPGNVSTIQGRDGRKNVVPCVLSVSGDVDLGTLAYLYDSFQLAENSFARKKSLQRKVLKLHPCLAPIKVALDVGKGPTVELRQVCQGLLNELLENGISVWPGYSETVHSSLEQLHSKYDEMSVLFSVLVTETTLENGLIQLRSRDTTMKEMMHISKLRDFLVKYLASASNVAAALDHHHHHH

>1GDTB

MRLFGYARVSTSQQSLDIQVRALKDAGVKANRIFTDKASGSSSDRKGLDLLRMKVEEGDVILVKKLDRLGRDTADMIQLIKEFDAQGVSIRFIDDGISTDGEMGKMVVTILSAVAQAERQRILERTNEGRQEAMAKGVVFGRKRKIDRDAVLNMWQQGLGASHISKTMNIARSTVYKVINESN

>1HCRA

GRPRAINKHEQEQISRLLEKGHPRQQLAIIFGIGVSTLYRYFPASSIKKRMN

>1HKQA

MVDNKVTQSNKLIESSHTLTLNEKRLVLCAASLIDSRKPLPKDGYLTIRADTFAEVFGIDVKHAYAALDDAATKLFNRDIRRYVKGKVVERMRWVFHVKYREGQGCVELGFSPTIIPHLTMLHKEFTSYQLK

>1HLVA

MGPKRRQLTFREKSRIIQEVEENPDLRKGEIARRFNIPPSTLSTILKNKRAILASERKYGVASTCRKTNKLSPYDKLEGLLIAWFQQIRAAGLPVKGIILKEKALRIAEELGMDDFTASNGWLDRFRRRRS

>1HQ3H

MSGRGKGGKGLGKGGAKRHRKVLRDNIQGITKPAIRRLARRGGVKRISGLIYEETRGVLKVFLENVIRDAVTYTEHAKRKTVTAMDVVYALKRQGRTLYGFGG

>1I11A

GSPHIKRPMNAFMVWAKDERRKILQAFPDMHNSNISKILGSRWKAMTNLEKQPYYEEQARLSKQHLEKYPDYKYKPRPKRT

>1IGNB

GALPSHNKASFTDEEDEFILDVVRKNPTRRTTHTLYDEISHYVPNHTGNSIRHRFRVYLSKRLEYVYEVDKFGKLVRDDDGNLIKTKVLPPSIKRKFSADEDYTLAIAVKKQFYRDLFQIDPDTGRSLITDEDTPTAIARRNMTMDPNHVPGSEPNFAAYRTQSRRGPIAREFFKHFAEEHAAHTENAWRDRFRKFLLAYGIDDYISYYEAEKAQNREPEPMKNLTNRPKRPGVPTPGNYNSAAKR

>1IN4A

MSEFLTPERTVYDSGVQFLRPKSLDEFIGQENVKKKLSLALEAAKMRGEVLDHVLLAGPPGLGKTTLAHIIASELQTNIHVTSGPVLVKQGDMAAILTSLERGDVLFIDEIHRLNKAVEELLYSAIEDFQIDIMIGKGPSAKSIRIDIQPFTLVGATTRSGLLSSPLRSRFGIILELDFYTVKELKEIIKRAASLMDVEIEDAAAEMIAKRSRGTPRIAIRLTKRVRDMLTVVKADRINTDIVLKTMEVLNIDDEGLDEFDRKILKTIIEIYRGGPVGLNALAASLGVEADTLSEVYEPYLLQAGFLARTPRGRIVTEKAYKHLKYEVPENRLF

>1IRZA

TAQKKPRVLWTHELHNKFLAAVDHLGVERAVPKKILDLMNVDKLTRENVASHLQKFRVALKKVS

>1IUFA

GIHMGKIKRRAITEHEKRALRHYFFQLQNRSGQQDLIEWFREKFGKDISQPSVSQILSSKYSYLDNTVEKPWDVKRNRPPKYPLLEAALFEWQVQQGDDATLSGETIKRAAAILWHKIPEYQDQPVPNFSNGWLEGFRKRHILH

>1IV6A

MTPEKHRARKRQAWLWEEDKNLRSGVRKYGEGNWSKILLHYKFNNRTSVMLKDRWRTMKKLKLISSDSED

>1IXCB

MEFRQLKYFIAVAEAGNMAAAAKRLHVSQPPITRQMQALEADLGVVLLERSHRGIELTAAGHAFLEDARRILELAGRSGDRSRAAARGDVGELSVAYFGTPIYRSLPLLLRAFLTSTPTATVSLTHMTKDEQVEGLLAGTIHVGFSRFFPRHPGIEIVNIAQEDLYLAVHRSQSGKFGKTCKLADLRAVELTLFPRGGRPSFADEVIGLFKHAGIEPRIARVVEDATAALALTMAGAASSIVPASVAAIRWPDIAFARIVGTRVKVPISCIFRKEKQPPILARFVEHVRRSAKD

>1IYMA

AMDDGVECAVCLAELEDGEEARFLPRCGHGFHAECVDMWLGSHSTCPLCRLTVVV

>1J2FB

GAMGSSLDNPTPFPNLGPSENPLKRLLVPGEEWEFEVTAFYRGRQVFQQTISCPEGLRLVGSEVGDRTLPGWPVTLPDPGMSLTDRGVMSYVRHVLSCLGGGLALWRAGQWLWAQRLGHCHTYWAVSEELLPNSGHGPDGEVPKDKEGGVFDLGPFIVDLITFTEGSGRSPRYALWFCVGESWPQDQPWTKRLVMVKVVPTCLRALVEMARVGGASSLENTVDLHISNSHPLSLTSDQYKAYLQDLVEGMDFQGPGES

>1JE5B

MAKKIFTSALGTAEPYAYIAKPDYGNEERGFGNPRGVYKVDLTIPNKDPRCQRMVDEIVKCHEEAYAAAVEEYEANPPAVARGKKPLKPYEGDMPFFDNGDGTTTFKFKCYASFQDKKTKETKHINLVVVDSKGKKMEDVPIIGGGSKLKVKYSLVPYKWNTAVGASVKLQLESVMLVELATFGGGEDDWADEVEENGYVASGSAK

>1JEQB

MVRSGNKAAVVLCMDVGFTMSNSIPGIESPFEQAKKVITMFVQRQVFAENKDEIALVLFGTDGTDNPLSGGDQYQNITVHRHLMLPDFDLLEDIESKIQPGSQQADFLDALIVSMDVIQHETIGKKFEKRHIEIFTDLSSRFSKSQLDIIIHSLKKCDISLQFFLPFSLGKEDGSGDRGDGPFRLGGHGPSFPLKGITEQQKEGLEIVKMVMISLEGEDGLDEIYSFSESLRKLCVFKKIERHSIHWPCRLTIGSNLSIRIAAYKSILQERVKKTWTVVDAKTLKKEDIQKETVYCLNDDDETEVLKEDIIQGFRYGSDIVPFSKVDEEQMKYKSEGKCFSVLGFCKSSQVQRRFFMGNQVLKVFAARDDEAAAVALSSLIHALDDLDMVAIVRYAYDKRANPQVGVAFPHIKHNYECLVYVQLPFMEDLRQYMFSSLKNSKKYAPTEAQLNAVDALIDSMSLAKKDEKTDTLEDLFPTTKIPNPRFQRLFQCLLHRALHPREPLPPIQQHIWNMLNPPAEVTTKSQIPLSKIKTLFPLIEAKKKDQVTAQEIFQDNHEDGPTAK

>1JEQA

MSGWESYYKTEGDEEAEEEQEENLEASGDYKYSGRDSLIFLVDASKAMFESQSEDELTPFDMSIQCIQSVYISKIISSDRDLLAVVFYGTEKDKNSVNFKNIYVLQELDNPGAKRILELDQFKGQQGQKRFQDMMGHGSDYSLSEVLWVCANLFSDVQFKMSHKRIMLFTNEDNPHGNDSAKASRARTKAGDLRDTGIFLDLMHLKKPGGFDISLFYRDIISIAEDEDLRVHFEESSKLEDLLRKVRAKETRKRALSRLKLKLNKDIVISVGIYNLVQKALKPPPIKLYRETNEPVKTKTRTFNTSTGGLLLPSDTKRSQIYGSRQIILEKEETEELKRFDDPGLMLMGFKPLVLLKKHHYLRPSLFVYPEESLVIGSSTLFSALLIKCLEKEVAALCRYTPRRNIPPYFVALVPQEEELDDQKIQVTPPGFQLVFLPFADDKRKMPFTEKIMATPEQVGKMKAIVEKLRFTYRSDSFENPVLQQHFRNLEALALDLMEPEQAVDLTLPKVEAMNKRLGSLVDEFKELVYPPDYNPEGKVTKRKHDNEGSGSKRPKVEYSEEELKTHISKGTLGKFTVPMLKEACRAYGLKSGLKKQELLEALTKHFQD

>1JSPB

GSHMRKKIFKPEELRQALMPTLEALYRQDPESLPFRQPVDPQLLGIPDYFDIVKNPMDLSTIKRKLDTGQYQEPWQYVDDVWLMFNNAWLYNRKTSRVYKFCSKLAEVFEQEIDPVMQSLG

>1K99A

MKKLKKHPDFPKKPLTPYFRFFMEKRAKYAKLHPEMSNLDLTKILSKKYKELPEKKKMKYIQDFQREKQEFERNLARFREDHPDLIQNAKKLEHHHHHH

>1KFTA

MGSSHHHHHHSSGLVPRGSHMNTSSLETIEGVGPKRRQMLLKYMGGLQGLRNASVEEIAKVPGISQGLAEKIFWSLKH

>1KIXA

MSTAAKQNRSTSRVSKKKTAAPKEGAAKKSDKGHKYEYVELAKASLTSAQPQHFYAVVIDATFPYKTNQERYICSLKIVDPTLYLKQQKGAGDASDYATLVLYAKRFEDLPIIHRAGDIIRVHRATLRLYNGQRQFNANVFYSSSWALFSTDKRSVTQEINNQDAVSDTTPFSFSSKHATIEKNEISILQNLRKWANQYFSSYSVISSDMYTALNKAQAQKGDFDVVAKILQVHELDEYTNELKLKDASGQVFYTLSLKLKFPHVRTGEVVRIRSATYDETSTQKKVLILSHYSNIITFIQSSKLAKELRAKIQDDHSVEVASLKKNVSLNAVVLTEVDKKHAALPSTSLQDLFHHADSDKELQAQDTFRTQFYVTKIEPSDVKEWVKGYDRKTKKSSSLKGASGKGDNIFQVQFLVKDASTQLNNNTYRVLLYTQDGLGANFFNVKADNLHKNADARKKLEDSAELLTKFNSYVDAVVERRNGFYLIKDTKLIY

>1KKXA

MRGSGSHHHHHHGSNNKQYELFMKSLIENCKKRNMPLQSIPEIGNRKINLFYLYMLVQKFGGADQVTRTQQWSMVAQRLQISDYQQLESIYFRILLPYERHMISQEGIKETQAKRILQPSLIS

>1KN0A

MSGTEEAILGGRDSHPAAGGGSVLCFGQCQYTAEEYQAIQKALRQRLGPEYISSRMAGGGQKVCYIEGHRVINLANEMFGYNGWAHSITQQNVDFVDLNNGKFYVGVCAFVRVQLKDGSYHEDVGYGVSEGLKSKALSLEKARKEAVTDGLKRALRSFGNALGNCILDKDYLRSLNKLPRQLPLEVDLTKAKRQDLEPSVEEARYNSCRPNM

>1KNUB

LNPESADLRALAKHLYDSYIKSFPLTKAKARAILTGKTTDKSPFVIYDMNSLMMGEDKIKFKHITPLQEQSKEVAIRIFQGCQFRSVEAVQEITEYAKSIPGFVNLDLNDQVTLLKYGVHEIIYTMLASLMNKDGVLISEGQGFMTREFLKSLRKPFGDFMEPKFEFAVKFNALELDDSDLAIFIAVIILSGDRPGLLNVKPIEDIQDNLLQALELQLKLNHPESSQLFAKLLQKMTDLRQIVTEHVQLLQVIKKTETDMSLHPLLQEIYKDLY

>1KU9B

MIIMEEAKKLIIELFSELAKIHGLNKSVGAVYAILYLSDKPLTISDIMEELKISKGNVSMSLKKLEELGFVRKVWIKGERKNYYEAVDGFSSIKDIAKRKHDLIAKTYEDLKKLEEKCNEEEKEFIKQKIKGIERMKKISEKILEALNDLDN

>1KW4A

METKRVNGTDRPPISSWSVDDVSNFIRELPGCQDYVDDFIQQEIDGQALLRLKEKHLVNAMGMKLGPALKIVAKVESIKEVRDHHHHHH

>1KZYD

ALEEQRGPLPLNKTLFLGYAFLLTMATTSDKLASRSKLPDGPTGSSEEEEEFLEIPPFNKQYTESQLRAGAGYILEDFNEAQCNTAYQCLLIADQHCRTRKYFLCLASGIPCVSHVWVHDSCHANQLQNYRNYLLPAGYSLEEQRILDWQPRENPFQNLKVLLVSDQQQNFLELWSEILMTGGAASVKQHHSSAHNKDIALGVFDVVVTDPSCPASVLKCAEALQLPVVSQEWVIQCLIVGERIGFKQHPKYKHDYVSH

>1L3AD

MASMTGGQQMGRGSDYFEPQQQQQQQQQQPQGASTPKVFVGYSIYKGKAALTVEPRSPEFSPLDSGAFKLSREGMVMLQFAPAAGVRQYDWSRKQVFSLSVTEIGSIISLGTKDSCEFFHDPNKGRSDEGRVRKVLKVEPLPDGSGHFFNLSVQNKLINLDENIYIPVTKAEFAVLVSAFNFVMPYLLGWHTAVNSFKPEDASRSNNANPRSGAELEWNLEHHHHHH

>1L8YA

MGKLPESPKRAEEIWQQSVIGDYLARFKNDRVKALKAMEMTWNNMEKKEKLMWIKKAAEDQKRYERELSEMRAPPAATNSSKKLEHHHHHH

>1MH3A

KIEEGKLVIWINGDKGYNGLAEVGKKFEKDTGIKVTVEHPDKLEEKFPQVAATGDGPDIIFWAHDRFGGYAQSGLLAEITPDKAFQDKLYPFTWDAVRYNGKLIAYPIAVEALSLIYNKDLLPNPPKTWEEIPALDKELKAKGKSALMFNLQEPYFTWPLIAADGGYAFKYENGKYDIKDVGVDNAGAKAGLTFLVDLIKNKHMNADTDYSIAEAAFNKGETAMTINGPWAWSNIDTSKVNYGVTVLPTFKGQPSKPFVGVLSAGINAASPNKELAKEFLENYLLTDEGLEAVNKDKPLGAVALKSYEEELAKDPRIAATMENAQKGEIMPNIPQMSAFWYAVRTAVINAASGRQTVDAALAAAQTAAAAAISPQARAFLEQVFRRKQSLNSKEKEEVAKKCGITPLQVRVWFINKRMRSK

>1MOJA

MSTQKNARATAGEVEGSDALRMDADRAEQCVDALNADLANVYVLYHQLKKHHWNVEGAEFRDLHLFLGEAAETAEEVADELAERVQALGGVPHASPETLQAEASVDVEDEDVYDIRTSLANDMAIYGDIIEATREHTELAENLGDHATAHMLREGLIELEDDAHHIEHYLEDDTLVTQGALE

>1MP9B

YIIPDEIPYKAVVNIENIVATVTLDQTLDLYAMERSVPNVEYDPDQFPGLIFRLESPKITSLIFKSGKMVVTGAKSTDELIKAVKRIIKTLKKYGMQLTGKPKIQIQNIVASANLHVIVNLDKAAFLLENNMYEPEQFPGLIYRMDEPRVVLLIFSSGKMVITGAKREDEVHKAVKKIFDKLVELDCVKPVEEEELEF

>1MSZA

MGSLNGGSPEGVESQDGVDHFRAMIVEFMASKKMQLEFPPSLNSHDRLRVHQIAEEHGLRHDSSGEGKRRFITVSKRAGSHHHHHH

>1N1JB

GSHMEEIRNLTVKDFRVQELPLARIKKIMKLDEDVKMISAEAPVLFAKAAQIFITELTLRAWIHTEDNKRRTLQRNDIAMAITKFDQFDFLIDIVPR

>1N1JA

SFREQDIYLPIANVARIMKNAIPQTGKIAKDAKECVQECVSEFISFITSEASERCHQEKRKTINGEDILFAMSTLGFDSYVEPLKLYLQKFRE

>1NGNA

ALSPPRRKSFKKWTPPRSPFNLVQEILFHDPWKLLIATIFLNRTSGKMAIPVLWEFLEKYPSAEVARAADWRDVSELLKPLGLYDLRAKTIIKFSDEYLTKQWRYPIELHGIGKYGNDSYRIFCVNEWKQVHPEDHKLNKYHDWLWENHEKLSLS

>1NK2P

ASDGLPNKKRKRRVLFTKAQTYELERRFRQQRYLSAPEREHLASLIRLTPTQVKIWFQNHRYKTKRAQNEKGYEGHP

>1NZPA

MAQPSSQKATNHNLHITEKLEVLAKAYSVQGDKWRALGYAKAINALKSFHKPVTSYQEACSIPGIGKRMAEKIIEILESGHLRKLDH

>1O57A

MKFRRSGRLVDLTNYLLTHPHELIPLTFFSERYESAKSSISEDLTIIKQTFEQQGIGTLLTVPGAAGGVKYIPKMKQAEAEEFVQTLGQSLANPERILPGGYVYLTDILGKPSVLSKVGKLFASVFAEREIDVVMTVATKGIPLAYAAASYLNVPVVIVRKDNKVTEGSTVSINYVSGSSNRIQTMSLAKRSMKTGSNVLIIDDFMKAGGTINGMINLLDEFNANVAGIGVLVEAEGVDERLVDEYMSLLTLSTINMKEKSIEIQNGNFLRFFKDNLLKNGETESHHHHHH

>1OQJB

GAMEDMEIAYPITCGESKAILLWKKFVCPGINVKCVKFNDQLISPKHFVHLAGKSTLKDWKRAIRLGGIMLRKMMDSGQIDFYQHDKVCSNTCRSTK

>1OSVB

AELTVDQQTLLDYIMDSYSKQRMPQEITNKILKEEFSAEENFLILTEMATSHVQILVEFTKRLPGFQTLDHEDQIALLKGSAVEAMFLRSAEIFNKKLPAGHADLLEERIRKSGISDEYITPMFSFYKSVGELKMTQEEYALLTAIVILSPDRQYIKDREAVEKLQEPLLDVLQKLCKIYQPENPQHFACLLGRLTELRTFNHHHAEMLMSWRVNDHKFTPLLCEIWDVQ

>1OY3D

VFGYVTEDGDTALHLAVIHQHEPFLDFLLGFSAGHEYLDLQNDLGQTALHLAAILGEASTVEKLYAAGAGVLVAERGGHTALHLACRVRAHTCACVLLQPRPSHPRDASDTYLTQSQDCTPDTSHAPAAVDSQPNPENEEEPRDEDWRLQLEAENYDGHTPLHVAVIHKDAEMVRLLRDAGADLNKPEPTCGRTPLHLAVEAQAASVLELLLKAGADPTARMYGGRTPLGSALLRPNPILARLLRAHGAPEPEDGGDKLSPCSSSGSDSDSDNRDEGDEYDD

>1OY3B

TAELKICRVNRNSGSCLGGDEIFLLCDKVQKEDIEVYFTGPGWEARGSFSQADVHRQVAIVFRTPPYADPSLQAPVRVSMQLRRPSDRELSEPMEFQYLPDTDDRHRIEEKRKRTYETFKSIMKKSPFNGPTEPRP

>1P1AA

GSHMQVTLKTLQQQTFKIDIDPEETVKALKEKIESEKGKDAFPVAGQKLIYAGKILNDDTALKEYKIDEKNFVVVMVTKPKAVST

>1P4EC

SQFDILCKTPPKVLVRQFVERFERPSGEKIASCAAELTYLCWMITHNGTAIKRATFMSYNTIISNSLSFDIVNKSLQFKYKTQKATILEASLKKLIPAWEFTIIPYNGQKHQSDITDIVSSLQLQFESSEEADKGNSHSKKMLKALLSEGESIWEITEKILNSFEYTSRFTKTKTLYQFLFLATFINCGRFSDIKNVDPKSFKLVQNKYLGVIIQCLVTETKTSVSRHIYFFSARGRIDPLVYLDEFLRNSEPVLKRVNRTGNSSSNKQEYQLLKDNLVRSYNKALKKNAPYPIFAIKNGPKSHIGRHLMTSFLSMKGLTELTNVVGNFSDKRASAVARTTYTHQITAIPDHYFALVSRYYAYDPISKEMIALKDETNPIEEWQHIEQLKGSAEGSIRYPAWNGIISQEVLDYLSSYINRRIGHHHHHH

>1P4WA

MRGSHHHHHHGSYTPESVAKLLEKISAGGYGDKRLSPKESEVLRLFAEGFLVTEIAKKLNRSIKTISSQKKSAMMKLGVDNDIALLNYLSSVSMTPVDK

>1P92A

MKDLVDTTEMYLRTIYELEEEGVTPLRARIAERLEQSGPTVSQTVARMERDGLVVVASDRSLQMTPTGRTLATAVMRKARLAERLLTDIIGLDINKVHDEACRWEHVMSDEVERRLVKVLKDVSRSPFGNPIPGLDELGVGNSDAAAPGTRVIDAATSMPRKVRIVQINEIFQVETDQFTQLLDADIRVGSEVEIVDRDGHITLSHNGKDVELLDDLAHTIRIEEL

>1PGZA

SKSESPKEPEQLRKLFIGGLSFETTDESLRSHFEQWGTLTDCVVMRDPNTKRSRGFGFVTYATVEEVDAAMNARPHKVDGRVVEPKRAVSREDSQRPGAHLTVKKIFVGGIKEDTEEHHLRDYFEQYGKIEVIEIMTDRGSGKKRGFAFVTFDDHDSVDKIVIQKYHTVNGHNCEVRKALSKQEMASASSSQRGR

>1PH1B

PQQQSAFKQLYTELFNNEGDFSKVSSNLKKPLKCYVKESYPHFLVTDGYFFVAPYFTKEAVNEFHAKFPNVNIVDLTDKVIVINNWSLELRRVNSAEVFTSYANLEARLIVHSFKPNLQERLNPTRYPVNLFRDDEFKTTIQHFRHTALQAAINKTVKGDNLVDISKVADAAGKKGKVDAGIVKASASKGDEFSDFSFKEGNTATLKIADIFVQEKG

>1PL5S

SNTTEILTSVDVLGTHSQTGTQQSNMYTSTQKTELEIDNKDSVTECSKDMKEDGLSFVDIVLSKAASALDEKEKQLAVANEIIRSLSDEVMRNEIRITSLQGDLTFTKKCLENARSQISEKDAKINKLMEKDFQVNKEIKPY

>1POGA

RGSHMRRRKKRTSIETNIRVALEKSFLENQKPTSEEITMIADQLNMEKEVIRVWFCNRRQKEKRIDI

>1PVEA

GSHMPLEFLRNQPQFQQMRQIIQQNPSLLPALLQQIGRENPQLLQQISQHQEHFIQMLNEPVQEAGGQGGGG

>1Q1VA

DEPLIKKLKKPPTDEELKETIKKLLASANLEEVTMKQICKKVYENYPTYDLTERKDFIKTTVKELISLEH

>1Q87B

PVNTKRSNGTKRVEFPTTKKSMCIGNSTPNEQETFRAKVDEIWFRLTQKTDGTVMRDFLIEKAAEYFKQPEQPKQNAIEVISAIMAPQEEQTKSKADLYKFLAMFGPYETIMLKIASLLLISNNKGHWLTFDPQAEKNANNQRDSISGWFDQNEPNCLILKTPTGIRKIWNKPLIEATGQYLMDENGEKYDSWDKYFEMKPIETYLTAYPTFAPMHHHHHH

>1QZGB

GPGGEDVIDSLQLNELLNAGEYKIGELTFQSIRSSQELQKKNTIVNLFGIVKDFTPSRQSLHGTKDWVTTVYLWDPTCDTSSIGLQIHLFSKQGNDLPVIKQVGQPLLLHQITLRSYRDRTQGLSKDQFRYALWPDFSSNSKDTLCPQPMPRLMKTGDKEEQFALLLNKIWDEQTNKHKNGELLSTS

>1QZQB

MEEYMPTEHHHHHHENLYFQGTSGEGQDIWDMLDKGNPFQFYLTRVSGVKPKYNSGALHIKDILSPLFGTLVSSAQFNYCFDVDWLVKQYPPEFRKKPILLVHGDKREAKAHLHAQAKPYENISLCQAKLDIAFGTHHTKMMLLLYEEGLRVVIHTSNLIHADWHQKTQGIWLSPLYPRIADGTHKSGESPTHFKADLISYLMAYNAPSLKEWIDVIHKHDLSETNVYLIGSTPGRFQGSQKDNWGHFRLKKLLKDHASSMPNAESWPVVGQFSSVGSLGADESKWLCSEFKESMLTLGKESKTPGKSSVPLYLIYPSVENVRTSLEGYPAGGSLPYSIQTAEKQNWLHSYFHKWSAETSGRSNAMPHIKTYMRPSPDFSKIAWFLVTSANLSKAAWGALEKNGTQLMIRSYELGVLFLPSAFGLDSFKVKQKFFAGSQEPMATFPVPYDLPPELYGSKDRPWIWNIPYVKAPDTHGNMWVPS

>1R5KC

MDPMIKRSKKNSLALSLTADQMVSALLDAEPPILYSEYDPTRPFSEASMMGLLTNLADRELVHMINWAKRVPGFVDLTLHDQVHLLECAWLEILMIGLVWRSMEHPGKLLFAPNLLLDRNQGKCVEGMVEIFDMLLATSSRFRMMNLQGEEFVCLKSIILLNSGVYTFLSSTLKSLEEKDHIHRVLDKITDTLIHLMAKAGLTLQQQHQRLAQLLLILSHIRHMSNKGMEHLYSMKCKNVVPLYDLLLEMLDAHRLHAPTS

>1RH6B

MYLTLQEWNARQRRPRSLETVRRWVRESRIFPPPVKDGREYLFHESAVKVDLNRP

>1RI7A

MGSSHHHHHHSSGLVPRGSHMRVPLDEIDKKIIKILQNDGKAPLREISKITGLAESTIHERIRKLRESGVIKKFTAIIDPEALGYSMLAFILVKVKAGKYSEVASNLAKYPEIVEVYETTGDYDMVVKIRTKNSEELNNFLDLIGSIPGVEGTHTMIVLKTHKETTELPIK

>1RIFB

MDIKVHFHDFSHVRIDCEESTFHELRDFFSFEADGYRFNPRFRYGNWDGRIRLLDYNRLLPFGLVGQIKKFCDNFGYKAWIDPQINEKEELSRKDFDEWLSKLEIYSGNKRIEPHWYQKDAVFEGLVNRRRILNLPTSAGRSLIQALLARYYLENYEGKILIIVPTTALTTQMADDFVDYRLFSHAMIKKIGGGASKDDKYKNDAPVVVGTWQTVVKQPKEWFSQFGMMMNDECHLATGKSISSIISGLNNCMFKFGLSGSLRDGKANIMQYVGMFGEIFKP

>1RW2A

MHHHHHHKLKTEQGGAHFSVSSLAEGSVTSVGSVNPAENFRVLVKQKKASFEEASNQLINHIEQFLDTNETPYFMKSIDCIRAFREEAIKFSEEQRFNNFLKALQEKVEIKQLNHFWEIVVQDGITLITKEEASGSSVTAEEAKKFLAPKDK

>1S6MA

MLSHMVLTRQDIGRAASYYEDGADDYYAKDGDASEWQGKGAEELGLSGEVDSKRFRELLAGNIGEGHRIMRSATRQDSKERIGLDLTFSAPKSVSLQALVAGDAEIIKAHDRAVARTLEQAEARAQARQKIQGKTRIETTGNLVIGKFRHETSRERDPQLHTHAVILNMTKRSDGQWRALKNDEIVKATRYLGAVYNAELAHELQKLGYQLRYGKDGNFDLAHIDRQQIEGFSKRTEQIAEWYAARGLDPNSVSLEQKQAAKVLSRAKKTSVDREALRAEWQATAKELGIDFS

>1SD4B

MTNKQVEISMAEWDVMNIIWDKKSVSANEIVVEIQKYKEVSDKTIRTLITRLYKKEIIKRYKSENIYFYSSNIKEDDIKMKTAKTFLNKLYGGDMKSLVLNFAKNEELNNKEIEELRDILNDISKK

>1SE8A

MARGMNHVYLIGALARDPELRYTGNGMAVFEATVAGEDRVIGNDGRERNLPWYHRVSILGKPAEWQAERNLKGGDAVVVEGTLEYRQWEAPEGGKRSAVNVKALRMEQLGTQPELIQDAGGGVRMSGAMNEVLVLGNVTRDPEIRYTPAGDAVLSLSIAVNENYQDRQGQRQEKVHYIDATLWRDLAENMKELRKGDPVMIMGRLVNEGWTDKDGNKRNSTRVEATRVEALARGAGNANSGYAAATPAAPRTQTASSAARPTSGGYQSQPSRAANTGSRSGGLDIDQGLDDFPPEEDDLPF

>1SFUB

MDLLSCTVNDAEIFSLVKKEVLSLNTNDYTTAISLSNRLKINKKKINQQLYKLQKEDTVKMVPSNPPKWFKNYNC

>1SQ8A

MLMGERIRARRIQLGLNQAELAQKVGVDQQAIEQLENGKAKRPRFLPELARALGVAVDWLLNGA

>1T0FC

GSAIKVVKPSDWDSLPDTDLRYIYSQRQPEKTMHERLKGKGVIVDMASLFKQAG

>1T0FA

GSAMAKANSSFSEVQIARRIKEGRGQGHGKDYIPWLTVQEVPSSGRSHRIYSHKTGRVHHLLSDLELAVFLSLEWESSVLDIREQFPLLPSDTRQIAIDSGIKHPVIRGVDQVMSTDFLVDCKDGPFEQFAIQVKPAAALQDERTLEKLELERRYWQQKQIPWFIFTDKEINPVVKENIEWLYSVKTEEVSAELLAQLSPLAHILQEKGDENIINVCKQVDIAYDLELGKTLSEIRALTANGFIKFNIYKSFRANKCADLCISQVVNMEELRYVAN

>1T23A

SNTRNFVLRDEDGNEHGVFTGKQPRQAALKAANRGSGTKANPDIIRLRERGTKKVHVFKAWKEIVDAPKNRPAWMPEKISKPFVKKERIEKLE

>1U2WD

MKKKDTCEIFGYDEEKVNRIQGDLQTVDISGVSQILKAIADENRAKITYALCQDEELCVCDIANILGVTIANASHHLRTLYKQGVVNFRKEGKLALYSLGDEHIRQIMMIALAHKKEVKVNV

>1U3EM

MEWKDIKGYEGHYQVSNTGEVYSIKSGKTLKHQIPKDGYHRIGLFKGGKGKTFQVHRLVAIHFCEGYEEGLVVDHKDGNKDNNLSTNLRWVTQKINVENQMSRGTLNVSKAQQIAKIKNQKPIIVISPDGIEKEYPSTKCACEELGLTRGKVTDVLKGHRIHHKGYTFRYKLNG

>1U78A

MPRGSALSDTERAQLDVMKLLNVSLHEMSRKISRSRHCIRVYLKDPVSYGTSKRAPRRKALSVRDERNVIRAASNSCKTARDIRNELQLSASKRTILNVIKRSGVIVRQKLRPAPLLSADHKLKRLEFAKNNMGTHHHHHH

>1U9NA

MGSSHHHHHHSSGLVPRGSHVTTSAASQASLPRGRRTARPSGDDRELAILATAENLLEDRPLADISVDDLAKGAGISRPTFYFYFPSKEAVLLTLLDRVVNQADMALQTLAENPADTDRENMWRTGINVFFETFGSHKAVTRAGQAARATSVEVAELWSTFMQKWIAYTAAVIDAERDRGAAPRTLPAHELATALNLMNERTLFASFAGEQPSVPEARVLDTLVHIWVTSIYGENR

>1UB4C

GPHMIHSSVKRWGNSPAVRIPATLMQALNLNIDDEVKIDLVDGKLIIEPVRKEPVFTLAELVNDITPENLHENIDWGEPKDKEVW

>1UB4B

VSRYVPDMGDLIWVDFDPTKGSEQAGHRPAVVLSPFMYNNKTGMCLCVPCTTQSKGYPFEVVLSGQERDGVALADQVKSIAWRARGATKKGTVAPEELQLIKAKINVLIG

>1UDVB

MTEKLNEIVVRKTKNVEDHVLDVIVLFNQGIDEVILKGTGREISKAVDVYNSLKDRLGDGVQLVNVQTGSEVRDRRRISYILLRLKRVY

>1UFID

GSHMPVPSFGEAMAYFAMVKRYLTSFPIDDRVQSHILHLEHDLVHVTRKNHARQAGVRGLGHQS

>1UKLF

RSSINDKIIELKDLVMGTDAKMHKSGVLRKAIDYIKYLQQVNHKLRQENMVLKLANQKNKL

>1UKLB

MELITILEKTVSPDRLELEAAQKFLERAAVENLPTFLVELSRVLANPGNSQVARVAAGLQIKNSLTSKDPDIKAQYQQRWLAIDANARREVKNYVLQTLGTETYRPSSASQCVAGIACAEIPVSQWPELIPQLVANVTNPNSTEHMKESTLEAIGYICQDIDPEQLQDKSNEILTAIIQGMRKEEPSNNVKLAATNALLNSLEFTKANFDKESERHFIMQVVCEATQCPDTRVRVAALQNLVKIMSLYYQYMETYMGPALFAITIEAMKSDIDEVALQGIEFWSNVCDEEMDLAIEASEAAEQGRPPEHTSKFYAKGALQYLVPILTQTLTKQDENDDDDDWNPCKAAGVCLMLLSTCCEDDIVPHVLPFIKEHIKNPDWRYRDAAVMAFGSILEGPEPNQLKPLVIQAMPTLIELMKDPSVVVRDTTAWTVGRICELLPEAAINDVYLAPLLQCLIEGLSAEPRVASNVCWAFSSLAEAAYEAADVADDQEEPATYCLSSSFELIVQKLLETTDRPDGHQNNLRSSAYESLMEIVKNSAKDCYPAVQKTTLVIMERLQQVLQMESHIQSTSDRIQFNDLQSLLCATLQNVLRKVQHQDALQISDVVMASLLRMFQSTAGSGGVQEDALMAVSTLVEVLGGEFLKYMEAFKPFLGIGLKNYAEYQVCLAAVGLVGDLCRALQSNILPFCDEVMQLLLENLGNENVHRSVKPQILSVFGDIALAIGGEFKKYLEVVLNTLQQASQAQVDKSDFDMVDYLNELRESCLEAYTGIVQGLKGDQENVHPDVMLVQPRVEFILSFIDHIAGDEDHTDGVVACAAGLIGDLCTAFGKDVLKLVEARPMIHELLTEGRRSKTNKAKTLATWATKELRKLKNQA

>1UL1Z

GIQGLAKLIADVAPSAIRENDIKSYFGRKVAIDASMSIYQFLIAVRQGGDVLQNEEGETTSHLMGMFYRTIRMMENGIKPVYVFDGKPPQLKSGELAKRSERRAEAEKQLQQAQAAGAEQEVEKFTKRLVKVTKQHNDECKHLLSLMGIPYLDAPSEAEASCAALVKAGKVYAAATEDMDCLTFGSPVLMRHLTASEAKKLPIQEFHLSRILQELGLNQEQFVDLCILLGSDYCESIRGIGPKRAVDLIQKHKSIEEIVRRLDPNKYPVPENWLHKEAHQLFLEPEVLDPESVELKWSEPNEEELIKFMCGEKQFSEERIRSGVKRLSKSRQGSTQGRLDDFFKVTGSLSSAKRKEPEPKGSTKKKAKTGAAGKFKRGK

>1UL4A

GSSGSSGLRLCQVDRCTADMKEAKLYHRRHKVCEVHAKASSVFLSGLNQRFCQQCSRFHDLQEFDEAKRSCRRRLAGHNERRRKSSGESGPSSG

>1ULYA

MAKKVKVITDPEVIKVMLEDTRRKILKLLRNKEMTISQLSEILGKTPQTIYHHIEKLKEAGLVEVKRTEMKGNLVEKYYGRTADVFYINLYLGDEELRYIARSRLKTKIDIFKRLGYQFEENELLNIMDRMSQKEFDATVRISKYIEEKEDALKDFSNEDIIHAIEWLSTAELARDEEYLELLKRLGSILKR

>1USTA

KKEEASSKSYRELIIEGLTALKERKGSSRPALKKFIKENYPIVGSASNFDLYFNNAIKKGVEAGDFEQPKGPAGAVKLAKKKSPEVKKEKEVS

>1UVHD

MTSFTIPGLSDKKASDVADLLQKQLSTYNDLHLTLKHVHWNVVGPNFIGVHEMIDPQVELVRGYADEVAERIATLGKSPKGTPGAIIKDRTWDDYSVERDTVQAHLAALDLVYNGVIEDTRKSIEKLEDLDLVSQDLLIAHAGELEKFQWFVRAHLESAGGQLTHEGQSTEKGAADKARRKSA

>1V63A

GSSGSSGPKKPPMNGYQKFSQELLSNGELNHLPLKERMVEIGSRWQRISQSQKEHYKKLAEEQQRQYKVHLDLWVKSLSPQDRAAYKEYISNKRKSGPSSG

>1VJFA

MGSDKIHHHHHHMKTRADLFAFFDAHGVDHKTLDHPPVFRVEEGLEIKAAMPGGHTKNLFLKDAKGQLWLISALGETTIDLKKLHHVIGSGRLSFGPQEMMLETLGVTPGSVTAFGLINDTEKRVRFVLDKALADSDPVNFHPLKNDATTAVSQAGLRRFLAALGVEPMIVDFAAMEVVG

>1WEOA

GSSGSSGPKPLKNLDGQFCEICGDQIGLTVEGDLFVACNECGFPACRPCYEYERREGTQNCPQCKTRYKRLRGSPRVEGDEDEEDIDSGPSSG

>1WEPA

GSSGSSGMALVPVYCLCRQPYNVNHFMIECGLCQDWFHGSCVGIEEENAVDIDIYHCPDCEAVFGPSIMKNWHSGPSSG

>1WEUA

GSSGSSGSPEYGMPSVTFGSVHPSDVLDMPVDPNEPTYCLCHQVSYGEMIGCDNPDCSIEWFHFACVGLTTKPRGKWFCPRCSQESGPSSG

>1WEWA

GSSGSSGEDPFQPEIKVRCVCGNSLETDSMIQCEDPRCHVWQHVGCVILPDKPMDGNPPLPESFYCEICRLTSGPSSG

>1WG2A

GSSGSSGPSRPVRPNNRCFSCNKKVGVMGFKCKCGSTFCGSHRYPEKHECSFDFKEVGSGPSSG

>1WG6A

GSSGSSGLKGEPDCYALSLESSEQLTLEIPLNDSGSAGLGVSLKGNKSRETGTDLGIFIKSIIHGGAAFKDGRLRMNDQLIAVNGETLLGKSNHEAMETLRRSMSMEGNIRGMIQLVILRRSGPSSG

>1WH5A

GSSGSSGSSAEAGGGIRKRHRTKFTAEQKERMLALAERIGWRIQRQDDEVIQRFCQETGVPRQVLKVWLHNNKHSGPSSG

>1WI3A

GSSGSSGPRSRTKISLEALGILQSFIHDVGLYPDQEAIHTLSAQLDLPKHTIIKFFQNQRYHVKHSGPSSG

>1WIJA

GSSGSSGSQFVLQDLQDATLGSLLSSLMQHCDPPQRKYPLEKGTPPPWWPTGNEEWWVKLGLPKSQSPPYRKPHDLKKMWKVGVLTAVINHMLPDIAKIKRHVRQSKCLQDKMTAKESAIWLAVLNQEESLIQQSGPSSG

>1WJ2A

GSSGSSGVQTTSEVDLLDDGYRWRKYGQKVVKGNPYPRSYYKCTTPGCGVRKHVERAATDPKAVVTTYEGKHNHDLPA

>1WJVA

GSSGSSGMVFFTCNACGESVKKIQVEKHVSNCRNCECLSCIDCGKDFWGDDYKSHVKCISEGQKYGGKGYEAKSGPSSG

>1WPKA

MKKATCLTDDQRWQSVLARDPNADGEFVFAVRTTGIFCRPSCRARHALRENVSFYANASEALAAGFRPCKRCQPEKANAQQHRLDKITHACRLLEQETPVTLEALADQVAMSPFHLHRLFKATTGMTPKAWQQAWRARRLRESLAK

>1X3CA

GSSGSSGRKKPVSQSLEFPTRYSPYRPYRCVHQGCFAAFTIQQNLILHYQAVHKSDLPAFSAEVEEESGPSSG

>1X51A

GSSGSSGPRKASRKPPREESSATCVLEQPGALGAQILLVQRPNSGLLAGLWEFPSVTWEPSEQLQRKALLQELQRWAGPLPATHLRHLGEVVHTFSHIKLTYQVYGLALEGQTPVTTVPPGARWLTQEEFHTAAVSTAMKKVFRVYQGQSGPSSG

>1X57A

GSSGSSGDRVTLEVGKVIQQGRQSKGLTQKDLATKINEKPQVIADYESGRAIPNNQVLGKIERAIGLKLRGKDIGKPIEKGPRAKSGPSSG

>1X6FA

GSSGSSGLKRDFIILGNGPRLQNSTYQCKHCDSKLQSTAELTSHLNIHNEEFQKRAKRQERRKQLLSKQKYADGAFADFKQESGPSSG

>1XCBA

MKVPEAAISRLITYLRILEELEAQGVHRTSSEQLGELAQVTAFQVRKDLSYFGSYGTRGVGYTVPVLKRELRHILGLNRKWGLCIVGMGRLGSALADYPGFGESFELRGFFDVDPEKVGRPVRGGVIEHVDLLPQRVPGRIEIALLTVPREAAQKAADLLVAAGIKGILNFAPVVLEVPKEVAVENVDFLAGLTRLSFAILNPKWREEMMG

>1XD7A

MSLINSRLAVAIHILSLISMDEKTSSEIIADSVNTNPVVVRRMISLLKKADILTSRAGVPGASLKKDPADISLLEVYRAVQKQEELFAVHENPNPKCPVGKKIQNALDETFESVQRAMENELASKSLKDVMNHLFEGGSHHHHHH

>1XNAA

MPEIRLRHVVSCSSQDSTHCAENLLKADTYRKWRAAKAGEKTISVVLQLEKEEQIHSVDIGNDGSAFVEVLVGSSAGGAGEQDYEVLLVTSSFMSPSESRSGSNPNRVRMFGPDKLVRAAAEKRWDRVKIVCSQPYSKDSPFGLSFVRFHSPPDKDEAEAPSQKVTVTKLGQFRVKEEEESAN

>1XP8A

GSHMSKDATKEISAPTDAKERSKAIETAMSQIEKAFGKGSIMKLGAESKLDVQVVSTGSLSLDLALGVGGIPRGRITEIYGPESGGKTTLALAIVAQAQKAGGTCAFIDAEHALDPVYARALGVNTDELLVSQPDNGEQALEIMELLVRSGAIDVVVVDSVAALTPRAEIEGDMGDSLPGLQARLMSQALRKLTAILSKTGTAAIFINQVREKIGVMYGNPETTTGGRALKFYASVRLDVRKIGQPTKVGNDAVANTVKIKTVKNKVAAPFKEVELALVYGKGFDQLSDLVGLAADMDIIKKAGSFYSYGDERIGQGKEKTIAYIAERPEMEQEIRDRVMAAIRAGNAGEAPALAPAPAAPEAAEA

>1XV9B

PVQLSKEQEELIRTLLGAHTRHMGTMFEQFVQFRPPAHLFIHHQPLPTLAPVLPLVTHFADINTFMVLQVIKFTKDLPVFRSLPIEDQISLLKGAAVEICHIVLNTTFCLQTQNFLCGPLRYTIEDGARVGFQVEFLELLFHFHGTLRKLQLQEPEYVLLAAMALFSPDRPGVTQRDEIDQLQEEMALTLQSYIKGQQRRPRDRFLYAKLLGLLAELRSINEAYGYQIQHIQGLSAMMPLLQEICS

>1XWRD

MVRANKRNEALRIESALLNKIAMLGTEKTAEAVGVDKSQISRWKRDWIPKFSMLLAVLEWGVVDDDMARLARQVAAILTNKKRPAATERSEQIQMEF

>1Y6UA

AGHMKQTDIPIWERYTLTIEEASKYFRIGENKLRRLAEENKNANWLIMNGNRIQIKRKQFEKIIDTLDAI

>1YD6A

MNERLKEKLAVLPEQPGCYLMKDKHGTVIYVGKAKSLKERVRSYFTGTHDGKTQRLVEEIADFEYIVTSSNAEALILEMNLIKKHDPKYNVMLKDDKSY

>1YDXA

MGHHHHHHHHHHSSGHIDDDDKHMTPKLKLNNNINWTKRTIDSLFDLKKGEMLEKELITPEGKYEYFNGGVKNSGRTDKFNTFKNTISVIVGGSCGYVRLADKNFFCGQSNCTLNLLDPLELDLKFAYYALKSQQERIEALAFGTTIQNIRISDLKELEIPFTSNKNEQHAIANTLSVFDERLENLASLIEINRKLRDEYAHKLFSLDEAFLSHWKLEALQSQMHEITLGEIFNFKSGKYLKSEERLEEGKFPYYGAGIDNTGFVAEPNTEKDTISIISNGYSLGNIRYHEIPWFNGTGSIALEPMNNEIYVPFFYCALKYLQKDIKERMKSDDSPFLSLKLAGEIKVPYVKSFQLQRKAGKIVFLLDQKLDQYKKELSSLTVIRDTLLKKLFPDMTERTKSIKDY

>1YIOA

MTAKPTVFVVDDDMSVREGLRNLLRSAGFEVETFDCASTFLEHRRPEQHGCLVLDMRMPGMSGIELQEQLTAISDGIPIVFITAHGDIPMTVRAMKAGAIEFLPKPFEEQALLDAIEQGLQLNAERRQARETQDQLEQLFSSLTGREQQVLQLTIRGLMNKQIAGELGIAEVTVKVHRHNIMQKLNVRSLANLVHLVEKYESFERGVS

>1YJMC

MSQLGSRGRLWLQSPTGGPPPIFLPSDGQALVLGRGPLTQVTDRKCSRNQVELIADPESRTVAVKQLGVNPSTVGVHELKPGLSGSLSLGDVLYLVNGLYPLTLRWEELS

>1YSEA

GSHVSRSMNKPLEQQVSTNTEVSSEIYQWVRDELKRAGISQAVFARVAFNRTQGLLSEILRKEEDPKTASQSLLVNLRAMQNFLQLPEAERDRIYQDERERSLNAASAMGPAPLISTPPSRPPQVKTATIATERNGKPENN

>1YUAA

MNGEVAPPKEDPVPLPELPCEKSDAYFVLRDGAAGVFLAANTFPKSRETRAPLVEELYRFRDRLPEKLRYLADAPQQDPEGNKTMVRFSRKTKQQYVSSEKDGKATGWSAFYVDGKWVEGKK

>1YUIA

PKAKRAKHPPGTEKPRSRSQSEQPATCPICYAVIRQSRNLRRHLELRHFAKPGV

>1Z1BB

MGRRRSHERRDLPPNLYIRNNGYYCYRDPRTGKEFGLGRDRRIAITEAIQANIELFSGHKHKPLTARINSDNSVTLHSWLDRYEKILASRGIKQKTLINYMSKIKAIRRGLPDAPLEDITTKEIAAMLNGYIDEGKAASAKLIRSTLSDAFREAIAEGHITTNHVAATRAAKSKVRRSRLTADEYLKIYQAAESSPCWLRLAMELAVVTGQRVGDLCEMKWSDIVDGYLYVEQSKTGVKIAIPTALHIDALGISMKETLDKCKEILGGETIIASTRREPLSSGTVSRYFMRARKASGLSFEGDPPTFHELRSLSARLYEKQISDKFAQHLLGHKSDTMASQYRDDRGREWDKIEIK

>1Z4HA

MQHELQPDSLVDLKFIMADTGFGKTFIYDRIKSGDLPKAKVIHGRARWLYRDHCEFKNKLLSRANG

>1Z91A

MENKFDHMKLENQLSFLLYASSREMTKQYKPLLDKLNITYPQYLALLLLWEHETLTVKKMGEQLYLDSGTLTPMLKRMEQQGLITRKRSEEDERSVLISLTEDGALLKEKAVDIPGTILGLSKQSGEDLKQLKSALYTLLETLHQKN

>1ZAEB

HMDKTVNLSACEVAVLDLYEQSNIRIPSDIIEDLVNQRLQSEQEVLNYIETQRTYWKLENQKKLYRGSLK

>1ZI0B

TQEDVVVTLSHQGYVKYQPLSEYEAQRRGGKGKSAARIKEEDFIDRLLVANTHDHILCFSSRGRVYSMKVYQLPEATRGARGRPIVNLLPLEQDERITAILPVTEFEEGVKVFMATANGTVKKTVLTEFNRLRTAGKVAIKLVDGDELIGVDLTSGEDEVMLFSAEGKVVRFKESSVRAMGCNTTGVRGIRLGEGDKVVSLIVPRGDGAILTATQNGYGKRTAVAEYPTKSRATKGVISIKVTERNGLVVGAVQVDDCDQIMMITDAGTLVRTRVSEISIVGRNTQGVILIRTAEDENVVGLQRVAE

>1ZP7B

MIRYPNGKTFQPKHSVSSQNSQKRAPSYSNRGMTLEDDLNETNKYYLTNQIAVIHKKPTPVQIVNVHYPKRSAAVIKEAYFKQSSTTDYNGIYKGRYIDFEAKETKNKTSFPLQNFHDHQIEHMKQVKAQDGICFVIISAFDQVYFLEADKLFYFWDRKEKNGRKSIRKDELEETAYPISLGYAPRIDYISIIEQLYFSPSSGAKG

>1ZRJA

GSSGSSGMDVRRLKVNELREELQRRGLDTRGLKAELAERLQAALSGPSSG

>1ZS3A

MITKLMIDEKYAKELDKAEIDHHKPTAGAMLGHVLSNLFIENIRLTQAGIYAKSPVKCEYLREIAQREVEYFFKISDLLLDENEIVPSTTEEFLKYHKFITEDPKAKYWTDEDLLESFIVDFQAQNMFITRAIKLANKEEKFALAAGVVELYGYNLQVIRNLAGDLGKSVADFHDEDEDNDN

>1ZZKA

GAMGPIITTQVTIPKDLAGSIIGKGGQRIKQIRHESGASIKIDEPLEGSEDRIITITGTQDQIQNAQYLLQNSVKQYSGKFF

>2A1IA

MGSSHHHHHHSQDPAKSNSIIVSPRQRGNPVLKFVRNVPWEFGDVIPDYVLGQSTCALFLSLRYHNLHPDYIHGRLQSLGKNFALRVLLVQVDVKDPQQALKELAKMCILADCTLILAWSPEEAGRYLETYKAYEQKPADLLMEKL

>2A1JB

MGSSHHHHHHSQDPADLLMEKLEQDFVSRVTECLTTVKSVNKTDSQTLLTTFGSLEQLIAASREDLALCPGLGPQKARRLFDVLHEPFLKV

>2A1JA

MPQDFLLKMPGVNAKNCRSLMHHVKNIAELAALSQDELTSILGNAANAKQLYDFIHTSFAEVV

>2ADLB

MKQRITVTVDSDSYQLLKAYDVNISGLVSTTMQNEARRLRAERWKVENQEGMVEVARFIEMNGSFADENKDW

>2AIFA

GSSQNEASEDTGFNPKAFPLASPDLNNKIINLVQQACNYKQLRKGANEATKALNRGIAEIVLLAADAEPLEILLHLPLVCEDKNTPYVFVRSKVALGRACGVSRPVIAAAITSKDGSSLSSQITELKDQIEQILV

>2ALCA

GSMADTRRRQNHSCDPCRKGKRRCDAPENRNEANENGWVSCSNCKRWNKDCTFNWLSSQRSKNSS

>2AN7B

MSRLTIDMTDQQHQSLKALAALQGKTIKQYALERLFPGDADADQAWQELKTMLGNRINDGLAGKVSTKSVGEILDEELSGDRA

>2AQLB

MNRVEVKVKIPEELKPWLVDDWDLITRQKQLFYLPAKKNVDSILEDYANYKKSRGNTDNKEYAVNEVVAGIKEYFNVMLGTQLLYKFERPQYAEILADHPDAPMSQVYGAPHLLRLFVRIGAMLAYTPLDEKSLALLLNYLHDFLKYLAKNSATLFSASDYEVAPPEYHRKAV

>2ATQB

MKGFSSEDKGEWKLKLDASGNGQAVIRFLPAKTDDALPFAILVNHGFKKNGKWYIETCSSTHGDYDSCPVCQYISKNDLYNTNKTEYSQLKRKTSYWANILVVKDPQAPDNEGKVFKYRFGKKIWDKINAMIAVDTEMGETPVDVTCPWEGANFVLKVKQVSGFSNYDESKFLNQSAIPNIDDESFQKELFEQMVDLSEMTSKDKFKSFEELNTKFNQVLGTAALGGAAAAAAS

>2ATQA

MKEFYLTVEQIGDSIFERYIDSNGRERTREVEYKPSLFAHCPESQATKYFDIYGKPCTRKLFANMRDASQWIKRMEDIGLEALGMDDFKLAYLSDTYNYEIKYDHTKIRVANFDIEVTSPDGFPEPSQAKHPIDAITHYDSIDDRFYVFDLLNSPYGNVEEWSIEIAAKLQEQGGDEVPSEIIDKIIYMPFDNEKELLMEYLNFWQQKTPVILTGWNVESFAIPYVYNRIKNIFGESTAKRLSPHRKTRVKVIENMYGSREIITLFGISVLDYIDLYKKFSFTNQPSYSLDYISEFELNVGKLKYDGPISKLRESNHQRYISYNIIAVYRVLQIDAKRQFINLSLDMGYYAKIQIQSVFSPIKTWDAIIFNSLKEQNKVIPQGRSHPVQPYPGAFVKEPIPNRYKYVMSFDLTSLYPSIIRQVNISPETIAGTFKVAPLHDYINAVAERPSDVYSCSPNGMMYYKDRDGVVPTEITKVFNQRKEHKGYMLAAQRNGEIIKEALHNPNLSVDEPLDVDYRFDFSDEIKEKIKKLSAKSLNEMLFRAQRTEVAGMTAQINRKLLINSLYGALGNVWFRYYDLRNATAITTFGQMALQWIERKVNEYLNEVCGTEGEAFVLYGDTDSIYVSADKIIDKVGESKFRDTNHWVDFLDKFARERMEPAIDRGFREMCEYMNNKQHLMFMDREAIAGPPLGSKGIGGFWTGKKRYALNVWDMEGTRYAEPKLKIMGLETQKSSTPKAVQKALKECIRRMLQEGEESLQEYFKEFEKEFRQLNYISIASVSSANNIAKYDVGGFPGPKCPFHIRGILTYNRAIKGNIDAPQVVEGEKVYVLPLREGNPFGDKCIAWPSGTEITDLIKDDVLHWMDYTVLLEKTFIKPLEGFTSAAKLDYEKKASLFDMFDF

>2AXLA

MDDSEDTSWDFGPQAFKLLSAVDILGEKFGIGLPILFLRGSNSQRLADQYRRHSLFGTGKDQTESWWKAFSRQLITEGFLVEVSRYNKFMKICALTKKGRNWLHKANTESQSLILQANEELCPKKLLLPSSKTVSSGTKEHCYN

>2AY0A

MGTTTMGVMLDDATRERIKSAATRIDRTPHWLIKQAIFSYLEQLENSDTLPEHHHHHH

>2AY0B

MGTTTMGVMLDDATRERIKSAATRIDRTPHWLIKQAIFSYLEQLENSDTLPEHHHHHH

>2AY0C

MGTTTMGVMLDDATRERIKSAATRIDRTPHWLIKQAIFSYLEQLENSDTLPEHHHHHH

>2B0LC

GSSHHHHHHMSKAVVQMAISSLSYSELEAIEHIFEELDGNEGLLVASKIADRVGITRSVIVNALRKLESAGVIESRSLGMKGTYIKVLNNKFLIELENLKSH

>2BA3B

SDSAVRKKSEVRQKTVVRTLRFSPVEDETIRKKAEDSGLTVSAYIRNAALN

>2BDEA

MDTHKVFVNRIINMRKIKLIGLDMDHTLIRYNSKNFESLVYDLVKERLAESFHYPEEIKKFKFNFDDAIRGLVIDSKNGNILKLSRYGAIRLSYHGTKQISFSDQKKIYRSIYVDLGDPNYMAIDTSFSIAFCILYGQLVDLKDTNPDKMPSYQAIAQDVQYCVDKVHSDGTLKNIIIKNLKKYVIREKEVVEGLKHFIRYGKKIFILTNSEYSYSKLLLDYALSPFLDKGEHWQGLFEFVITLANKPRFFYDNLRFLSVNPENGTMTNVHGPIVPGVYQGGNAKKFTEDLGVGGDEILYIGDHIYGDILRLKKDCNWRTALVVEELGEEIASQIRALPIEKKIGEAMAIKKELEQKYVDLCTRSIDESSQQYDQEIHDLQLQISTVDLQISRLLQEQNSFYNPKWERVFRAGAEESYFAYQVDRFACIYMEKLSDLLEHSPMTYFRANRRLLAHDIDIAAALEHHHHHH

>2BNZA

MAKKDIMGDKTVRVRADLHHIIKIETAKNGGNVKEVMDQALEEYIRKYLPDKL

>2COBA

GSSGSSGRGRYRQYNSEILEEAISVVMSGKMSVSKAQSIYGIPHSTLEYKVKERLGTLKNPPKKKMKLMR

>2CXYA

GSSGSSGEKITKVYELGNEPERKLWVDRYLTFMEERGSPVSSLPAVGKKPLDLFRLYVCVKEIGGLAQVNKNKKWRELATNLNVGTSSSAASSLKKQYIQYLFAFECKIERGEEPPPEVFSTGDT

>2D7LA

GSSGSSGRPKTGFQMWLEENRSNILSDNPDFSDEADIIKEGMIRFRVLSTEERKVWANKAKGETASEGTEAKKRKSGPSSG

>2D8MA

GSSGSSGEPRRPRAGPEELGKILQGVVVVLSGFQNPFRSELRDKALELGAKYRPDWTRDSTHLICAFANTPKYSQVLGLGGRIVRKEWVLDCHRMRRRLPSQRYLMAGPGSSSEEDEASHSGGSGPSSG

>2D9HA

GSSGSSGLQCEICGFTCRQKASLNWHQRKHAETVAALRFPCEFCGKRFEKPDSVAAHRSKSHPALLLAPQESSGPSSG

>2DA6A

GSSGSSGRNRFKWGPASQQILYQAYDRQKNPSKEEREALVEECNRAECLQRGVSPSKAHGLGSNLVTEVRVYNWFANRRKEEAFRQKLAMDAYSSNSGPSSG

>2DGZA

GSSGSSGSSQPVISAQEQETQIVLYGKLVEARQKHANKMDVPPAILATNKILVDMAKMRPTTVENVKRIDGVSEGKAAMLAPLWEVIKHFCQTNSVQTDLFSSTKPQSGPSSG

>2DIGA

GSSGSSGMPSRKFADGEVVRGRWPGSSLYYEVEILSHDSTSQLYTVKYKDGTELELKENDIKSGPSSG

>2DINA

GSSGSSGKKTEWSREEEEKLLHLAKLMPTQWRTIAPIIGRTAAQCLEHYEFLLDKAAQRDSGPSSG

>2DMPA

GSSGSSGAYPDFAPQKFKEKTQGQVKILEDSFLKSSFPTQAELDRLRVETKLSRREIDSWFSERRKLRDSMEQAVLDSMGSGKSGPSSG

>2DMQA

GSSGSSGKRMRTSFKHHQLRTMKSYFAINHNPDAKDLKQLAQKTGLTKRVLQVWFQNARAKFRRNLLRQENGGVSGPSSG

>2DPDB

MKEEKRSSTGFLVKQRAFLKLYMITMTEQERLYGLKLLEVLRSEFKEIGFKPNHTEVYRSLHELLDDGILKQIKVKKEGAKLQEVVLYQFKDYEAAKLYKKQLKVELDRSKKLIEKALSDNF

>2DQBA

MRFSREALLELEASRLAPYAQKARDTRGRAHPEPESLYRTPYQKDRDRILHTTAFRRLEYKTQVLPGWAGDYYRTRLTHTLEVAQVSRSIARALGLNEDLTEAIALSHDLGHPPFGHTGEHVLNALMQDHGGFEHNAQALRILTHLEVRYPGFRGLNLTYEVLEGIATHEAAYSPGFKPLYEGQGTLEAQVVDLSDAIAYAAHDLDDGFRAGLLHPEELKEVELLQALALEEGLDLLRLPELDRRVLVRQLLGYFITAAIEATHRRVEEAGVQSAEAVRRHPSRLAALGEEAEKALKALKAFLMERFYRHPEVLRERRKAEAVLEGLFAAYTRYPELLPREVQAKIPEEGLERAVCDYIAGMTDRFALEAYRRLSP

>2E5RA

GSSGSSGVFHPVECSYCHSESMMGFRYRCQQCHNYQLCQDCFWRGHAGGSHSNQHQMKEYTSW

>2E6RA

GSSGSSGHSSAQFIDSYICQVCSRGDEDDKLLFCDGCDDNYHIFCLLPPLPEIPRGIWRCPKCILAECKQPPEAFGFEQATQEYSLSGPSSG

>2EBIA

KKRAETWVQDETRSLIMFRRGMDGLFNTSKSNKHLWEQISSKMREKGFDRSPDMCTDKWRNLLKEFKKAKHHDRGNGSAKMSYYKE

>2ELHA

GSSGSSGMNIRMGTKGKRPLRSLTPRDKIHAIQRIHDGESKASVARDIGVPESTLRGWCKNEDKLRFMSRQSATDNLCADALGDKMD

>2EO0B

MYIVNSNKSRGSSVERYIVSRLRDKGFAVIRAPASGSKRKDHVPDIIALKSGVIILIEVKSRKNGQKIYIEKEQAEGIREFAKRSGGELFLGVKLPKMLRFIKFDMLRQTEGGNYAIDLETVEKGMELEDLVRYVESKISRTLDSFL

>2EWTA

MSSEYAKQLGAKLRAIRTQQGLSLHGVEEKSQGRWKAVVVGSYERGDRAVTVQRLAELADFYGVPVQELLP

>2F2EB

MVKRTSHKQASCPVARPLDVIGDGWSMLIVRDAFEGLTRFGEFQKSLGLAKNILAARLRNLVEHGVMVAVPAESGSHQEYRLTDKGRALFPLLVAIRQWGEDYFFAPDESHVRLVERDSGQPVPRLQVRAGDGSPLAAEDTRVSRD

>2F7NA

MTKKSTKSEAASKTKKSGVPETGAQGVRAGGADHADAAHLGTVNNALVNHHYLEEKEFQTVAETLQRNLATTISLYLKFKKYHWDIRGRFFRDLHLAYDEFIAEIFPSIDEQAERLVALGGSPLAAPADLARYSTVQVPQETVRDARTQVADLVQDLSRVGKGYRDDSQACDEANDPVTADMYNGYAATIDKIRWMLQAIMDDERLD

>2FC7A

GSSGSSGQQMQAESGFVQHVGFKCDNCGIEPIQGVRWHCQDCPPEMSLDFCDSCSDCLHETDIHKEDHQLEPIYRSSGPSSG

>2FE3B

MAAHELKEALETLKETGVRITPQRHAILEYLVNSMAHPTADDIYKALEGKFPNMSVATVYNNLRVFRESGLVKELTYGDASSRFDFVTSDHYHAICENCGKIVDFHYPGLDEVEQLAAHVTGFKVSHHRLEIYGVCQECSKKENH

>2FMYA

ATQMRLTDTNLLEVLNSEEYSGVLKEFREQRYSKKAILYTPNTERNLVFLVKSGRVRVYLAYEDKEFTLAILEAGDIFCTHTRAFIQAMEDTTILYTDIRNFQNIVVEFPAFSLNMVKVLGDLLKNSLTIINGLVFKDARLRLAEFLVQAAMDTGLKVPQGIKLELGLNTEEIALMLGTTRQTVSVLLNDFKKMGILERVNQRTLLLKDLQKLKEFSSGV

>2FPHX

GIYQHFSIEDRPFLDKGMEWIKKVEDSYAPFLTPFINPHQEKLLKILAKTYGLACSSSGEFVSSEYVRVLLYPDYFQPEFSDFEISLQEIVYSNKFEYLTHAKILGTVINQLGIERKLFGDILVDEERAQIMINQQFLLLFQDGLKKIGRIPVSLEERPFTEKID

>2FU4B

MTDNNTALKKAGLKVTLPRLKILEVLQEPDNHHVSAEDLYKRLIDMGEEIGLATVYRVLNQFDDAGIVTRHNFEGGKSVFELT

>2FWRA

MGSSHHHHHHSSGLVPRGSHMQMIAEIYYERGTIVVKGDAHVPHAKFDSRSGTYRALAFRYRDIIEYFESNGIEFVDNAADPIPTPYFDAEISLRDYQEKALERWLVDKRGCIVLPTGSGKTHVAMAAINELSTPTLIVVPTLALAEQWKERLGIFGEEYVGEFSGRIKELKPLTVSTYDSAYVNAEKLGNRFMLLIFDEVHHLPAESYVQIAQMSIAPFRLGLTATFEREDGRHEILKEVVGGKVFELFPDSLAGKHLAKYTIKRIFVPLAEDERVEYEKREKVYKQFLRARGITLRRAEDFNKIVMASGYDERAYEALRAWEEARRIAFNSKNKIRKLREILERHRKDKIIIFTRHNELVYRISKVFLIPAITHRTSREEREEILEGFRTGRFRAIVSSQVLDEGIDVPDANVGVIMSGSGSAREYIQRLGRILRPSKGKKEAVLYELISRGTGEVNTARRRKNAAKGAA

>2G9WB

MAKLTRLGDLERAVMDHLWSRTEPQTVRQVHEALSARRDLAYTTVMAVLQRLAKKNLVLQIRDDRAHRYAPVHGRDELVAGLMVDALAQAEDSGSRQAALVHFVERVGADEADALRRALAELEAGHGNRPPAGAATET

>2GFUA

KAKNLNGGLRRSVAPAAPTSSDFSPGDLVWAKMEGYPWWPSLVYNHPFDGTFIREKGKSVRVHVQFFDDSPTRGWVSKRLLKPYTGSKSKEAQKGGHFYSAKPEILRAMQRADEALNKDKIKRLELAVSDEPSE

>2GMGA

AHHHHHHGSATRREKIIELLLEGDYSPSELARILDMRGKGSKKVILEDLKVISKIAKREGMVLLIKPAQCRKCGFVFKAEINIPSRCPKCKSEWIEEPRFKLERK

>2H6BB

MSVEGLGKDFCGAIIPDNFFPIEKLRNYTQMGLIRDFAKGSAVIMPGEEITSMIFLVEGKIKLDIIFEDGSEKLLYYAGGNSLIGKLYPTGNNIYATAMEPTRTCWFSEKSLRTVFRTDEDMIFEIFKNYLTKVAYYARQVAEMNTYNPTIRILRLFYELCSSQGKRVGDTYEITMPLSQKSIGEITGVHHVTVSRVLACLKRENILDKKKNKIIVYNLGELKHLSEQTSYYSDPNSSSVDKLAAALDHH

>2H9UA

MACEGAPEVRIGRKPVMNYVLAILTTLMEQGTNQVVVKARGRNINRAVDAVEIVRKRFAKNIEIKDIKIDSQEIEVQTPEGQTRTRRVSSIEICLEKAGESA

>2HGVA

GSSHHHHHHMALLQKTRIINSMLQAAAGKPVNFKEMAETLRDVIDSNIFVVSRRGKLLGYSINQQIENDRMKKMLEDRQFPEEYTKNLFNVPETSSNLDINSEYTAFPVENRDLFQAGLTTIVPIIGGGERLGTLILSRLQDQFNDDDLILAEYGATVVGMEIL

>2HKVA

GMTDWQQALDRHVGVGVRTTRDLIRLIQPEDWDKRPISGKRSVYEVAVHLAVLLEADLRIATGATADEMAQFYAVPVLPEQLVDRLDQSWQYYQDRLMADFSTETTYWGVTDSTTGWLLEAAVHLYHHRSQLLDYLNLLGYDIKLDLFE

>2HQLF

GGGGGGMLNRVFLEGEIESSCWSVKKTGFLVTIKQMRFFGERLFTDYYVIYANGQLAYELEKHTKKYKTISIEGILRTYLERKSEIWKTTIEIVKIFNPKNEIVIDYKEI

>2HUEA

PLGSPNSSIVSLLGIKVLNNPAKFTDPYEFEITFECLESLKHDLEWKLTYVGSSRSLDHDQELDSILVGPVPVGVNKFVFSADPPSAELIPASELVSVTVILLSCSYDGREFVRVGYYVNNEYDEEELRENPPAKVQVDHIVRNILAEKPRVTRFNIVWDNENEGDLYPPEQPGV

>2I13B

ISEFGSSSSVAQAALEPGEKPYACPECGKSFSRSDHLAEHQRTHTGEKPYKCPECGKSFSDKKDLTRHQRTHTGEKPYKCPECGKSFSQRANLRAHQRTHTGEKPYACPECGKSFSQLAHLRAHQRTHTGEKPYKCPECGKSFSREDNLHTHQRTHTGEKPYKCPECGKSFSRRDALNVHQRTHTGKKTS

>2IJGX

MNDHIHRVPALTEEEIDSVAIKTFERYALPSSSSVKRKGKGVTILWFRNDLRVLDNDALYKAWSSSDTILPVYCLDPRLFHTTHFFNFPKTGALRGGFLMECLVDLRKNLMKRGLNLLIRSGKPEEILPSLAKDFGARTVFAHKETCSEEVDVERLVNQGLKRVGNSTKLELIWGSTMYHKDDLPFDVFDLPDVYTQFRKSVEAKCSIRSSTRIPLSLGPTPSVDDWGDVPTLEKLGVEPQEVTRGMRFVGGESAGVGRVFEYFWKKDLLKVYKETRNGMLGPDYSTKFSPWLAFGCISPRFIYEEVQRYEKERVANNSTYWVLFELIWRDYFRFLSIKCGNSLFHLGGPRNVQGKWSQDQKLFESWRDAKTGYPLIDANMKELSTTGFMSNRGRQIVCSFLVRDMGLDWRMGAEWFETCLLDYDPCSNYGNWTYGAGVGNDPREDRYFSIPKQAQNYDPEGEYVAFWLQQLRRLPKEKRHWPGRLMYMDTVVPLKHGNGPMAGGSKSGGGFRGSHSGRRSRHNGP

>2INGX

GPRMSMVVSGLTPEEFMLVYKFARKHHITLTNLITEETTHVVMKTDAEFVCERTLKYFLGIAGGKWVVSYFWVTQSIKERKMLNEHDFEVRGDVVNGRNHQGPKRARESQDRKIFRGLEICCYGPFTNKPTDQLEWMVQLCGASVVKELSSFTLGTGVHPIVVVQPDAWTEDNGFHAIGQMCEAPVVTREWVLDSVALYQCQELDTYLIPQIP

>2IO4B

MMKAKVIDAVSFSYILRTVGDFLSEANFIVTKEGIRVSGIDPSRVVFLDIFLPSSYFEGFEVSQEKEIIGFKLEDVNDILKRVLKDDTLILSSNESKLTLTFDGEFTRSFELPLIQVESTQPPSVNLEFPFKAQLLTITFADIIDELSDLGEVLNIHSKENKLYFEVIGDLSTAKVELSTDNGTLLEASGADVSSSYGMEYVANTTKMRRASDSMELYFGSQIPLKLRFKLPQEGYGDFYIAPRAD

>2IO4A

MVKIVYPNAKDFFSFINSITNVTDSIILNFTEDGIFSRHLTEDKVLMAIMRIPKDVLSEYSIDSPTSVKLDVSSVKKILSKASSKKATIELTETDSGLKIIIRDEKSGAKSTIYIKAEKGQVEQLTEPKVNLAVNFTTDESVLNVIAADVTLVGEEMRISTEEDKIKIEAGEEGKRYVAFLMKDKPLKELSIDTSASSSYSAEMFKDAVKGLRGFSAPTMVSFGENLPMKIDVEAVSGGHMIFWIAPRL

>2JD3B

MDDERKRKKYTLYLHPEKAADFQTLEAIESVPRSERGELFRNAFISGMALHQLDPRLPVLLTAILSEEFSADQVVTLLSQTTGWKPSQADIRAVLTELGASQSVEKMPPSATDSVQEAMNDVRLKMKKLF

>2JMPA

MGGGGGGMEQFNAFKSLLKKHYEKTIGFHDKYIKDINRFVFKNNVLLILLENEFARNSLNDNSEIIHLAESLYEGIKSVNFVNEQDFFFNLAKLEENSRDTLYQNSG

>2JPCA

LRERQVLKLIDEGYTNHGISEKLHISIKTVETHRMNMMRKLQVHKVTELLNCARRMRLIEY

>2JR1A

FNVKQKSEITALVKEVTPPRKAPSKAKREAPIKYWLPHSGATWSGRGKIPKPFEAWIGTAAYTAWKAKHPDEKFPAFPG

>2JTMA

MSSGKKPVKVKTPAGKEAELVPEKVWALAPKGRKGVKIGLFKDPETGKYFRHKLPDDYPI

>2JULA

MQRTKEAVKASDGNLLGDPGRIPLSKRESIKWQRPRFTRQALMRCCLIKWILSSAAPQGSDSSDSELELSTVRHQPEGLDQLQAQTKFTKKELQSLYRGFKNECPTGLVDEDTFKLIYSQFFPQGDATTYAHFLFNAFDADGNGAIHFEDFVVGLSILLRGTVHEKLKWAFNLYDINKDGCITKEEMLAIMKSIYDMMGRHTYPILREDAPLEHVERFFQKMDRNQDGVVTIDEFLETCQKDENIMNSMQLFENVI

>2JX3A

FTIAQGKGQKLCEIERIHFFLSKKKTDELRNLHKLLYNRPGTVSSLKKNVGQFSGFPFEKGSVQYKKKEEMLKKFRNAMLKSICEVLDLERSGVNSELVKRILNFLMHPKPSGKPLPKSKKTCSKGSKKER

>2K4BA

MSYYHHHHHHDYDIPTTENLYFQGAMNEVEFNVSNAELIVMRVIWSLGEARVDEIYAQIPQELEWSLATVKTLLGRLVKKEMLSTEKEGRKFVYRPLME

>2K5VA

MNYKISELMPNLSGTINAEVVAAYPKKEFSRKDGTKGQLKSLFLKDDTGSIRGTLWNELADFEVKKGDIAEVSGYVKQGYSGLEISVDNIGIIEKSLEHHHHHH

>2K6GA

KRTNYQAYRSYLNREGPKALGSKEIPKGAENCLEGLIFVITGVLESIERDEAKSLIERYGGKVTGNVSKKTNYLVMGRDSGQSKSDKAAALGTKIIDEDGLLNLIRNLE

>2K75A

SDLVKIRDVSLSTPYVSVIGKITGIHKKEYESDGTTKSVYQGYIEDDTARIRISSFGKQLQDSDVVRIDNARVAQFNGYLSLSVGDSSRIESVNVNIPLEHHHHHH

>2K86A

GSSSRRNAWGNLSYADLITRAIESSPDKRLTLSQIYEWMVRCVPYFKDKGDSNSSAGWKNSIRHNLSLHSRFMRVQNEGTGKSSWWIINPDGGKSGKAPRRRA

>2K9IB

GRPYKLLNGIKLGVYIPQEWHDRLMEIAKEKNLTLSDVCRLAIKEYLDNHDKQKK

>2K9NA

KVKFTEEEDLKLQQLVMRYGAKDWIRISQLMITRNPRQCRERWNNYINPALRTDPWSPEEDMLLDQKYAEYGPKWNKISKFLKNRSDNNIRNRWMMIARHRAKHQKS

>2KEBA

MGSSHHHHHHGSSLEVLFQGPGSMSASAQQLAEELQIFGLDCEEALIEKLVELCVQYGQNEEGMVGELIAFCTSTHKVGLTSEILNSFEHEFLSKRLSKAR

>2KHQA

MITFADYFYQWYEVNKLPHVSESTKRHYESAYKHIKDHFRHKLLKDIKRTEYQKFLNEYGLTHSYETIRKLNSYIRNAFDDAIHEGYVIKNPTYKAELHASVLEHHHHHH

>2KI2A

MRNIYVGNLVYSATSEQVKELFSQFGKVFNVKLIYDRETKKPKGFGFVEMQEESVSEAIAKLDNTDFMGRTIRVTEANPKKSLEHHHHHH

>2KIWA

TFKQVADDWLKQYANDVKVSSVRAREKAIQHAIERFNTKPIQTIKKHDYQRFVDDISAQYSKNYVDSIVASTNMIFKYAYDTRLIKAMPSEGIKRPKKKVSVELEHHHHHH

>2KJ8A

SSNNNSFSAIYKEWYEHKKQVWSVGYATELAKMFDDDILPIIGGLEIQDIEPMQLLEVIRRFEDRGAMERANKARRRCGEVFRYAIVTGRAKYNPAPDLADAMKGYRKKNLEHHHHHH

>2KKOB

MAGQSDRKAALLDQVARVGKALANGRRLQILDLLAQGERAVEAIATATGMNLTTASANLQALKSGGLVEARREGTRQYYRIAGEDVARLFALVQVVADEHLEHHHHHH

>2KKPA

MIEPSKITVEQWLNRWLTDYAKPHLRQSTWESYETVLRLHVIPTLGSIPLKKLQPADIQRLYASKLESGLSPTRVRYIHVVLHEAMSQARESGLLLQNPTEAAKPPRHPLEHHHHHH

>2KKVA

MENSGAYTFETIAREWHESNKRWSEDHRSRVLRYLELYIFPHIGSSDIRQLKTSHLLAPIKEVDTSGKHDVAQRLQQRVTAIMRYAVQNDYIDSNPASDMAGALSTTKARHYPLEHHHHHH

>2KNGA

SGSGRGRGAIDREQSAAIREWARRNGHNVSTRGRIPADVIDAYHAATLEHHHHHH

>2KW3C

GHGTGSFGDRPARPTLLEQVLNQKRLSLLRSPEVVQFLQKQQQLLNQQVLEQRQQQFPGTSM

>2KW3B

GAGEPTTLLQRLRGTISKAVQNKVEGILQDVQKFSDNDKLYLYLQLPSGPTTGDKSSEPSTLSNEEYM

>2KWQA

GPMGMQSIREQSCRVVTCKTCKYTHFKPKETCVSENHDFHWHNGVKRFFKCPCGNRTISLDRLPKKHCSTCGLFKWERVGMLKEKTGPKLGG

>2L1PA

MGHHHHHHSHMLPPEQWSHTTVRNALKDLLKDMNQSSLAKECPLSQSMISSIVNSTYYANVSAAKCQEFGRWYKHFKKTKDMM

>2L3NA

SVSILRSSVNHREVDEAIDNILRYTNSTEQQFLEAMESTGGRVRIAIAKLLSKQTSGGSGGSKLGGSGGSRKDLSVKGMLYDSDSQQILNRLRERVSGSTAQSA

>2L49B

MSNTISEKIVLMRKSEYLSRQQLADLTGVPYGTLSYYESGRSTPPTDVMMNILQTPQFTKYTLWFMTNQIAPESGQIAPALAHFGQNETTSPHSGQKTG

>2L92A

MSTVPKYRDPATGKTWSGRGRQPAWLGNDPAAFLIQPDLPAILEHHHHHH

>2L93A

AARPAKYSYVDENGETKTWTGQGRTPAVIKKAMEEQGKQLEDFLIKELEHHHHHH

>2LFHB

MGHHHHHHSHMGGGKGPAAEEPLSLLDDMNHCYSRLRELVPGVPRGTQLSQVEILQRVIDYILDLQVV

>2LJ6A

MPSSKPLAEYARKRDFRQTPEPSGRKPRKDSTGLLRYCVQKHDASRLHYDFRLELDGTLKSWAVPKGPCLDPAVKRLAVQVEDHPLDYADFEGSIPQGHYGAGDVIVWDRGAWTPLDDPREGLEKGHLSFALDGEKLSGRWHLIRTNLRGKQSQWFLVKAKDGEARSLDRFDVLKER

>2LLHA

GSHMQESFKKQEKTPKTPKGPSSVEDIKAKMQASIEKGGSLPKVEAKFINYVKNCFRMTDQEAIQDLWQWRKSL

>2LSSA

MATNIVGKVKWYNSTKNFGFIEQDNGGKDVFVHKSAVDAAGLHSLEEGQDVIFDLEEKQGKAYAVNLRIK

>2LTTB

MADKLKFEIIEELIVLSENAKGWRKELNRVSWNDAEPKYDIRTWSPDHEKMGKGITLSEEEFGVLLKELGNKLEHHHHHH

>2LUAA

SPPKPKCRCGISGSSNTLTTCRNSRCPCYKSYNSCAGCHCVGCKNPHKEDYV

>2LUYA

HMGKNDNDALIMCMRCRKVKGIDSYSKTQWSKTFTFVRGRTVSVSDPKVICRTCQPKQHDSIWCTACQQTKGINEFSKAQRHVLDPRCQICVHSQRN

>2LW1A

GSHMKAETVKRSSSKLSYKLQRELEQLPQLLEDLEAKLEALQTQVADASFFSQPHEQTQKVLADMAAAEQELEQAFERWEYLEALKNGG

>2LYJB

MIINNLKLIREKKKISQSELAALLEVSRQTINGIEKNKYNPSLQLALKIAYYLNTPLEDIFQWQPE

>2M14A

PSHSGAAIFEKVSGIIAINEDVSPAELTWRSTDGDKVHTVVLSTIDKLQATPASSEKMMLRLIGKVDESKKRKDNEGNEVVPKPQRHMFSFNNRTVMDNIKMTLQQIISRYKDADGNSS

>2M8EA

ASMGKSKEISQDLRKKIVDLHKSGSSLGAISKRLKVPRSSVQTIVRKYKHHGTTQHH

>2MA1A

HDAPLFEALRAWRLQKAKELSLPPYTIFHDATLKTIAELRPGSHATLGTVSGVGGRKLAAYGDEVLQVVRDSSGG

>2MAMA

ADEPAYLTVGTDVSAKYRGAFCEAKIKTVKRLVKVKVLLKQDNTTQLVQDDQVKGPLRVGAIVETRTSDGSFQEAIISKLTDASWYTVVFDDGDERTLRRTSLCLKGERHFAESETLD

>2MH3B

MKPKTASEHRKSSKPIMEKRRRARINESLSQLKTLILDALKKDSSRHSKLEKADILEMTVKHLRNLQRAQ

>2NMUA

MAGDPNSMTVSHHNASTARFYALRLLPGQEVFSQLHAFVQQNQLRAAWIAGCTGSLTDVALRYAGQEATTSLTGTFEVISLNGTLELTGEHLHLAVSDPYGVMLGGHMMPGCTVRTTLELVIGELPALTFSRQPCAISGYDELHISSRLEHHHHHH

>2NOGB

MVSEPKVPKAPRPPKQPNVQDFQFFPPRLFELLEKEILYYRKTIGYKVPRNPDLPNSAQVQKEEQLKIDEAEPLNDEELEEKEKLLTQGFTNWNKRDFNQFIKANEKWGRDDIENIAREVEGKTPEEVIEYSAVFWERCNELQDIEKTMAQIERGEARIQRRISILEHHHHHH

>2NP2B

MSFSRRPKVTKSDIVDQIALNIKNNNLKLEKKYIRLVIDAFFEELKSNLCSNNVIEFRSFGTFEVRKRKGRLNARNPQTGEYVKVLDHHVAYFRPGKDLKERVWGIKG

>2O3CC

GSHMEAPILYEDPPEKLTSKDGRAANMKITSWNVDGLRAWVKKNGLDWVRKEDPDILCLQETKCAEKALPADITAMPEYPHKYWAGSEDKEGYSGVAMLCKTEPLNVTYGIGKEEHDKEGRVITAEFPDFFLVTAYVPNASRGLVRLDYRKTWDVDFRAYLCGLDARKPLVLCGDLNVAHQEIDLKNPKGNRKNAGFTPEEREGFTQLLEAGFTDSFRELYPDQAYAYTFWTYMMNARSKNVGWRLDYFVLSSALLPGLCDSKIRNTAMGSDHCPITLFLAV

>2O8BB

MGSAPQNSESQAHVSGGGDDSSRPTVWYHETLEWLKEEKRRDEHRRRPDHPDFDASTLYVPEDFLNSCTPGMRKWWQIKSQNFDLVICYKVGKFYELYHMDALIGVSELGLVFMKGNWAHSGFPEIAFGRYSDSLVQKGYKVARVEQTETPEMMEARCRKMAHISKYDRVVRREICRIITKGTQTYSVLEGDPSENYSKYLLSLKEKEEDSSGHTRAYGVCFVDTSLGKFFIGQFSDDRHCSRFRTLVAHYPPVQVLFEKGNLSKETKTILKSSLSCSLQEGLIPGSQFWDASKTLRTLLEEEYFREKLSDGIGVMLPQVLKGMTSESDSIGLTPGEKSELALSALGGCVFYLKKCLIDQELLSMANFEEYIPLDSDTVSTTRSGAIFTKAYQRMVLDAVTLNNLEIFLNGTNGSTEGTLLERVDTCHTPFGKRLLKQWLCAPLCNHYAINDRLDAIEDLMVVPDKISEVVELLKKLPDLERLLSKIHNVGSPLKSQNHPDSRAIMYEETTYSKKKIIDFLSALEGFKVMCKIIGIMEEVADGFKSKILKQVISLQTKNPEGRFPDLTVELNRWDTAFDHEKARKTGLITPKAGFDSDYDQALADIRENEQSLLEYLEKQRNRIGCRTIVYWGIGRNRYQLEIPENFTTRNLPEEYELKSTKKGCKRYWTKTIEKKLANLINAEERRDVSLKDCMRRLFYNFDKNYKDWQSAVECIAVLDVLLCLANYSRGGDGPMCRPVILLPEDTPPFLELKGSRHPCITKTFFGDDFIPNDILIGCEEEEQENGKAYCVLVTGPNMGGKSTLMRQAGLLAVMAQMGCYVPAEVCRLTPIDRVFTRLGASDRIMSGESTFFVELSETASILMHATAHSLVLVDELGRGTATFDGTAIANAVVKELAETIKCRTLFSTHYHSLVEDYSQNVAVRLGHMACMVENECEDPSQETITFLYKFIKGACPKSYGFNAARLANLPEEVIQKGHRKAREFEKMNQSLRLFREVCLASERSTVDAEAVHKLLTLIKEL

>2O8BA

MAVQPKETLQLESAAEVGFVRFFQGMPEKPTTTVRLFDRGDFYTAHGEDALLAAREVFKTQGVIKYMGPAGAKNLQSVVLSKMNFESFVKDLLLVRQYRVEVYKNRAGNKASKENDWYLAYKASPGNLSQFEDILFGNNDMSASIGVVGVKMSAVDGQRQVGVGYVDSIQRKLGLCEFPDNDQFSNLEALLIQIGPKECVLPGGETAGDMGKLRQIIQRGGILITERKKADFSTKDIYQDLNRLLKGKKGEQMNSAVLPEMENQVAVSSLSAVIKFLELLSDDSNFGQFELTTFDFSQYMKLDIAAVRALNLFQGSVEDTTGSQSLAALLNKCKTPQGQRLVNQWIKQPLMDKNRIEERLNLVEAFVEDAELRQTLQEDLLRRFPDLNRLAKKFQRQAANLQDCYRLYQGINQLPNVIQALEKHEGKHQKLLLAVFVTPLTDLRSDFSKFQEMIETTLDMDQVENHEFLVKPSFDPNLSELREIMNDLEKKMQSTLISAARDLGLDPGKQIKLDSSAQFGYYFRVTCKEEKVLRNNKNFSTVDIQKNGVKFTNSKLTSLNEEYTKNKTEYEEAQDAIVKEIVNISSGYVEPMQTLNDVLAQLDAVVSFAHVSNGAPVPYVRPAILEKGQGRIILKASRHACVEVQDEIAFIPNDVYFEKDKQMFHIITGPNMGGKSTYIRQTGVIVLMAQIGCFVPCESAEVSIVDCILARVGAGDSQLKGVSTFMAEMLETASILRSATKDSLIIIDELGRGTSTYDGFGLAWAISEYIATKIGAFCMFATHFHELTALANQIPTVNNLHVTALTTEETLTMLYQVKKGVCDQSFGIHVAELANFPKHVIECAKQKALELEEFQYIGESQGYDIMEPAAKKCYLEREQGEKIIQEFLSKVKQMPFTEMSEENITIKLKQLKAEVIAKNNSFVNEIISRIKVTT

>2O99A

GHMSRNLLAIVHPILRNLMEESGETVNMAVLDQSDHEAIIIDQVQCTHLMRMSAPIGGKLPMHASGAGKAFLAQLSEEQVTKLLHRKGLHAYTHATLVSPVHLKEDLAQTRKRGYSFDDEEHALGLRCLAACIFDEHREPFAAISISGPISRITDDRVTEFGAMVIKAAKEVTLAYGGMRGS

>2OBPB

GMSDPGNEQNGDGIDPAIVEVLLVLREAGIENGATPWSLPKIAKRAQLPMSVLRRVLTQLQAAGLADVSVEADGRGHASLTQEGAALAAQLFPDPF

>2OCJD

SSSVPSQKTYQGSYGFRLGFLHSGTAKSVTCTYSPALNKMFCQLAKTCPVQLWVDSTPPPGTRVRAMAIYKQSQHMTEVVRRCPHHERCSDSDGLAPPQHLIRVEGNLRVEYLDDRNTFRHSVVVPYEPPEVGSDCTTIHYNYMCNSSCMGGMNRRPILTIITLEDSSGNLLGRNSFEVRVCACPGRDRRTEEENLRKKGEPHHELPPGSTKRALPNNT

>2OD5A

GMTGAVETESMKTVRIREKIKKFLGDRPRNTAEILEHINSTMRHGTTSQQLGNVLSKDKDIVKVGYIKRSGILSGGYDICEWATRNWVAEHCPEWTEGQPIILNEEGDFTLGPLPE

>2OKFA

GMSARDVFHEVVKTALKKDGWQITDDPLTISVGGVNLSIDLAAQKLIAAERQGQKIAVEVKSFLKQSSAISEFHTALGQFINYRGALRKVEPDRVLYLAVPLTTYKTFFQLDFPKEIIIENQVKMLVYDVEQEVIFQWIN

>2OWYB

MWFRNLLVYRLTQDLQLDADSLEKALGEKSARPCASQELTTYGFTAPFGKGPDAPLVHVSQDFFLISARKEERILPGSVVRDALKEKVDEIEAQQMRKVYKKERDQLKDEIVQTLLPRAFIRRSSTFAAIAPSLGLILVDSASAKKAEDLLSTLREALGSLPVRPLSVKVAPTATLTDWVKTQEAAGDFHVLDECELRDTHEDGGVVRCKRQDLTSEEIQLHLTAGKLVTQLSLAWSDKLSFVLDDKLAVKRLRFEDLLQEQAEKDGGEDALGQLDASFTLMMLTFAEFLPALFEALGGEEIPQGV

>2OZ9R

AQQSPYSAAMAEQRHQEWLRFVDLLKNAYQNDLHLPLLNLMLTPDEREALGTRVRIVEELLRGEMSQRELKNELGAGIATITRGSNSLKAAPVELRQWLEEVLLKSD

>2OZEA

MIQYYYTKKEWGVVMEKEELKILEELRRILSNKNEAIVILNNYFKGGVGKSKLSTMFAYLTDKLNLKVLMIDKDLQATLTKDLAKTFKVELPRVNFYEGLKNGNLASSIVHLTDNLDLIPGTFDLMLLPKLTRSWTFENESRLLATLLAPLKSDYDLIIIDTVPTPSVYTNNAIVASDYVMIPLQAEEESTNNIQNYISYLIDLQEQFNPGLDMIGFVPYLVDTDSATIKSNLEELYKQHKEDNLVFQNIIKRSNKVSTWSKNGITEHKGYDKKVLSMYKNVFFEMLERIIQLENEKE

>2P2UB

SLSRRKPNPVIVADIRQAEGALAEIATIDRKVGEIEAQMNEAIDAAKARASQKSAPLLARRKELEDGVATFATLNKTEMFKDRKSLDLGFGTIGFRLSTQIVQMSKITKDMTLERLRQFGISEGIRIKEDVNKEAMQGWPDERLEMVGLKRRTTDAFYIEINREEVADTAA

>2P5KA

MNKGQRHIKIREIITSNEIETQDELVDMLKQDGYKVTQATVSRDIKELHLVKVPTNNGSYKYSL

>2P5MC

MQRFNPLSKLKRALMDAFVKIDSASHMIVLKTMPGNAQAIGALMDNLDWDEMMGTICGDDTILIICRTPEDTEGVKNRLLELL

>2P6RA

MKVEELAESISSYAVGILKEEGIEELFPPQAEAVEKVFSGKNLLLAMPTAAGKTLLAEMAMVREAIKGGKSLYVVPLRALAGEKYESFKKWEKIGLRIGISTGDYESRDEHLGDCDIIVTTSEKADSLIRNRASWIKAVSCLVVDEIHLLDSEKRGATLEILVTKMRRMNKALRVIGLSATAPNVTEIAEWLDADYYVSDWRPVPLVEGVLCEGTLELFDGAFSTSRRVKFEELVEECVAENGGVLVFESTRRGAEKTAVKLSAITAKYVENEGLEKAILEENEGEMSRKLAECVRKGAAFHHAGLLNGQRRVVEDAFRRGNIKVVVATPTLAAGVNLPARRVIVRSLYRFDGYSKRIKVSEYKQMAGRAGRPGMDERGEAIIIVGKRDREIAVKRYIFGEPERITSKLGVETHLRFHSLSIICDGYAKTLEELEDFFADTFFFKQNEISLSYELERVVRQLENWGMVVEAAHLAPTKLGSLVSRLYIDPLTGFIFHDVLSRMELSDIGALHLICRTPDMERLTVRKTDSWVEEEAFRLRKELSYYPSDFSVEYDWFLSEVKTALCLKDWIEEKDEDEICAKYGIAPGDLRRIVETAEWLSNAMNRIAEEVGNTSVSGLTERIKHGVKEELLELVRIRHIGRVRARKLYNAGIRNAEDIVRHREKVASLIGRGIAERVVEGISVKSLNPESAAALEHHHHHH

>2PG4A

GMDDETLRLQFGHLIRILPTLLEFEKKGYEPSLAEIVKASGVSEKTFFMGLKDRLIRAGLVKEETLSYRVKTLKLTEKGRRLAECLEKCRDVLGS

>2PI2E

MGHHHHHHHHHHSSGHIEGRHMVDMMDLPRSRINAGMLAQFIDKPVCFVGRLEKIHPTGKMFILSDGEGKNGTIELMEPLDEEISGIVEVVGRVTAKATILCTSYVQFKEDSHPFDLGLYNEAVKIIHDFPQFYPLGIVQHD

>2PI2A

MWNSGFESYGSSSYGGAGGYTQSPGGFGSPAPSQAEKKSRARAQHIVPCTISQLLSATLVDEVFRIGNVEISQVTIVGIIRHAEKAPTNIVYKIDDMTAAPMDVRQWVDTDDTSSENTVVPPETYVKVAGHLRSFQNKKSLVAFKIMPLEDMNEFTTHILEVINAHMVLSKANSQPSAGRAPISNPGMSEAGNFGGNSFMPANGLTVAQNQVLNLIKACPRPEGLNFQDLKNQLKHMSVSSIKQAVDFLSNEGHIYSTVDDDHFKSTDAE

>2PKHH

SNAARGHRHTCKVMVLKEEAAGSERALALDMREGQRVFHSLIVHFENDIPVQIEDRFVNAQVAPDYLKQDFTLQTPYAYLSQVAPLTEGEHVVEAILAEADECKLLQIDAGEPCLLIRRRTWSGRQPVTAARLIHPGSRHRLEGRFTK

>2PNHB

GSHMTNRLVLSGTVCRAPLRKVSPSGIPHCQFVLEHRSVQEAAGFHRQAWCQMPVIVSGHENQAITHSITVGSRITVQGFISCHKAKNGLSKMVLHAEQIELIDSGD

>2Q2KB

MGSSHHHHHHSSGLVPGSHMDKKETKHLLKIKKEDYPQIFDFLENVPRGTKTAHIREALRRYIEEIGENP

>2Q79A

TTPIVHLKGDANTLKCLRYRFKKHCTLYTAVSSTWHWTGHNVKHKSAIVTLTYDSEWQRDQFLSQVKIPKTITVSTGFMSIGGGTGGGSGGGS

>2QLCA

MNLKVKGARDVFEYMKGRIPDETKEHLFVLFLSTKNQILRHETITIGTLTASLIHPREIFKAAIRESAHSIILVHNHPSGDVQPSNADKQVTSILKKAGDLLQIELLDHVIVGNNDWFSFRDHALL

>2QPYA

CQPIFLNVLEAIEPGVVCAGHDNNQPDSFAALLSSLNELGERQLVHVVKWAKALPGFRNLHVDDQMAVIQYSWMGLMVFAMGWRSFTNVNSRMLYFAPDLVFNEYRMHKSRMYSQCVRMRHLSQEFGWLQITPQEFLCMKALLLFSIIPVDGLKNQKFFDELRMNYIKELDRIIACKRKNPTSCSRRFYQLTKLLDSVQPIARELHQFTFDLLIKSHMVSVDFPEMMAEIISVQVPKILSGKVKPIYFHTQ

>2QSFX

GSGNASSGALGTTGGATDAAQGGPPGSIGLTVEDLLSLRQVVSGNPEALAPLLENISARYPQLREHIMANPEVFVSMLLEAVGDNMQDVMEGADDMVEGEDIEVTGEAAAAGLGQGEGEGSFQVDYTPEDDQAISRLCELGFERDLVIQVYFACDKNEEAAANILFSDHAD

>2QSHA

GSSRAMGNEVAGVEDISVEIKPSSKRNSDARRTSRNVCSNEERKRRKYFHMLYLVCLMVHGFIRNEWINSKRLSRKLSNLVPEKVFELLHPQKDEELPLRSTRKLLDGLKKCMELWQKHWKITKKYDNEGLYMRTWKEIEMSANNKRKFKTLKRSDFLRAVSKGHGDPDISVQGFVAMLRACNVNARLIMSCQPPDFTNMKIDTSLNGNNAYKDMVKYPIFWCEVWDKFSKKWITVDPVNLKTIEQVRLHSKLAPKGVACCERNMLRYVIAYDRKYGCRDVTRRYAQWMNSKVRKRRITKDDFGEKWFRKVITALHHRKRTKIDDYEDQYFFQRDESEGIPDSVQDLKNHPYYVLEQDIKQTQIVKPGCKECGYLKVHGKVGKVLKVYAKRDIADLKSARQWYMNGRILKTGSRCKKVIKRTVGRPKGEAEEEDERLYSFEDTELYIPPLASASGEITKNTFGNIEVFAPTMIPGNCCLVENPVAIKAARFLGVEFAPAVTSFKFERGSTVKPVLSGIVVAKWLREAIETAIDGIEFI

>2QUQA

KLITASSSKEYLPDLLLFWQNYEYWITNIGLYKTKQRDLTRTPANLDTDTEECMFWMNYLQKDQSFQLMNFAMENLGALYFGSIGDISELYLRVEQYWDRRADKNHSVDGKYWDALIWSVFTMCIYYMPVEKLAEIFSVYPLHEYLGSNKRLNWEDGMQLVMCQNFARCSLFQLKQCDFMAHPDIRLVQAYLILATTTFPYDEPLLANSLLTQCIHTFKNFHVDDFRPLLNDDPVESIAKVTLGRIFYRLCGCDYLQSGPRKPIALHTEVSSLLQHAAYLQDLPNVDVYREENSTEVLYWKIISLDRDLDQYLNKSSKPPLKTLDAIRRELDIFQYKVDSLEEDFRSNNSRFQKFIALFQISTVSWKLFKMYLIYYDTADSLLKVIHYSKVIISLIVNNFHAKSEFFNRHPMVMQTITRVVSFISFYQIFVESAAVKQLLVDLTELTANLPTIFGSKLDKLVYLTERLSKLKLLWDKVQLLDSGDSFYHPVFKILQNDIKIIELKNDEMFSLIKGLGSLVPLNKLRQESLLEEEDENNTEPSDFRTIVEEFQSEYNISDILS

>2RH3A

IQVFLSARPPAPEVSKIYDNLILQYSPSKSLQMILRRALGDFENMLADGSFRAAPKSYPIPHTAFEKSIIVQTSRMFPVSLIEAARNHFDPLGLETARAFGHKLATAALACFFAREKATNS

>2ROHA

GSPFADPNSLALANVPLSRSKRPDFGQRRIRRPFTVAEVELLVEAVEHLGTGRWRDVKFRAFENVHHRTYVDLKDKWKTLVHTASIAPQQRRGAPVPQELLDRVLAAQAYWSVDSSGRIVTL

>2RRDA

GIPEFKQKALVAKVSQREEMVKKCLGELTEVCKSLGKVFGVHYFNIFNTVTLKKLAESLSSDPEVLLQIDGVTEDKLEKYGAEVISVLQKYSEWTSPAEDS

>2RT6A

MKTALLLEKLEGQLATLRQRCAPVSQFATLSARFDRHLFQTRATTLQACLDEAGDNLAALRHAVEQQQLPQVAWLAEHLAAQLEAIAREASAWSLREW

>2VL6C

MEIPSKQIDYRDVFIEFLTTFKGNNNQNKYIERINELVAYRKKSLIIEFSDVLSFNENLAYEIINNTKIILPILEGALYDHILQLDPTYQRDIEKVHVRIVGIPRVIELRKIRSTDIGKLITIDGILVKVTPVKERIYKATYKHIHPDCMQEFEWPEDEEMPEVLEMPTICPKCGKPGQFRLIPEKTKLIDWQKAVIQERPEEVPSGQLPRQLEIILEDDLVDSARPGDRVKVTGILDIKQDSPVKRGSRAVFDIYMKVSSIEVSQKV

>2WKCD

GTIITVTAQANEKNTRTVSTAKGDKKIISVPLFEKEKGSNVKVAYGSAFLPDFIQLGDTVTVSGRVQAKESGEYVNYNFVFPTVEKVFITNDNSSQSQAKQDLFGGSEPIEVNSEDLPF

>2WP0D

MDTNNNIEKEILALVKQNPKVSLIEYENYFSQLKYNPNASKSDIAFFYAPNQVLCTTITAKYGALLKEILSQNKVGMHLAHSVDVRIEVAPKIQINAQSNINYKAIKTSVKD

>2WP0B

MKNFYDWIKEFIRDQGEFIAQQSGWLELERSSYAKLIAQTISHVLNGGSLLVSADSSRHWFLNYILSNLNPKDLKERPLLSVIDFNASSFYPKNDANLSLATIEMTYQNPMFWHVGKIENEGLKTILLSKIPSFLWLFEELKEDCLLLKEHDSLLDYKLLQLFKLFENALFSVLYNKVTL

>2XE0B

NTKYNKEFLLYLAGFVDGDGSIIAQIKPRASNKFAHQLSLTFAVTQKTQRRWFLDKLVDEIGVGYVYDSGSVSDYRLSEIKPLHNFLTQLQPFLKLKQKQANLVLAIIEQLPSAKASPDAFLEVCTWVDQIAALNDSKTRATTSATVRAALD

>2XIWB

MRGSHHHHHHGSVKVKFLLNGEEKEVDTSKIRDVSRQGKNVKFLYNDNGKYGAGNVDEKDAPKELLDMLARAEREKKLN

>2XMAA

GSHMTYVILPLEMKKGRGYVYQLEYHLIWCVKYRHQVLVGEVADGLKDILRDIAAQNGLEVITMEVMPDHVHLLLSATPQQAIPDFVKALKGASARRMFVAYPQLKEKLWGGNLWNPSYCILTVSENTRAQIQKYIESQHDKE

>2YPFA

VDLRTLAYSQQQQEKIKPKVRSTVAQHHEALVAHAFTHAHIVALSQHPAALATVAVKYQDMIAALPEATHEAIVAVAKQWSGARALEALLTVAGELRGPPLQLDTGQLLKIAKRGGVTAVEAVHAWRNALTGAPLNLTPEQVVAIASHDGGKQALETVQRLLPVLCQAHGLTPQQVVAIASNGGGKQALETVQRLLPVLCQAHGLTPEQVVAIASNIGGKQALETVQALLPVLCQAHGLTPQQVVAIASNGGGKQALETVQRLLPVLCQAHGLTPEQVVAIASNIGGKQALETVQALLPVLCQAHGLTPEQVVAIASNIGGKQALETVQALLPVLCQAHGLTPEQVVAIASNIGGKQALETVQALLPVLCQAHGLTPEQVVAIASHDGGKQALETVQRLLPVLCQAHGLTPEQVVAIASHDGGKQALETVQRLLPVLCQAHGLTPQQVVAIASNGGGKQALETVQRLLPVLCQAHGLTPEQVVAIASNIGGKQALETVQALLPVLCQAHGLTPEQVVAIASNIGGKQALETVQALLPVLCQAHGLTPEQVVAIASHDGGKQALETVQRLLPVLCQAHGLTPEQVVAIASHDGGKQALETVQRLLPVLCQAHGLTPEQVVAIASHDGGKQALETVQRLLPVLCQAHGLTPQQVVAIASNGGGKQALETVQRLLPVLCQAAGLTPEQVVAIASHDGGKQALETVQRLLPVLCQAHGLTPQQVVAIASNGGGRPALESIVAQLSRPDPGSSAALEHHHHHH

>2YRQA

GSSGSSGMGKGDPKKPRGKMSSYAFFVQTCREEHKKKHPDASVNFSEFSKKCSERWKTMSAKEKGKFEDMAKADKARYEREMKTYIPPKGETKKKFKDPNAPKRPPSAFFLFCSEYRPKIKGEHPGLSIGDVAKKLGEMWNNTAADDKQPYEKKAAKLKEKYEKDIAAYRAKG

>2YVAB

MQERIKACFTESIQTQIAAAEALPDAISRAAMTLVQSLLNGNKILCCGNGTSAANAQHFAASMINRFETERPSLPAIALNTDNVVLTAIANDRLHDEVYAKQVRALGHAGDVLLAISTRGNSRDIVKAVEAAVTRDMTIVALTGYDGGELAGLLGPQDVEIRIPSHRSARIQEMHMLTVNCLCDLIDNTLFPHQDD

>2Z3XC

AKLLIPQAASAIEQMKLEIASEFGVQLGAETTSRANGSVGGEITKRLVRLAQQNMGGQFHGQQ

>2Z4RC

MKERILQEIKTRVNRKSWELWFSSFDVKSIEGNKVVFSVGNLFIKEWLEKKYYSVLSKAVKVVLGNDATFEITYEAFEPHSSYSEPLVKKRAVLLTPLNPDYTFENFVVGPGNSFAYHAALEVAKHPGRYNPLFIYGGVGLGKTHLLQSIGNYVVQNEPDLRVMYITSEKFLNDLVDSMKEGKLNEFREKYRKKVDILLIDDVQFLIGKTGVQTELFHTFNELHDSGKQIVICSDREPQKLSEFQDRLVSRFQMGLVAKLEPPDEETRKSIARKMLEIEHGELPEEVLNFVAENVDDNLRRLRGAIIKLLVYKETTGKEVDLKEAILLLKDFIKPNRVKAMDPIDELIEIVAKVTGVPREEILSNSRNVKALTARRIGMYVAKNYLKSSLRTIAEKFNRSHPVVVDSVKKVKDSLLKGNKQLKALIDEVIGEISRRALSG

>2ZDSF

MPRNFTLFTGQWADLPLEEVCRLARDFGYDGLELACWGDHFEVDKALADPSYVDSRHQLLDKYGLKCWAISNHLVGQAVCDAIIDERHEAILPARIWGDGDAEGVRQRAAAEIKDTARAAARLGVDTVIGFTGSAIWHLVAMFPPAPESMIERGYQDFADRWNPILDVFDAEGVRFAHEVHPSEIAYDYWTTHRALEAVGHRPAFGLNFDPSHFVWQDLDPVGFLWDFRDRIYHVDCKEARKRLDGRNGRLGSHLPWGDPRRGWDFVSAGHGDVPWEDVFRMLRSIDYQGPVSVEWEDAGMDRLQGAPEALTRLKAFDFEPPSASFDAAFNSLEHHHHHH

>2ZQEA

MREVKEVDLRGLTVAEALLEVDQALEEARALGLSTLRLLHGKGTGALRQAIREALRRDKRVESFADAPPGEGGHGVTVVALRP

>2ZTCD

MASMTGGQQMGRGSEFMIASVRGEVLEVALDHVVIEAAGVGYRVNATPATLATLRQGTEARLITAMIVREDSMTLYGFPDGETRDLFLTLLSVSGVGPRLAMAALAVHDAPALRQVLADGNVAALTRVPGIGKRGAERMVLELRDKVGVAATGGALSTNGHAVRSPVVEALVGLGFAAKQAEEATDTVLAANHDATTSSALRSALSLLGKAR

>3AL2A

GPLGSLKKQYIFQLSSLNPQERIDYCHLIEKLGGLVIEKQCFDPTCTHIVVGHPLRNEKYLASVAAGKWVLHRSYLEACRTAGHFVQEEDYEWGSSSILDVLTGINVQQRRLALAAMRWRKKIQQRQESGIVEGAFSGWKVILHVDQSREAGFKRLLQSGGAKVLPGHSVPLFKEATHLFSDLNKLKPDDSGVNIAEAAAQNVYCLRTEYIADYLMQESPPHVENYCLPEAISFI

>3AQQD

MSSEPPPPPQPPTHQASVGLLDTPRSRERSPSPLRGNVVPSPLPTRRTRTFSATVRASQGPVYKGVCKCFCRSKGHGFITPADGGPDIFLHISDVEGEYVPVEGDEVTYKMCSIPPKNEKLQAVEVVITHLAPGTKHETWSGHVISS

>3ASKA

SLYKVNEYVDARDTNMGAWFEAQVVRVTRKAPSRPALEEDVIYHVKYDDYPENGVVQMNSRDVRARARTIIKWQDLEVGQVVMLNYNPDNPKERGFWYDAEISRKRETRTARELYANVVLGDDSLNDCRIIFVDEVFKIERPGEGSPMVDNPMRRKSGPSCKHCKDDVNRLCRVCACHLCGGRQDPDKQLMCDECDMAFHIYCLDPPLSSVPSEDEWYCPECRNDA

>3AXJB

MPKNGGAGHRNTAPRKRQIPAAQLDEDSPIVQQFRIYSNELIMKHDRHERIVKLSRDITIESKRIIFLLHSIDSRKQNKEKVLEEARQRLNKLIAVNFRAVALELRDQDVYQFRSSYSPGLQEFIQAYTYMEYLCHEDAEGENETKSVSDWQAIQAVMQYVEESSQPKEEPTEGEDVQAIAQVESPKKFQFFVDPTEYILGLSDLTGELMRRCINSLGSGDTDTCLDTCKALQHFYSGYISLNCQRARELWRKITTMKQSVLKAENVCYNVKVRGGEAAKWGATFDQKPADEVDEGFY

>3B0CT

GSTREPEIASSLIKQIFSHYVKTPVTRDAYKIVEKCSERYFKQISSDLEAYSQHAGRKTVEMADVELLMRRQGLVTDKMPLHVLVERHLPLEYRKLLIPIAVSGNKVIPCK

>3BOSB

GMRSNRVTQHPPLQLSLPVHLPDDETFTSYYPAAGNDELIGALKSAASGDGVQAIYLWGPVKSGRTHLIHAACARANELERRSFYIPLGIHASISTALLEGLEQFDLICIDDVDAVAGHPLWEEAIFDLYNRVAEQKRGSLIVSASASPMEAGFVLPDLVSRMHWGLTYQLQPMMDDEKLAALQRRAAMRGLQLPEDVGRFLLNRMARDLRTLFDVLDRLDKASMVHQRKLTIPFVKEMLRL

>3BRFA

SGPLGSGDSVQSLTSDRMIDFLSNKEKYECVISIFHAKVAQKSYGNEKRFFCPPPCIYLIGQGWKLKKDRVAQLYKTLKASAQKDAAIENDPIHEQQATELVAYIGIGSDTSERQQLDFSTGKVRHPGDQRQDPNIYDYCAAKTLYISDSDKRKYFDLNAQFFYGCGMEIGGFVSQRIKVISKPSKKKQSMKNTDCKYLCIASGTKVALFNRLRSQTVSTRYLHVEGNAFHASSTKWGAFTIHLFDDERGLQETDNFAVRDGFVYYGSVVKLVDSVTGIALPRLRIRKVDKQQVILDASCSEEPVSQLHKCAFQMIDNELVYLCLSHDKIIQHQATAINEHRHQINDGAAWTIISTDKAEYRFFEAMGQVANPISPCPVVGSLEVDGHGEASRVELHGRDFKPNLKVWFGATPVETTFRSEESLHCSIPPVSQVRNEQTHWMFTNRTTGDVEVPISLVRDDGVVYSSGLTFSYKSLER

>3BS3A

SNAMSNNQQMMLNRIKVVLAEKQRTNRWLAEQMGKSENTISRWCSNKSQPSLDMLVKVAELLNVDPRQLINGKIKI

>3BTPB

MVIIKLNANKNMPVLAVEKPQEIHKEELSDHHQSNGFTSLDLEMIELENFVLHCPLPEENLAG

>3BTPA

MDPKAEGNGENITETAAGNVETSDFVNLKRQKREGVNSTGMSEIDMTGSQETPEHNMHGSPTHTDDLGPRLDADMLDSQSSHVSSSAQGNRSEVENELSNLFAKMALPGHDRRTDEYILVRQTGQDKFAGTTKCNLDHLPTKAEFNASCRLYRDGVGNYYPPPLAFERIDLPEQLAAQLHNLEPREQSKQCFQYKLEVWNRAHAEMGITGTDIFYQTDKNIKLDRNYKLRPEDRYIQTEKYGRREIQKRYEHQFQAGSLLPDILIKTPQNDIHFSYRFAGDAYANKRFEEFERAIKTKYGSDTEIKLKSKSGIMHDSKYLESWERGSADIRFAEFAGENRAHNKQFPAATVNMGRQPDGQGGMTRDRHVSVDYLLQNLPNSPWTQALKEGKLWDRVQVLARDGNRYMSPSRLEYSDPEHFTQLMDQVGLPVSMGRQSHANSVKFEQFDRQAAVIVADGPNLREVPDLSPEKLQQLSQKDVLIADRNEKGQRTGTYTNVVEYERLMMKLPSDAAQLLAEPSDRYSRAFVRPEPALPPISDSRRTYESRPRGPTVNSL

>3BU8B

GAGEARLEEAVNRWVLKFYFHEALRAFRGSRYGDFRQIRDIMQALLVRPLGKEHTVSRLLRVMQCLSRIEEGENLDCSFDMEAELTPLESAINVLEMIKTEFTLTEAVVESSRKLVKEAAVIICIKNKEFEKASKILKKHMSKDPTTQKLRNDLLNIIREKNLAHPVIQNFSYETFQQKMLRFLESHLDDAEPYLLTMAKKALKSESAASSTGKEDKQPAPGPVEKPPREPARQL

>3C1DB

GPAYARLLDRAVRILAVRDHSEQELRRKLAAPIMGKNGPEEIDATAEDYERVIAWCHEHGYLDDSRFVARFIASRSRKGYGPARIRQELNQKGISREATEKAMREADIDWAALARDQATRKYGEPLPTVFSEKVKIQRFLLYRGYLMEDIQDIWRNFAD

>3C1YB

MGSSHHHHHHSSGLVPRGSHMGVKSLVPQELIEKIKLISPGTELRKALDDIINANFGALIFLVDDPKKYEDVIQGGFWLDTDFSAEKLYELSKMDGAIVLSEDITKIYYANVHLVPDPTIPTGETGTRHRTAERLAKQTGKVVIAVSRRRNIISLYYKNYKYVVNQVDFLISKVTQAISTLEKYKDNFNKLLSELEVLELENRVTLADVVRTLAKGFELLRIVEEIRPYIVELGEEGRLARMQLRELTEDVDDLLVLLIMDYSSEEVEEETAQNILQDFITRREPSPISISRVLGYDVQQAAQLDDVLVSARGYRLLKTVARIPLSIGYNVVRMFKTLDQISKASVEDLKKVEGIGEKRARAISESISSLKHRKTSE

>3CNBC

MSLNVKNDFSILIIEDDKEFADMLTQFLENLFPYAKIKIAYNPFDAGDLLHTVKPDVVMLDLMMVGMDGFSICHRIKSTPATANIIVIAMTGALTDDNVSRIVALGAETCFGKPLNFTLLEKTIKQLVEQKKATSEGHHHHHH

>3D6WB

GKSVVTLKTTDGWIPVPFSKVMYLEAKDKKTYVNAEELTGTHKYSLQEFEYLLPKDSFIRCHRSFIVNVNHIKAIYPDTHSTFLLSMDNGERVPVSQSYASYFRKLLGFGS

>3DPJA

SNAMVQAQTRDQIVAAADELFYRQGFAQTSFVDISAAVGISRGNFYYHFKTKDEILAEVIRLRLARTAQMLADWQGTGDSPRARIASFIDLMIMNRAKITRYGCPVGSLCTELSKLDHAAQGQANGLFTLFRDWLQRQFAEAGCTTEAPALAMHLLARSQGAATLAQSFHDEGFLRSEVADMHRWLDNTLPMTT

>3EI4F

MHHHHHHRRLVPRGSGGRQKTSEIVLRPRNKRSRSPLELEPEAKKLCAKGSGPSRRCDSDCLWVGLAGPQILPPCRSIVRTLHQHKLGRASWPSVQQGLQQSFLHTLDSYRILQKAAPFDRRATSLAWHPTHPSTVAVGSKGGDIMLWNFGIKDKPTFIKGIGAGGSITGLKFNPLNTNQFYASSMEGTTRLQDFKGNILRVFASSDTINIWFCSLDVSASSRMVVTGDNVGNVILLNMDGKELWNLRMHKKKVTHVALNPCCDWFLATASVDQTVKIWDLRQVRGKASFLYSLPHRHPVNAACFSPDGARLLTTDQKSEIRVYSASQWDCPLGLIPHPHRHFQHLTPIKAAWHPRYNLIVVGRYPDPNFKSCTPYELRTIDVFDGNSGKMMCQLYDPESSGISSLNEFNPMGDTLASAMGYHILIWSQEEARTRK

>3EIVD

MAGETVITVVGNLVDDPELRFTPSGAAVAKFRVASTPRTFDRQTNEWKDGESLFLTCSVWRQAAENVAESLQRGMRVIVQGRLKQRSYEDREGVKRTVYELDVDEVGASLRSATAKVTKTSGQGRGGQGGYGGGGGGQGGGGWGGGPGGGQQGGGAPADDPWATGGAPAGGQQGGGGQGGGGWGGGSGGGGGYSDEPPF

>3EQXB

GMEWQAEQAYNHLPPLPLDSKLAELAETLPILKACIPARAALAELKQAGELLPNQGLLINLLPLLEAQGSSEIENIVTTTDKLFQYAQEDSQADPMTKEALRYRTALYQCFTQLSNRPLCVTTALEICSTIKSVQMDVRKVPGTSLTNQATGEVIYTPPAGESVIRDLLSNWEAFLHNQDDVDPLIKMAMAHYQFEAIHPFIDGNGRTGRVLNILYLIDQQLLSAPILYLSRYIVAHKQDYYRLLLNVTTQQEWQPWIIFILNAVEQTAKWTTHKIAAARELIAHTTEYVRQQLPKIYSHELVQVIFEQPYCRIQNLVESGLAKRQTASVYLKQLCDIGVLEEVQSGKEKLFVHPKFVTLMTKDSNQFSRYAL

>3EUSB

QAMTKTLRTPEHVYLCQRLRQARLDAGLTQADLAERLDKPQSFVAKVETRERRLDVIEFAKWMAACEGLDVVSEIVATIAEGRAQA

>3EYIB

HMASPQFSQQREEDIYRFLKDNGPQRALVIAQALGMRTAKDVNRDLYRMKSRHLLDMDEQSKAWTIYRWTIY

>3EZ2A

MSDSSQLHKVAQRANRMLNVLTEQVQLQKDELHANEFYQVYAKAALAKLPLLTRANVDYAVSEMEEKGYVFDKRPAGSSMKYAMSIQNIIDIYEHRGVPKYRDRYSEAYVIFISNLKGGVSKTVSTVSLAHAMRAHPHLLMEDLRILVIDLDPQSSATMFLSHKHSIGIVNATSAQAMLQNVSREELLEEFIVPSVVPGVDVMPASIDDAFIASDWRELCNEHLPGQNIHAVLKENVIDKLKSDYDFILVDSGPHLDAFLKNALASANILFTPLPPATVDFHSSLKYVARLPELVKLISDEGCECQLATNIGFMSKLSNKADHKYCHSLAKEVFGGDMLDVFLPRLDGFERCGESFDTVISANPATYVGSADALKNARIAAEDFAKAVFDRIEFIRSN

>3F1ZA

GASKAFYSAGDKLFQPGDDAVASMQTYSVAQFLQPFTLNPAKASSDYLGKWVKVRGVIVDIRRKSGIAGSYYFIVTMRDEQNKTDKRLTFNFGSHNSADVEALSNGSVATIVGQVHQVQDSTIPTLQNPKVVK

>3F6CB

SLNAIIIDDHPLAIAAIRNLLIKNDIEILAELTEGGSAVQRVETLKPDIVIIDVDIPGVNGIQVLETLRKRQYSGIIIIVSAKNDHFYGKHCADAGANGFVSKKEGMNNIIAAIEAAKNGYCYFPFSLNRFVGS

>3FDQB

MPKSEIRKLLQEIKKQVDNPGNSSTTEIKKMASEAGIDEQTAEEIYHLLTEFYQAVEEHGGIEKYMHSNISWLKIELELLSACYQIAILEDMKVLDISEMLSLNDLRIFPKTPSQLQNTYYKLKKELIQVEDIPKNKPGRKRKTQKNTKKEKTNIFGKVVPALEHHHHHH

>3FDQA

MPKSEIRKLLQEIKKQVDNPGNSSTTEIKKMASEAGIDEQTAEEIYHLLTEFYQAVEEHGGIEKYMHSNISWLKIELELLSACYQIAILEDMKVLDISEMLSLNDLRIFPKTPSQLQNTYYKLKKELIQVEDIPKNKPGRKRKTQKNTKKEKTNIFGKVVPALEHHHHHH

>3FHWB

MNTLELSARVLECGAMRHTPAGLPALELLLVHESEVVEAGHPRRVELTISAVALGDLALLLADTPLGTEMQVQGFLAPARKDSVKVKLHLQQARRIAGSMGRDPLVGLEHHHHHH

>3FRQA

GMPRPKLKSDDEVLEAATVVLKRCGPIEFTLSGVAKEVGLSRAALIQRFTNRDTLLVRMMERGVEQVRHYLNAIPIGAGPQGLWEFLQVLVRSMNTRNDFSVNYLISWYELQVPELRTLAIQRNRAVVEGIRKRLPPGAPAAAELLLHSVIAGATMQWAVDPDGELADHVLAQIAAILCLMFPEHDDFQLLQAHA

>3FYMA

MKTVGEALKGRRERLGMTLTELEQRTGIKREMLVHIENNEFDQLPNKNYSEGFIRKYASVVNIEPNQLIQAHQDEIPSNQAEWDEVITVFNNNKDLDYKSKSKEPIQLLVIMGITVLITLLLWIMLVLIF

>3G1CA

MNNKLKTQAVEQLFQAILSLKDLDEAYDFFEDVCTINEILSLSQRFEVAKMLREHRTYLDIAEKTGASTATISRVNRSLNYGNDGYDRVFERLGMLEKESEDNK

>3GIOB

MGSSHHHHHHSSGLVPRGSHMPNTSQRNSFLQDVPYWMLQNRSEYITQGVDSSHIVDGKKTEEIEKIATKRATIRVAQNIVHKLKEAYLSKTNRIKQKITNEMFIQMTQPIYDSLMNVDRLGIYINPNNEEVFALVRARGFDKDALSEGLHKMSLDNQAVSILVAKVEEIFKDSVNYGDVKVPIAM

>3GN5B

GHMKCPVCHQGEMVSGIKDIPYTFRGRKTVLKGIHGLYCVHCEESIMNKEESDAFMAQVKAFRASVNAETVAPEFIVKVRKKLSLTQKEASEIFGGGVNAFSRYEKGNAQPHPSTIKLLRVLDKHPELLNEIR

>3GVAB

MRMDEFYTKVYDAVCEIPYGKVSTYGEIARYVGMPSYARQVGQAMKHLHPETHVPWHRVINSRGTISKRDISAGEQRQKDRLEEEGVEIYQTSLGEYKLNLPEYMWKPGSHHHHHH

>3GXQB

ENSVFFGKKKKVSLHLLVDPDMKDEIIKYAQEKDFDNVSQAGREILKKGLEQIA

>3GYDB

MGSDKIHHHHHHENLYFQGMYPDLVHLGGADKYFEEILEIVNKIKLFGDFSNEEVRYLCSYMQCYAAPRDCQLLTEGDPGDYLLLILTGEVNVIKDIPNKGIQTIAKVGAGAIIGEMSMIDGMPRSASCVASLPTDFAVLSRDALYQLLANMPKLGNKVLIRLLQLLTARFRESYDRILPKTLGELI

>3GZ5B

GSHMTEAEYLANYDPKAFKAQLLTVDAVLFTYHDQQLKVLLVQRSNHPFLGLWGLPGGFIDETCDESLEQTVLRKLAEKTAVVPPYIEQLCTVGNNSRDARGWSVTVCYTALMSYQACQIQIASVSDVKWWPLADVLQMPLAFDHLQLIEQARERLTQKALYSLVPGFALSEPFTLPELQHVHEVLLGKPIQGKSFRRRVEQADLLIDTGLKRTERGRPANLYCLKPDTASYRFLRNLEC

>3H4LB

GSMTQIHQINDIDVHRITSGQVITDLTTAVKELVDNSIDANANQIEIIFKDYGLESIECSDNGDGIDPSNYEFLALKHYTSKIAKFQDVAKVQTLGFRGEALSSLCGIAKLSVITTTSPPKADKLEYDMVGHITSKTTTSRNKGTTVLVSQLFHNLPVRQKEFSKTFKRQFTKCLTVIQGYAIINAAIKFSVWNITPKGKKNLILSTMRNSSMRKNISSVFGAGGMRGLEEVDLVLDLNPFKNRMLGKYTDDPDFLDLDYKIRVKGYISQNSFGCGRNSKDRQFIYVNKRPVEYSTLLKCCNEVYKTFNNVQFPAVFLNLELPMSLIDVNVTPDKRVILLHNERAVIDIFKTTLSDYYNRQELALPK

>3HI2D

GSHMEKRTPHTRLSQVKKLVNAGQVRTTRSALLNADELGLDFDGMCNVIIGLSESDFYKSMTTYSDHTIWQDVYRPRLVTGQVYLKITVIHDVLIVSFKEK

>3HOSA

MSSFVPNKEQTRTVLIFCFHLKKTAAESHRMLVEAFGEQVPTVKTCERWFQRFKSGDFDVDDKEHGKPPKRYEDAELQALLDEDDAQTQKQLAEQLEVSQQAVSNRLREMGKIQKVGRWVPHELNERQMERRKNTCEILLSRYKRKSFLHRIVTGDEKWIFFVNPKRKKSYVDPGQPATSTARPNRFGKKTMLCVWWDQSGVIYYELLKPGETVNAARYQQQLINLNRALQRKRPEYQKRQHRVIFLHDNAPSHTARAVRDTLETLNWEVLPHAAYSPDLAPSDYHLFASMGHALAEQRFDSYESVKKWLDEWFAAKDDEFYWRGIHKLPERWEKCVASDGKYFE

>3HTAD

MGSSHHHHHHSSGLVPRGSHMPRRHDPERRQRIIDAAIRVVGQKGIAGLSHRTVAAEADVPLGSTTYHFATLDDLMVAALRQANEGFARVVAAHPALSDPEADLSGELARVLGEWLGGDRTGVELEYELYLAALRRPALRPVAAEWAEGVGALLAARTDPTTARALVAVLDGICLQVLLTDTPYDEEYAREVLTRLIPVPATRDGRGPGSHPPATAG

>3I54D

MGSSHHHHHHSSGLGGTENLYFQSHMDEILARAGIFQGVEPSAIAALTKQLQPVDFPRGHTVFAEGEPGDRLYIIISGKVKIGRRAPDGRENLLTIMGPSDMFGELSIFDPGPRTSSATTITEVRAVSMDRDALRSWIADRPEISEQLLRVLARRLRRTNNNLADLIFTDVPGRVAKQLLQLAQRFGTQEGGALRVTHDLTQEEIAQLVGASRETVNKALADFAHRGWIRLEGKSVLISDSERLARRAR

>3II2A

GSEMSVVEYEVVSKNLTSKMSHELLFSVKKRWFVKPFRHDRQLGKLHYKLLPGNYIKFGLYVLKNQDYARFEIAWVHVDKDGKIEERTVYSIETYWHIFIDIENDLNCPYVLAKFIEMRPEFHKTAWVEESNYSIAEDDIQMVESIKRYLERKIASD

>3II6X

GAMGSKISNIFEDVEFCVMSGTDSQPKPDLENRIAEFGGYIVQNPGPDTYCVIAGSENIRVKNIILSNKHDVVKPAWLLECFKTKSFVPWQPRFMIHMCPSTKEHFAREYDCYGDSYFIDTDLNQLKEVFSGIKNSNEQTPEEMASLIADLEYRYSWDCSPLSMFRRHTVYLDSYAVINDLSTKNEGTRLAIKALELRFHGAKVVSCLAEGVSHVIIGEDHSRVADFKAFRRTFKRKFKILKESWVTDSIDKCELQEENQYLI

>3II6A

MERKISRIHLVSEPSITHFLQVSWEKTLESGFVITLTDGHSAWTGTVSESEISQEADDMEMEKGKYVGELRKALLSGAGPADVYTFNFSKESCYFFFEKNLKDVSFRLGSFNLEKVENPAEVIRELICYCLDTTAENQAKNEHLQKENERLLRDWNDVQGRFEKCVSAKEALETDLYKRFILVLNEKKTKIRSLHNKLLNAAQ

>3IO5B

GSHMDVVRTKIPMMNIALSGEITGGMQSGLLILAGPSKSFKSNFGLTMVSSYMRQYPDAVCLFYDSEFGITPAYLRSMGVDPERVIHTPVQSLEQLRIDMVNQLDAIERGEKVVVFIDSLGNLASKKETEDALNEKVVSDMTRAKTMKSLFRIVTPYFSTKNIPCIAINHTYETQEMFSKTVMGGGTGPMYSADTVFIIGKRQIKDGSDLQGYQFVLNVEKSRTVKEKSKFFIDVKFDGGIDPYSGLLDMALELGFVVKPKNGWYAREFLDEETGEMIREEKSWRAKDTNCTTFWGPLFKHQPFRDAIKRAYQLGAIDSNEIVEAEVDELINS

>3IV5B

MFEQRVNSDVLTVSTVNSQDQVTQKPLRDSVKQALKNYFAQLNGQDVNDLYELVLAEVEQPLLDMVMQYTRGNQTRAALMMGINRGTLRKKLKKYGMN

>3IVPD

MRKKEDKYDFRALGLAIKEARKKQGLTREQVGAMIEIDPRYLTNIENKGQHPSLQVLYDLVSLLNVSVDEFFLPASSQVKSTKRRQLENKIDNFTDADLVIMESVADGIVKSKEVGEMAGENLYFQ

>3JU0A

MSLTDSKVKNAKSLEKEYKLTDGFGMHLLVHPNGSKYWRLSYRFEKKQRLLALGVYPAVSLADARQRRDEAKKLLAAGIDPSAKKQADNKTIQEKRNNTRLEHHHHHH

>3K2AB

GSGIFPKVATNIMRAWLFQHLTHPYPSEEQKKQLAQDTGLTILQVNNWFINARRRIVQPMIDQSNRA

>3K4XA

GPHMASMLEAKFEEASLFKRIIDGFKDCVQLVNFQCKEDGIIAQAVDDSRVLLVSLEIGVEAFQEYRCDHPVTLGMDLTSLSKILRCGNNTDTLTLIADNTPDSIILLFEDTKKDRIAEYSLKLMDIDADFLKIEELQYDSTLSLPSSEFSKIVRDLSQLSDSINIMITKETIKFVADGDIGSGSVIIKPFVDMEHPETSIKLEMDQPVDLTFGAKYLLDIIKGSSLSDRVGIRLSSEAPALFQFDLKSGFLQFFLAPKFNDEGSNSQSNGSGALEAKFEEASLFKRIIDGFKDCVQLVNFQCKEDGIIAQAVDDSRVLLVSLEIGVEAFQEYRCDHPVTLGMDLTSLSKILRCGNNTDTLTLIADNTPDSIILLFEDTKKDRIAEYSLKLMDIDADFLKIEELQYDSTLSLPSSEFSKIVRDLSQLSDSINIMITKETIKFVADGDIGSGSVIIKPFVDMEHPETSIKLEMDQPVDLTFGAKYLLDIIKGSSLSDRVGIRLSSEAPALFQFDLKSGFLQFFLAPKFNDEGSNSQASNSGALEAKFEEASLFKRIIDGFKDCVQLVNFQCKEDGIIAQAVDDSRVLLVSLEIGVEAFQEYRCDHPVTLGMDLTSLSKILRCGNNTDTLTLIADNTPDSIILLFEDTKKDRIAEYSLKLMDIDADFLKIEELQYDSTLSLPSSEFSKIVRDLSQLSDSINIMITKETIKFVADGDIGSGSVIIKPFVDMEHPETSIKLEMDQPVDLTFGAKYLLDIIKGSSLSDRVGIRLSSEAPALFQFDLKSGFLQFFLAPKFNDEE

>3KDEC

MKYCKFCCKAVTGVKLIHVPKCAIKRKLWEQSLGCSLGENSQICDTHFNDSQWKAAPAKGQTFKRRRLNADAVPSKV

>3KHKB

MSLDIEQQFLNDLDNQLWRAADKLRSNLDAANYKHVVLGLIFLKYVSDAFEERQQELTELFQKDDDDNIYYLPREDYDSDEAYQQAIAEELEIGDYYTEKNVFWVPKTARWNKLRDVITLPTGSVIWQDEQGEDVKLRSVSWLIDNAFDDIEKANPKLKGILNRISQYQLDADKLIGLINEFSLTSFNNPEYNGEKLNLKSKDILGHVYEYFLGQFALAEGKQGGQYYTPKSIVTLIVEMLEPYKGRVYDPAMGSGGFFVSSDKFIEKHANVKHYNASEQKKQISVYGQESNPTTWKLAAMNMVIRGIDFNFGKKNADSFLDDQHPDLRADFVMTNPPFNMKDWWHEKLADDPRWTINTNGEKRILTPPTGNANFAWMLHMLYHLAPTGSMALLLANGSMSSNTNNEGEIRKTLVEQDLVECMVALPGQLFTNTQIPACIWFLTKDKNAKNGKRDRRGQVLFIDARKLGYMKDRVLRDFKDEDIQKLADTFHNWQQEWSEENNQAGFCFSADLALIRKNDFVLTPGRYVGAEAEEDEGHHHHHH

>3KJOA

MSLVPATNYIYTPLNQLKGGTIVNVYGVVKFFKPPYLSKGTDYCSVVTIVDQTNVKLTCLLFSGNYEALPIIYKNGDIVRFHRLKIQVYKKETQGITSSGFASLTFEGTLGAPIIPRTSSKYFNFTTEDHKMVEALRVWASTHMSPSWTLLKLCDVQPMQYFDLTCQLLGKAEVDGASFLLKVWDGTRTPFPSWRVLIQDLVLEGDLSHIHRLQNLTIDILVYDNHVHVARSLKVGSFLRIYSLHTKLQSMNSENQTMLSLEFHLHGGTSYGRGIRVLPESNSDVDQLKKDLESANLTA

>3KOJB

MGHHHHHHSHMNSCILQATVVEAPQLRYAQDNQTPVAEMVVQFPGLSSKDAPARLKVVGWGAVAQELQDRCRLNDEVVLEGRLRINSLLKPDGNREKQTELTVTRVHH

>3LDAA

MSQVQEQHISESQLQYGNGSLMSTVPADLSQSVVDGNGNGSSEDIEATNGSGDGGGLQEQAEAQGEMEDEAYDEAALGSFVPIEKLQVNGITMADVKKLRESGLHTAEAVAYAPRKDLLEIKGISEAKADKLLNEAARLVPMGFVTAADFHMRRSELICLTTGSKNLDTLLGGGVETGSITELFGEFRTGKSQLCHTLAVTCQIPLDIGGGEGKCLYIDTEGTFRPVRLVSIAQRFGLDPDDALNNVAYARAYNADHQLRLLDAAAQMMSESRFSLIVVDSVMALYRTDFSGRGELSARQMHLAKFMRALQRLADQFGVAVVVTNQVVAQVDGGMAFNPDPKKPIGGNIMAYSSTTRLGFKKGKGCQRLCKVVDSPCLPEAECVFAIYEDGVGDPREEDE

>3LHKD

SNAKIIGYARVSFNAQKDDLERQIQLIKSYAEENGWDIQILKDIGSGLNEKRKNYKKLLKMVMNRKVEKVIIAYPDRLTRFGFETLKEFFKSYGTEIVIINKKHKTPQEELVEDLITIVSHFAGKLYGMHSHKYKKLTKTVKEIVREEDAKEKE

>3M03C

MSNIGIRDLAVQFSCIEAVNMASKILKSYESSLPQTQQVDLDLSRPLFTSAALLSACKILKLKVDKNKMVATSGVKKAIFDRLCKQLEKIGQQVD

>3M8EB

MGSSHHHHHHSSGLVPRGSHMNRDHFYTLNIAEIAERIGNDDCAYQVLMAFINENGEAQMLNKTAVAEMIQLSKPTVFATVNSFYCAGYIDETRVGRSKIYTLSDLGVEIVECFKQKAMEMRNL

>3MAJA

GHMDVGERSSDQGTTVLTEAQRIDWMRLIRAENVGPRTFRSLINHFGSARAALERLPELARRGGAARAGRIPSEDEARREIEAGRRIGVELVAPGETGYPTRLATIDDAPPLLGVHALPEALAVMARPMIAIVGSRNASGAGLKFAGQLAADLGAAGFVVISGLARGIDQAAHRASLSSGTVAVLAGGHDKIYPAEHEDLLLDIIQTRGAAISEMPLGHVPRGKDFPRRNRLISGASVGVAVIEAAYRSGSLITARRAADQGREVFAVPGSPLDPRAAGTNDLIKQGATLITSASDIVEAVASILERPIELPGREPEHAPPEGEPDTGDRTRILALLGPSPVGIDDLIRLSGISPAVVRTILLELELAGRLERHGGSLVSLS

>3MKYB

MGSSHHHHHHSSGLVPRGSHYRPTSAYERGQRYASRLQNEFAGNISALADAENISRKIITRCINTAKLPKSVVALFSHPGELSARSGDALQKAFTDKEELLKQQASNLHEQKKAGVIFEADEVITLLTSVLKTSSASRTSLSSRHQFAPGATVLYKGDKMVLNLDRSRVPTECIEKIEAILKELEKPAP

>3MU6D

GRKKIQITRIMDERNRQVTFTKRKFGLMKKAYELSVLCDCEIALIIFNSSNKLFQYASTDMDKVLLKYTAY

>3MUJB

SMEATPCIKAISPSEGWTTGGATVIIIGDNFFDGLQVVFGTMLVWSELITPHAIRVQTPPRHIPGVVEVTLSYKSKQFCKGAPGRFVYTALNEPTIDYGFQRLQKVIPRHPGDPERLPKEVLLKRAADLVEALYGMPH

>3N4PA

MGHHHHHHDYDIPTTENLYFQGGGTNKISQNTVLITDQSREEFDILRYSTLNTNAYDYFGKTLYVYLDPAFTTNRKASGTGVAAVGAYRHQFLIYGLEHFFLRDLSESSEVAIAECAAHMIISVLSLHPYLDELRIAVEGNTNQAAAVRIACLIRQSVQSSTLIRVLFYHTPDQNHIEQPFYLMGRDKALAVEQFISRFNSGYIKASQELVSYTIKLSHDPIEYLLEQIQNLHRVTLAEGTTARYSAKRQNRISDDLIIAVIMATYLCDDIHAIRFRVS

>3NCTD

MKTELTLNVLQTMNAQEYEDIRAAGSDERRELTHAVMRELDAPDNWTMNGEYGSEFGGFFPVQVRFTPAHERFHLALCSPGDVSQVWVLVLVNAGGEPFAVVQVQRRFASEAVSHSLALAASLDTQGYSVNDIIHILMAEGGQV

>3NFHB

MTDSAIDIVDSVRTASKDLPTRAQLDEITSNDRPTPLANIDATDVEQIYPIESIIPKKELQFIRVSSILKEADKEKKLELFPYQNNSKYVAKKLDSLTQPSQMTKLQLLYYLSLLLGVYENRRVNNKTKLLERLNSPPEILVDGILSRFTVIKPGQFGRSKDRSYFIDPQNEDKILCYILAIIMHLDNFIVEITPLAHELNLKPSKVVSLFRVLGAIVKGATVAQAEAFGIPKSTAASYKIATMKV

>3NO7A

GPEASARSEVKMTVTVGEERRARLRTAYTLTHLQEGHRTFSGFIAAALDAEVQRLEQRYNEGRRFENAERGVTRGRPLGS

>3NQUA

MGPRRRSRKPEAPRRRSPSPTPTPGPSRRGPSLGASSHQHSRRRQGWLKEIRKLQKSTHLLIRKLPFSRLAREICVKFTRGVDFNWQAQALLALQEAAEAFLVHLFEDAYLLTLHAGRVTLFPKDVQLARRIRGLEEGLG

>3NR7B

GSHMSEALKILNNIRTLRAQARESTLETLEEMLEKLEVVVNERREEESAAAAEVEERTRKLQQYREMLIADGIDPNELLNSMAAAK

>3NXCA

MPPGKCLFSGVFCNMAEKQTAKRNRREEILQSLALMLESSDGSQRITTAKLAASVGVSEAALYRHFPSKTRMFDSLIEFIEDSLITRINLILKDEKDTTARLRLIVLLLLGFGERNPGLTRILTGHALMFEQDRLQGRINQLFERIEAQLRQVLREKRMREGEGYATDETLLASQILAFCEGMLSRFVRSEFKYRPTDDFDARWPLIAAQLQ

>3O27B

MRPGIRKLVVLNPRAYKGGSGHTTFYLLIPKDIAEALDIKPDDTFILNMEQKDGDIVLSYKRVKELKI

>3OA6A

MKKHHHHHHMSASEGMKFKFHSGEKVLCFEPDPTKARVLYDAKIVDVIVGKDEKGRKIPEYLIHFNGWNRSWDRWAAEDHVLRDTDENRRLQRKLARKAVARLRSTGRKK

>3OD8H

MGSSHHHHHHSSGLVPRGSHMAESSDKLYRVEYAKSGRASCKKCSESIPKDSLRMAIMVQSPMFDGKVPHWYHFSCFWKVGHSIRHPDVEVDGFSELRWDDQQKVKKTAEAGGVTG

>3OKGA

MSHHHHHHSMDIEFMTEGPYKLPPGWRWVRLGEVCLPTERRDPTKNPSTYFVYVDISAIDSTVGKIVSPKEILGQHAPSRARKVIRSGDVIFATTRPYLKNIALVPPDLDGQICSTGFCVIRANREFAEPEFLFHLCRSDFITNQLTASKMRGTSYPAVTDNDVYNTLIPLPPLEEQRRIVAKVEALMERVREVRRLRAEAQKDTELLMQTALAEVFPHPGADLPPGWRWVRLGEVCDIIMGQSPPSSTYNFEGNGLPFFQGKADFGDLHPTPRIWCSAPQKVARPGDVLISVRAPVGSTNVANLACCIGRGLAALRPRDSLERFWLLYYLHYLEPELSKMGAGSTFNAITKKDLQNVFIPLPPLEEQRRIVAYLDQIQQQVAALKRAQAETEAELKRLEQAILDKAFRGDL

>3OLCX

MSRNDKEPFFVKFLKSSDNSKCFFKALESIKEFQSEEYLQIITEEEALKIKENDRSLYICDPFSGVVFDHLKKLGCRIVGPQVVIFCMHHQRCVPRAEHPVYNMVMSDVTISCTSLEKEKREEVHKYVQMMGGRVYRDLNVSVTHLIAGEVGSKKYLVAANLKKPILLPSWIKTLWEKSQEKKITRYTDINMEDFKCPIFLGCIICVTGLCGLDRKEVQQLTVKHGGQYMGQLKMNECTHLIVQEPKGQKYECAKRWNVHCVTTQWFFDSIEKGFCQDESIYKTEPRPEALEHHHHHH

>3ON0D

MPKIQTYVNNNVYEQITDLVTIRKQEGIEEASLSNVSSMLLELGLRVYMIQQEKREGGFNQMEYNKLMLENVSRVRAMCTEILKMSVLNQESIASGNFDYAVIKPAIDKFAREQVSIFFPDDEDDQE

>3P7NB

MRGSHHHHHHGMASMTGGQQMGRDLYDDDDKDHPFTMGQDRPIDGSGAPGADDTRVEVQPPAQWVLDLIEASPIASVVSDPRLADNPLIAINQAFTDLTGYSEEECVGRNCRFLAGSGTEPWLTDKIRQGVREHKPVLVEILNYKKDGTPFRNAVLVAPIYDDDDELLYFLGSQVEVDDDQPNMGMARRERAAEMLKTLSPRQLEVTTLVASGLRNKEVAARLGLSEKTVKMHRGLVMEKLNLKTSADLVRIAVEAGI

>3P83F

GPLGSPEFPGRLMKAGIDEAGKGCVIGPLVVAGVACSDEDRLRKLGVKDSKKLSQGRREELAEEIRKICRTEVLKVSPENLDERMAAKTINEILKECYAEIILRLKPEIAYVDSPDVIPERLSRELEEITGLRVVAEHKADEKYPLVAAASIIAKVEREREIERLKEKFGDFGSGYASDPRTREVLKEWIASGRIPSCVRMRWKTVSNLRQKTLDDF

>3P83C

MIDVIMTGELLKTVTRAIVALVSEARIHFLEKGLHSRAVDPANVAMVIVDIPKDSFEVYNIDEEKTIGVDMDRIFDISKSISTKDLVELIVEDESTLKVKFGSVEYKVALIDPSAIRKEPRIPELELPAKIVMDAGEFKKAIAAADKISDQVIFRSDKEGFRIEAKGDVDSIVFHMTETELIEFNGGEARSMFSVDYLKEFCKVAGSGDLLTIHLGTNYPVRLVFELVGGRAKVEYILAPRIESE

>3P9AA

MAAPKGNRFWEARSSHGRNPKFESPEALWAACCEYFEWVEANPLWEMKAFSYQGEVIQEPIAKMRAMTITGLTLFIDVTLETWRTYRLREDLSEVVTRAEQVIYDQKFSGAAADLLNANIIARDLGLKEQSQVEDVTPDKGDRDKRRSRIKELFNRGTGRDS

>3PGGB

SYKVNYMSETPANKSQGGSNQKGGNIILPLALIDKCIGNRIYVVMKGDKEFSGVLRGFDEYVNMVLDDVQEYGFKADEEDISGGNKKLKRVMVNRLETILLSGNNVAMLVPGGDPDSFNFS

>3POVA

GAMEATPTPADLFSEDYLVDTLDGLTVDDQQAVLASLSFSKFLKHAKVRDWCAQAKIQPSMPALRMAYNYFLFSKVGEFIGSEDVCNFFVDRVFGGVRLLDVASVYAACSQMNAHQRHHICCLVERATSSQSLNPVWDALRDGIISSSKFHWAVKQQNTSKKIFSPWPITNNHFVAGPLAFGLRCEEVVKTLLATLLHPDETNCLDYGFMQSPQNGIFGVSLDFAANVKTDTEGRLQFDPNCKVYEIKCRFKYTFAKMECDPIYAAYQRLYEAPGKLALKDFFYSISKPAVEYVGLGKLPSESDYLVAYDQEWEACPRKKRKLTPLHNLIRECILHNSTTESDVYVLTDPQDTRGQISIKARFKANLFVNVRHSYFYQVLLQSSIVEEYIGLDSGIPRLGSPKYYIATGFFRKRGYQDPVNCTIGGDALDPHVEIPTLLIVTPVYFPRGAKHRLLHQAANFWSRSAKDTFPYIKWDFSYLSANVPHSP

>3PVPB

GPHMISAATIMAATAEYFDTTVEELRGPGKTRALAQSRQIAMYLCRELTDLSLPKIGQAFGRDHTTVMYAQRKILSEMAERREVFDHVKELTTRIRQRSKR

>3Q8DB

MEGWQRAFVLHSRPWSETSLMLDVFTEESGRVRLVAKGARSKRSTLKGALQPFTPLLLRFGGRGEVKTLRSAEAVSLALPLSGITLYSGLYINELLSRVLEYETRFSELFFDYLHCIQSLAGVTGTPEPALRRFELALLGHLGYGVNFTHCAGSGEPVDDTMTYRYREEKGFIASVVIDNKTFTGRQLKALNAREFPDADTLRAAKRFTRMALKPYLGGKPLKSRELFRQFMPKRTVKTHYE

>3QMBA

MHHHHHHSSRENLYFQGQIKRSARMCGECEACRRTEDCGHCDFCRDMKKFGGPNKIRQKCRLRQCQLRARESYKYFPSS

>3QO2A

HMGEDVFEVEKILDMKTEGGKVLYKVRWKGYTSDDDTWEPEIHLEDCKEVLLEFRKKIAENKAK

>3QODB

SNAMSNDVDLIKRLGPSAMDQIMLYLAFSAMRTSGHRHGAFLDAAATAAKCAIYMTYLEQGQNLRMTGHLHHLEPKRVKAIVEEVRQALTEGKLLKMLGSQEPRYLIQFPYVWMEKYPWRPGRSRIPGTSLTSEEKRQIEQKLPSNLPDAHLITSFEFLELIEFLHKRSQEDLPKEHQMPLSEALAEHIKRRLLYSGTVTRIDSPWGMPFYALTRPFYAPADDQERTYIMVEDTARFFRMMRDWAEKRPNTMRVLEELDILPEKMQQAKDELDEIIRAWADKYHQDDGVPVVLQMVFGKKED

>3QRFN

SSVPLEWPLSSQSGSYELRIEVQPKPHHRAHYETEGSRGAVKAPTGGHPVVQLHGYMENKPLGLQIFIGTADERILKPHAFYQVHRITGKTVTTTSYEKIVGNTKVLEIPLEPKNNMRATIDCAGILKLRNADIELRKGETDIGRKNTRVRLVFRVHIPESSGRIVSLQTASNPIECSQRSAHELPMVERQDTDSCLVYGGQQMILTGQNFTSESKVVFTEKTTDGQQIWEMEATVAAAAAAPNMLFVEIPEYRNKHIRTPVKVNFYVINGKRKRSQPQHFTYHPV

>3QU3A

GSHMAEVRGVQRVLFGDWLLGEVSSGQYEGLQWLNEARTVFRVPWKHFGRRDLDEEDAQIFKAWAVARGRWPPSGVNLPPPEAEAAERRERRGWKTNFRCALHSTGRFILRQDNSGDPVDPHKVYELSRELGSTVGP

>3QVGD

MQEPPDLPVPELPDFFEGKHFFLYGEFPGDERRRLIRYVTAFNGELEDRMNERVQFVITAQEWDPNFEEALMENPSLAFVRPRWIYSCNEKQKLLPHQLYGVVPQA

>3QVGC

SNSADETLCQTKVLLDIFTGVRLYLPPSTPDFSRLRRYFVAFDGDLVQEFDMTSATHVLGSRDKNPAAQQVSPEWIWACIRKRRLVAPS

>3R0JB

GTHMRKGVDLVTAGTPGENTTPEARVLVVDDEANIVELLSVSLKFQGFEVYTATNGAQALDRARETRPDAVILDVMMPGMDGFGVLRRLRADGIDAPALFLTARDSLQDKIAGLTLGGDDYVTKPFSLEEVVARLRVILRRAGKGNKEPRNVRLTFADIELDEETHEVWKAGQPVSLSPTEFTLLRYFVINAGTVLSKPKILDHVWRYDFGGDVNVVESYVSYLRRKIDTGEKRLLHTLRGVGYVLREPR

>3R4KA

GMGTVSKALTLLTYFNHGRLEIGLSDLTRLSGMNKATVYRLMSELQEAGFVEQVEGARSYRLGPQVLRLAALREASVPILSASRRVLRELSEDTGETTHLSLLQGEQLASLSHAYSSRNATKVMMEDAEVLTFHGTASGLAVLAYSEPSFVDAVLAAPLTARTPQTQTDPAAIRAEIAEVRRTGLAQSIGGFEAEVHSHAVPIFGPDRAVLGALAVAAPTSRMTPDQKRTIPPALRAAGLSLTERIGGACPPEFPTDIAA

>3RCOA

ENLYFQGMLEGDLVSKMLRAVLQSHKNGVALPRLQGEYRSLTGDWIPFKQLGFPTLEAYLRSVPAVVRIETSRSGEITCYAMACTETAR

>3RH2A

GMKTRDKIIQASLELFNEHGERTITTNHIAAHLDISPGNLYYHFRNKEDIIRCIFDQYEQHLLLGFKPYADQKVDLELLMSYFDAMFYTMWQFRFMYANLADILARDDTLKARYLKVQQAVLEQSIAVLNQLKKDGILQIEDERIADLADTIKMIIGFWISYKLTQSSIATISKASLYEGLLRVLMIFKAYSTPDSLANFDRLEQHFRSQSN

>3RLOA

GSVDDSAQSDLKEVMVLNATESFVYEPKEQKKMFHATVATENEVFRVKVFNIDLKEKFTPKKIIAIANYVCRNGFLEVYPFTLVADVNADRNMEIPKGLIRSASVTPKINQLCSQTKGSFVNGVFEVHKKNVRGEFTYYEIQDNTGKMEVVVHGRLTTINCEEGDKLKLTCFELAPKSGNTGELRSVIHSHIKVIKTRKNAAAS

>3S4WB

SHNSHEVEENGSVFVKLLKASGLTLKTGENQNQLGVDQVIFQRKLFQALRKHPAYPKVIEEFVNGLESYTEDSESLRNCLLSCERLQDEEASMGTFYSKSLIKLLLGIDILQPAIIKMLFEKVPQFLFESENRDGINMARLIINQLKWLDRIVDGKDLTAQMMQLISVAPVNLQHDFITSLPEILGDSQHANVGKELGELLVQNTSLTVPILDVFSSLRLDPNFLSKIRQLVMGKLSSVRLEDFPVIVKFLLHSVTDTTSLEVIAELRENLNVQQFILPSRIQASQSKLKSKGLASSSGNQENSDKDCIVLVFDVIKSAIRYEKTISEAWFKAIERIESAAEHKSLDVVMLLIIYSTSTQTKKGVEKLLRNKIQSDCIQEQLLDSAFSTHYLVLKDICPSILLLAQTLFHSQDQRIILFGSLLYKYAFKFFDTYCQQEVVGALVTHVCSGTEAEVDTALDVLLELIVLNASAMRLNAAFVKGILDYLENMSPQQIRKIFCILSTLAFSQQPGTSNHIQDDMHLVIRKQLSSTVFKYKLIGIIGAVTMAGIMAEDRSVPSNSSQRSANVSSEQRTQVTSLLQLVHSCTEHSPWASSLYYDEFANLIQERKLAPKTLEWVGQTIFNDFQDAFVVDFCAAPEGDFPFPVKALYGLEEYSTQDGIVINLLPLFYQECAKDASRATSQESSQRSMSSLCLASHFRLLRLCVARQHDGNLDEIDGLLDCPLFLPDLEPGEKLESMSAKDRSLMCSLTFLTFNWFREVVNAFCQQTSPEMKGKVLSRLKDLVELQGILEKYLAVIPDYVPPFASVDLDTLDMMPRKTFVSLQNYRAFFRELDIEVFSILHSGLVTKFILDTEMHTEATEVVQLGPAELLFLLEDLSQKLENMLTAPFAKRICCFKNKGRQNIGFSHLHQRSVQDIVHCVVQLLTPMCNHLENIHNFFQCLGAEHLSADDKARATAQEQHTMACCYQKLLQVLHALFAWKGFTHQSKHRLLHSALEVLSNRLKQMEQDQPLEELVSQSFSYLQNFHHSVPSFQCGLYLLRLLMALLEKSAVPNQKKEKLASLAKQLLCRAWPHGEKEKNPTFNDHLHDVLYIYLEHTDNVLKAIEEITGVGVPELVSAPKDAASSTFPTLTRHTFVIFFRVMMAELEKTVKGLQAGTAADSQQVHEEKLLYWNMAVRDFSILLNLMKVFDSYPVLHVCLKYGRRFVEAFLKQCMPLLDFSFRKHREDVLSLLQTLQLNTRLLHHLCGHSKIRQDTRLTKHVPLLKKSLELLVCRVKAMLVLNNCREAFWLGTLKNRDLQGEEIISQDPSSSESNAEDSEDG

>3S4WA

MDLKILSLATDKTTDKLQEFLQTLKDDDLASLLQNQAVKGRAVGTLLRAVLKGSPCSEEDGALRRYKIYSCCIQLVESGDLQQDVASEIIGLLMLEVHHFPGPLLVDLASDFVGAVREDRLVNGKSLELLPIILTALATKKEVLACGKGDLNGEEYKRQLIDTLCSVRWPQRYMIQLTSVFKDVCLTPEEMNLVVAKVLTMFSKLNLQEIPPLVYQLLVLSSKGSRRSVLDGIIAFFRELDKQHREEQSSDELSELITAPADELYHVEGTVILHIVFAIKLDCELGRELLKHLKAGQQGDPSKCLCPFSIALLLSLTRIQRFEEQVFDLLKTSVVKSFKDLQLLQGSKFLQTLVPQRTCVSTMILEVVRNSVHSWDHVTQGLIEFGFILMDSYGPKKILDGKAVEIGTSLSKMTNQHACKLGANILLETFKIHEMIRQEILEQVLNRVVTRTSSPINHFLDLFSDIIMYAPLILQNCSKVTETFDYLTFLPLQTVQGLLKAVQPLLKISMSMRDSLILVLRKAMFASQLDARKSAVAGFLLLLKNFKVLGSLPSSQCTQSIGVTQVRVDVHSRYSAVANETFCLEIIDSLKRSLGQQADIRLMLYDGFYDVLRRNSQLASSIMQTLFSQLKQFYEPEPDLLPPLKLGACVLTQGSQIFLQEPLDHLLSCIQHCLAWYKSRVVPLQQGDEGEEEEEELYSELDDMLESITVRMIKSELEDFELDKSADFSQNTNVGIKNNICACLIMGVCEVLMEYNFSISNFSKSKFEEILSLFTCYKKFSDILSEKAGKGKAKMTSKVSDSLLSLKFVSDLLTALFRDSIQSHEESLSVLRSSGEFMHYAVNVTLQKIQQLIRTGHVSGPDGQNPDKIFQNLCDITRVLLWRYTSIPTSVEESGKKEKGKSISLLCLEGLQKTFSVVLQFYQPKVQQFLQALDVMGTEEEEAGVTVTQRASFQIRQFQRSLLNLLSSEEDDFNSKEALLLIAVLSTLSRLLEPTSPQFVQMLSWTSKICKEYSQEDASFCKSLMNLFFSLHVLYKSPVTLLRDLSQDIHGQLGDIDQDVEIEKTDHFAVVNLRTAAPTVCLLVLSQAEKVLEEVDWLIAKIKGSANQETLSDKVTPEDASSQAVPPTLLIEKAIVMQLGTLVTFFHELVQTALPSGSCVDTLLKGLSKIYSTLTAFVKYYLQVCQSSRGIPNTVEKLVKLSGSHLTPVCYSFISYVQNKSSDAPKCSEKEKAAVSTTMAKVLRETKPIPNLVFAIEQYEKFLIQLSKKSKVNLMQHMKLSTSRDFKIKGSVLDMVLREDEEHHHHHH

>3S5RB

GMQATMTDKNTRELLLDAATTLFAEQGIAATTMAEIAASVGVNPAMIHYYFKTRDSLLDTIIEERIGRIIDMIWEPVTGEEDDPLIMVRDLVNRIVNTCETMLWLPSLWIREIVNEGGALREKMLNNIPIDKMNKFSAKIAEGQKQGVINSGIDSRLLIGSIIGLTMLPLATAKLRDQIPTMKGLSSEDIVCHVTALLFTGLTNPSNSDDIKKQRT

>3SIAA

MQPPVANFCLWNLQPIQGSWMGAACIYQMPPSVRNTWWFPLLNTIPLDQYTRIYQWFMGVDRDRSGTLEINELMMGQFPGGIRLSPQTALRMMRIFDTDFNGHISFYEFMAMYKFMELAYNLFVMNARARSGTLEPHEILPALQQLGFYINQRTSLLLHRLFARGMAFCDLNCWIAICAFAAQTRSAYQMIFMNPYYGPMKPFNPMEFGKFLDVVTSLLE

>3SQIA

MSKLDSLLKELPTRTAHLYRSIWHKYTEWLKTMPDLTGADLKLFLSQKYIVKYIASHDDIAKDPLPTCDAMIWFSRALDIENNDVLVLQQRLYGLVKLLEFDYSNVIAILQKISINLWNPSTDSLQSKHFKTCQDKLKLLLDFQWKFNTNVSFEDRTTVSLKDLQCILDDENGKCGLAHSSKPNFVLVPNFQSPFTCPIFTMAVYYYLRFHGVKKYYKGDGYQILSQLEHIPIIRGKSLDQYPRELTLGNWYPTIFKYCQLPYTKKHWFQVNQEWPQFPDFSDSSENTSTLAESDSENTIGIPDFYIEKMNRTKLQPCPQVHVHLFPTDLPPDIQAVFDLLNSVLVTSLPLLYRVFPTHDIFLDPSLKTPQNIAFLTGTLPLDIESQEHLLAQLIDKTGTVSELVPNPVKIDQNEHTLTPIGTSLSQTDIPMLDQLKTELQKLIQLQTSTGFSQLITVLLEIFQRLDFKKSNKQFVIDLLQSCRKDMRNKLMDPCSLSTNFADELSDDENEKGNKTGAIYDPETDNGNEESVSD

>3SSCB

MESIQPWIEKFIKQAQQQRSQSTKDYPTSYRNLRVKLSFGYGNFTSIPWFAFLGEGQEASNGIYPVILYYKDFDELVLAYGISDTNEPHAQWQFSSDIPKTIAEYFQATSGVYPKKYGQSYYACSQKVSQGIDYTRFASMLDNIINDYKLIFNSGKSVIPPLEGHHHHHH

>3TB6B

GIDPFTSAKSALHSNKTIGVLTTYISDYIFPSIIRGIESYLSEQGYSMLLTSTNNNPDNERRGLENLLSQHIDGLIVEPTKSALQTPNIGYYLNLEKNGIPFAMINASYAELAAPSFTLDDVKGGMMAAEHLLSLGHTHMMGIFKADDTQGVKRMNGFIQAHRERELFPSPDMIVTFTTEEKESKLLEKVKATLEKNSKHMPTAILCYNDEIALKVIDMLREMDLKVPEDMSIVGYDDSHFAQISEVKLTSVKHPKSVLGKAAAKYVIDCLEHKKPKQEDVIFEPELIIRQSARKLNE

>3TEDA

GPDMDSIGESEVRALYKAILKFGNLKEILDELIADGTLPVKSFEKYGETYDEMMEAAKDCVHEEEKNRKEILEKLEKHATAYRAKLKSGEIKAENQPKDNPLTRLSLKKREKKAVLFNFKGVKSLNAESLLSRVEDLKYLKNLINSNYKDDPLKFSLGNNTPKPVQNWSSNWTKEEDEKLLIGVFKYGYGSWTQIRDDPFLGITDKIFLNEVHNPVAKKSASSSDTTPTPSKKGKGITGSSKKVPGAIHLGRRVDYLLSFLRGGLNTKSPS

>3TEKB

MGEELREEERGEVRSELITKGEKKLVLIRWNTGKTSAGRLFGRYGPGGRPEFFKLLFGAVAGSLREQFGPDGENIFNRIRDSEKFRETSRELFDGLKKWFFEEAVPRYNLERGDIFMISTELVLDPDTGELLWNRDKTQLIYWIRSDR

>3THOB

MKILHTSDWHLGVTSWTSSRPVDRREELKKALDKVVEEAEKREVDLILLTGDLLHSRNNPSVVALHDLLDYLKRMMRTAPVVVLPGNQDWKGLKLFGNFVTSISSDITFVMSFEPVDVEAKRGQKVRILPFPYPDESEALRKNEGDFRFFLESRLNKLYEEALKKEDFAIFMGHFTVEGLAGYAGIEQGREIIINRALIPSVVDYAALGHIHSFREIQKQPLTIYPGSLIRIDFGEEADEKGAVFVELKRGEPPRYERIDASPLPLKTLYYKKIDTSALKSIRDSCRNFPGYVRVVYEEDSGILPDLMGEIDNLVKIERKSRREIEEVLRESPEEFKEELDKLDYFELFKEYLKKREENHEKLLKILDELLDEVKKSEA

>3THOA

HHHHHHSSGENLYFQGHMRPERLTVRNFLGLKNVDIEFQSGITVVEGPNGAGKSSLFEAISFALFGNGIRYPNSYDYVNRNAVDGTARLVFQFERGGKRYEIIREINALQRKHNAKLSEILENGKKAAIAAKPTSVKQEVEKILGIEHRTFIRTVFLPQGEIDKLLISPPSEITEIISDVFQSKETLEKLEKLLKEKMKKLENEISSGGAGGAGGSLEKKLKEMSDEYNNLDLLRKYLFDKSNFSRYFTGRVLEAVLKRTKAYLDILTNGRFDIDFDDEKGGFIIKDWGIERPARGLSGGERALISISLAMSLAEVASGRLDAFFIDEGFSSLCTENKEKIASVLKELERLNKVIVFITCDREFSEAFDRKLRITGGVVVNE

>3THWD

CCTCTATCTGAAGCCGATCGATGAAGCATCGATCGCACAGCTTCAGATAGAGG

>3TOCB

GSFTMNLNFSLLDEPIPLRGGTILVLEDVCVFSKIVQYCYQYEEDSELKFFDHKMKTIKESEIMLVTDILGFDVNSSTILKLIHADLESQFNEKPEVKSMIDKLVATITELIVFECLENELDLEYDEITILELIKSLGVKVETQSDTIFEKCLEILQIFKYLTKKKLLIFVNSGAFLTKDEVASLQEYISLTNLTVLFLEPRELYDFPQYILDEDYFLITKNMV

>3TRBB

SNAMAANRMRPIHPGEILAEELGFLDKMSANQLAKHLAIPTNRVTAILNGARSITADTALRLAKFFGTTPEFWLNLQDAYDIKMALKKSGKKIEKEVTPYDQAA

>3TUOD

GPGSGTMLPVFCVVEHYENAIEYDCKEEHAEFVLVRKDMLFNQLIEMALLSLGYSHSSAAQAKGLIQVGKWNPVPLSYVTDAPDATVADMLQDVYHVVTLKIQLH

>3U50C

QRIYSSIEEIIQQAQASEIGQKKEFYVYGNLVSIQMKNKLYYYRCTCQGKSVLKYHGDSFFCESCQQFINPQVHLMLRAFVQDSTGTIPVMIFDQQSSQLINQIDPSIHVQEAGQYVKNCIENGQEEIIRQLFSKLDFARFIFEIQFENKEFNNEQEIAYKVLKIEKENIKE

>3U58A

GSLSDQLSKQTLLISQLQVGKNRFSFKFEGRVVYKSSTFQNQQDSKYFFITAQDANNQEINLSFWQKVDQSYQTLKVGQYYYFIGGEVKQFKNNLELKFKFGDYQIIPKETLGGSGGSTLLISEVLKTSKQYLSVLAQVVDIQSSDKNIRLKICDNSCNQELKVVIFPDLCYEWRDKFSINKWYYFNEFVRQIYNDEVQLKNNIHSSIKESDD

>3U5ZF

MKLSKDTTALLKNFATINSGIMLKSGQFIMTRAVNGTTYAEANISDVIDFDVAIYDLNGFLGILSLVNDDAEISQSEDGNIKIADARSTIFWPAADPSTVVAPNKPIPFPVASAVTEIKAEDLQQLLRVSRGLQIDTIAITVKEGKIVINGFNKVEDSALTRVKYSLTLGDYDGENTFNFIINMANMKMQPGNYKLLLWAKGKQGAAKFEGEHANYVVALEADSTHDF

>3U5ZA

SLFKDDIQLNEHQVAWYSKDWTAVQSAADSFKEKAENEFFEIIGAINNKTKCSIAQKDYSKFMVENALSQFPECMPAVYAMNLIGSGLSDEAHFNYLMAAVPRGKRYGKWAKLVEDSTEVLIIKLLAKRYQVNTNDAINYKSILTKNGKLPLVLKELKGLVTDDFLKEVTKNVKEQKQLKKLALEWGLEHHHHHHHHHH

>3U5ZM

GPGGSMITVNEKEHILEQKYRPSTIDECILPAFDKETFKSITSKGKIPHIILHSPSPGTGKTTVAKALCHDVNADMMFVNGSDCKIDFVRGPLTNFASAASFDGRQKVIVIDEFDRSGLAESQRHLRSFMEAYSSNCSIIITANNIDGIIKPLQSRCRVITFGQPTDEDKIEMMKQMIRRLTEICKHEGIAIADMKVVAALVKKNFPDFRKTIGELDSYSSKGVLDAGILSLVTNDRGAIDDVLESLKNKDVKQLRALAPKYAADYSWFVGKLAEEIYSRVTPQSIIRMYEIVGENNQYHGIAANTELHLAYLFIQLACEMQWK

>3ULJA

GSDPQVLRGSGHCKWFNVRMGFGFISMTSREGSPLENPVDVFVHQSKLYMEGFRSLKEGEPVEFTFKKSSKGFESLRVTGPGGNPCLGNE

>3ULXA

MGMRRERDAEAELNLPPGFRFHPTDDELVEHYLCRKAAGQRLPVPIIAEVDLYKFDPWDLPERALFGAREWYFFTPRDRKYPNGSRPNRAAGNGYWKATGADKPVAPRGRTLGIKKALVFYAGKAPRGVKTDWIMHEYRLADAGRAAAGAKKGSLRLDDWVLCRLYNKKNEWEK

>3UWXB

MGSSHHHHHHSSGLVPRGSHMGPKKVEGRFQLVSPYEPQGDQPQAIAKLVDGLRLGVKHQTLLGATGTGKTFTISNVIAQVNKPTLVIAHNKTLAGQLHSELKEFFPHNAVEYFVSYYDYYQPEAYVPQTDTYIEKDAKINDEIDKLRHSATSALFERRDVIIVASVSCIYGLGSPEEYRELVVSLRVGMEIERNALLRRLVDIQYDRNDIDFRRGTFRVRGDVVEIFPASRDEHCIRVEFFGDEIERIREVDALTGEVLGEREHVAIFPASHFVTREEKMRLAIQNIEQELEERLAELRAQGKLLEAQRLEQRTRYDLEMMREMGFCSGIENYSRHLALRPPGSTPYTLLDYFPDDFLIIVDESHVTLPQLRGMYNGDRARKQVLVDHGFRLPSALDNRPLTFEEFEQKINQIIYISATPGPYELEHSPGVVEQIIRPTGLLDPTIDVRPTKGQIDDLIGEIHERVERNERTLVTTLTKKMAEDLTDYLKEAGIKVAYLHSEIKTLERIEIIRDLRLGKYDVLVGINLLREGLDIPEVSLVAILDADKEGFLRSERSLIQTIGRAARNANGHVIMYADTITKSMEIAIQETKRRRAIQEEYNRKHGIVPRTVKKEIRDVIRATYAAEETEMYEAKPAAAMTKQEREELIRKLEAEMKEAAKALDFERAAQLRDIIFELKAEG

>3V20B

MTTNLTNSNCVEEYKENGKTKIRIKPFNALIELYHHQTPTGSIKENLDKLENYVKDVVKAKGLAIPTSGAFSNTRGTWFEVMIAIQSWNYRVKRELNDYLIIKMPNVKTFDFRKIFDNETREKLHQLEKSLLTHKQQVRLITSNPDLLIIRQKDLIKSEYNLPINKLTHENIDVALTLFKDIEGKCKWDSLVAGVGLKTSLRPDRRLQLVHEGNILKSLFAHLKMAYWNPKAEFKYYGASSEPVSKADDDALQTAATHTIVNVNSTPERAVDDIFSLTSFEDIDKMLDQIIKK

>3V4GA

MHHHHHHSSGVDLGTENLYFQSNAMRPSEKQDNLVRAFKALLKEERFGSQGEIVEALKQEGFENINQSKVSRMLTKFGAVRTRNAKMEMVYCLPTELGVPTVSSSLRELVLDVDHNQALVVIHTGPGAAQLIARMLDSLGKSEGILGVVAGDDTIFITPTLTITTEQLFKSVCELFEYAG

>3V60A

GSHMRPETHINLKVSDGSSEIFFKIKKTTPLRRLMEAFAKRQGKEMDSLRFLYDGIRIQADQTPEDLDMEDNDIIEAHREQIGG

>3V62C

SHNPDDTTVDNRPIISNAKFLADAAMKKTQKFSKKVKNEPASSQMDIFSQLSRAKKKSKLNNGEIIVID

>3V68A

MIERILEFTAKHEEWIVGENVEDFTNENIAMFLSRVSNTVSSKIPGYLGEKIDVNGLLSIKIEGSLEEKLKALISPKVSRQIGRLVMEDDKKLKKLLVEVAKAVLTREILKNELPIEFPGGKIEGLKIQPRYEEDHINFTARYGSWIVVKRMIIDEKTPLLDIARLLASINETAVNKIKDFADVDDKKIVEYFGGFKKVKKEEEIKEIVQLFREFKGNEFEVRYAAREMLSKLGLKVDVPSKNLEKYLEKAG

>3V72A

MSKRKAPQETLNGGITDMLVELANFEKNVSQAIHKYNAYRKAASVIAKYPHKIKSGAEAKKLPGVGTKIAEKIDEFLATGKLRKLEKIRQDDTSSSINFLTRVTGIGPSAARKLVDEGIKTLEDLRKNEDKLNHHQRIGLKYFEDFEKRIPREEMLQMQDIVLNEVKKLDPEYIATVCGSFRRGAESSGDMDVLLTHPNFTSESSKQPKLLHRVVEQLQKVRFITDTLSKGETKFMGVCQLPSENDENEYPHRRIDIRLIPKDQYYCGVLYFTGSDIFNKNMRAHALEKGFTINKYTIRPLGVTGVAGEPLPVDSEQDIFDYIQWRYREPKDRSE

>3V9RD

MLSKEALIKILSQNEGGNDMKIADEVVPMIQKYLDIFIDEAVLRSLQSHKDINGERGDKSPLELSHQDLERIVGLLLMDMLEHHHHHH

>3V9RC

MNDDEDRAQLKARLWIRVEERLQQVLSSEDIKYTPRFINSLLELAYLQLGEMGSDLQAFARHAGRGVVNKSDLMLYLRKQPDLQERVTQE

>3VEAA

MKYQQLENLESGWKWAYLVKKHREGEAITRHIENSAAQDAVEQLMKLENEPVKVQEWIDAHMNVNLATRMKQTIRARRKRHFNAEHQHTRKKSIDLEFLVWQRLAVLARRRGNTLSDTVVQLIEDAERKEKYASQMSSLKQDLKDILDKEV

>3VH5W

GYRRTVPRGTLRKIIKKHKPHLRLAANTDLLVHLSFLLFLHRLAEEARTNAFENKSKIIKPEHTIAAAKVILKKSRG

>3VH5A

GSEAAGGEQRELLIQRLRAAVHYTTGALAQDVAEDKGVLFSKQTVAAISEITFRQAENFARDLEMFARHAKRSTITSEDVKLLARRSNSLLKYITQKSDELASSNMEQKEKKKKKSSAAKGRKTEENETPVTESEDSNMA

>3VIBA

MRKTKTEALKTKEHLMLAALETFYRKGIARTSLNEIAQAAGVTRDALYWHFKNKEDLFDALFQRICDDIENCIAQDAADAEGGSWTVFRHTLLHFFERLQSNDIHYKFHNILFLKCEHTEQNAAVIAIARKHQAIWREKITAVLTEAVENQDLADDLDKETAVIFIKSTLDGLIWRWFSSGESFDLGKTAPRIIGIMMDNLENHPCLRRK

>3VK0C

MMGNKLTLPAELPDEQDLRAVLAYNMRLFRVNKGWSQEELARQCGLDRTYVSAVERKRWNIALSNIEKMAAALGVAAYQLLLPPQERLKLMTNSADTRQMPSESGILEHHHHHH

>3VPRD

VTTTRDRILEEAAKLFTEKGYEATSVQDLAQALGLSKAALYHHFGSKEEILYEISLLALKGLVAAGEKALEVADPKEALRRFMEAHARYFEENYPFFVTMLQGIKSLSPENRLKTIALRDRHEENLRAILRRGVEQGVFREVDVALAGRAVLSMLNWMIRWFRPDGPMRAEEVARAYHDLILRGLERGSA

>3VW4A

MGHHHHHHRNYHLFEKVRKWAYRAIRQGWPVFSQWLDAVIQRVEMYNASLPVPLSPAECRAIGKSIAKYTHRKFSPEGFSAVQAARGRKGGTKSKRAAVPTSARSLKPWEALGISRATYYRKLKCDPD

>3VZHA

GHMYRSRDFYVRVSGQRALFTNPATKGGSERSSYSVPTRQALNGIVDAIYYKPTFTNIVTEVKVINQIQTELQGVRALLHDYSADLSYVSYLSDVVYLIKFHFVWNEDRKDLNSDRLPAKHEAIMERSIRKGGRRDVFLGTRECLGLVDDISQEEYETTVSYYNGVNIDLGIMFHSFAYPKDKKTPLKSYFTKTVMKNGVITFKAQSECDIVNTLSSYAFKAPEEIKSVNDECMEYDAMEKGEN

>3W03B

SGMEELEQGLLMQPWAWLQLAENSLLAKVFITKQGYALLVSDLQQVWHEQVDTSVVSQRAKELNKRLTAPPAAFLCHLDNLLRPLLKDAAHPSEATFSCDCVADALILRVRSELSGLPFYWNFHCMLASPSLVSQHLIRPLMGMSLALQCQVRELATLLHMKDLEIQDYQESGATLIRDRLKTEPFEENSFLEQFMIEKLPEACSIGDGKPFVMNLQDLYMAVTTQEVQVGQKHQ

>3W3WB

PRRRTVGMKSSQGNVPTGNKQSVGKSAKISKPLHIKTSAYQKQYKINLETKARPSAGDEDSAHPDKNKE

>3W3WA

MSALPEEVNRTLLQIVQAFASPDNQIRSVAEKALSEEWITENNIEYLLTFLAEQAAFSQDTTVAALSAVLFRKLALKAPITHIRKEVLAQIRSSLLKGFLSERADSIRHKLSDAIAECVQDDLPAWPELLQALIESLKSGNPNFRESSFRILTTVPYLITAVDINSILPIFQSGFTDASDNVKIAAVTAFVGYFKQLPKSEWSKLGILLPSLLNSLPRFLDDGKDDALASVFESLIELVELAPKLFKDMFDQIIQFTDMVIKNKDLEPPARTTALELLTVFSENAPQMCKSNQNYGQTLVMVTLIMMTEVSIDDDDAAEWIESDDTDDEEEVTYDHARQALDRVALKLGGEYLAAPLFQYLQQMITSTEWRERFAAMMALSSAAEGCADVLIGEIPKILDMVIPLINDPHPRVQYGCCNVLGQISTDFSPFIQRTAHDRILPALISKLTSECTSRVQTHAAAALVNFSEFASKDILEPYLDSLLTNLLVLLQSNKLYVQEQALTTIAFIAEAAKNKFIKYYDTLMPLLLNVLKVNNKDNSVLKGKCMECATLIGFAVGKEKFHEHSQELISILVALQNSDIDEDDALRSYLEQSWSRICRILGDDFVPLLPIVIPPLLITAKATQDVGLIEEEEAANFQQYPDWDVVQVQGKHIAIHTSVLDDKVSAMELLQSYATLLRGQFAVYVKEVMEEIALPSLDFYLHDGVRAAGATLIPILLSCLLAATGTQNEELVLLWHKASSKLIGGLMSEPMPEITQVYHNSLVNGIKVMGDNCLSEDQLAAFTKGVSANLTDTYERMQDRHGDGDEYNENIDEEEDFTDEDLLDEINKSIAAVLKTTNGHYLKNLENIWPMINTFLLDNEPILVIFALVVIGDLIQYGGEQTASMKNAFIPKVTECLISPDARIRQAASYIIGVCAQYAPSTYADVCIPTLDTLVQIVDFPGSKLEENRSSTENASAAIAKILYAYNSNIPNVDTYTANWFKTLPTITDKEAASFNYQFLSQLIENNSPIVCAQSNISAVVDSVIQALNERSLTEREGQTVISSVKKLLGFLPSSDAMAIFNRYPADIMEKVHKWFA

>3WE2B

GPLGSKTKDYKTRDVTDDVKSIVRFVQEHSSSQGMRNIKHVGPSGRFTMNMLVDIFLGSKSAKIQSGIFGKGSAYSRHNAERLFKKLILDKILDEDLYINANDQAIAYVMLGNKAQTVLNGNLKVDFMETENSSSVKKQKALVAKVS

>3ZLJD

PNAAATQVDGTQMSLLSVPEETSPAVEALENLDPRSLTPRQALEWIYRLKSLV

>3ZQJA

MGHHHHHHHHHHSSGHIEGRHMADRLIVKGAREHNLRSVDLDLPRDALIVFTGLSGSGKSSLAFDTIFAEGQRRYVESLSAYARQFLGQMDKPDVDFIEGLSPAVSIDQKSTNRNPRSTVGTITEVYDYLRLLYARAGTPHCPTCGERVARQTPQQIVDQVLAMPEGTRFLVLAPVVRTRKGEFADLFDKLNAQGYSRVRVDGVVHPLTDPPKLKKQEKHDIEVVVDRLTVKAAAKRRLTDSVETALNLADGIVVLEFVDHELGAPHREQRFSEKLACPNGHALAVDDLEPRSFSFNSPYGACPECSGLGIRKEVDPELVVPDPDRTLAQGAVAPWSNGHTAEYFTRMMAGLGEALGFDVDTPWRKLPAKARKAILEGADEQVHVRYRNRYGRTRSYYADFEGVLAFLQRKMSQTESEQMKERYEGFMRDVPCPVCAGTRLKPEILAVTLAGESKGEHGAKSIAEVCELSIADCADFLNALTLGPREQAIAGQVLKEIRSRLGFLLDVGLEYLSLSRAAATLSGGEAQRIRLATQIGSGLVGVLYVLDEPSIGLHQRDNRRLIETLTRLRDLGNTLIVVEHDEDTIEHADWIVDIGPGAGEHGGRIVHSGPYDELLRNKDSITGAYLSGRESIEIPAIRRSVDPRRQLTVVGAREHNLRGIDVSFPLGVLTSVTGVSGSGKSTLVNDILAAVLANRLNGARQVPGRHTRVTGLDYLDKLVRVDQSPIGRTPRSNPATYTGVFDKIRTLFAATTEAKVRGYQPGRFSFNVKGGRCEACTGDGTIKIEMNFLPDVYVPCEVCQGARYNRETLEVHYKGKTVSEVLDMSIEEAAEFFEPIAGVHRYLRTLVDVGLGYVRLGQPAPTLSGGEAQRVKLASELQKRSTGRTVYILDEPTTGLHFDDIRKLLNVINGLVDKGNTVIVIEHNLDVIKTSDWIIDLGPEGGAGGGTVVAQGTPEDVAAVPASYTGKFLAEVVGGGASAATSRSNRRRNVSA

>4A0AA

MHHHHHHVDENLYFQGGGRMSYNYVVTAQKPTAVNGCVTGHFTSAEDLNLLIAKNTRLEIYVVTAEGLRPVKEVGMYGKIAVMELFRPKGESKDLLFILTAKYNACILEYKQSGESIDIITRAHGNVQDRIGRPSETGIIGIIDPECRMIGLRLYDGLFKVIPLDRDNKELKAFNIRLEELHVIDVKFLYGCQAPTICFVYQDPQGRHVKTYEVSLREKEFNKGPWKQENVEAEASMVIAVPKPFGGAIIIGQESITYHNGDKYLAIAPPIIKQSTIVCHNRVDPNGSRYLLGDMEGRLFMLLLEKEEQMDGTVTLKDLRVELLGETSIAECLTYLDNGVVFVGSRLGDSQLVKLNVDSNEQGSYVVAMETFTNLGPIVDMCVVDLERQGQGQLVTCSGAFKEGSLRIIRNGIGIHEHASIDLPGIKGLWPLRSDPNRETDDTLVLSFVGQTRVLMLNGEEVEETELMGFVDDQQTFFCGNVAHQQLIQITSASVRLVSQEPKALVSEWKEPQAKNISVASCNSSQVVVAVGRALYYLQIHPQELRQISHTEMEHEVACLDITPLGDSNGLSPLCAIGLWTDISARILKLPSFELLHKEMLGGEIIPRSILMTTFESSHYLLCALGDGALFYFGLNIETGLLSDRKKVTLGTQPTVLRTFRSLSTTNVFACSDRPTVIYSSNHKLVFSNVNLKEVNYMCPLNSDGYPDSLALANNSTLTIGTIDEIQKLHIRTVPLYESPRKICYQEVSQCFGVLSSRIEVQDTSGGTTALRPSASTQALSSSVSSSKLFSSSTAPHETSFGEEVEVHNLLIIDQHTFEVLHAHQFLQNEYALSLVSCKLGKDPNTYFIVGTAMVYPEEAEPKQGRIVVFQYSDGKLQTVAEKEVKGAVYSMVEFNGKLLASINSTVRLYEWTTEKELRTECNHYNNIMALYLKTKGDFILVGDLMRSVLLLAYKPMEGNFEEIARDFNPNWMSAVEILDDDNFLGAENAFNLFVCQKDSAATTDEERQHLQEVGLFHLGEFVNVFCHGSLVMQNLGETSTPTQGSVLFGTVNGMIGLVTSLSESWYNLLLDMQNRLNKVIKSVGKIEHSFWRSFHTERKTEPATGFIDGDLIESFLDISRPKMQEVVANLQYDDGSGMKREATADDLIKVVEELTRIH

>4A11B

MLGFLSARQTGLEDPLRLRRAESTRRVLGLELNKDRDVERIHGGGINTLDIEPVEGRYMLSGGSDGVIVLYDLENSSRQSYYTCKAVCSIGRDHPDVHRYSVETVQWYPHDTGMFTSSSFDKTLKVWDTNTLQTADVFNFEETVYSHHMSPVSTKHCLVAVGTRGPKVQLCDLKSGSCSHILQGHRQEILAVSWSPRYDYILATASADSRVKLWDVRRASGCLITLDQHNGKKSQAVESANTAHNGKVNGLCFTSDGLHLLTVGTDNRMRLWNSSNGENTLVNYGKVCNNSKKGLKFTVSCGCSSEFVFVPYGSTIAVYTVYSGEQITMLKGHYKTVDCCVFQSNFQELYSGSRDCNILAWVPSLYEPVPDDDETTTKSQLNPAFEDAWSSSDEEGGTSAWSHPQFEK

>4ACOA

MRSSILFLLKLMKIMDVQQQQEAMSSEDRFQELVDSLKPRTAHQYKTYYTKYIQWCQLNQIIPTPEDNSVNSVPYKDLPISAELIHWFLLDTLITDDKPGEKREETEDLDEEEENSFKIATLKKIIGSLNFLSKLCKVHENPNANIDTKYLESVTKLHTHWIDSQKAITTNETNNTNTQVLCPPLLKVSLNLWNPETNHLSEKFFKTCSEKLRFLVDFQLRSYLNLSFEERSKIRFGSLKLGKRDRDAIIYHKVTHSAEKKDTPGHHQLLALLPQDCPFICPQTTLAAYLYLRFYGIPSVSKGDGFPNLNADENGSLLQDIPILRGKSLTTYPREETFSNYYTTVFRYCHLPYKRREYFNKCNLVYPTWDEDTFRTFFNEENHGNWLEQPEAFAFPDKIPFDFKKIMNFKSPYTSYSTNAKKDPFPPPKDLLVQIFPEIDEYKRHDYEGLSQNSRDFLDLMEVLRERFLSNLPWIYKFFPNHDIFQDPIFGNSDFQSYFNDKTIHSKGSPILSFDILPGFNKIYKNKTNFYSLLIERPSQLTFASSHNPDTHPTQKQESEGPLQMSQLDTTQLNELLKQQSFEYVQFQTLSNFQILLSVFNKIFEKLEMKKSSRGYILHQLNLFKITLDERIKKSKIDDADKFIRDNQPIKKEENIVNEDGPNTSRRTKRPKQIRLLSIADSSDESSTEDSNVFKKDGESIEDGAYGENEDENDSEMQEQLKSMINELINSKISTFLRDQMDQFELKINALLDKILEEKVTRIIEQKLGSHTGKFSTLKRPQLYMTEEHNVGFDMEVPKKLRTSGKYAETVKDNDDHQAMSTTASPSPEQDQEAKSYTDEQEFMLDKSIDSIEGIILEWFTPNAKYANQCVHSMNKSGNKSWRANCEALYKERKSIVEFYIYLVNHESLDRYKAVDICEKLRDQNEGSFSRLAKFLRKWRHDHQNSFDGLLVYLSN

>4AD8A

GIDPFTMTRKARTPKAAPVPEAVAVVEPPPPDAAPTGPRLSRLEIRNLATITQLELELGGGFCAFTGETGAGKSIIVDALGLLLGGRANHDLIRSGEKELLVTGFWGDGDESEADSASRRLSSAGRGAARLSGEVVSVRELQEWAQGRLTIHWQHSAVSLLSPANQRGLLDRRVTKEAQAYAAAHAAWREAVSRLERLQASQRERARQIDLLAFQVQEISEVSPDPGEEEGLNTELSRLSNLHESSKHPTSLVPRGSGSAADPEALDRVEARLSALSKLKNKYGPTLEDVVEFGAQAAEELAGLEEDERDAGSLQADVDALHAELLKVGQALDAAREREAEPLVDSLLAVIRELGMPHARMEFALSALAEPAAYGLSDVLLRFSANPGEELGPLSDVASGGELSRVMLAVSTVLGADTPSVVFDEVDAGIGGAAAIAVAEQLSRLADTRQVLVVTHLAQIAARAHHHYKVEKQVEDGRTVSHVRLLTGDERLEEIARMLSGNTSEAALEHARELLAG

>4ATKB

ANIKRELTACIFPTESEARALAKERQKKDNHNLIERRRRFNINDRIKELGTLIPKSNDPDMRWNKGTILKASVDYIRKLQREQQRAKDLENRQKKLEHANRHLLLRVQELEMQARAHG

>4BHXB

GHMTDSEFFHQRFRNLIYVEFVGPRKTLIKLRNLCLDWLQPETRTKEEIIELLVLEQYLTIIPEKLKPWVRAKKPENCEKLVTLLENYKEMYQP

>4BJ1A

GGGRVDHVFYQKFKSMALQELGTNYLSISYVPSLSKFLSKNLRSMKNCIVFFDKVEHIHQYAGIDRAVSETLSLVDINVVIIEMNDYLMKEGIQSSKSKECIESMGQASYSGQLDFEASEKPSNHTSDLMMMVMRKINNDESIDHIVYFKFEQLDKLSTSTIIEPSKLTEFINVLSVLEKSNNIAFKVLIYSNNVSISSLLSTSLKKKLNTKYTVFEMPILTCAQEQEYLKKMIKFTFDSGSKLLQSYNSLVTCQLNNKESNLAIFFEFLKVFPHPFTYLFNAYTEIIVQSRTFDELLDKIRNRLTIKNYPHSAYNFKK

>4BJIA

GAMGKGQCRVWIITTNMGVESVPTCRHSKLGEPSKTIQEVIEALKPLFEKRPVWTRRALLNHLDPSYTHYLKFALPYLSYLWTSGPFRDTYTRFGYDPRKDSNAAAYQALFFKLKLNGKHKGTKTHVFDGKTLFPTNRVYQVCDIVDPTIAPLLKDTQLRSECHRDTGWYRSGRYYKVRDLMREKLFALIEGEMPSEVAVNMILNAEEVEESDRY

>4BJXA

GSSHHHHHHSSSMNPPPPETSNPNKPKRQTNQLQYLLRVVLKTLWKHQFAWPFQQPVDAVKLNLPDYYKIIKTPMDMGTIKKRLENNYYWNAQECIQDFNTMFTNCYIYNKPGDDIVLMAEALEKLFLQKINELPTEEQELVVTIPNSHKKGA

>4DAPA

MEFSPPLQRATLIQRYKRFLADVITPDGRELTLHCPNTGAMTGCATPGDTVWYSTSDNTKRKYPHTWELTQSQSGAFICVNTLWANRLTKEAILNESISELSGYSSLKSEVKYGAERSRIDFMLQADSRPDCYIEVKSVTLAENEQGYFPDAVTERGQKHLRELMSVAAEGQRAVIFFAVLHSAITRFSPARHIDEKYAQLLSEAQQRGVEILAYKAEISAEGMALKKSLPVTL

>4DFCC

GPHMASALVMKKGQRLSRDALRTQLDSAGYRHVDQVMEHGEYATRGALLDLFPMGSELPYRLDFFDDEIDSLRVFDVDSQRTLEEVEAINLLP

>4DG7H

MGSSHHHHHHSSGLVPRGSHMSNFVNLDIFSNYQKYIDNEQEVRENIRIVVREIEHLSKEAQIKLQIIHSDLSQISAACGLARKQVELCAQKYQKLAELVPAGQYYRYSDHWTFITQRLIFIIALVIYLEAGFLVTRETVAEMLGLKISQSEGFHLDVEDYLLGILQLASELSRFATNSVTMGDYERPLNISHFIGDLNTGFRLLNLKNDGLRKRFDALKYDVKKIEEVVYDVSIRGLSSKEKDQQEEPAVPATE

>4DHXF

MVVSKMNKDAQMRAAINQKLIETGERERLKELLRAKLIECGWKDQLKAHCKEVIKEKGLEHVTVDDLVAEITPKGRALVPDSVKKELLQRIRTFLAQHASL

>4DHXD

GSLVLSELSQGLAVELMERVMMEFVRETCSQELKNAVETDQRVRVARCCEDVCAHLVDLFLVEEIFQTAKETLQE

>4DKYB

MNKAELIDVLTQKLGSDRRQATAAVENVVDTIVRAVHKGDSVTITGFGVFEQRRRAARVARNPRTGETVKVKPTSVPAFRPGAQFKAVVSGAQRLPAEGPHHHHHH

>4DRAE

GSHMEGAGAGSGFRKELVSRLLHLHFKDDKTKVSGDALQLMVELLKVFVVEAAVRGVRQAQAEDALRVDVDQLEKVLPQLLLDF

>4DRBI

GSIFSYRDGMRQSSLKKDWFLSEEEFKLWNRLYRLRDSDEIKEITLPQVQFSSLQNEENKPAQESTTGIHQLSLSEWRLWQDHPLPTHQVDHSDRCRHFIGLMQMIEGMRHEEGECSYELEVESYLQMEDVTSTFIAPRNE

>4DWPA

MGSSHHHHHHSSGLVPRGSHMLAAKRKTKTPVLVERIDQFVGQIKEAMKSDDASRNRKIRDLWDAEVRYHFDNGRTEKTLELYIMKYRNALKAEFGPKSTPLAICNMKKLRERLNTYIARGDYPKTGVATSIVEKIERAEFNTAGRKPTVLLRIADFIAAMNGMDAKQDMQALWDAEIAIMNGRAQTTIISYITKYRNAIREAFGDDHPMLKIATGDAAMYDEARRVKMEKIANKHGALITFENYRQVLKICEDCLKSSDPLMIGIGLIGMTGRRPYEVFTQAEFSPAPYGKGVSKWSILFNGQAKTKQGEGTKFGITYEIPVLTRSETVLAAYKRLRESGQGKLWHGMSIDDFSSETRLLLRDTVFNLFEDVWPKEELPKPYGLRHLYAEVAYHNFAPPHVTKNSYFAAILGHNNNDLETSLSYMTYTLPEDRDNALARLKRTNERTLQQMATIAPVSRKG

>4E1RB

MSYYHHHHHHDYDIPTTENLYFQGAMAKKVTVTLVDDFDGSGAADETVEFGLDGVTYEIDLSTKNATKLRGDLKQWVAAGRRVGGR

>4E2IL

KQVSWKLVTEYAMETKCDDVLLLLGMYLEFQYSFEMCLKCIKKEQPSHYKYHEKHYANAAIFADSKNQKTICQQAVDTVLAKKRVDSLQLTREQMLTNRFNDLLDRMDIMFGSTGSADIEEWMAGVAWLHCLLPKMDSVVYDFLKCMVYNIPKKRYWLFKGPIDSGKTTLAAALLELCGGKALNVNLPLDRLNFELGVAIDQFLVVFEDVKGTGGESRDLPSGQGINNLDNLRDYLDGSVKVNLEKKHLNKRTQIFPPGIVTMNEYSVPKTLQARFVKQIDFRPKDYLKHCLERSEFLLEKRIIQSGIALLLMLIWYRPVAEFAQSIQSRIVEWKERLDKEFSLSVYQKMKFNVAMGIGVLD

>4EOGA

MGMRVLVTTWGNPFQWEPITYEYRGIKVKSRNTLPILVKTLEPERILILVADTMANYYDSGKNKPEIEEKSFSSYSEVVEDTKERILWHIKEEVIEELREEDPELAKKIENMLKDERITIEVLPGVGVFGNITVEGEMLDFYYYATYKLAEWLPVQNNLEVYLDLTHGINFMPTFTYRALRNLLGLLAYLYNVKFEIVNSEPYPLGVSQEIREDTILHIREIGEGVVRPRPQYSPVEGKLYWNAFISSVANGFPLVFASFYPNIRDVEDYLNKKLEEFLVGIEVGEREDGKPYVKREKALDRSFKNASKLYYALRVFNTKFQNYPKKEVPIEEIMEISKIFESLPRIGIILERQVEWLRNLVYGRLWYENGEQKIKKGLLEIIKDKKDKRKEAEALKKGKTISLAEAAKLTRIFSPSGERIETIESPNVVRNFIAHSGFEYNIVYVKYDRLSDRLYFFYKDKEKAANLAYEALLYRGEKE

>4EQ6B

MGSSHHHHHHSHGSMEVLKNIRIYPLSNFITSTKNYINLPNELRNLISEEQESKLGFLHIIESDFKPSVALQKLVNCTTGDEKILIIDIVSIWSQQKQRQHGAIYMNSLSCINITGLIVFLELLYDSPMDALRRCQVDNFNFQLRGIVIDNLSFLNFESDKNYDVINLSKFEKLFKILRKLREFLGCWIITKSFPTDFYNGIENTLVDKWSIKRKSGVTLYPTKLPDSYMKGMDLIIYREVVDGRPQYRRIAALEE

>4EQ6A

MMEYEDLELITIWPSPTKNKLCQFIKQNLSKEHVVTQLFFIDATSSFPLSQFQKLVPPTLPENVRIYENIRINTCLDLEELSAITVKLLQILSMNKINAQRGTEDAVTEPLKIILYINGLEVMFRNSQFKSSPQRSHELLRDTLLKLRVMGNDENENASIRTLLEFPKEQLLDYYLKKNNNTRTSSVRSKRRRIKNGDSLAEYIWKYYADSLFE

>4ER8A

MSEYRRYYIKGGTWFFTVNLRNRRSQLLTTQYQMLRHAIIKVKRDRPFEINAWVVLPEHMHCIWTLPEGDDDFSSRWREIKKQFTHACGLKNIWQPRFWEHAIRNTKDYRHHVDYIYINPVKHGWVKQVSDWPFSTFHRDVARGLYPIDWAGDVTDFSAGERIIS

>4EXWE

GIDPFTMLHIEFITDLGAKVTVDVESADKLLDVQRQYGRLGWTSGEVPVGGYQFPLENEPDFDWSLIGARKWTNPEGEEMILHRGHAYRRRELEAVDSRKMKLPAAVKYSRGAKNTDPEHVREKADGEFEYVTLAIFRGGKRQERYAVPGSNRPQAGAPARSAATRAQGARPGAVAVQDEETPF

>4F6MA

ANKRMKVKHDDHYELIVDGRVYYICIVCKRSYVCLTSLRRHFNIHSWEKKYPCRYCEKVFPLAEYRTKHEIHHTGERRYQCLACGKSFINYQFMSSHIKSVHSQDPSGDSKLYRLHPCRSLQIRQYAYLSDRS

>4FB3E

MHHHHHHSDFPSSLTGYLSHAIYSNKTFPAFLVYSTKEKCKQLYDTIGKFRPEFKCLVHYEEGGMLFFLTMTKHRVSAVKNYCSKLCSVSFLMCKAVTKPMECYQVVTAAPFQLITENKPGLHQFEFTDEPEEQKAVDGSHHHHHH

>4FCYC

GTTTTCGCATTTATCGTGAAACGCTTTCGCGTTTTTCGTGCGCCGCTTCATCTGATGTGTTGTTGACG

>4FCYB

IARPTLEAHDYDREALWSKWDNASDSQRRLAEKWLPAVQAADEMLNQGISTKTAFATVAGHYQVSASTLRDKYYQVQKFAKPDWAAALVDGRGASRRNVHKSEFDEDAWQFLIADYLRPEKPAFRKCYERLELAAREHGWSIPSRATAFRRIQQLDEAMVVACREGEHALMHLIPAQQRTVEHLDAMQWINGDGYLHNVFVRWFNGDVIRPKTWFWQDVKTRKILGWRCDVSENIDSIRLSFMDVVTRYGIPEDFHITIDNTRGAANKWLTGGAPNRYRFKVKEDDPKGLFLLMGAKMHWTSVVAGKGWGQAKPVERAFGVGGLEEYVDKHPALAGAYTGPNPQAKPDNYGDRAVDAELFLKTLAEGVAMFNARTGRETEMCGGKLSFDDVFEREYARTIVRKPTEEQKRMLLLPAEAVNVSRKGEFTLKVGGSLKGAKNVYYNLALMLAGVKKVVVRFDPQQLHSTVYCYTLDGRFICEAECLAPVAFNDAAAGREYRRRQKQLKSATKAAIKAQKQMDALEVAELLP

>4FE7A

MGSSHHHHHHSSGLVPRGSHMFTKRHRITLLFNANKAYDRQVVEGVGEYLQASQSEWDIFIEEDFRARIDKIKDWLGDGVIADFDDKQIEQALADVDVPIVGVGGSYHLAESYPPVHYIATDNYALVESAFLHLKEKGVNRFAFYGLPESSGKRWATEREYAFRQLVAEEKYRGVVYQGLETAPENWQHAQNRLADWLQTLPPQTGIIAVTDARARHILQVCEHLHIPVPEKLCVIGIDNEELTRYLSRVALSSVAQGARQMGYQAAKLLHRLLDKEEMPLQRILVPPVRVIERRSTDYRSLTDPAVIQAMHYIRNHACKGIKVDQVLDAVGISRSNLEKRFKEEVGETIHAMIHAEKLEKARSLLISTTLSINEISQMCGYPSLQYFYSVFKKAYDTTPKEYRDVNSEVML

>4FJOC

MTTLTRQDLNFGQVVADVLSEFLEVAVHLILYVREVYPVGIFQKRKKYNVPVQMSCHPELNQYIQDTLHCVKPLLEKNDVEKVVVVILDKEHRPVEKFVFEITQPPLLSINSDSLLSHVEQLLAAFILKISVCDAVLDHNPPGCTFTVLVHTREAATRNMEKIQVIKDFPWILADEQDVHMHDPRLIPLKTMTSDILKMQLYVEERAHKN

>4FJOA

AAPNLAGAVEFSDVKTLLKEWITTISDPMEEDILQVVRYCTDLIEEKDLEKLDLVIKYMKRLMQQSVESVWNMAFDFILDNVQVVLQQTYGSTLKVT

>4FW2B

PLREAKDLHTALHIGPRALSKASNISMQQAREVVQTCPHCNSAPALEAGVNPRGLGPLQIWQTDFTLEPRMAPRSWLAVTVDTASSAIVVTQHGRVTSVAVQHHWATAIAVLGRPKAIKTDNGSCFTSKSTREWLARWGIAHTTGIPGNSQGQAMVERANRLLKDKIRVLAEGDGFMKRIPTSKQGELLAKAMYALNHKERGENTKTPIQKHWRPTVLTEGPPVKIRIETGEWEKGWNVLVWGRGYAAVKNRDTDKVIWVPSRKVKPDIT

>4G12B

MTASAPDGRPGQPEATNRRSQLKSDRRFQLLAAAERLFAERGFLAVRLEDIGAAAGVSGPAIYRHFPNKESLLVELLVGVSARLLAGARDVTTRSANLAAALDGLIEFHLDFALGEADLIRIQDRDLAHLPAVAERQVRKAQRQYVEVWVGVLRELNPGLAEADARLMAHAVFGLLNSTPHSMKAADSKPARTVRARAVLRAMTVAALSAADRCL

>4G4KB

MDNSVETIELKRGSNSVYVQYDDIMFFESSTKSHRLIAHLDNRQIEFYGNLKELSQLDDRFFRCHNSFVVNRHNIESIDSKERIVYFKNKEHCYASVRNVKKI

>4G6DB

MKLKILDKDNATLNVFHRNKEHKTIDNVPTANLVDWYPLSNAYEYKLSRNGEYLELKRLRSTLPSSYGLDDNNQDIIRDNNHRCKIGYWYNPAVRKDNLKIIEKAKQYGLPIITEEYDANTVEQGFRDIGVIFQSLKTIVVTRYLEGKTEEELRIFNMKSEESQLNEALKESDFSVDLTYSDLGQIYNMLLLMKKISK

>4G6DA

MKEQLEDVLDTLTDREENVLRLRFGLDDGRTRTLEEVGKVFGVTRERIRQIEAKALRKLRHPSRSKRLKDFMD

>4GNXL

TTTTTTTTTTTTTTTTTTTTTTTTTTTTTTTTTTTTTTTTTTTTTTTTTTTTTTTTTTTTTT

>4GNXK

TTTTTTTTTTTTTTTTTTTTTTTTTTTTTTTTTTTTTTTTTTTTTTTTTTTTTTTTTTTTTT

>4GNXZ

MPIYPIEGLSPYQNRWTIKARVTSKSDIRHWSNQRGEGKLFSVNLLDDSGEIKATGFNDAVDRFYPLLQENHVYLISKARVNIAKKQFSNLQNEYEITFENSTEIEECTDATDVPEVKYEFVRINELESVEANQQCDVIGILDSYGELSEIVSKASQRPVQKRELTLVDQGNRSVKLTLWGKTAETFPTNAGVDEKPVLAFKGVKVGDFGGRSLSMFSSSTMLINPDITESHVLRGWYDNDGAHAQFQPYTNGGVGGGAMGGGGAGANMAERRTIVQVKDENLGMSEKPDYFNVRATVVYIKQENLYYTACASEGCNKKVNLDHENNWRCEKCDRSYATPEYRYILSTNVADATGQMWLSGFNEDATQLIGMSAGELHKLREESESEFSAALHRAANRMYMFNCRAKMDTFNDTARVRYTISRAAPVDFAKAGMELVDAIRAYM

>4GS3A

SNAMAGNFLENNTVTLVGKVFTPLEFSHELYGEKFFNFILEVPRLSETKDYLPITISNRLFEGMNLEVGTRVKIEGQLRSYNRKSPEEGKNKLILTVFARDISVVPE

>4H79A

GSMTTTETPKTISLTWVGTFVDQRVREIQEGYRLDNPRAVATLARLRRGAGKEIGDTPDLWGLILDDRFYADAPPLKEKDMEVAENSAHIALTLYAIHQQSRRDDRMHQRGWGLGEAVRRLMPSSEIDEPLRKRFVQVGHAVTYKALAQRLREIVTLLRRDAIPLDYGLLADQLYQFRTPQGAQRVRTAWGRGFHAYRPKTTQNPDSTTTTEKDNS

>4H7AB

MSRGHHHHHHGSMSPGERFLDWLKRLQGQKAWTAARAAFRRSLAFPPGAYPRAMPYVEPFLAKGDWRQEEREAHYLVAALYALKDGDHQVGRTLARALWEKAQGSASVEKRFLALLEADRDQIAFRLRQAVALVEGGIDFARLLDDLLRWFSPERHVQARWAREYYGA

>4H9SE

GSRRQIQRLEQLLALYVAEIRRLQEKELDLSELDDPDSAYLQEARLKRKLIRLFGRLCELKDCSSLTGRVIEQRIPYRGTRYPEVNRRIERLINKPGPDTFPDYGDVLRAVEKAAARHSLGLPRQQLQLMAQDAFRDVGIRLQERRHLDLIYNFGCHLTDDYRPGVDPALSDPVLARRLRENRSLAMSRLDEVISKYAMLQDKSEEGERKKRRARL

>4HD0B

HHHHHHMKFAHLADIHLGYEQFHKPQREEEFAEAFKNALEIAVQENVDFILIAGDLFHSSRPSPGTLKKAIALLQIPKEHSIPVFAIEGNHDRTQRGPSVLNLLEDFGLVYVIGMRKEKVENEYLTSERLGNGEYLVKGVYKDLEIHGMKYMSSAWFEANKEILKRLFRPTDNAILMLHQGVREVSEARGEDYFEIGLGDLPEGYLYYARGHIHKRYETSYSGSPVVYPGSLERWDFGDYEVRYEWDGIKFKERYGVNKGFYIVEDFKPRFVEIKVRPFIDVKIKGSEEEIRKAIKRLIPLIPKNAYVRLNIGWRKPFDLTEIKELLNVEYLKIDTWRI

>4HIDA

MSDSFSLLSQITPHQRCSFYAQVIKTWYSDKNFTLYVTDYTENELFFPMSPYTSSSRWRGPFGRFSIRCILWDEHDFYCRNYIKEGDYVVMKNVRTKIDHLGYLECILHGDSAKRYNMSIEKVDSEEPELNEIKSRKRLYVQN

>4HLXD

MHHHHHHSSGVDLGTENLYFQSMGKASIKDWIVCQVNSGKFPGVEWEDEERTRFRIPVTPLADPCFEWRRDGELGVVYIRERGNMPVDASFKGTRGRRRMLAALRRTRGLQEIGKGISQDGHHFLVFRVRKP

>4HOBA

GSHMASNPISEEMNLKILAYLGTKQGAKAVHIAQSLGAQRSEVNRHLYRMSEDGRVRKHPQHPVWYLPA

>4HP1C

MHHHHHHSSGRENLYFQGSNKKRKRCGVCVPCLRKEPCGACYNCVNRSTSHQICKMRKCEQLKKKRVVPMKG

>4HTOA

MAASQTSQTVASHVPFADLCSTLERIQKSKGRAEKIRHFREFLDSWRKFHDALHKNHKDVTDSFYPAMRLILPQLERERMAYGIKETMLAKLYIELLNLPRDGKDALKLLNYRTPTGTHGDAGDFAMIAYFVLKPRCLQKGSLTIQQVNDLLDSIASNNSAKRKDLIKKSLLQLITQSSALEQKWLIRMIIKDLKLGVSQQTIFSVFHNDAAELHNVTTDLEKVCRQLHDPSVGLSDISI

>4HW0C

MQLERRKRGTMEIMFDILRNCEPKCGITRVIYGAGINYVVAQKYLDQLVKVGALNIKTENDRKIYEITEKGKLLRTHIEEFIKIRENLYSAKEKVSELLRTDSE

>4I1KB

MRGSHHHHHHGSRSKFYESASARKRTVTAEERERAINAAKTFEPTNPFFRVVLRPSYLYRGCIMYLPSGFAEKYLSGISGFIKVQLAEKQWPVRCLYKAGRAKFSQGWYEFTLENNLGEGDVCVFELLRTRDFVLKVTAFRVNEYV

>4I99D

KKVEIDEEIFVIDDFRVDIEKYVEELYKVVKKIYEKTGTPIKFWDLVPDVEPKIIARTFLYLLFLENMGRVEIIQEEPFGEILVVPM

>4I99B

MPYIEKLELKGFKSYGNKKVVIPFSKGFTAIVGANGSGKSNIGDAILFVLGGLSAKAMRASRISDLIFAGSKNEPPAKYAEVAIYFNNEDRGFPIDEDEVVIRRRVYPDGRSSYWLNGRRATRSEILDILTAAMISPDGYNIVLQGDITKFIKMSPLERRLLIDDISGIAEYDSKKEKALEEEKEKKNVFMRTFEAISRNFSEIFAKLSPGGSARLILENPEDPFSGGLEIEAKPAGKDVKRIEAMSGGEKALTALAFVFAIQKFKPAPFYLFDEIDAHLDDANVKRVADLIKESSKESQFIVITLRDVMMANADKIIGVSMRDGVSKVVSLSLEKAMKILEEIRKKQGWEHGN

>4ICGD

GSHMSDKPLTKTDYLMRLRRCQTIDTLERVIEKNKYELSDNELAVFYSAADHRLAELTMNKLYDKIPSSVWKFIR

>4IDUB

MSSQFIFEDVPQRNAATFNPEVGYVAFIGKYGQQLNFGVARVFFLNQKKAKMVLHKTAQPSVDLTFGGVKFTVVNNHFPQYVSNPVPDNAITLHRMSGYLARWIADTCKASVLKLAEASAQIVMPLAEVKGCTWADGYTMYLGFAPGAEMFLDAFDFYPLVIEMHRVLKDNMDVNFMKKVLRQRYGTMTAEEWMTQKITEIKAAFNSVGQLAWAKSGFSPAARTFLQQFGINI

>4IJHA

GSHMVGQLSRGAIAAIMQKGDTNIKPILQVINIRPITTGNSPPRYRLLMSDGLNTLSSFMLATQLNPLVEEEQLSSNCVCQIHRFIVNTLKDGRRVVILMELEVLKSAEAVGVKIGNPVPYNE

>4IRHA

GAMVPKTEDQRPQLDPYQILGPTSSRLANPGSGQIQLWQFLLELLSDSSNSSCITWEGTNGEFKMTDPDEVARRWGERKSKPNMNYDKLSRALRYYYDKNIMTKVHGKRYAYKFDFHGIAQALQPHPPE

>4IX7B

DNVMVSIGPNNTCVPASVFENINWSVCSLATRKLLVTIFDRETLATHSVTGKPSPAFKDQDKPLKRMLDPGKIQDIIFAVTHKCNASEKEVRNAITTKCADENKMMKIQNVKRRS

>4JJNJ

ATCGGATGTATATATCTGACACGTGCCTGGAGACTAGGGAGTAATCCCCTTGGCGGTTAAAACGCGGGGGACAGCGCGTACGTGCGTTTAAGCGGTGCTAGAGCTGTCTACGACCAATTGAGCGGCCTCGGCACCGGGATTCTCGAT

>4JJNI

ATCGAGAATCCCGGTGCCGAGGCCGCTCAATTGGTCGTAGACAGCTCTAGCACCGCTTAAACGCACGTACGCGCTGTCCCCCGCGTTTTAACCGCCAAGGGGATTACTCCCTAGTCTCCAGGCACGTGTCAGATATATACATCCGAT

>4JJNL

SAKTLKDLDGWQVIITDDQGRVIDDNNRRRSRKRGGENVFLKRISDGLSFGKGESVIFNDNVTETYSVYLIHEIRLNTLNNVVEIWVFSYLRWFELKPKLYYEQFRPDLIKEDHPLEFYKDKFFNEVNKSELYLTAELSEIWLKDFIAVGQILPESQWNDSSIDKIEDRDFLVRYACEPTAEKFVPIDIFQIIRRVKEMEPKQSNEYLKRVSVPVSGQKTNRQVMHKMGVERSSKRLAKKPSMKKIKIEPSADDDVNNGNIPSQRGTSTTHGSISPQEESVSPNISSASPSALTSPTDSSKILQKRSISKELIVSEEIPINSSEQESDYEPNNETSVLSSKPGSKPEKTSTELVDGRENFVYANNPEVSDDGGLEEETDEVS

>4JJNH

SSAAEKKPASKAPAEKKPAAKKTSTSVDGKKRSKVRKETYSSYIYKVLKQTHPDTGISQKSMSILNSFVNDIFERIATEASKLAAYNKKSTISAREIQTAVRLILPGELAKHAVSEGTRAVTKYSSSTQA

>4JJNG

SGGKGGKAGSAAKASQSRSAKAGLTFPVGRVHRLLRRGNYAQRIGSGAPVYLTAVLEYLAAEILELAGNAARDNKKTRIIPRHLQLAIRNDDELNKLLGNVTIAQGGVLPNIHQNLLPKKSAKTAKASQEL

>4JLXA

GAMGAWKLQTVLEKVRLSRHEISEAAEVVNWVVEHLLRRLQGGESEFKGVALLRTGSYYERVKISAPNEFDVMFKLEVPRIQLEEYCNSGAHYFVKFKRNPGGNPLEQFLEKEILSASKMLSKFRKIIKEEIKNIEDTGVTVERKRRGSPAVTLLISKPKEISVDIILALESKSSWPASTQKGLPISQWLGAKVKNNLKRQPFYLVPKHAKEGSGFQEETWRLSFSHIEKDILKNHGQSKTCCEIDGVKCCRKECLKLMKYLLEQLKKKFGNRRELAKFCSYHVKTAFFHVCTQDPHDNQWHLKNLECCFDNCVAYFLQCLKTEQLANYFIPGVNLFSRDLIDKPSKEFLSKQIEYERNNGFPVFW

>4JOIC

MLPKPGTYYLPWEVSAGQVPDGSTLRTFGRLCLYDMIQSRVTLMAQHGSDQHQVLVCTKLVEPFHAQVGSLYIVLGELQHQQDRGSVVKARVLTCVEGMNLPLLEQAIREQRLYKQERGGSQ

>4JOIB

LDPVFLAFAKLYIRDILDMKESRQVPGVFLYNGHPIKQVDVLGTVIGVRERDAFYSYGVDDSTGVINCICWKKLNTESVSAAPSAARELSLTSQLKKLQETIEQKTKIEIGDTIRVRGSIRTYREEREIHATTYYKVDDPVWNIQIARMLELPTIYRKVYDQPFHS

>4JOLD

SEEMIDHRLTDREWAEEWKHLDHLLNCIMDMVEKTRRSLTVLRRCQEADREELNYWIRRYSDAE

>4JQFA

AEALSNPGALDLPSLTSLLSEKAKEFLMENRVQSFYQQELEMVESLLSLANQPVIHSASSDQVNFKKDTTSKAIHSIFKNAIQLLQEKGLVFQKDDGFDNLYYVTREDKDLHRKIHRIIQQDCQKPNHMEKGCHFLHILACARLSIRPGLSEAVLQQVLELLEDQSDIVSTMEHYYTAF

>4JW3D

MRGSHHHHHHTDPEKVEMYIKNLQDDSYFVRRAAAAALGKIGDERAVEPLIKALKDEDRFVRSSAAYALGEIGDERAVEPLIKALKDEDWFVRRAAAVALGEIGGERVRAAMEKLAETGTGFARKVAVNYLETHKSLIS

>4JW3B

AAPTATVTPSSGLSDGTVVKVAGAGLQAGTAYWVAQWARVDTGVWAYNPADNSSVTADANGSASTSLTVRRSFEGFLFDGTRWGTVDCTTAACQVGLSDAAGNGPEGVAISFAAHHHHHH

>4K2JA

SHPRYQQPPVPYRQIDDCPAKARPQHIFYRRFLGKDGRRDPKCQWKFAVIFWGNDPYGLKKLSQAFQFGGVKAGPVSCLPHPGPDQSPITYCVYVYCQNKDTSKKVQMARLAWEASHPLAGNLQSSIVKFKKPLPLTQPG

>4K74B

HHHHHHMKFTVEREHLLKPLQQVSGPLGGRPTLPILGNLLLQVADGTLSLTGTDLEMEMVARVALVQPHEPGATTVPARKFFDICRGLPEGAEIAVQLEGERMLVRSGRSRFSLSTLPAADFPNLDDWQSEVEFTLPQATMKRLIEATQFSMAHQDVRYYLNGMLFETEGEELRTVATDGHRLAVCSMPIGQSLPSHSVIVPRKGVIELMRMLDGGDNPLRVQIGSNNIRAHVGDFIFTSKLVDGRFPDYRRVLPKNPDKHLEAGCDLLKQAFARAAILSNEKFRGVRLYVSENQLKITANNPEQEEAEEILDVTYSGAEMEIGFNVSYVLDVLNALKCENVRMMLTDSVSSVQIEDAASQSAAYVVMPMRL

>4KPYA

MNHLGKTEVFLNRFALRPLNPEELRPWRLEVVLDPPPGREEVYPLLAQVARRAGGVTVRMGDGLASWSPPEVLVLEGTLARMGQTYAYRLYPKGRRPLDPKDPGERSVLSALARRLLQERLRRLEGVWVEGLAVYRREHARGPGWRVLGGAVLDLWVSDSGAFLLEVDPAYRILCEMSLEAWLAQGHPLPKRVRNAYDRRTWELLRLGEEDPKELPLPGGLSLLDYHASKGRLQGREGGRVAWVADPKDPRKPIPHLTGLLVPVLTLEDLHEEEGSLALSLPWEERRRRTREIASWIGRRLGLGTPEAVRAQAYRLSIPKLMGRRAVSKPADALRVGFYRAQETALALLRLDGAQGWPEFLRRALLRAFGASGASLRLHTLHAHPSQGLAFREALRKAKEEGVQAVLVLTPPMAWEDRNRLKALLLREGLPSQILNVPLREEERHRWENALLGLLAKAGLQVVALSGAYPAELAVGFDAGGRESFRFGGAACAVGGDGGHLLWTLPEAQAGERIPQEVVWDLLEETLWAFRRKAGRLPSRVLLLRDGRVPQDEFALALEALAREGIAYDLVSVRKSGGGRVYPVQGRLADGLYVPLEDKTFLLLTVHRDFRGTPRPLKLVHEAGDTPLEALAHQIFHLTRLYPASGFAFPRLPAPLHLADRLVKEVGRLGIRHLKEVDREKLFFV

>4LDUA

MMASLSCVEDKMKTSCLVNGGGTITTTTSQSTLLEEMKLLKDQSGTRKPVINSELWHACAGPLVCLPQVGSLVYYFSQGHSEQVAVSTRRSATTQVPNYPNLPSQLMCQVHNVTLHADKDSDEIYAQMSLQPVHSERDVFPVPDFGMLRGSKHPTEFFCKTLTASDTSTHGGFSVPRRAAEKLFPPLDYSAQPPTQELVVRDLHENTWTFRHIYRGQPKRHLLTTGWSLFVGSKRLRAGDSVLFIRDEKSQLMVGVRRANRQQTALPSSVLSADSMHIGVLAAAAHATANRTPFLIFYNPRACPAEFVIPLAKYRKAICGSQLSVGMRFGMMFETEDSGKRRYMGTIVGISDLDPLRWPGSKWRNLQVEWDEPGCNDKPTRVSPWDIETPNSYSQSM

>4LG8A

MHHHHHHSSGRENLYFQGTPEIIQKLQDKATVLTTERKERGKTVPEELVKPEELSKYRQVASHVGLHSASIPGILALDLCPSDTNKILTGGADKNVVVFDKSSEQILATLKGHTKKVTSVVFHPSQDLVFSASPDATIRIWSVPNASCVQVVRAHESAVTGLSLHATGDYLLSSSDDQYWAFSDIQTGRVLTKVTDETSGCSLTCAQFHPDGLIFGTGTMDSQIKIWDLKERTNVANFPGHSGPITSIAFSENGYYLATAADDSSVKLWDLRKLKNFKTLQLDNNFEVKSLIFDQSGTYLALGGTDVQIYICKQWTEILHFTEHSGLTTGVAFGHHAKFIASTGMDRSLKFYSL

>4LJKA

MKSHFQYSTLENIPKAFDILKDPPKKLYCVGDTKLLDTPLKVAIIGTRRPTPYSKQHTITLARELAKNGAVIVSGGALGVDIIAQENALPKTIMLSPCSLDFIYPTNNHKVIQEIAQNGLILSEYEKDFMPIKGSFLARNRLVIALSDVVIIPQADLKSGSMSSARLAQKYQKPLFVLPQRLNESDGTNELLEKGQAQGIFNIQNFINTLLKDYHLKEMPELEHHHHHH

>4LRVL

SMLPNRMALSRQTEDQLKKLKGYTGITPNIAARLAFFRSVESEFRYSPERDSKKLDGTLVLDKITWLGETLQATELVLKMLYPQLEQKALIKAWAAHVEDGIAALRNHK

>4M6WB

MGHIVANEKWRGSQLAQEMQGKIKLIFEDGLTPDFYLSNRCCILYVTEADLVAGNGYRKRLVRVRNSNNLKGIVVVEKTRMSEQYFPALQKFTVLDLGMVLLPVASQMEASCLVIQLVQEQTKEPSKNPLLGKKRALLLSEPSLLRTVQQIPGVGKVKAPLLLQKFPSIQQLSNASIGELEQVVGQAVAQQIHAFFTQPRLEHHHHHH

>4M6WA

MGQEGKGTCILVGGHEITSGLEVISSLRAIHGLQVEVCPLNGCDYIVSNRMVVERRSQSEMLNSVNKNKFIEQIQHLQSMFERICVIVEKDREKTGDTSRMFRRTKSYDSLLTTLIGAGIRILFSSCQEETADLLKELSLVEQRKNVGIHVPTVVNSNKSEALQFYLSIPNISYITALNMCHQFSSVKRMANSSLQEISMYAQVTHQKAEEIYRYIHYVFD

>4MZ9D

MASRGVNKVILVGNLGQDPEVRYMPNGGAVANITLATSESWRDKATGEMKEQTEWHRVVLFGKLAEVASEYLRKGSQVYIEGQLRTRKWTDQSGQDRYTTEVVVNVGGTMQMLGGRQGGGAPAGGNIGGGQPQGGWGQPQQPQGGNQFSGGAQSRPQQSAPAAPSNEPPMDFDDDIPF

>4N0UE

GGPSVFLFPPKPKDTLYITREPEVTCVVVDVSHEDPEVKFNWYVDGVEVHNAKTKPREEQYNSTYRVVSVLTVLHQDWLNGKEYKCKVSNKALPAPIEKTISKAKGQPREPQVYTLPPSRDELTKNQVSLTCLVKGFYPSDIAVEWESNGQPENNYKTTPPVLDSDGSFFLYSKLTVDKSRWQQGNVFSCSVMHEALHNHYTQKSLSLS

>4N0UD

HKSEVAHRFKDLGEENFKALVLIAFAQYLQQCPFEDHVKLVNEVTEFAKTCVADESAENCDKSLHTLFGDKLCTVATLRETYGEMADCCAKQEPERNECFLQHKDDNPNLPRLVRPEVDVMCTAFHDNEETFLKKYLYEIARRHPYFYAPELLFFAKRYKAAFTECCQAADKAACLLPKLDELRDEGKASSAKQRLKCASLQKFGERAFKAWAVARLSQRFPKAEFAEVSKLVTDLTKVHTECCHGDLLECADDRADLAKYICENQDSISSKLKECCEKPLLEKSHCIAEVENDEMPADLPSLAADFVESKDVCKNYAEAKDVFLGMFLYEYARRHPDYSVVLLLRLAKTYETTLEKCCAAADPHECYAKVFDEFKPLVEEPQNLIKQNCELFEQLGEYKFQNALLVRYTKKVPQVSTPTLVEVSRNLGKVGSKCCKHPEAKRMPCAEDYLSVVLNQLCVLHEKTPVSDRVTKCCTESLVNRRPCFSALEVDETYVPKEFNAETFTFHADICTLSEKERQIKKQTALVELVKHKPKATKEQLKAVMDDFAAFVEKCCKADDKETCFAEEGKKLVAASQAALGL

>4N0UB

IQRTPKIQVYSRHPAENGKSNFLNCYVSGFHPSDIEVDLLKNGERIEKVEHSDLSFSKDWSFYLLYYTEFTPTEKDEYACRVNHVTLSQPKIVKWDRDM

>4N0UA

HLSLLYHLTAVSSPAPGTPAFWVSGWLGPQQYLSYNSLRGEAEPCGAWVWENQVSWYWEKETTDLRIKEKLFLEAFKALGGKGPYTLQGLLGCELGPDNTSVPTAKFALNGEEFMNFDLKQGTWGGDWPEALAISQRWQQQDKAANKELTFLLFSCPHRLREHLERGRGNLEWKEPPSMRLKARPSSPGFSVLTCSAFSFYPPELQLRFLRNGLAAGTGQGDFGPNSDGSFHASSSLTVKSGDEHHYCCIVQHAGLAQPLRVEL

>4N6QA

MSAANYPDPSLPRPSTSDDFELIVRQNPNRARVAGGKEKERKPVDPPPIVQIRVREEGTYLAQHYLQSPYFFMSCSLYDAQEDAPASIPPSTALTGTLVSSLHRLKDVDNTDGGFFVWGDLSIKVEGDFRLKFSLFEMRKTDVVFLKSIVSERFTVSPPKSFPGMAESTFLSRSFADQGVKLRIRKEPRTSAWSHPQFEK

>4N6RB

MYAVEDRAHSGHHPPPLSMDRIPPPSTMYPSSAGPSAMVSPAGQPEPESLSTVHDGRIWSLQVVQQPIRARMCGFGDKDRRPITPPPCIRLIVKDAQTQKEVDINSLDSSFYVVMADLWNADGTHEVNLVKHSATSPSISTAMSSSYPPPPHPTSSDYPASYQTNPYGQPVGQPVGQPVGYAGVGNYYGGSTQLQYQNAYPNPQAQYYQPMYGGMAQPQMPAAQPVTPGPGGMFTRNLIGCLSASAYRLYDTEDKIGVWFVLQDLSVRTEGIFRLKFSFVNVGKSVSDLPQSDIAEVINKGTAPILASTFSEPFQVFSAKKFPGVIESTPLSKVFANQGIKIPIRKDGVKGQGSRGRHSDEDDGLDNEYSAHHHHHH

>4NDFA

GSHMGHWSQGLKISMQDPKMQVYKDEQVVVIKDKYPKARYHWLVLPWTSISSLKAVAREHLELLKHMHTVGEKVIVDFAGSSKLRFRLGYHAIPSMSHVHLHVISQDFDSPCLKNKKHWNSFNTEYFLESQAVIEMVQEAGRVTVRDGMPELLKLPLRCHECQQLLPSIPQLKEHLRKHWTQ

>4NJXD

MSHHHHHHSMAAAVVLAAGLRAARRAVAATGVRGGQVRGAAGVTDGNEVAKAQQATPGGAAPTIFSRILDKSLPADILYEDQQCLVFRDVAPQAPVHFLVIPKKPIPRISQAEEEDQQLLGHLLLVAKQTAKAEGLGDGYRLVINDGKLGAQSVYHLHIHVLGGRQLQWPPG

>4NL4H

HHHHHHSSGLVPRGSHMSVAHVALPVPLPRTFDYLLPEGMAVKAGCRVRVPFGKQERIGIVAAVSERSELPLDELKPVAEALDDEPVFSTTVWRLLMWAAEYYHHPIGDVLFHALPVMLRQGKPASATPLWYWFATEQGQVVDLNGLKRSRKQQQALAALRQGKIWRHQVGELEFNEAALQALRGKGLAELACEAPALTDWRSAYSVAGERLRLNTEQATAVGAIHSAADRFSAWLLAGITGSGKTEVYLSVLENVLAQGRQALVMVPEIGLTPQTIARFRQRFNAPVEVLHSGLNDSERLSAWLKAKNGEAAIVIGTRSSLFTPFKDLGVIVIDEEHDSSYKQQEGWRYHARDLAVWRAHSEQIPIILGSATPALETLHNVRQGKYRQLTLSKRAGNARPAQQHVLDLKGQPLQAGLSPALISRMRQHLQADNQVILFLNRRGFAPALLCHDCGWIAECPRCDSYYTLHQAQHHLRCHHCDSQRPIPRQCPSCGSTHLVPVGIGTEQLEQALAPLFPEVPISRIDRDTTSRKGALEEHLAAVHRGGARILIGTQMLAKGHHFPDVTLVSLLDVDGALFSADFRSAERFAQLYTQVSGRAGRAGKQGEVILQTHHPEHPLLQTLLYKGYDAFAEQALAERQTMQLPPWTSHVLIRAEDHNNQQAPLFLQQLRNLLQASPLADEKLWVLGPVPALAPKRGGRWRWQILLQHPSRVRLQHIVSGTLALINTLPEARKVKWVLDVDPIEG

>4NQWB

MTEHTDFELLELATPYALNAVSDDERADIDRRVAAAPSPVAAAFNDEVRAVRETMAVVSAATTAEPPAHLRTAILDATKP

(2).550 non DNA-binding proteins

>1RQWA

ATFEIVNRCSYTVWAAASKGDAALDAGGRQLNSGESWTINVEPGTKGGKIWARTDCYFDDSGSGICKTGDCGGLLRCKRFGRPPTTLAEFSLNQYGKDYIDISNIKGFNVPMDFSPTTRGCRGVRCAADIVGQCPAKLKAPGGGCNDACTVFQTSEYCCTTGKCGPTEYSRFFKRLCPDAFSYVLDKPTTVTCPGSSNYRVTFCPTA

>1H2GB

SNMWVIGKSKAQDAKAIMVNGPQFGWYAPAYTYGIGLHGAGYDVTGNTPFAYPGLVFGHNGVISWGSTAGLGDDVDIFAERLSAEKPGYYLHNGKWVKMLSREETITVKNGQAETFTVWRTVHGNILQTDQTTQTAYAKSRAWDGKEVASLLAWTHQMKAKNWQEWTQQAAKQALTINWYYADVNGNIGYVHTGAYPDRQSGHDPRLPVPGTGKWDWKGLLPFEMNPKVYNPQSGYIANWNNSPQKDYPASDLFAFLWGGADRVTEIDRLLEQKPRLTADQAWDVIRQTSRQDLNLRLFLPTLQAATSGLTQSDPRRQLVETLTRWDGINLLNDDGKTWQQPGSAILNVWLTSMLKRTVVAAVPMPFDKWYSASGYETTQDGPTGSLNISVGAKILYEAVQGDKSPIPQAVDLFAGKPQQEVVLAALEDTWETLSKRYGNNVSNWKTPAMALTFRANNFFGVPQAAAEETRHQAEYQNRGTENDMIVFSPTTSDRPVLAWDVVAPGQSGFIAPDGTVDKHYEDQLKMYENFGRKSLWLTKQDVEAHKESQEVLHVQR

>1A12A

RRSPPADAIPKSKKVKVSHRSHSTEPGLVLTLGQGDVGQLGLGENVMERKKPALVSIPEDVVQAEAGGMHTVCLSKSGQVYSFGCNDEGALGRDTSVEGSEMVPGKVELQEKVVQVSAGDSHTAALTDDGRVFLWGSFRDNNGVIGLLEPMKKSMVPVQVQLDVPVVKVASGNDHLVMLTADGDLYTLGCGEQGQLGRVPELFANRGGRQGLERLLVPKCVMLKSRGSRGHVRFQDAFCGAYFTFAISHEGHVYGFGLSNYHQLGTPGTESCFIPQNLTSFKNSTKSWVGFSGGQHHTVCMDSEGKAYSLGRAEYGRLGLGEGAEEKSIPTLISRLPAVSSVACGASVGYAVTKDGRVFAWGMGTNYQLGTGQDEDAWSPVEMMGKQLENRVVLSVSSGGQHTVLLVKDKEQS

>3INGA

GMKEIRIILMGTGNVGLNVLRIIDASNRRRSAFSIKVVGVSDSRSYASGRNLDISSIISNKEKTGRISDRAFSGPEDLMGEAADLLVDCTPASRDGVREYSLYRMAFESGMNVVTANKSGLANKWHDIMDSANQNSKYIRYEATVAGGVPLFSVLDYSILPSKVKRFRGIVSSTINYVIRNMANGRSLRDVVDDAIKKGIAESNPQDDLNGLDAARKSVILVNHIFGTEYTLNDVEYSGVDERSYNANDRLVTEVYVDDRRPVAVSRIISLNKDDFLMSIGMDGLGYQIETDSNGTVNVSDIYDGPYETAGAVVNDILLLSKVQK

>2RAUA

GMYEEWKIVKREAPILGNDQLIENIWKMKREDSPYDIISLHKVNLIGGGNDAVLILPGTWSSGEQL23VTISWNGVHYTIPDYRKSIVLYLARNGFNVYTIDYRTHYVPPFLKDRQLSFTANWGWSTWISDIKEVVSFIKRDSGQERIYLAGESFGGIAALNYSSLYWKNDIKGLILLDGGPTKHGIRPKFYTPEVNSIEEMEAKGIYVIPSRGGPNNPIWSYALANPDMPSPDPKYKSISDFLMDSLYVTGSANPYDYPYSKKEDMFPILASFDPYWPYRLSLERDLKFDYEGILVPTIAFVSERFGIQIFDSKILPSNSEIILLKGYGHLDVYTGENSEKDVNSVVLKWLSQQR

>1UAIA

AEPCDYPAQQLDLTDWKVTLPIGSSGKPSEIEQPALDTFATAPWFQVNAKCTGVQFRAAVNGVTTSGSGYPRSELREMTDGGEEKASWSATSGTHTMVFREAFNHLPEVKPHLVGAQIHDGDDDVTVFRLEGTSLYITKGDDTHHKLVTSDYKLNTVFEGKFVVSGGKIKVYYNGVLQTTISHTSSGNYFKAGAYTQANCSNSSPCSSSNYGQVSLYKLQVTHS

>3H5QA

SNAMRMIDIIEKKRDGHTLTTEEINFFIGGYVKGDIPDYQASSLAMAIYFQDMNDDERVALTMAMVNSGDMIDLSDIKGVKVDKHSTGGVGDTTTLVLAPLVAAVDVPVAKMSGRGLGHTGGTIDKLEAIDGFHVEIDEATFVKLVNENKVAVVGQSGNLTPADKKLYALRDVTGTVNSIPLIASSIMSKKIAAGADAIVLDVKTGSGAFMKTLEDAEALAHAMVRIGNNVGRNTMAIISDMNQPLGRAIGNALELQEAIDTLKGQGPKDLTELVLTLGSQMVVLANKAETLEEARALLIEAINSGAALEKFKTFIKNQGGDETVIDHPERLPQAQYQIEYKAKKSGYVTELVSNDIGVASMMLGAGRLTKEDDIDLAVGIVLNKKIGDKVEEGESLLTIHSNRQDVDDVVKKLDSSITIADHVVSPTLIHKIITE

>3N20A

MSMLKREDWYDLTRTTNWTPKYVTENELFPEEMSGARGISMEAWEKYDEPYKITYPEYVSIQREKDSGAYSIKAALERDGFVDRADPGWVSTMQLHFGAIALEEYAASTAEARMARFAKAPGNRNMATFGMMDENRHGQIQLYFPYANVKRSRKWDWAHKAIHTNEWAAIAARSFFDDMMMTRDSVAVSIMLTFAFETGFVNMQFLGLAADAAEAGDHTFASLISSIQTDESRHAQQGGPSLKILVENGKKDEAQQMVDVAIWRSWKLFSVLTGPIMDYYTPLESRNQSFKEFMLEWIVAQFERQLLDLGLDKPWYWDQFMQDLDETHHGMHLGVWYWRPTVWWDPAAGVSPEEREWLEEKYPGWNDTWGQCWDVITDNLVNGKPELTVPETLPTICNMCNLPIAHTPGNKWNVKDYQLEYEGRLYHFGSEADRWCFQIDPERYENHTNLVDRFLKGEIQPADLAGALMYMSLEPGVMGDDAHDYEWVKAYQKKTNAA

>3N2BA

MHHHHHHSSGVDLGTENLYFQSNAMDYFNYQEDGQLWAEQVPLADLANQYGTPLYVYSRATLERHWHAFDKSVGDYPHLICYAVKANSNLGVLNTLARLGSGFDIVSVGELERVLAAGGDPSKVVFSGVGKTEAEMKRALQLKIKCFNVESEPELQRLNKVAGELGVKAPISLRINPDVDAKTHPYISTGLRDNKFGITFDRAAQVYRLAHSLPNLDVHGIDCHIGSQLTALAPFIDATDRLLALIDSLKAEGIHIRHLDVGGGLGVVYRDELPPQPSEYAKALLDRLERHRDLELIFEPGRAIAANAGVLVTKVEFLKHTEHKNFAIIDAAMNDLIRPALYQAWQDIIPLRPRQGEAQTYDLVGPVCETSDFLGKDRDLVLQEGDLLAVRSSGAYGFTMSSNYNTRPRVAEVMVDGNKTYLVRQREELSSLWALESVLPE

>1P0WA

TIKEMPQPKTFGELKNLPLLNTDKPVQALMKIADELGEIFKFEAPGRVTRYLSSQRLIKEACDESRFDKNLSQALKFVRDFAGDGLFTSWTHEKNWKKAHNILLPSFSQQAMKGYHAMMVDIAVQLVQKWERLNADEHIEVPEDMTRLTLDTIGLCGFNYRFNSFYRDQPHPFITSMVRALDEAMNKLQRANPDDPAYDENKRQFQEDIKVMNDLVDKIIADRKASGEQSDDLLTHMLNGKDPETGEPLDDENIRYQIITFLIAGHETTSGLLSFALYFLVKNPHVLQKAAEEAARVLVDPVPSYKQVKQLKYVGMVLNEALRLWPTAPAFSLYAKEDTVLGGEYPLEKGDELMVLIPQLHRDKTIWGDDVEEFRPERFENPSAIPQHAFKPWGNGQRACIGQQFALHEATLVLGMMLKHFDFEDHTNYELDIKETLTLKPEGFVVKAKSKKIPL

>1UJ0A

AGHMARRVRALYDFEAVEDNELTFKHGELITVLDDSDANWWQGENHRGTGLFPSNFVTTDLS

>1UJMA

AKIDNAVLPEGSLVLVTGANGFVASHVVEQLLEHGYKVRGTARSASKLANLQKRWDAKYPGRFETAVVEDMLKQGAYDEVIKGAAGVAHIASVVSFSNKYDEVVTPAIGGTLNALRAAAATPSVKRFVLTSSTVSALIPKPNVEGIYLDEKSWNLESIDKAKTLPESDPQKSLWVYAASKTEAELAAWKFMDENKPHFTLNAVLPNYTIGTIFDPETQSGSTSGWMMSLFNGEVSPALALMPPQYYVSAVDIGLLHLGCLVLPQIERRRVYGTAGTFDWNTVLATFRKLYPSKTFPADFPDQGQDLSKFDTAPSLEILKSLGRPGWRSIEESIKDLVGSETA

>1PAHA

TVPWFPRTIQELDRFANQILSYGAELDADHPGFKDPVYRARRKQFADIAYNYRHGQPIPRVEYMEEEKKTWGTVFKTLKSLYKTHACYEYNHIFPLLEKYCGFHEDNIPQLEDVSQFLQTCTGFRLRPVAGLLSSRDFLGGLAFRVFHCTQYIRHGSKPMYTPEPDICHELLGHVPLFSDRSFAQFSQEIGLASLGAPDEYIEKLATIYWFTVEFGLCKQGDSIKAYGAGLLSSFGELQYCLSEKPKLLPLELEKTAIQNYTVTEFQPLYYVAESFNDAKEKVRNFAATIPRPFSVRYDPYTQRIEVL

>1XKWA

ESTSATQPPGVTTLGKVPLKPRELPQSASVIDHERLEQQNLFSLDEAMQQATGVTVQPFQLLTTAYYVRGFKVDSFELDGVPALLGNTASSPQDMAIYERVEILRGSNGLLHGTGNPAATVNLVRKRPQREFAASTTLSAGRWDRYRAEVDVGGPLSASGNVRGRAVAAYEDRDYFYDVADQGTRLLYGVTEFDLSPDTLLTVGAQYQHIDSITNMAGVPMAKDGSNLGLSRDTYLDVDWDRFKWDTYRAFGSLEQQLGGGWKGKVSAEYQEADSRLRYAGSFGAIDPQTGDGGQLMGAAYKFKSIQRSLDANLNGPVRLFGLTHELLGGVTYAQGETRQDTARFLNLPNTPVNVYRWDPHGVPRPQIGQYTSPGTTTTTQKGLYALGRIKLAEPLTLVVGGRESWWDQDTPATRFKPGRQFTPYGGLIWDFARDWSWYVSYAEVYQPQADRQTWNSEPLSPVEGKTYETGIKGELADGRLNLSLAAFRIDLENNPQEDPDHPGPPNNPFYISGGKVRSQGFELEGTGYLTPYWSLSAGYTYTSTEYLKDSQNDSGTRYSTFTPRHLLRLWSNYDLPWQDRRWSVGGGLQAQSDYSVDYRGVSMRQGGYALVNMRLGYKIDEHWTAAVNVNNLFDRTYYQSLSNPNWNNRYGEPRSFNVSLRGAF

>2EX4A

MGSSHHHHHHSSGLVPRGSTSEVIEDEKQFYSKAKTYWKQIPPTVDGMLGGYGHISSIDINSSRKFLQRFLREGPNKTGTSCALDCGAGIGRITKRLLLPLFREVDMVDITEDFLVQAKTYLGEEGKRVRNYFCCGLQDFTPEPDSYDVIWIQWVIGHLTDQHLAEFLRRCKGSLRPNGIIVIKDNMAQEGVILDDVDSSVCRDLDVVRRIICSAGLSLLAEERQENLPDEIYHVYSFALR

>1ZDQA

ATAAEIAALPRQKVELVDPPFVHAHSQVAEGGPKVVEFTMVIEEKKIVIDDAGTEVHAMAFNGTVPGPLMVVHQDDYLELTLINPETNTLMHNIDFHAATGALGGGGLTEINPGEKTILRFKATKPGVFVYHCAPPGMVPWHVVSGGNGAIMVLPREGLHDGKGKALTYDKIYYVGEQDFYVPRDENGKYKKYEAPGDAYEDTVKVMRTLTPTHVVFNGAVGALTGDKAMTAAVGEKVLIVHSQANRDTRPHLIGGHGDYVWATGKFNTPPDVDQETWFIPGGAAGAAFYTFQQPGIYAYVNHNLIEAFELGAAAHFKVTGEWNDDLMTSVLAPSG

>3ME7A

MSLGTYVPGDITLVDSYGNEFQLKNLKGKPIILSPIYTHCRAACPLITKSLLKVIPKLGTPGKDFWVITFTFDPKDTLEDIKRFQKEYGIDGKGWKVVKAKTSEDLFKLLDAIDFRFMTAGNDFIHPNVVVVLSPELQIKDYIYGVNYNYLEFVNALRLARGEGHHHHHH

>2NT3A

GSHMSKKILIVESDTALSATLRSALEGRGFTVDETTDGKGSVEQIRRDRPDLVVLAVDLSAGQNGYLICGKLKKDDDLKNVPIVIIGNPDGFAQHRKLKAHADEAVAKPVDADQLVERAGALIGFPE

>1RFXA

SSMPLCPIDEAIDKKIKQDFNSLFPNAIKNIGLNCWTVSSRGKLASCPEGTAVLSCSCGSACGSWDIREEKVCHCQCARIDWTAARCCKLQVAS

>1C7JA

MTHQIVTTQYGKVKGTTENGVHKWKGIPYAKPPVGQWRFKAPEPPEVWEDVLDATAYGPVCPQPSDLLSLSYTELPRQSEDCLYVNVFAPDTPSQNLPVMVWIHGGAFYLGAGSEPLYDGSKLAAQGEVIVVTLNYRLGPFGFMHLSSFDEAYSDNLGLLDQAAALKWVRENISAFGGDPDNVTVFGESAGGMSIAALLAMPAAKGLFQKAIMESGASRTMTKEQAASTAAAFLQVLGINESQLDRLHTVAAEDLLKAADQLRIAEKENIFQLFFQPALDPKTLPEEPEKSIAEGAASGIPLLIGTTRDEGYLFFTSDSDVRSQETLDAALEYSLGKPLAEKAADLYPRSLESQIHMVTDLLFWRPAVAFASAQSHYAPVWMYRFDWHPEKPPYNKAFHALELPFVFGNLDGLERMAKAEITDEVKQLSHTIQSAWITFAKTGNPSTEAVNWPAYHEETRETVILDSEITIENDPESEKRQKLFPSKGE

>1H0HB

SKGFFVDTTRCTACRGCQVACKQWHGNPATPTENTGFHQNPPDFNFHTYKLVRMHEQEIDGRIDWLFFPDQCRHCIAPPCKATADMEDESAIIHDDATGCVLFTPKTKDLEDYESVISACPYDVPRKVAESNQMAKCDMCIDRITNGLRPACVTSCPTGAMNFGDLSEMEAMASARLAEIKAAYSDAKLCDPDDVRVIFLTAHNPKLYHEYAVA

>1ZE3D

DLYFNPRFLADDPQAVADLSRFENGQELPPGTYRVDIYLNNGYMATRDVTFNTGDSEQGIVPCLTRAQLASMGLNTASVAGMNLLADDACVPLTTMVQDATAHLDVGQQRLNLTIPQAFMSNRAR

>1UHAA

APECGERASGKRCPNGKCCSQWGYCGTTDNYCGQGCQSQCDYWRCGRDFGGRLCEEDMCCSKYGWCGYSDDHCEDGCQSQCD

>1J8MF

SKLLDNLRDTVRKFLTGSSSYDKAVEDFIKELQKSLISADVNVKLVFSLTNKIKERLKNEKPPTYIERREWFIKIVYDELSNLFGGDKEPKVIPDKIPYVIMLVGVQGTGKTTTAGKLAYFYKKKGFKVGLVGADVYRPAALEQLQQLGQQIGVPVYGEPGEKDVVGIAKRGVEKFLSEKMEIIIVDTAGRHGYGEEAALLEEMKNIYEAIKPDEVTLVIDASIGQKAYDLASKFNQASKIGTIIITKMDGTAKGGGALSAVAATGATIKFIGTGEKIDELEVFNPRRFVARLHHHH

>3K8GA

GSGAWKASVDPLGVVGSGADVYLYFPVAGNENLISRIIENHESKADIKKIVDRTTAVYGAFFARSKEFRLFGSGSYPYAFTNLIFSRSDGWASTKTEHGITYYESEHTDVSIPAPHFSCVIFGSSKRERMSKMLSRLVNPDRPQLPPRFEKECTSEGTSQTVALYIKNGGHFITKLLNFPQLNLPLGAMELYLTARRNEYLYTLSLQLGNAKINFPIQFLISRVLNAHIHVEGDRLIIEDGTISAERLASVISSLYSKKGSS

>2B1LA

MQFYQADVLTQGKPVLLNVWATWCPTCRAEHQYLNQLSAQGIRVVGMNYKDDRQKAISWLKELGNPYALSLFDGDGMLGLDLGVYGAPETFLIDGNGIIRYRHAGDLNPRVWEEEIKPLWEKYSKEAAQ

>2ZZ3A

GSHMRSRRVDVMDVMNRLILAMDLMNRDDALRVTGEVREYIDTVKIGYPLVLSEGMDIIAEFRKRFGCRIIAAFKVADIPETNEKICRATFKAGADAIIVHGFPGADSVRACLNVAEEMGREVFLLTEMSHPGAEMFIQGAADEIARMGVDLGVKNYVGPSTRPERLSRLREIIGQDSFLISPGVGAQGGDPGETLRFADAIIVGRSIYLADNPAAAAAGIIESIKDLRIPEDPAANKARKEAELAAATAEQ

>3GY1A

MSLEPTIITDVLCYITKPDRHNLVVVKVETNKGIYGLGCATFQQRPKAVSLVVSEYLKPILIGRDANNIEDLWQMMMVNSYWRNGPILNNAISGVDMALWDIKGKLANMPLYQLFGGKSRDAIAAYTHAVADNLEDLYTEIDEIRKKGYQHIRCQLGFYGGNSSEFHTTDNPTQGSYFDQDEYMRTTVSMFSSLREKYGYKFHILHDVHERLFPNQAVQFAKDVEKYKPYFIEDILPPDQNEWLGQIRSQTSTPLATGELFNNPMEWKSLIANRQVDFIRCHVSQIGGITPALKLGSLCAAFGVRIAWHTPSDITPIGVAVNIHLNINLHNAAIQENIEINDNTRCVFSGIPEAKNGFFYPIESPGIGVDIDENEIIKYPVEYRPHEWTQSRIPDGTIVTEGHHHHHH

>1XMZA

MRGSHHHHHHGSASFLKKTMPFKTTIEGTVNGHYFKCTGKGEGNPFEGTQEMKIEVIEGGPLPFAFHILSTSCXSKTFIKYVSGIPDYFKQSFPEGFTWERTTTYEDGGFLTAHQDTSLDGDCLVYKVKILGNNFPADGPVMQNKAGRWEPGTEIVYEVDGVLRGQSLMALKCPGGRHLTCHLHTTYRSKKPASALKMPGFHFEDHRIEIMEEVEKGKCYKQYEAAVGRYCDAAPSKLGHN

>2P35A

QGHMAWSAQQYLKFEDERTRPARDLLAQVPLERVLNGYDLGCGPGNSTELLTDRYGVNVITGIDSDDDMLEKAADRLPNTNFGKADLATWKPAQKADLLYANAVFQWVPDHLAVLSQLMDQLESGGVLAVQMPDNLQEPTHIAMHETADGGPWKDAFSGGGLRRKPLPPPSDYFNALSPKSSRVDVWHTVYNHPMKDADSIVEWVKGTGLRPYLAAAGEENREAFLADYTRRIAAAYPPMADGRLLLRFPRLFVVAVKK

>2P3JA

VLDGPYQPTTFNPPVDYWMLLAPTAAGVVVEGTNNTDAWLATILVEPNVTSETRSYTLFGTQEQITIANASQTQWKFIDVVKTTQNGSYSQYGPLQSTPKLYAVMKHNGKIYTYNGETPNVTTKYYSTTNYDSVNMTAFCDFYIIPREEESTCTEYINNGL

>2OFXA

QGHMATNVTYQAHHVSRNKRGQVVGTRGGFRGCTVWLTGLSGAGKTTVSMALEEYLVCHGIPCYTLDGDNIRQGLNKNLGFSPEDREENVRRIAEVAKLFADAGLVCITSFISPYTQDRNNARQIHEGASLPFFEVFVDAPLHVCEQRDVKGLYKKARAGEIKGFTGIDSEYEKPEAPELVLKTDSCDVNDCVQQVVELLQERDIVP

>2QSIA

GHMSGSLARAAARPNAPTLVDEATVDDFIAHSGKIVVLFFRGDAVRFPEAADLAVVLPELINAFPGRLVAAEVAAEAERGLMARFGVAVCPSLAVVQPERTLGVIAKIQDWSSYLAQIGAMLAEVDQPGEAELQSGS

>2J9FA

SSLDDKPQFPGASAEFIDKLEFIQPNVISGIPIYRVMDRQGQIINPSEDPHLPKEKVLKLYKSMTLLNTMDRILYESQRQGRISFYMTNYGEEGTHVGSAAALDNTDLVFGQYREAGVLMYRDYPLELFMAQCYGNISDLGKGRQMPVHYGCKERHFVTISSPLATQIPQAVGAAYAAKRANANRVVICYFGEGAASEGDAHAGFNFAATLECPIIFFCRNNGYAISTPTSEQYRGDGIAARGPGYGIMSIRVDGNDVFAVYNATKEARRRAVAENQPFLIEAMTYRIGHHSTSDDSSAYRPVDEVNYWDKQDHPISRLRHYLLSQGWWDEEQEKAWRKQSRRKVMEAFEQAERKPKPNPNLLFSDVYQEMPAQLRKQQESLARHLQTYGEHYPLDHFDK

>3BB0A

MGSVTPIPLPKIDEPEEYNTNYILFWNHVGLELNRVTHTVGGPLTGPPLSARALGMLHLAIHDAYFSICPPTDFTTFLSPDTENAAYRLPSPNGANDARQAVAGAALKMLSSLYMKPVEQPNPNPGANISDNAYAQLGLVLDRSVLEAPGGVDRESASFMFGEDVADVFFALLNDPRGASQEGYHPTPGRYKFDDEPTHPVVLIPVDPNNPNGPKMPFRQYHAPFYGKTTKRFATQSEHFLADPPGLRSNADETAEYDDAVRVAIAMGGAQALNSTKRSPWQTAQGLYWAYDGSNLIGTPPRFYNQIVRRIAVTYKKEEDLANSEVNNADFARLFALVDVACTDAGIFSWKEKWEFEFWRPLSGVRDDGRPDHGDPFWLTLGAPATNTNDIPFKPPFPAYPSGHATFGGAVFQMVRRYYNGRVGTWKDDEPDNIAIDMMISEELNGVNRDLRQPYDPTAPIEDQPGIVRTRIVRHFDSAWELMFENAISRIFLGVHWRFDAAAARDILIPTTTKDVYAVDNNGATVFQNVEDIRYTTRGTREDREGLFPIGGVPLGIEIADEIFNNGLKPTPPEIQPMPQETPVQKPVGQQPVKGMWEEEQAPVVKEAP

>3H9CA

TQVAKKILVTCALPYANGSIHLGHMLEHIQADVWVRYQRMRGHEVNFICADDAHGTPIMLKAQQLGITPEQMIGEMSQEHQTDFAGFNISYDNYHSTHSEENRQLSELIYSRLKENGFIKNRTISQLYDPEKGMFLPDRFVKGTCPKCKSPDQYGDNCEVCGATYSPTELIEPKSVVSGATPVMRDSEHFFFDLPSFSEMLQAWTRSGALQEQVANKMQEWFESGLQQWDISRDAPYFGFEIPNAPGKYFYVWLDAPIGYMGSFKNLCDKRGDSVSFDEYWKKDSTAELYHFIGKDIVYFHSLFWPAMLEGSNFRKPSNLFVHGYVTVNGAKMSKSRGTFIKASTWLNHFDADSLRYYYTAKLSSRIDDIDLNLEDFVQRVNADIVNKVVNLASRNAGFINKRFDGVLASELADPQLYKTFTDAAEVIGEAWESREFGKAVREIMALADLANRYVDEQAPWVVAKQEGRDADLQAICSMGINLFRVLMTYLKPVLPKLTERAEAFLNTELTWDGIQQPLLGHKVNPFKALYNRIDMRQVEALVEASK

>2FHLA

ARKCSLTGKWTNNLGSIMTIRAVNSRGEFTGTYLTAVADNPGNITLSPLLGIQHKRASQPTFGFTVHWNFSESTTVFTGQCFIDRNGKEVLKTMWLLRSSVNDISYDWKATRVGYNNFTRLS

>2PRVA

GMIYSKVENFINENKQNAIFTEGASHENIGRIEENLQCDLPNSYKWFLEKYGAGGLFGVLVLGYNFDHASVVNRTNEYKEHYGLTDGLVVIEDVDYFAYCLDTNKMKDGECPVVEWDRVIGYQDTVADSFIEFFYNKIQEAKDDWDEDEDWDD

>1JLJA

MATEGMILTNHDHQIRVGVLTVSDSCFRNLAEDRSGINLKDLVQDPSLLGGTISAYKIVPDEIEEIKETLIDWCDEKELNLILTTGGTGFAPRDVTPEATKEVIEREAPGMALAMLMGSLNVTPLGMLSRPVCGIRGKTLIINLPGSKKGSQECFQFILPALPHAIDLLRDAIVKVKEVHDRSHHHHHH

>1XFIA

SESDSEMVPFPQLPMPIENNYRACTIPYRFPSDDPKKATPNEISWINVFANSIPSFKKRAESDITVPDAPARAEKFAERYAGILEDLKKDPESHGGPPDGILLCRLREQVLRELGFRDIFKKVKDEENAKAISLFPQVVSLSDAIEDDGKRLENLVRGIFAGNIFDLGSAQLAEVFSRDGMSFLASCQNLVPRPWVIDDLENFQAKWINKSWKKAVIFVDNSGADIILGILPFARELLRRGAQVVLAANELPSINDITCTELTEILSQLKDENGQLLGVDTSKLLIANSGNDLPVIDLSRVSQELAYLSSDADLVIVEGMGRGIETNLYAQFKCDSLKIGMVKHLEVAEFLGGRLYDCVFKFNEVQS

>1KZ1A

MFSGIKGPNPSDLKGPELRILIVHARGNLQAIEPLVKGAVETMIEKHDVKLENIDIESVPGSWELPQGIRASIARNTYDAVIGIGVLIKGSTMHFEYISEAVVHGLMRVGLDSGVPVILGLLTVLNEEQALYRAGLNGGHNHGNDWGSAAVEMGLKALY

>2WL5A

STPSIVIASAARTAVGSFNGAFANTPAHELGATVISAVLERAGVAAGEVNEVILGQVLPAGEGQNPARQAAMKAGVPQEATAWGMNQLCGSGLRAVALGMQQIATGDASIIVAGGMESMSMAPHCAHLRGGVKMGDFKMIDTMIKDGLTDAFYGYHMGTTAENVAKQWQLSRDEQDAFAVASQNKAEAAQKDGRFKDEIVPFIVKGRKGDITVDADEYIRHGATLDSMAKLRPAFDKEGTVTAGNASGLNDGAAAALLMSEAEASRRGIQPLGRIVSWATVGVDPKVMGTGPIPASRKALERAGWKIGDLDLVEANEAFAAQACAVNKDLGWDPSIVNVNGGAIAIGNPIGASGARILNTLLFEMKRRGARKGLATLCIGGGMGVAMCIESL

>1E2XA

FADRMVIKAQSPAGFAEEYIIESIWNNRFPPGTILPAERELSELIGVTRTTLREVLQRLARDGWLTIQHGKPTKVNNFWETSGLNILETLARLDHESVPQLIDNLLSVRTNISTIFIRTAFRQHPDKAQEVLATANEVADHADAFAELDYNIFRGLAFASGNPIYGLILNGMKGLYTRIGRHYFANPEARSLALGFYHKLSALCSEGAHDQVYETVRRYGHESGEIWHRMQKNLPGDLAIQGR

>2O7IA

MQVSLPREDTVYIGGALWGPATTWNLYAPQSTWGTDQFMYLPAFQYDLGRDAWIPVIAERYEFVDDKTLRIYIRPEARWSDGVPITADDFVYALELTKELGIGPGGGWDTYIEYVKAVDTKVVEFKAKEENLNYFQFLSYSLGAQPMPKHVYERIRAQMNIKDWINDKPEEQVVSGPYKLYYYDPNIVVYQRVDDWWGKDIFGLPRPKYLAHVIYKDNPSASLAFERGDIDWNGLFIPSVWELWEKKGLPVGTWYKKEPYFIPDGVGFVYVNNTKPGLSDPAVRKAIAYAIPYNEMLKKAYFGYGSQAHPSMVIDLFEPYKQYIDYELAKKTFGTEDGRIPFDLDMANKILDEAGYKKGPDGVRVGPDGTKLGPYTISVPYGWTDWMMMCEMIAKNLRSIGIDVKTEFPDFSVWADRMTKGTFDLIISWSVGPSFDHPFNIYRFVLDKRLSKPVGEVTWAGDWERYDNDEVVELLDKAVSTLDPEVRKQAYFRIQQIIYRDMPSIPAFYTAHWYEYSTKYWINWPSEDNPAWFRPSPWHADAWPTLFIISKKSDPQPVPSWLGTVDEGGIEIPTAKIFEDLQKATMHHHHHH

>1YU0A

MSTAVQFRGGTTAQHATFTGAAREITVDTDKNTVVVHDGATAGGFPLARHDLVKTAFIKADKSAVAFTRTGNATASIKAGTIVEVNGKLVQFTADTAITMPALTAGTDYAIYVCDDGTVRADSNFSAPTGYTSTTARKVGGFHYAPGSNAAAQAGGNTTAQINEYSLWDIKFRPAALDPRGMTLVAGAFWADIYLLGVNHLTDGTSKYNVTIADGSASPKKSTKFGGDGSAAYSDGAWYNFAEVMTHHGKRLPNYNEFQALAFGTTEATSSGGTDVPTTGVNGTGATSAWNIFTSKWGVVQASGCLWTWGNEFGGVNGASEYTANTGGRGSVYAQPAAALFGGAWNGTSLSGSRAALWYSGPSFSFAFFGARGVCDHLILE

>1YUKA

QECTKFKVSSCRECIESGPGCTWCQKLNFTGPGDPDSIRCDTRPQLLMRGCAADDIMDPTSLAETQEDHNGGQKQLSPQKVTLYLRPGQAAAFNVTFRRAKGY

>2QNDA

ASRFHEQFIVREDLMGLAIGTHGANIQQARKVPGVTAIDLDEDTCTFHIYGEDQDAVKKARSFLEFAEDVIQVPRNLVGKVIGKNGKLIQEIVDKSGVVRVRIEAENEKNVPQEEGMVPFVFVGTKDSIANATVLLDYHLNYLK

>3EOFA

GMMDTVKNRRTIRKYQQKDITPDLLNDLLETSFRASTMGGMQLYSVVVTRDAEKKEILSPAHFNQPMVKEAPVVLTFCADFRRFCKYCQERNAVPGYGNLMSFLNAAMDTLLVAQTFCTLAEEAGLGICYLGTTTYNPQMIIDALHLPELVFPITTVTVGYPAESPKQVDRLPIEGIIHEESYHDYTAEDINRLYAYKESLPENKLFIEENQKETLPQVFTDVRYTKKDNEFMSENLLKVLRRQGFMD

>2GRRA

GSHMSGIALSRLAQERKAWRKDHPFGFVAVPTKNPDGTMNLMNWECAIPGKKGTPWEGGLFKLRMLFKDDYPSSPPKCKFEPPLFHPNVYPSGTVCLSILEEDKDWRPAITIKQILLGIQELLNEPNIQSPAQAEAYTIYCQNRVEYEKRVRAQAKKFAPS

>3EJVA

MGSDKIHHHHHHENLYFQGMTMADETIILNVLGQYTRAHDRRDPDAMAALFAPEATIEIVDAVGGASRSISRLEGRDAIRVAVRQMMAPHGYRAWSQNVVNAPIIVIEGDHAVLDAQFMVFSILAAEVPDGGWPTGTFGAQGRIVPIEAGQYRLTLRTVADGWVISAMRIEHRLPMAFG

>3GWBA

ELDGKAPSHRNLNVQTWSTAEGAKVLFVEARELPMFDLRLIFAAGSSQDGNAPGVALLTNAMLNEGVAGKDVGAIAQGFEGLGADFGNGAYKDMAVASLRSLSAVDKREPALKLFAEVVGKPTFPADSLARIKNQMLAGFEYQKQNPGKLASLELMKRLYGTHPYAHASDGDAKSIPPITLAQLKAFHAKAYAAGNVVIALVGDLSRSDAEAIAAQVSAALPKGPALAKIEQPAEPKASIGHIEFPSSQTSLMLAQLGIDRDDPDYAAVSLGNQILGGGGFGTRLMSEVREKRGLTYGVYSGFTPMQARGPFMINLQTRAEMSEGTLKLVQDVFAEYLKNGPTQKELDDAKRELAGSFPLSTASNADIVGQLGAMGFYNLPLSYLEDFMRQSQELTVEQVKAAMNKHLNVDKMVIVSAGPTVAQKPLEHHHHHH

>1GWTA

MQLTPTFYDNSCPNVSNIVRDTIVNELRSDPRIAASILRLHFHDCFVNGCDASILLDNTTSFRTEKDAFGNANSARGFPVIDRMKAAVESACPRTVSCADLLTIAAQQSVTLAGGPSWRVPLGRRDSLQAFLDLANANLPAPFFTLPQLKDSFRNVGLNRSSDLVALSGGHTFGKNQCRFIMDRLYNFSNTGLPDPTLNTTYLQTLRGLCPLNGNLSALVDMDLRTPTIFDNKYYVNLEEQKGLIQSDQELFSSPNATDTIPLVRSFANSTQTFFNAFVEAMDRMGNITPLTGTQGQIRLNCRVVNSNS

>1E39A

ADNLAEFHVQNQECDSCHTPDGELSNDSLTYENTQCVSCHGTLAEVAETTKHEHYNAHASHFPGEVACTSCHSAHEKSMVYCDSCHSFDFNMPYAKKWLRDEPTIAELAKDKSERQAALASAPHDTVDVVVVGSGGAGFSAAISATDSGAKVILIEKEPVIGGNAKLAAGGMNAAWTDQQKAKKITDSPELMFEDTMKGGQNINDPALVKVLSSHSKDSVDWMTAMGADLTDVGMMGGASVNRAHRPTGGAGVGAHVVQVLYDNAVKRNIDLRMNTRGIEVLKDDKGTVKGILVKGMYKGYYWVKADAVILATGGFAKNNERVAKLDPSLKGFISTNQPGAVGDGLDVAENAGGALKDMQYIQAAPTLSVKGGVMVTEAVRGNGAILVNREGKRFVNEITTRDKASAAILAQTGKSAYLIFDDSVRKSLSKIDKYIGLGVAPTADSLVKLGKMEGIDGKALTETVARYNSLVSSGKDTDFERPNLPRALNEGNYYAIEVTPGVHHTMGGVMIDTKAEVMNAKKQVIPGLYGAGEVTGGVHGANRLGGNAISDIITFGRLAGEEAAKYSKKN

>3E3UA

MAVVPIRIVGDPVLHTATTPVTVAADGSLPADLAQLIATMYDTMDAANGVGLAANQIGCSLRLFVYDCAADRAMTARRRGVVINPVLETSEIPETMPDPDTDDEGCLSVPGESFPTGRAKWARVTGLDADGSPVSIEGTGLFARMLQHETGHLDGFLYLDRLIGRYARNAKRAVKSHGWGVPGLSWLPGEDPDPFGH

>1E30A

GTLDTTWKEATLPQVKAMLEKDTGKVSGDTVTYSGKTVHVVAAAVLPGFPFPSFEVHDKKNPTLEIPAGATVDVTFINTNKGFGHSFDITKKGPPYAVMPVIDPIVAGTGFSPVPKDGKFGYTNFTWHPTAGTYYYVCQIPGHAATGQFGKIVVK

>3K2CA

MAHHHHHHMGTLEAQTQGPGSMAKEASGNVYFDVYANEESLGRIVMKLEDDIVPKTAKNFRTLCERPKGEGYKGSTFHRIIPGFMVQGGDYTAHNGTGGRSIYGEKFPDENFELKHTKEGILSMANCGAHTNGSQFFITLGKTQWLDEKHVVFGEVVEGMDVVHKIAKYGSESGQVKKGYRIEIRDCGVLGSN

>2R1BA

GSSLRGGHAGTTYIFSKGGGQITYKWPPNDRPSTRADRLAIGFSTVQKEAVLVRVDSSSGLGDYLELHIHQGKIGVKFNVGTDDIAIEESNAIINDGKYHVVRFTRSGGNATLQVDSWPVIERYPAGNNDNERLAIARQRIPYRLGRVVDEWLLDKGRQLTIFNSQATIIIGGKEQGQPFQGQLSGLYYNGLKVLNMAAENDANIAIVGNVRLVGEVPSS

>1I9YA

YDPIHEYVNHELRKRENEFSEHKNVKIFVASYNLNGCSATTKLENWLFPENTPLADIYVVGFQEIVQLTPQQVISADPAKRREWESCVKRLLNGKCTSGPGYVQLRSGQLVGTALMIFCKESCLPSIKNVEGTVKKTGLGGVSGNKGAVAIRFDYEDTGLCFITSHLAAGYTNYDERDHDYRTIASGLRFRRGRSIFNHDYVVWFGDFNYRISLTYEEVVPCIAQGKLSYLFEYDQLNKQMLTGKVFPFFSELPITFPPTYKFDIGTDIYDTSDKHRVPAWTDRILYRGELVPHSYQSVPLYYSDHRPIYATYEANIVKVDREKKKILFEELYNQRKQEVRDASQTS

>1OAIA

PTLSPEQQEMLQAFSTQSGMNLEWSQKCLQDNNWDYTRSAQAFTHLKAKGEIPEVAFMK

>3C1JA

APAVADKADNAFMMICTALVLFMTIPGIALFYGGLIRGKNVLSMLTQVTVTFALVCILWVVYGYSLAFGEGNNFFGNINWLMLKNIELTAVMGSIYQYIHVAFQGSAACITVGLIVGALAERIRFSAVLIFVVVWLTLSYIPIAHMVWGGGLLASHGALDFAGGTVVHINAAIAGLVGAYLIGKRVGFGKEAFKPHNLPMVFTGTAILYIGWFGANAGSAGTANEIAALAFVNTVVATAAAILGWIFGEWALRGKPSLLGACSGAIAGLVGVTPACGYIGVGGALIIGVVAGLAGLWGVTMLKRLLRVDDPCDVFGVHGVCGIVGCIMTGIFAASSLGGVGFAEGVTMGHQLLVQLESIAITIVWSGVVAFIGYKLADLTVGLRVPEEQEREGLDVNSHGENAYNADQAQQPAQADLEHHHHHH

>2C15A

MSFTPANRAYPYTRLRRNRRDDFSRRLVRENVLTVDDLILPVFVLDGVNQRESIPSMPGVERLSIDQLLIEAEEWVALGIPALALFPVTPVEKKSLDAAEAYNPEGIAQRATRALRERFPELGIITDVALDPFTTHGQDGILDDDGYVLNDVSIDVLVRQALSHAEAGAQVVAPSDMMDGRIGAIREALESAGHTNVRVMAYSAKYASAYYGPFRDAVGSASNLGKGNKATYQMDPANSDEALHEVAADLAEGADMVMVXPGMPYLDIVRRVKDEFRAPTFVYQVSGEYAMHMGAIQNGWLAESVILESLTAFKRAGADGILTYFAKQAAEQLRRGR

>1QL3A

ADPAAGEKVFGKCKACHKLDGNDGVGPHLNGVVGRTVAGVDGFNYSDPMKAHGGDWTPEALQEFLTNPKAVVKGTKMAFAGLPKIEDRANLIAYLEGQQ

>1CHMA

QMPKTLRIRNGDKVRSTFSAQEYANRQARLRAHLAAENIDAAIFTSYHNINYYSDFLYCSFGRPYALVVTEDDVISISANIDGGQPWRRTVGTDNIVYTDWQRDNYFAAIQQALPKARRIGIEHDHLNLQNRDKLAARYPDAELVDVAAACMRMRMIKSAEEHVMIRHGARIADIGGAAVVEALGDQVPEYEVALHATQAMVRAIADTFEDVELMDTWTWFQSGINTDGAHNPVTTRKVNKGDILSLNCFPMIAGYYTALERTLFLDHCSDDHLRLWQVNVEVHEAGLKLIKPGARCSDIARELNEIFLKHDVLQYRTFGYGHSFGTLSHYYGREAGLELREDIDTVLEPGMVVSMEPMIMLPEGLPGAGGYREHDILIVNENGAENITKFPYGPEKNIIR

>3CZVA

GSMSRLSWGYREHNGPIHWKEFFPIADGDQQSPIEIKTKEVKYDSSLRPLSIKYDPSSAKIISNSGHSFNVDFDDTENKSVLRGGPLTGSYRLRQVHLHWGSADDHGSEHIVDGVSYAAELHVVHWNSDKYPSFVEAAHEPDGLAVLGVFLQIGEPNSQLQKITDTLDSIKEKGKQTRFTNFDLLSLLPPSWDYWTYPGSLTVPPLLESVTWIVLKQPINISSQQLAKFRSLLCTAEGEAAAFLVSNHRPPQPLKGRKVRASFH

>1CZYA

AMADLEQKVLEMEASTYDGVFIWKISDFPRKRQEAVAGRIPAIFSPAFYTSRYGYKMCLRIYLNGDGTGRGTHLSLFFVVMKGPNDALLRWPFNQKVTLMLLDQNNREHVIDAFRPDVTSSSFQRPVNDMNIASGCPLFCPVSKMEAKNSYVRDDAIFIKAIVDLTGL

>2V5IA

MVSVGDAAFRQEANKKFKYSVKLSDYSTLQDAVTDAVDGLLIDINYNFTDGESVDFXGKILTINCKAKFIGDGALIFNNMGPGSVINQPFMESKTTPWVIFPWDADGKWITDAALVAATLKQSKIEGYQPGVNDWVKFPGLEALLPQNVKDQHIAATLDIRSASRVEIRNAGGLMAAYLFRSCHHCKVIDSDSIIGGKDGIITFENLSGDWGLGNYVIGGRVHYGSGSGVQFLRNNGGESHNGGVIGVTSWRAGESGFKTYQGSVGGGTARNYNLQFRDSVALSPVWDGFDLGSDPGMAPEPDRPGDLPVSEYPFHQLPNNHLVDNILVMNSLGVGLGMDGSGGYVSNVTVQDCAGAGMLAHTYNRVFSNITVIDCNYLNFDSDQIIIIGDCIVNGIRAAGIKPQPSNGLVISAPNSTISGLVGNVPPDKILVGNLLDPVLGQSRVIGFNSDTAELALRINKLSATLDSGALRSHLNGYAGSGSAWTELTALSGSTPNAVSLKVNRGDYKTTEIPISGTVLPDEGVLDINTMSLYLDAGALWALIRLPDGSKTRMKLSV

>1YCLA

PSVESFELDHNAVVAPYVRHCGVHKVGTDGVVNKFDIRFCQPNKQAMKPDTIHTLEHLLAFTIRSHAEKYDHFDIIDISPMGAQTGYYLVVSGEPTSAEIVDLLEDTMKEAVEITEIPAANEKQCGQAKLHDLEGAKRLMRFWLSQDKEELLKVFG

>2G8FA

GSHMAKEEIIWESLSVDVGSQGNPGIVEYKGVDTKTGEVLFEREPIPIGTNNMGEFLAIVHGLRYLKERNSRKPIYSDSQTAIKWVKDKKAKSTLVRNEETALIWKLVDEAEEWLNTHTYETPILKWQTDKWGAIKADYGRK

>1G8KB

RTTLAYPATAVSVAKNLAANEPVSFTYPDTSSPCVAVKLGAPVPGGVGPDDDIVAYSVLCTHMGCPTSYDSSSKTFSCPCHFTEFDAEKAGQMICGEATADLPRVLLRYDAASDALTAVGVDGLIYGRQANVI

>2NS9A

SLRLHAGVWGLKVRYEGSFEVSKTPEEVFEFLTDPKRFSRAFPGFKSVEVEDGSFTIELRLSLGPLRGDARVRASFEDLEKPSKATVKGSGRGAGSTLDFTLRFAVEPSGGGSRVSWVFEGNVGGLAASMGGRVLDSLARRMINDVISGVKRELGEA

>2V9MA

MQNITQSWFVQGMIKATTDAWLKGWDERNGGNLTLRLDDADIAPYHDNFHQQPRYIPLSQPMPLLANTPFIVTGSGKFFRNVQLDPMANLGIVKVDSDGAGYHILWGLFNEAVPTSELPAHFLSHCERIKATNGKDRVIMHCHATNLIALTYVLENDTAVFTRQLWEGSTECLVVFPDGVGILPWMVPGTDAIGQATAQEMQKHSLVLWPFHGVFGSGPTLDETFGLIDTAEKSAQVLVKVYSMGGMKQTISREELIALGKRFGVTPLASALAL

>2QIQA

AGFRKMAFPSGKVEGCMVQVTCGTTTLNGLWLDDTVYCPRHVICTAEDMLNPNYEDLLIRKSNHSFLVQAGNVQLRVIGHSMQNCLLRLKVDTSNPKTPKYKFVRIQPGQTFSVLACYNGSPSGVYQCAMRPNHTIKGSFLNGSCGSVGFNIDYDCVSFCYMHHMELPTGVHAGTDLEGKFYGPFVDRQTAQAAGTDTTITLNVLAWLYAAVINGDRWFLNRFTTTLNDFNLVAMKYNYEPLTQDHVDILGPLSAQTGIAVLDMCAALKELLQNGMNGRTILGSTILEDEFTPFDVVRQCS

>2Z25A

TAPSQVLKIRRPDDWHLHLRDGDMLKTVVPYTSEIYGRAIVMPNLAPPVTTVEAAVAYRQRILDAVPAGHDFTPLMTCYLTDSLDPNELERGFNEGVFTAAKLYPANATVNSSHGVTSVDAIMPVLERMEKIGMPLLVHGEVTHADIDIFDREARFIESVMEPLRQRLTALKVVFEHITTKDAADYVRDGNERLAATITPQHLMFNRNHMLVGGVRPHLYCLPILKRNIHQQALRELVASGFNRVFLGTDSAPHARHRKESSCGCAGCFNAPTALGSYATVFEEMNALQHFEAFCSVNGPQFYGLPVNDTFIELVREEQQVAESIALTDDTLVPFLAGETVRWSVKQ

>3NZNA

SNAVNLFGQKDRGNHVSGVDRGKVIMYGLSTCVWCKKTKKLLTDLGVDFDYVYVDRLEGKEEEEAVEEVRRFNPSVSFPTTIINDEKAIVGFKEKEIRESLGF

>1YPQA

RVANCSAPCPQDWIWHGENCYLFSSGSFNWEKSQEKCLSLDAKLLKINSTADLDFIQQAISYSSFPFWMGLSRRNPSYPWLWEDGSPLMPHLFRVRGAVSQTYPSGTCAYIQRGAVYAENCILAAFSICQKKANL

>3ENUA

TIEVPVLTFVPVQVSAELENRGCWVKFFDKKNFQGDSLFLSGPATLPRLIGPFGYDWENKVRSVKVGPRANLTIFDNHNYRDEDKFLDAGANVANLSKEMGFFDNFRSMVLNCI

>3APAA

GSARSSSYSGEYGSGGGKRFSHSGNQLDGPITALRVRVNTYYIVGLQVRYGKVWSDYVGGRNGDLEEIFLHPGESVIQVSGKYKWYLKKLVFVTDKGRYLSFGKDSGTSFNAVPLHPNTVLRFISGRSGSLIDAIGLHWDV

>2Q5XA

GIILTKVGYYTIPSMDDLAKITNEKGECIVSDFTIGRKGYGSIYFEGDVNLTNLNLDDIVHIRRKEVVVYLDDNQKPPVGEGLNRKAEVTLDGVWPTDKTSRCLIKSPDRLADINYEGRLEAVSRKQGAQFKEYRPETGSWVFKVSHFAKYGLQD

>2Q66A

KVFGITGPVSTVGATAAENKLNDSLIQELKKEGSFETEQETANRVQVLKILQELAQRFVYEVSKKKNMSDGMARDAGGKIFTYGSYRLGVHGPGSDIDTLVVVPKHVTREDFFTVFDSLLRERKELDEIAPVPDAFVPIIKIKFSGISIALICARLDQPQVPLSLTLSDKNLLRNLDEKDLRALNGTRVTDEILELVPKPNVFRIALRAIKLWAQRRAVYANIFGFPGGVAWAMLVARICQLYPNACSAVILNRFFIILSEWNWPQPVILKPIEDGPLQVRVWNPKIYAQDRSHRMPVITPAYPSMCATHNITESTKKVILQEFVRGVQITNDIFSNKKSWANLFEKNDFFFRYKFYLEITAYTRGSDEQHLKWSGLVESKVRLLVMKLEVLAGIKIAHPFTKPFESSYCCPTEDDYEMIQDKYGSHKTETALNALKLVTDENKEEESIKDAPKAYLSTMYIGLDFNIENKKEKVDIHIPCTEFVNLCRSFNEDYGDHKVFNLALRFVKGYDLPDEVFDENEKRP

>1DQEA

SQEVMKNLSLNFGKALDECKKEMTLTDAINEDFYNFWKEGYEIKNRETGCAIMCLSTKLNMLDPEGNLHHGNAMEFAKKHGADETMAQQLIDIVHGCEKSTPANDDKCIWTLGVATCFKAEIHKLNWAPSMDVAVGE

>1PC5A

MAFVVTDNCIKCKYTDCVEVCPVDCFYEGPNFLVIHPDECIDCALCEPECGAQAIFSEDEVPEDMQEFIQLNAELAEVWPNITEKKDPLPDAEDWDGVKGKLQHLER

>2FW6A

MMSETAPLPSASSALEDKAASAPVVGIIMGSQSDWETMRHADALLTELEIPHETLIVSANRTPDRLADYARTAAERGLNVIIAGAGGAAHLPGMCAAWTRLPVLGVPVESRALKGMDSLLSIVQMPGGVPVGTLAIGASGAKNAALLAASILALYNPALAARLETWRALQTASVPNSPITEDK

>2QQRA

GHMQSITAGQKVISKHKNGRFYQCEVVRLTTETFYEVNFDDGSFSDNLYPEDIVSQDCLQFGPPAEGEVVQVRWTDGQVYGAKFVASHPIQMYQVEFEDGSQLVVKRDDVYTLDEELP

>1BF6A

SFDPTGYTLAHEHLHIDLSGFKNNVDCRLDQYAFICQEMNDLMTRGVRNVIEMTNRYMGRNAQFMLDVMRETGINVVACTGYYQDAFFPEHVATRSVQELAQEMVDEIEQGIDGTELKAGIIAEIGTSEGKITPLEEKVFIAAALAHNQTGRPISTHTSFSTMGLEQLALLQAHGVDLSRVTVGHCDLKDNLDNILKMIDLGAYVQFDTIGKNSYYPDEKRIAMLHALRDRGLLNRVMLSMDITRRSHLKANGGYGYDYLLTTFIPQLRQSGFSQADVDVMLRENPSQFFQ

>3AHYA

MHHHHHHMLPKDFQWGFATAAYQIEGAVDQDGRGPSIWDTFCAQPGKIADGSSGVTACDSYNRTAEDIALLKSLGAKSYRFSISWSRIIPEGGRGDAVNQAGIDHYVKFVDDLLDAGITPFITLFHWDLPEGLHQRYGGLLNRTEFPLDFENYARVMFRALPKVRNWITFNEPLCSAIPGYGSGTFAPGRQSTSEPWTVGHNILVAHGRAVKAYRDDFKPASGDGQIGIVLNGDFTYPWDAADPADKEAAERRLEFFTAWFADPIYLGDYPASMRKQLGDRLPTFTPEERALVHGSNDFYGMNHYTSNYIRHRSSPASADDTVGNVDVLFTNKQGNCIGPETQSPWLRPCAAGFRDFLVWISKRYGYPPIYVTENGTSIKGESDLPKEKILEDDFRVKYYNEYIRAMVTAVELDGVNVKGYFAWSLMDNFEWADGYVTRFGVTYVDYENGQKRFPKKSAKSLKPLFDELIAAA

>2AHEA

MALSMPLNGLKEEDKEPLIELFVKAGSDGESIGNCPFSQRLFMILWLKGVVFSVTTVDLKRKPADLQNLAPGTHPPFITFNSEVKTDVNKIEEFLEEVLCPPKYLKLSPKHPESNTAGMDIFAKFSAYIKNSRPEANEALERGLLKTLQKLDEYLNSPLPDEIDENSMEDIKFSTRKFLDGNEMTLADCNLLPKLHIVKVVAKKYRNFDIPKEMTGIWRYLTNAYSRDEFTNTCPSDKEVEIAYSDVAKRLPSKVPKGEFQHTGGRY

>1OX3A

ADIVLNDLPFVDGPPAEGQSRISWIKNGEEILGADTQYGSEGSMNRPTVSVLRNVEVLDKNIGILKTSLETANSDIKTIQEAGYIPEAPRDGQAYVRKDGEWVLLSTFL

>1QZ0A

MRERPHTSGHHGAGEARATAPSTVSPYGPEARAELSSRLTTLRNTLAPATNDPRYLQACGGEKLNRFRDIQCRRQTAVRADLNANYIQVGNTRTIACQYPLQSQLESHFRMLAENRTPVLAVLASSSEIANQRFGMPDYFRQSGTYGSITVESKMTQQVGLGDGIMADMYTLTIREAGQKTISVPVVHVGNWPDQTAVSSEVTKALASLVDQTAETKRNMYESKGSSAVADDSKLRPVIHCRAGVGRTAQLIGAMCMNDSRNSQLSVEDMVSQMRVQRNGIMVQKDEQLDVLIKLAEGQGRPLLNS

>1LT4A

NGDRLYRADSRPPDEIKRSGGLMPRGHNEYFDRGTQMNINLYDHARGTQTGFVRYDDGYVSTKLSLRSAHLAGQSILSGYSTYYIYVIATAPNMFNVNDVLGVYSPHPYEQEVSALGGIPYSQIYGWYRVNFGVIDERLHRNREYRDRYYRNLNIAPAEDGYRLAGFPPDHQAWREEPWIHHAPQGCGNSSNSSRTITRTITGDTCNEETQNLSTIYLREYQSKVKRQIFSDYQSEVDIYNRIRDEL

>1OCBA

YNGNPFEGVQLWANNYYRSEVHTLAIPQITDPALRAAASAVAEVPSFQWLDRNVTVDTLLVQTLSEIREANQAGANPQYAAQIVVYDLPDRDCAAAASNGEWAIANNGVNNYKAYINRIREILISFSDVRTILVIEPDSLANMVTNMNVPKCSGAASTYRELTIYALKQLDLPHVAMYMDAGHAGWLGWPANIQPAAELFAKIYEDAGKPRAVRGLATNVANYNAWSVSSPPPYTSPNPNYDEKHYIEAFRPLLEARGFPAQFIVDQGRSGKQPTGQKEWGHWCNAIGTGFGMRPTANTGHQYVDAFVWVKPGGECDGTSDTTAARYDYHCGLEDALKPAPEAGQWFNEYFIQLLRNANPPF

>3OCCA

MATPHINAEMGDFADVVLMPGDPLRAKFIAETFLQDVREVNNVRGMLGFTGTYKGRKISVMGHGMGIPSCSIYAKELITDFGVKKIIRVGSCGAVRTDVKLRDVVIGMGACTDSKVNRMRFKDHDYAAIADFEMTRNAVDAAKAKGVNVRVGNLFSADLFYTPDPQMFDVMEKYGILGVEMEAAGIYGVAAEFGAKALTICTVSDHIRTGEQTTAAERQTTFNDMIEIALESVLLGDNA

>1ZCJA

ASGQAKALQYAFFAEKSANKWSTPSGASWKTASAQPVSSVGVLGLGTMGRGIAISFARVGISVVAVESDPKQLDAAKKIITFTLEKEASRAHQNGQASAKPKLRFSSSTKELSTVDLVVEAVFEDMNLKKKVFAELSALCKPGAFLCTNTSALNVDDIASSTDRPQLVIGTHFFSPAHVMRLLEVIPSRYSSPTTIATVMSLSKKIGKIGVVVGNCYGFVGNRMLAPYYNQGFFLLEEGSKPEDVDGVLEEFGFKMGPFRVSDLAGLDVGWKIRKGQGLTGPSLPPGTPVRKRGNSRYSPLGDMLCEAGRFGQKTGKGWYQYDKPLGRIHKPDPWLSTFLSQYREVHHIEQRTISKEEILERCLYSLINEAFRILEEGMAARPEHIDVIYLHGYGWPRHKGGPMFYAASVGLPTVLEKLQKYYRQNPDIPQLEPSDYLRRLVAQGSPPLKEWQSLAGPHGSKL

>1LVMA

GHHHHHHHGESLFKGPRDYNPISSTICHLTNESDGHTTSLYGIGFGPFIITNKHLFRRNNGTLLVQSLHGVFKVKNTTTLQQHLIDGRDMIIIRMPKDFPPFPQKLKFREPQREERICLVTTNFQTKSMSSMVSDTSCTFPSSDGIFWKHWIQTKDGQCGSPLVSTRDGFIVGIHSASNFTNTNNYFTSVPKNFMELLTNQEAQQWVSGWRLNADSVLWGGHKVFMDKP

>1E0WA

AESTLGAAAAQSGRYFGTAIASGRLSDSTYTSIAGREFNMVTAENEMKIDATEPQRGQFNFSSADRVYNWAVQNGKQVRGHTLAWHSQQPGWMQSLSGSALRQAMIDHINGVMAHYKGKIVQWDVVNEAFADGSSGARRDSNLQRSGNDWIEVAFRTARAADPSAKLCYNDYNVENWTWAKTQAMYNMVRDFKQRGVPIDCVGFQSHFNSGSPYNSNFRTTLQNFAALGVDVAITELDIQGAPASTYANVTNDCLAVSRCLGITVWGVRDSDSWRSEQTPLLFNNDGSKKAAYTAVLDALNGGDSSEPPADGG

>3EMHA

GPLGSPEFQSKPTPVKPNYALKFTLAGHTKAVSSVKFSPNGEWLASSSADKLIKIWGAYDGKFEKTISGHKLGISDVAWSSDSNLLVSASDDKTLKIWDVSSGKCLKTLKGHSNYVFCCNFNPQSNLIVSGSFDESVRIWDVKTGKCLKTLPAHSDPVSAVHFNRDGSLIVSSSYDGLCRIWDTASGQCLKTLIDDDNPPVSFVKFSPNGKYILAATLDNTLKLWDYSKGKCLKTYTGHKNEKYCIFANFSVTGGKWIVSGSEDNLVYIWNLQTKEIVQKLQGHTDVVISTACHPTENIIASAALENDKTIKLWKSDC

>3G5JA

SNAMSVIKIEKALKLDKVIFVDVRTEGEYEEDHILNAINMPLFKNNEHNEVGTIYKMQGKHEAIQKGFDYVSYKLKDIYLQAAELALNYDNIVIYCARGGMRSGSIVNLLSSLGVNVYQLEGGYKAYRNFVLEY

>3B7AA

MTMEQFLTSLDMIRSGCAPKFKLKTEDLDRLRVGDFNFPPSQDLMCYTKCVALMAGTVNKKGEFNAPKALAQLPHLVPPEMMEMSRKSVEACRDTHKQFKESCERVYQTAKCFSENADGQFMWP

>3B7CA

GMPTDDIVQLLKGQEEAWNRGDLDAYMQGYWQNEQLMLISNGKFRNGWDETLAAYKKNYPDKESLGELKFTIKEIKMLSNYAAMVVGRWDLKRLKDTPTGVFTLLVEKIDDRWVITMDHSSD

>2AD6A

DADLDKQVNTAGAWPIATGGYYSQHNSPLAQINKSNVKNVKAAWSFSTGVLNGHEGAPLVIGDMMYVHSAFPNNTYALNLNDPGKIVWQHKPKQDASTKAVMCCDVVDRGLAYGAGQIVKKQANGHLLALDAKTGKINWEVEVCDPKVGSTLTQAPFVAKDTVLMGCSGAELGVRGAVNAFDLKTGELKWRAFATGSDDSVRLAKDFNSANPHYGQFGLGTKTWEGDAWKIGGGTNWGWYAYDPKLNLFYYGSGNPAPWNETMRPGDNKWTMTIWGRDLDTGMAKWGYQKTPHDEWDFAGVNQMVLTDQPVNGKMTPLLSHIDRNGILYTLNRENGNLIVAEKVDPAVNVFKKVDLKTGTPVRDPEFATRMDHKGTNICPSAMGFHNQGVDSYDPESRTLYAGLNHICMDWEPFMLPYRAGQFFVGATLAMYPGPNGPTKKEMGQIRAFDLTTGKAKWTKWEKFAAWGGTLYTKGGLVWYATLDGYLKALDNKDGKELWNFKMPSGGIGSPMTYSFKGKQYIGSMYGVGGWPGVGLVFDLTDPSAGLGAVGAFRELQNHTQMGGGLMVFSL

>1WSRA

AQEVLRRTPLYDFHLAHGGKMVAFAGWSLPVQYRDSHTDSHLHTRQHCSLFDVSHMLQTKILGSDRVKLMESLVVGDIAELRPNQGTLSLFTNEAGGILDDLIVTNTSEGHLYVVSNAGCWEKDLALMQDKVRELQNQGRDVGLEVLDNALLALQGPTAAQVLQAGVADDLRKLPFMTSAVMEVFGVSGCRVTRCGYTGEDGVEISVPVAGAVHLATAILKNPEVKLAGLAARDSLRLEAGLCLYGNDIDEHTTPVEGSLSWTLGKRRRAAMDFPGAKVIVPQLKGRVQRRRVGLMCEGAPMRAHSPILNMEGTKIGTVTSGCPSPSLKKNVAMGYVPCEYSRPGTMLLVEVRRKQQMAVVSKMPFVPTNYYTLK

>1BCH1

AIEVKLANMEAEINTLKSKLELTNKLHAFSMGKKSGKKFFVTNHERMPFSKVKALCSELRGTVAIPRNAEENKAIQEVAKTSAFLGITDEVTEGQFMYVTGGRLTYSNWKKDQPDDWYGHGLGGGEDCVHIVDNGLWNDISCQASHTAVCEFPA

>1DXKA

SQKVEKTVIKNETGTISISQLNKNVWVHTELGSFNGEAVPSNGLVLNTSKGLVLVDSSWDDKLTKELIEMVEKKFQKRVTDVIITHAHADRIGGIKTLKERGIKAHSTALTAELAKKNGYEEPLGDLQTVTNLKFGNMKVETFYPGKGHTEDNIVVWLPQYNILVGGSLVKSTSAKDLGNVADAYVNEWSTSIENVLKRYRNINAVVPGHGEVGDKGLLLHTLDLLK

>3H8TA

DEPNQPSTPEAVTKTVTIDASKYETWQYFSFSKGEVVNVTDYKNDLNWDMALHRYDVRLNCGESGKGKGGAVFSGKTEMDQATTVPTDGYTVDVLGRITVKYEMGPDGHQMEYEEQGFSEVITGKKNAQGFASGGWLEFSHGPAGPTYKLSKRVFFVRGADGNIAKVQFTDYQDAELKKGVITFTYTYPVK

>3M7OA

MNGVAAALLVWILTSPSSSDHGSENGWPKHTACNSGGLEVVYQSCDPLQDFGLSIDQCSKQIQSNLNIRFGIILRQDIRKLFLDITLMAKGSSILNYSYPLCEEDQPKFSFCGRRKGEQIYYAGPVNNPGLDVPQGEYQLLLELYNENRATVACANATVTSS

>3OYYA

MASMKTAQEFRAGQVANINGAPWVIQKAEFNKSGRNAAVVKMKLKNLLTGAGTETVFKADDKLEPIILDRKEVTYSYFADPLYVFMDSEFNQYEIEKDDLEGVLTFIEDGMTDICEAVFYNDKVISVELPTTIVRQIAYTEPAVRGDTSGKVMKTARLNNGAELQVSAFCEIGDSIEIDTRTGEYKSRVKA

>1FAZA

APADKPQVLASFTQTSASSQNAWLAANRNQSAWAAYEFDWSTDLCTQAPDNPFGFPFNTACARHDFGYRNYKAAGSFDANKSRIDSAFYEDMKRVCTGYTGEKNTACNSTAWTYYQAVKIFG

>1ODZA

MRADVKPVTVKLVDSQATMETRSLFAFMQEQRRHSIMFGHQHETTQGLTITRTDGTQSDTFNAVGDFAAVYGWDTLSIVAPKAEGDIVAQVKKAYARGGIITVSSHFDNPKTDTQKGVWPVGTSWDQTPAVVDSLPGGAYNPVLNGYLDQVAEWANNLKDEQGRLIPVIFRLYHENTGSWFWWGDKQSTPEQYKQLFRYSVEYLRDVKGVRNFLYAYSPNNFWDVTEANYLERYPGDEWVDVLGFDTYGPVADNADWFRNVVANAALVARMAEARGKIPVISGIGIRAPDIEAGLYDNQWYRKLISGLKADPDAREIAFLLVWRNAPQGVPGPNGTQVPHYWVPANRPENINNGTLEDFQAFYADEFTAFNRDIEQVYQRPTLIVK

>1PF3A

AERPTLPIPDLLTTDARNRIQLTIGAGQSTFGGKTATTWGYNGNLLGPAVKLQRGKAVTVDIYNQLTEETTLHWHGLEVPGEVDGGPQGIIPPGGKRSVTLNVDQPAATCWFHPHQHGKTGRQVAMGLAGLVVIEDDEILKLMLPKQWGIDDVPVIVQDKKFSADGQIDYQLDVMTAAVGWFGDTLLTNGAIYPQHAAPRGWLRLRLLNGCNARSLNFATSDNRPLYVIASDGGLLPEPVKVSELPVLMGERFEVLVEVNDNKPFDLVTLPVSQMGMAIAPFDKPHPVMRIQPIAISASGALPDTLSSLPALPSLEGLTVRKLQLSMDPMLDMMGMQMLMEKYGDQAMAGMDHSQMMGHMGHGNMNHMNHGGKFDFHHANKINGQAFDMNKPMFAAAKGQYERWVISGVGDMLLHPFHIHGTQFRILSENGKPPAAHRAGWKDTVKVEGNVSEVLVKFNHDAPKEHAYMAHCHLLEHEDTGMMLGFTVSAWSHPNFEK

>1UWLA

MTDNNNYRDVEIRAPRGNKLTAKSWLTEAPLRMLMNNLDPQVAENPKELVVYGGIGRAARNWECYDKIVETLTRLEDDETLLVQSGKPVGVFKTHSNAPRVLIANSNLVPHWANWEHFNELDAKGLAMYGQMTAGSWIYIGSQGIVQGTYETFVEAGRQHYGGSLKGKWVLTAGLGGMGGAQPLAATLAGACSLNIESQQSRIDFRLETRYVDEQATDLDDALVRIAKYTAEGKAISIALHGNAAEILPELVKRGVRPDMVTDQTSAHDPLNGYLPAGWTWEQYRDRAQTEPAAVVKAAKQSMAVHVQAMLDFQKQGVPTFDYGNNIRQMAKEEGVANAFDFPGFVPAYIRPLFCRGVGPFRWAALSGEAEDIYKTDAKVKELIPDDAHLHRWLDMARERISFQGLPARICWVGLGLRAKLGLAFNEMVRSGELSAPVVIGRDHLDSGSVSSPNRETEAMRDGSDAVSDWPLLNALLNTAGGATWVSLHHGGGVGMGFSQHSGMVIVCDGTDEAAERIARVLTNDPGTGVMRHADAGYDIAIDCAKEQGLDLPMITG

>3MCXA

GDWLDLNTTSSVETGQAIVTLDDAQIALNGIYRLASGHSYYGDNYWYYGDCRAADVQARITKGDGKRVSPYYEYNVLASDNLNIVLPWNTVYKVIRQTNNLIQKIESGSIQSSDTKELNRIKSEALVMRGLSLFNLTRLFGMPYTNDKGASLGVPIETSPSDPTHKPSRSTVAQCYEQVVSDMSNALSGLRQETSNGYINYWAAQALLSRVYLNMGEYQKAYDAATDVIKNNGGRYQLYSYEEYPNVWGQDFQSESLFELYITLSEPSGGTGGEGAPMVYANEATVDWNNLILSEDFLNLLNEDPKDVRHCLTKESVIENNTGLPAAAMHEKVYLAKFPGKTGDDPKTNNICIIRLSEVYLNAAEAGLKKGTDIEEAQGYLNDIISRRTTDTSQQVSTETFTLDRILKERRKELVGEGEVFYDYLRNGLAIERKGSWHLETLKASNAQKIEATDLRIALPIPQSEIDANPNIQQNPR

>1Z82A

MGSDKIHHHHHHMEMRFFVLGAGSWGTVFAQMLHENGEEVILWARRKEIVDLINVSHTSPYVEESKITVRATNDLEEIKKEDILVIAIPVQYIREHLLRLPVKPSMVLNLSKGIEIKTGKRVSEIVEEILGCPYAVLSGPSHAEEVAKKLPTAVTLAGENSKELQKRISTEYFRVYTCEDVVGVEIAGALKNVIAIAAGILDGFGGWDNAKAALETRGIYEIARFGMFFGADQKTFMGLAGIGDLMVTCNSRYSRNRRFGELIARGFNPLKLLESSNQVVEGAFTVKAVMKIAKENKIDMPISEEVYRVVYEGKPPLQSMRDLMRRSLKDEFWAS

>3GZRA

GGEGTDAIQALIQAYFTAWNTNAPERFAEIFWPDGSWVNVVGMHWRGRDQIVFAHTAFLKTIFKDCKQELVTIEARTIAPGSALAVVTLIQDAYVTPDGRQMPRAHDRLTLLAVEREGVWRFIHGHNTIVNPDAANNDPVLRMKPA

>2A33A

MEIKGESMQKSKFRRICVFCGSSQGKKSSYQDAAVDLGNELVSRNIDLVYGGGSIGLMGLVSQAVHDGGRHVIGIIPKTLMPRELTGETVGEVRAVADMHQRKAEMAKHSDAFIALPGGYGTLEELLEVITWAQLGIHDKPVGLLNVDGYYNSLLSFIDKAVEEGFISPTAREIIVSAPTAKELVKKLEEYAPCHERVATKLCWEMERIGYSSEE

>1DHIA

MISLIAALAVDRVIGMENAMPWNLPASLAWFKRNTLDKPVIMGRHTWESIGRPLPGRKNIILSSQPGTDDRVTWVKSVDEAIAACGDVPEIMVIGGGRVYEQFLPKAQKLYLTHIDAEVEGDTHFPDYEPDDWESVFSEFHDADAQNSHSYCFEILERR

>3A8GA

MSVTIDHTTENAAPAQAPVSDRAWALFRALDGKGLVPDGYVEGWKKTFEEDFSPRRGAELVARAWTDPEFRQLLLTDGTAAVAQYGYLGPQGEYIVAVEDTPTLKNVIVCSLCACTAWPILGLPPTWYKSFEYRARVVREPRKVLSEMGTEIASDIEIRVYDTTAETRYMVLPQRPAGTEGWSQEQLQEIVTKDCLIGVAIPQVPTV

>1VDWA

MSVKTWRKIAIDIIRDFDHNIMPLFGNPKASETISISPSGDETKVVDKVAENIIISKFKDLGVNVVSEEIGRIDQGSDYTVVVDPLDGSYNFINGIPFFAVSVAIFHEKDPIYAFIYEPIVERLYEGIPGKGSYLNGEKIKVRELAEKPSISFYTKGKGTKIIDKVKRTRTLGAIALELAYLARGALDAVVDIRNYLRPTDIAAGVVIAREAGAIVKDLDGKDVEITFSATEKVNIIAANNEELLETILRSIEK

>2QKFA

MDIKINDITLGNNSPFVLFGGINVLESLDSTLQTCAHYVEVTRKLGIPYIFKASFDKANRSSIHSYRGVGLEEGLKIFEKVKAEFGIPVITDVHEPHQCQPVAEVCDVIQLPAFLARQTDLVVAMAKTGNVVNIKKPQFLSPSQMKNIVEKFHEAGNGKLILCERGSSFGYDNLVVDMLGFGVMKQTCGNLPVIFDVTHSLQTRDAGSAASGGRRAQALDLALAGMATRLAGLFLESHPDPKLAKCDGPSALPLHLLEDFLIRIKALDDLIKSQPILTIE

>2ZU1A

GPAFEFAVAMMKRNSSTVKTEYGEFTMLGIYDRWAVLPRHAKPGPTILMNDQEVGVLDAKELVDKDGTNLELTLLKLNRNEKFRDIRGFLAKEEVEVNEAVLAINTSKFPNMYIPVGQVTEYGFLNLGGTPTKRMLMYNFPTRAGQAGGVLMSTGKVLGIHVGGNGHQGFSAALLKHYFNDEQ

>2GJPA

HHMGTNGTMMQYFEWHLPNDGQHWNRLRDDASNLRNRGITAIWIPPAWKGTSQNDVGYGAYDLYDLGEFNQKGTVRTKYGTRSQLESAIHALKNNGVQVYGDVVMNHKGGADATENVLAVEVNPNNRNQEISGDYTIEAWTKFDFPGRGNTYSDFKWRWYHFDGVDWDQSRQFQNRIYKFRGDGKAWDWEVDSENGNYDYLMYADVDMDHPEVVNELRRWGEWYTNTLNLDGFRIDAVKHIKYSFTRDWLTHVRNATGKEMFAVAEFWKNDLGALENYLNKTNWNHSVFDVPLHYNLYNASNSGGNYDMAKLLNGTVVQKHPMHAVTFVDNHDSQPGESLESFVQEWFKPLAYALILTREQGYPSVFYGDYYGIPTHSVPAMKAKIDPILEARQNFAYGTQHDYFDHHNIIGWTREGNTTHPNSGLATIMSDGPGGEKWMYVGQNKAGQVWHDITGNKPGTVTINADGWANFSVNGGSVSIWVKR

>3B5NC

GSIKFTKQSSVASTRNTLKMAQDAERAGMNTLGMLGHQSEQLNNVEGNLDLMKVQNKVADEKVAELKKLQ

>1HQGA

MSSKPKPIEIIGAPFSKGQPRGGVEKGPAALRKAGLVEKLKETEYNVRDHGDLAFVDVPNDSPFQIVKNPRSVGKANEQLAAVVAETQKNGTISVVLGGDHSMAIGSISGHARVHPDLCVIWVDAHTDINTPLTTSSGNLCGQPVAFLLKELKGKFPDVPGFSWVTPCISAKDIVYIGLRDVDPGEHYIIKTLGIKYFSMTEVDKLGIGKVMEETFSYLLGRKKRPIHLSFDVDGLDPVFTPATGTPVVGGLSYREGLYITEEIYKTGLLSGLDIMEVNPTLGKTPEEVTRTVNTAVALTLSCFGTKREGNHKPETDYLKPPK

>2OQYA

MKITDLELHAVGIPRHTGFVNKHVIVKIHTDEGLTGIGEMSDFSHLPLYSVDLHDLKQGLLSILLGQNPFDLMKINKELTDNFPETMYYYEKGSFIRNGIDNALHDLCAKYLDISVSDFLGGRVKEKIKVCYPIFRHRFSEEVESNLDVVRQKLEQGFDVFRLYVGKNLDADEEFLSRVKEEFGSRVRIKSYDFSHLLNWKDAHRAIKRLTKYDLGLEMIESPAPRNDFDGLYQLRLKTDYPISEHVWSFKQQQEMIKKDAIDIFNISPVFIGGLTSAKKAAYAAEVASKDVVLGTTQELSVGTAAMAHLGCSLTNINHTSDPTGPELYVGDVVKNRVTYKDGYLYAPDRSVKGLGIELDESLLAKYQVPDLSWDNVTVHQLQDRTADTKS

>2OQAA

DVSFSLSGSSSTSYSKFIGALRKALPSNGTVYNITLLLSSASGASRYTLMKLSNYDGKAITVAIDVTNVYIMGYLVNSTSYFFNESDAKLASQYVFAGSTIVTLPYSGNYEKLQTAAGKIREKIPLGFPALDSAITTLFHYDSTAAAAAFLVIIQTTAESSRFKYIEGQIIMRISKNGVPSLATISLENEWSALSKQIQLAQTNNGTFKTPVVIMDAGGQRVEIGNVGSKVVTKNIQLLLN

>3QD5A

GPGSMAATPLPPLRLAIACDDAGVSYKEALKAHLSDNPLVSSITDVGVTSTTDKTAYPHVAIQAAQLIKDGKVDRALMICGTGLGVAISANKVPGIRAVTAHDTFSVERAILSNDAQVLCFGQRVIGIELAKRLAGEWLTYRFDQKSASAQKVQAISDYEKKFVEVN

>3D1RA

GMRRELAIEFSRVTESAALAGYKWLGRGDKNTADGAAVNAMRIMLNQVNIDGTIVIGEGEIAEAPMLYIGEKVGTGRGDAVDIAVDPIEGTRMTAMGQANALAVLAVGDKGCFLNAPDMYMEKLIVGPGAKGTIDLNLPLADNLRNVAAALGKPLSELTVTILAKPRHDAVIAEMQQLGVRVFAIPDGDVAASILTCMPDSEVDVLYGIGGAPEGVVSAAVIRALDGDMNGRLLARHDVKGDNEENRRIGEQELARCKAMGIEAGKVLRLGDMARSDNVIFSATGITKGDLLEGISRKGNIATTETLLIRGKSRTIRRIQSIHYLDRKDPEMQVHIL

>1RWIB

RPSWSPTQASGQTVLPFTGIDFRLSPSGVAVDSAGNVYVTSEGMYGRVVKLATGSTGTTVLPFNGLYQPQGLAVDGAGTVYVTDFNNRVVTLAAGSNNQTVLPFDGLNYPEGLAVDTQGAVYVADRGNNRVVKLAAGSKTQTVLPFTGLNDPDGVAVDNSGNVYVTDTDNNRVVKLEAESNNQVVLPFTDITAPWGIAVDEAGTVYVTEHNTNQVVKLLAGSTTSTVLPFTGLNTPLAVAVDSDRTVYVADRGNDRVVKLTSLEHHHHHH

>3NXDC

GSHMGGVEVLEVKTGVDSITEVECFLTPEMGDPDEHLRGFSKSISISDTFESDSPNRDMLPCYSVARIPLPNLNEDLTCGNILMWEAVTLKTEVIGVTSLMNVHSNGQATHDNGAGKPVQGTSFHFFSVGGEALELQGVLFNYRTKYPDGTIFPKNATVQSQVMNTEHKAYLDKNKAYPVECWVPDPTRNENTRYFGTLTGGENVPPVLHITNTATTVLLDEFGVGPLCKGDNLYLSAVDVCGMFTNRSGSQQWRGLSRYFKVQLRKRRVKN

>1TEJB

NSVNPCCDPQTCKPIEGKHCISGPCCENCYFLRSGTICQRARGDGNNDYCTGITPDCPRNRYNV

>3EWDA

MNILQEPIDFLKKEELKNIDLSQMSKKERYKIWKRIPKCELHCHLDLCFSADFFVSCIRKYNLQPNLSDEEVLDYYLFAKGGKSLGEFVEKAIKVADIFHDYEVIEDLAKHAVFNKYKEGVVLMEFRYSPTFVAFKYNLDIELIHQAIVKGIKEVVELLDHKIHVALMCIGTGHEAANIKASADFCLKHKADFVGFDHGGHEVDLKEYKEIFDYVRESGVPLSVHAGEDVTLPNLNTLYSAIQVLKVERIGHGIRVAESQELIDMVKEKNILLEVCPISNVLLKNAKSMDTHPIRQLYDAGVKVSVNSDDPGMFLTNINDDYEELYTHLNFTLEDFMKMNEWALEKSFMDSNIKDKIKNLYFKGEFEAYV

>1EW4A

MNDSEFHRLADQLWLTIEERLDDWDGDSDIDCEINGGVLTITFENGSKIIINRQEPLHQVWLATKQGGYHFDLKGDEWICDRSGETFWDLLEQAATQQAGETVSFR

>1H6WA

LSYPNATESVYGLTRYSTNDEAIAGVNNESSITPAKFTVALNNVFETRVSTESSNGVIKISSLPQALAGADDTTAMTPLKTQQLAVKLIAQIAPSKNAATESEQGVIQLATVAQARQGTLREGYAISPYTFMNSTATEEYKGVIKLGTQSEVNSNNASVAVTGATLNGRGSTTSMRGVVKLTTTAGSQSGGDASSALAWNADVIHQRGGQTINGTLRINNTLTIASGGANITGTVNMTGGYIQGKRVVTQNEIDRTIPVGAIMMWAADSLPSDAWRFCHGGTVSASDCPLYASRIGTRYGGSSSNPGLPDMR

>2CB9A

MARSQLSAAGEQHVIQLNQQGGKNLFCFPPISGFGIYFKDLALQLNHKAAVYGFHFIEEDSRIEQYVSRITEIQPEGPYVLLGYSAGGNLAFEVVQAMEQKGLEVSDFIIVDAYKKDQSITADTENDDSAAYLPEAVRETVMQKKRCYQEYWAQLINEGRIKSNIHFIEAGIQTETSGAMVLQKWQDAAEEGYAEYTGYGAHKDMLEGEFAEKNANIILNILDKINSDQKVLPNKHGSHHHHHH

>1GXJA

EKEMIERDMREYRGFSRAVRAVFEEKERFPGLVDVVSNLIEVDEKYSLAVSVLLGGTAQNIVVRNVDTAKAIVEFLKQNEAGRVTILPLDLIDGSFNRISGLENERGFVGYAVDLVKFPSDLEVLGGFLFGNSVVVETLDDAIRMKKKYRLNTRIATLDGELISGRGAITGGREERSSNVFERRIK

>1VLCA

MGSDKIHHHHHHMKIAVLPGDGIGPEVVREALKVLEVVEKKTGKTFEKVFGHIGGDAIDRFGEPLPEETKKICLEADAIFLGSVGGPKWDDLPPEKRPEIGGLLALRKMLNLYANIRPIKVYRSLVHVSPLKEKVIGSGVDLVTVRELSYGVYYGQPRGLDEEKGFDTMIYDRKTVERIARTAFEIAKNRRKKVTSVDKANVLYSSMLWRKVVNEVAREYPDVELTHIYVDNAAMQLILKPSQFDVILTTNMFGDILSDESAALPGSLGLLPSASFGDKNLYEPAGGSAPDIAGKNIANPIAQILSLAMMLEHSFGMVEEARKIERAVELVIEEGYRTRDIAEDPEKAVSTSQMGDLICKKLEEIW

>2VLBA

GQMQQASTPTIGMIVPPAAGLVPADGARLYPDLPFIASGLGLGSVTPEGYDAVIESVVDHARRLQKQGAAVVSLMGTSLSFYRGAAFNAALTVAMREATGLPCTTMSTAVLNGLRALGVRRVALATAYIDDVNERLAAFLAEESLVPTGCRSLGITGVEAMARVDTATLVDLCVRAFEAAPDSDGILLSCGGLLTLDAIPEVERRLGVPVVSSSPAGFWDAVRLAGGGAKARPGYGRLFDESGGSHHHHHH

>2VLPB

MESKRNKPGKATGKGKPVGDKWLDDAGKDSGAPIPDRIADKLRDKEFKSFDDFAKAVWEEVSKDPELSKNLNPSNKSSVSKGYSPFTPKNQQVGGRKVYELHHDKPISQGGEVYDMDNIRVTTPKRHIDIHRGK

>2Y88A

MPLILLPAVNVVEGRAVRLVQGKAGSQTEYGSAVDAALGWQRDGAEWIHLVDLDAAFGRGSNHELLAEVVGKLDVQVELSGGIRDDESLAAALATGCARVNVGTAALENPQWCARVIGEHGDQVAVGLDVQIIDGEHRLRGRGWETDGGDLWDVLERLDSEGCSRFVVTDITKDGTLGGPNLDLLAGVADRTDAPVIASGGVSSLDDLRAIATLTHRGVEGAIVGKALYARRFTLPQALAAVRD

>3DV9A

SNAMFKEAINNYLHTHGYESIDLKAVLFDMDGVLFDSMPNHAESWHKIMKRFGFGLSREEAYMHEGRTGASTINIVSRRERGHDATEEEIKAIYQAKTEEFNKCPKAERMPGALEVLTKIKSEGLTPMVVTGSGQTSLLDRLNHNFPGIFQANLMVTAFDVKYGKPNPEPYLMALKKGGFKPNEALVIENAPLGVQAGVAAGIFTIAVNTGPLHDNVLLNEGANLLFHSMPDFNKNWETLQSALKQD

>2QRJA

MGHHHHHHHHHHSSGHIEGRHMAAVTLHLRAETKPLEARAALTPTTVKKLIAKGFKIYVEDSPQSTFNINEYRQAGAIIVPAGSWKTAPRDRIIIGLKEMPETDTFPLVHEHIQFAHCYKDQAGWQNVLMRFIKGHGTLYDLEFLENDQGRRVAAFGFYAGFAGAALGVRDWAFKQTHSDDEDLPAVSPYPNEKALVKDVTKDYKEALATGARKPTVLIIGALGRCGSGAIDLLHKVGIPDANILKWDIKETSRGGPFDEIPQADIFINCIYLSKPIAPFTNMEKLNNPNRRLRTVVDVSADTTNPHNPIPIYTVATVFNKPTVLVPTTAGPKLSVISIDHLPSLLPREASEFFSHDLLPSLELLPQRKTAPVWVRAKKLFDRHCARVKRSSRL

>2O6XA

NDDLWHQWKRMYNKEYNGADDQHRRNIWEKNVKHIQEHNLRHDLGLVTYTLGLNQFTDMTFEEFKAKYLTEMSRASDILSHGVPYEANNRAVPDKIDWRESGYVTEVKDQGNCGSGWAFSTTGTMEGQYMKNERTSISFSEQQLVDCSRPWGNNGCGGGLMENAYQYLKQFGLETESSYPYTAVEGQCRYNKQLGVAKVTGFYTVHSGSEVELKNLVGAEGPAAVAVDVESDFMMYRSGIYQSQTCSPLRVNHAVLAVGYGTQGGTDYWIVKNSWGLSWGERGYIRMVRNRGNMCGIASLASLPMVARFP

>1GQGA

DTSSLIVEDAPDHVRPYVIRHYSHARAVTVDTQLYRFYVTGPSSGYAFTLMGTNAPHSDALGVLPHIHQKHYENFYCNKGSFQLWAQSGNETQQTRVLSSGDYGSVPRNVTHTFQIQDPDTEMTGVIVPGGFEDLFYYLGTNATDTTHTPYIPSSSDSSSTTGPDSSTISTLQSFDVYAELSFTPRTDTVNGTAPANTVWHTGANALASTAGDPYFIANGWGPKYLNSQYGYQIVAPFVTATQAQDTNYTLSTISMSTTPSTVTVPTWSFPGACAFQVQEGRVVVQIGDYAATELGSGDVAFIPGGVEFKYYSEAYFSKVLFVSSGSDGLDQNLVNGGEEWSSVSFPADW

>3MSUA

SNAMEVMLMSKYATLKYADKNIEIELPVYSPSLGNDCIDVSSLVKHGIFTYDPGFMSTAACESKITYIDGGKGVLLHRGYPIEEWTQKSNYRTLCYALIYGELPTDEQVKSFRQEIINKMPVCEHVKAAIAAMPQHTHPMSSLIAGVNVLAAEHIHNGQKESQDEVAKNIVAKIATIAAMAYRHNHGKKFLEPKMEYGYAENFLYMMFADDESYKPDELHIKAMDTIFMLHADHEQNASTSTVRLSGSTGNSPYAAIIAGITALWGPAHGGANEAVLKMLSEIGSTENIDKYIAKAKDKDDPFRLMGFGHRVYKNTDPRATAMKKNCEEILAKLGHSDNPLLTVAKKLEEIALQDEFFIERKLFSNVDFYSGIILKAMGIPEDMFTAIFALARTSGWISQWIEMVNDPAQKIGRPRQLYTGATNRNF

>1KTBA

LENGLARTPPMGWLAWERFRCNVNCREDPRQCISEMLFMEMADRIAEDGWRELGYKYINIDDCWAAKQRDAEGRLVPDPERFPRGIKALADYVHARGLKLGIYGDLGRLTCGGYPGTTLDRVEQDAQTFAEWGVDMLKLDGCYSSGKEQAQGYPQMARALNATGRPIVYSCSWPAYQGGLPPKVNYTLLGEICNLWRNYDDIQDSWDSVLSIVDWFFTNQDVLQPFAGPGHWNDPDMLIIGNFGLSYEQSRSQMALWTIMAAPLLMSTDLRTISPSAKKILQNRLMIQINQDPLGIQGRRIIKEGSHIEVFLRPLSQAASALVFFSRRTDMPFRYTTSLAKLGFPMGAAYEVQDVYSGKIISGLKTGDNFTVIINPSGVVMWYLCPKALLIQQQAPGGPSRLPLL

>2WGVA

MGSITENTSWNKEFSAEAVNGVFVLCKSSSKSCATNDLARASKEYLPASTFKIPNAIIGLETGVIKNEHQVFKWDGKPRAMKQWERDLTLRGAIQVSATPVFQQIAREVGEVRMQKYLKKFSYGNQNISGGIDKFWLEGQLRISAVNQVEFLESLYLNKLSASKENQLIVKEALVTEAAPEYLVHSKTGFSGVGTESNPGVAWWVGWVEKETEVYFFAFNMDIDNESKLPLRKSIPTKIMESEGIIGG

>3GBXA

SNAMLKREMNIADYDAELWQAMEQEKVRQEEHIELIASENYTSPRVMQAQGSQLTNKYAEGYPGKRYYGGCEYVDVVEQLAIDRAKELFGADYANVQPHSGSQANFAVYTALLQPGDTVLGMNLAQGGHLTHGSPVNFSGKLYNIVPYGIDESGKIDYDEMAKLAKEHKPKMIIGGFSAYSGVVDWAKMREIADSIGAYLFVDMAHVAGLIAAGVYPNPVPHAHVVTTTTHKTLAGPRGGLILAKGGDEELYKKLNSAVFPSAQGGPLMHVIAGKAVALKEAMEPEFKVYQQQVAKNAKAMVEVFLNRGYKVVSGGTENHLFLLDLVDKNLTGKEADAALGRANITVNKNSVPNDPKSPFVTSGIRIGSPAVTRRGFKEAEVKELAGWMCDVLDNINDEATIERVKAKVLDICARFPVYA

>1E6WA

AAAVRSVKGLVAVITGGASGLGLSTAKRLVGQGATAVLLDVPNSEGETEAKKLGGNCIFAPANVTSEKEVQAALTLAKEKFGRIDVAVNCAGIAVAIKTYHEKKNQVHTLEDFQRVINVNLIGTFNVIRLVAGVMGQNEPDQGGQRGVIINTASVAAFEGQVGQAAYSASKGGIVGMTLPIARDLAPIGIRVVTIAPGLFATPLLTTLPDKVRNFLASQVPFPSRLGDPAEYAHLVQMVIENPFLNGEVIRLDGAIRMQP

>1E6FA

MKSNEHDDCQVTNPSTGHLFDLSSLSGRAGFTAAYSEKGLVYMSICGENENCPPGVGACFGQTRISVGKANKRLRYVDQVLQLVYKDGSPCPSKSGLSYKSVISFVCRPEAGPTNRPMLISLDKQTCTLFFSWHTPLACEQAT

>3A75B

TTHFTVADRWGNVVSYTTTIEQLFGTGIMVPDYGVILNNELTDFDAIPGGANEVQPNKRPLSSMTPTILFKDDKPVLTVGSPGGATIISSVLQTILYHIEYGMELKAAVEEPRIYTNSMSSYRYEDGVPKDVLSKLNGMGHKFGTSPVDIGNVQSISIDHENGTFKGVADSSRNGAAIGINLKRK

>3FIRA

MKKIITLFGACALAFSMANADVNLYGPGGPHTALKDIANKYSEKTGVKVNVNFGPQATWFEKAKKDADILFGASDQSALAIASDFGKDFNVSKIKPLYFREAIILTQKGNPLKIKGLKDLANKKVRIVVPEGAGESNTSGTGVWEDMIGRTQDIKTIQNFRNNIVAFVPNSGSARKLFAQDQADAWITWIDWSKSNPDIGTAVAIEKDLVVYRTFNVIAKEGASKETQDFIAYLSSKEAKEIFKKYGWREH

>2IAPA

IPVIEPLFTKVTEDIPGAQGPVFDKNGDFYIVAPEVEVNGKPAGEILRIDLKTGKKTVICKPEVNGYGGIPAGCQCDRDANQLFVADMRLGLLVVQTDGTFEEIAKKDSEGRRMQGCNDCAFDYEGNLWITAPAGEVAPADYTRSMQEKFGSIYCFTTDGQMIQVDTAFQFPNGIAVRHMNDGRPYQLIVAETPTKKLWSYDIKGPAKIENKKVWGHIPGTHEGGADGMDFDEDNNLLVANWGSSHIEVFGPDGGQPKMRIRCPFEKPSNLHFKPQTKTIFVTEHENNAVWKFEWQRNGKKQYCETLKFGIF

>1Q74A

MSETPRLLFVHAHPDDESLSNGATIAHYTSRGAQVHVVTCTLGEEGEVIGDRWAQLTADHADQLGGYRIGELTAALRALGVSAPIYLGGAGRWRDSGMAGTDQRSQRRFVDADPRQTVGALVAIIRELRPHVVVTYDPNGGYGHPDHVHTHTVTTAAVAAAGVGSGTADHPGDPWTVPKFYWTVLGLSALISGARALVPDDLRPEWVLPRADEIAFGYSDDGIDAVVEADEQARAAKVAALAAHATQVVVGPTGRAAALSNNLALPILADEHYVLAGGSAGARDERGWETDLLAGLGFTASGT

>2CWLA

MFLRIDRLQIELPMPKEQDPNAAAAVQALLGGRFGEMSTLMNYMYQSFNFRGKKALKPYYDLIANIATEELGHIELVAATINSLLAKNPGKDLEEGVDPESAPLGFAKDVRNAAHFIAGGANSLVMGAMGEHWNGEYVFTSGNLILDLLHNFFLEVAARTHKLRVYEMTDNPVAREMIGYLLVRGGVHAAAYGKALESLTGVEMTKMLPIPKIDNSKIPEAKKYMDLGFHRNLYRFSPEDYRDLGLIWKGASPEDGTEVVVVDGPPTGGPVFDAGHDAAEFAPEFHPGELYEIAKKLYEKAK

>3NK6A

SEFMTEPAIITNASDPAVQRIIDVTKHSRASIKTTLIEDTEPLMECIRAGVQFIEVYGSSGTPLDPALLDLCRQREIPVRLIDVSIVNQLFKAERKAKVFGIARVPRPARLADIAERGGDVVVLDGVKIVGNIGAIVRTSLALGAAGIVLVDSDLATIADRRLLRASRGYVFSLPVVLADREEAVSFLRDNDIALMVLDTDGDLGVKDLGDRADRMALVFGSEKGGPSGLFQEASAGTVSIPMLSSTESLNVSVSVGIALHERSARNFAVRRAAAQA

>1V3IA

ATSDSNMLLNYVPVYVMLPLGVVNVDNVFEDPDGLKEQLLQLRAAGVDGVMVDVWWGIIELKGPKQYDWRAYRSLLQLVQECGLTLQAIMSFHQCGGNVGDIVNIPIPQWVLDIGESNHDIFYTNRSGTRNKEYLTVGVDNEPIFHGRTAIEIYSDYMKSFRENMSDFLESGLIIDIEVGLGPAGELRYPSYPQSQGWEFPGIGEFQCYDKYLKADFKAAVARAGHPEWELPDDAGKYNDVPESTGFFKSNGTYVTEKGKFFLTWYSNKLLNHGDQILDEANKAFLGCKVKLAIKVSGIHWWYKVENHAAELTAGYYNLNDRDGYRPIARMLSRHHAILNFTCLEMRDSEQPSDAKSGPQELVQQVLSGGWREDIRVAGQNALPRYDATAYNQIILNARPQGVNNNGPPKLSMFGVTYLRLSDDLLQKSNFNIFKKFVLKMHADQDYCANPQKYNHAITPLKPSAPKIPIEVLLEATKPTLPFPWLPETDMKVDG

>2W87A

ALLLQEAQAGFCRVDGTIDNNHTGFTGSGFANTNNAQGAAVVWAIDATSSGRRTLTIRYANGGTANRNGSLVINGGSNGNYTVSLPTTGAWTTWQTATIDVDLVQGNNIVQLSATTAEGLPNIDSLSVVGGTVRAGNCG

>2W8TA

TEAAAQPHALPADAPDIAPERDLLSKFDGLIAERQKLLDSGVTDPFAIVMEQVKSPTEAVIRGKDTILLGTYNYMGMTFDPDVIAAGKEALEKFGSGTCGSRMLNGTFHDHMEVEQALRDFYGTTGAIVFSTGYMANLGIISTLAGKGEYVILDADSHASIYDGCQQGNAEIVRFRHNSVEDLDKRLGRLPKEPAKLVVLEGVYSMLGDIAPLKEMVAVAKKHGAMVLVDEAHSMGFFGPNGRGVYEAQGLEGQIDFVVGTFSKSVGTVGGFVVSNHPKFEAVRLACRPYIFTASLPPSVVATATTSIRKLMTAHEKRERLWSNARALHGGLKAMGFRLGTETCDSAIVAVMLEDQEQAAMMWQALLDGGLYVNMARPPATPAGTFLLRCSICAEHTPAQIQTVLGMFQAAGRAVGVIGLEHHHHHH

>1Y0BA

SNAMEALKRKIEEEGVVLSDQVLKVDSFLNHQIDPLLMQRIGDEFASRFAKDGITKIVTIESSGIAPAVMTGLKLGVPVVFARKHKSLTLTDNLLTASVYSFTKQTESQIAVSGTHLSDQDHVLIIDDFLANGQAAHGLVSIVKQAGASIAGIGIVIEKSFQPGRDELVKLGYRVESLARIQSLEEGKVSFVQEVHS

>3K3KA

MGSSHHHHHHSSGLVPRGSHMPSELTPEERSELKNSIAEFHTYQLDPGSCSSLHAQRIHAPPELVWSIVRRFDKPQTYKHFIKSCSVEQNFEMRVGCTRDVIVISGLPANTSTERLDILDDERRVTGFSIIGGEHRLTNYKSVTTVHRFEKENRIWTVVLESYVVDMPEGNSEDDTRMFADTVVKLNLQKLATVAEAMARNSGDGSGSQVT

>3LIDA

MEKYQALLANNVENTAKEALHQLAYTGREYNNIQDQIETISDLLGHSQSLYDYLREPSKANLTILENMWSSVARNQKLYKQIRFLDTSGTEKVRIKYDFKTSIAGPSLILRDKSAREYFKYAQSLDNEQISAWGIELERDKGELVYPLSPSLRILMPISVNDVRQGYLVLNVDIEYLSSLLNYSPVRDFHIELVKHKGFYIASPDESRLYGDIIPERSQFNFSNMYPDIWPRVVSEQAGYSYSGEHLIAFSSIKFVSNEPLHLIIDLSNEQLSKRATRDINDLIQESLEHHHHHH

>1PGTA

MPPYTVVYFPVRGRCAALRMLLADQGQSWKEEVVTVETWQEGSLKASCLYGQLPKFQDGDLTLYQSNTILRHLGRTLGLYGKDQQEAALVDMVNDGVEDLRCKYVSLIYTNYEAGKDDYVKALPGQLKPFETLLSQNQGGKTFIVGDQISFADYNLLDLLLIHEVLAPGCLDAFPLLSAYVGRLSARPKLKAFLASPEYVNLPINGNGKQ

>1ZLHB

NECVSKGFGCLPQSDCPQEARLSYGGCSTVCCDLSKLTGCKGKGGECNPLDRQCKELQAESASCGKGQKCCVWLH

>3CIFA

MGSSHHHHHHSSGLVPRGSHMTATLGINGFGRIGRLVLRACMERNDITVVAINDPFMDVEYMAYLLKYDSVHGNFNGTVEVSGKDLCINGKVVKVFQAKDPAEIPWGASGAQIVCESTGVFTTEEKASLHLKGGAKKVIISAPPKDNVPMYVMGVNNTEYDPSKFNVISNASSTTNCLAPLAKIINDKFGIVEGLMTTVHSLTANQLTVDGPSKGGKDWRAGRCAGNNIIPASTGAAKAVGKVIPALNGKLTGMAIRVPTPDVSVVDLTCKLAKPASIEEIYQAVKEASNGPMKGIMGYTSDDVVSTDFIGCKYSSILDKNACIALNDSFVKLISWYDNESGYSNRLVDLAVYVASRGL

>2JINA

SMRVDYLVTEEEINLTRGPSGLGFNIVGGTDQQYVSNDSGIYVSRIKENGAAALDGRLQEGDKILSVNGQDLKNLLHQDAVDLFRNAGYAVSLRVQHRESSI

>2RK6A

GSHMASKRALVILAKGAEEMETVIPVDVMRRAGIKVTVAGLAGKDPVQCSRDVVICPDASLEDAKKEGPYDVVVLPGGNLGAQNLSESAAVKEILKEQENRKGLIAAICAGPTALLAHEIGFGSKVTTHPLAKDKMMNGGHYTYSENRVEKDGLILTSRGPGTSFKFALAIVEALNGKEVAAQVKAPLVLKD

>2GAIA

MAKKVKKYIVVESPAKAKTIKSILGNEYEVFASMGHIIDLPKSKFGVDLEKDFEPEFAVIKGKEKVVEKLKDLAKKGELLIASDMDREGEAIAWHIARVTNTLGRKNRIVFSEITPRVIREAVKNPREIDMKKVRAQLARRILDRIVGYSLSPVLWRNFKSNLSAGRVQSATLKLVCDREREILRFVPKKYHRITVNFDGLTAEIDVKEKKFFDAETLKEIQSIDELVVEEKKVSVKKFAPPEPFKTSTLQQEAYSKLGFSVSKTMMIAQQLYEGVETKDGHIAFITYMRTDSTRVSDYAKEEARNLITEVFGEEYVGSKRERRKSNAKIQDAHEAIRPTNVFMTPEEAGKYLNSDQKKLYELIWKRFLASQMKPSQYEETRFVLRTKDGKYRFKGTVLKKIFDGYEKVWKTERNTGEFPFEEGESVKPVVVKIEEQETKPKPRYTEGSLVKEMERLGIGRPSTYASTIKLLLNRGYIKKIRGYLYPTIVGSVVMDYLEKKYSDVVSVSFTAEMEKDLDEVEQGKKTDKIVLREFYESFSSVFDRNDRIVVDFPTNQKCSCGKEMRLSFGKYGFYLKCECGKTRSVKNDEIAVIDDGKIFLGRKDSESGSPDGRSVEGKGNLSEKRRKGKKGS

>2GAKA

PEFFSVRHLELAGDDPYSNVNCTKILQGDPEEIQKVKLEILTVQFKKRPRWTPHDYINMTRDCASFIRTRKYIVEPLTKEEVGFPIAYSIVVHHKIEMLDRLLRAIYMPQNFYCIHVDRKAEESFLAAVQGIASCFDNVFVASQLESVVYASWTRVKADLNCMKDLYRMNANWKYLINLCGMDFPIKTNLEIVRKLKCSTGENNLETEKMPPNKEERWKKRYAVVDGKLTNTGIVKAPPPLKTPLFSGSAYFVVTREYVGYVLENENIQKLMEWAQDTYSPDEFLWATIQRIPEVPGSFPSSNKYDLSDMNAIARFVKWQYFEGDVSNGAPYPPCSGVHVRSVCVFGAGDLSWMLRQHHLFANKFDMDVDPFAIQCLDEHLRRKALENLEH

>2RLCA

CTGLALETKDGLHLFGRNMDIEYSFNQSIIFIPRNFKCVNKSNKKELTTKYAVLGMGTIFDDYPTFADGMNEKGLGCAGLNFPVYVSYSKEDIEGKTNIPVYNFLLWVLANFSSVEEVKEALKNANIVDIPISENIPNTTLHWMISDITGKSIVVEQTKEKLNVFDNNIGVLTNSPTFDWHVANLNQYVGLRYNQVPEFKLGDQSLTALGQGTGLVGLPGDFTPASRFIRVAFLRDAMIKNDKDSIDLIEFFHILNNVAMVRGSTRTVEEKSDLTQYTSCMCLEKGIYYYNTYENNQINAIDMNKENLDGNEIKTYKYNKTLSINHVN

>3DR3A

SNAMLNTLIVGASGYAGAELVTYVNRHPHMNITALTVSAQSNDAGKLISDLHPQLKGIVELPLQPMSDISEFSPGVDVVFLATAHEVSHDLAPQFLEAGCVVFDLSGAFRVNDATFYEKYYGFTHQYPELLEQAAYGLAEWCGNKLKEANLIAVPGCYPTAAQLALKPLIDADLLDLNQWPVINATSGVSGAGRKAAISNSFCEVSLQPYGVFTHRHQPEIATHLGADVIFTPHLGNFPRGILETITCRLKSGVTQAQVAQALQQAYAHKPLVRLYDKGVPALKNVVGLPFCDIGFAVQGEHLIIVATEDNLLKGAAAQAVQCANIRFGYAETQSLI

>2CN3A

ISSQAVTSVPYKWDNVVIGGGGGFMPGIVFNETEKDLIYARAAIGGAYRWDPSTETWIPLLDHFQMDEYSYYGVESIATDPVDPNRVYIVAGMYTNDWLPNMGAILRSTDRGETWEKTILPFKMGGNMPGRSMGERLAIDPNDNRILYLGTRCGNGLWRSTDYGVTWSKVESFPNPGTYIYDPNFDYTKDIIGVVWVVFDKSSSTPGNPTKTIYVGVADKNESIYRSTDGGVTWKAVPGQPKGLLPHHGVLASNGMLYITYGDTCGPYDGNGKGQVWKFNTRTGEWIDITPIPYSSSDNRFCFAGLAVDRQNPDIIMVTSMNAWWPDEYIFRSTDGGATWKNIWEWGMYPERILHYEIDISAAPWLDWGTEKQLPEINPKLGWMIGDIEIDPFNSDRMMYVTGATIYGCDNLTDWDRGGKVKIEVKATGIEECAVLDLVSPPEGAPLVSAVGDLVGFVHDDLKVGPKKMHVPSYSSGTGIDYAELVPNFMALVAKADLYDVKKISFSYDGGRNWFQPPNEAPNSVGGGSVAVAADAKSVIWTPENASPAVTTDNGNSWKVCTNLGMGAVVASDRVNGKKFYAFYNGKFYISTDGGLTFTDTKAPQLPKSVNKIKAVPGKEGHVWLAAREGGLWRSTDGGYTFEKLSNVDTAHVVGFGKAAPGQDYMAIYITGKIDNVLGFFRSDDAGKTWVRINDDEHGYGAVDTAITGDPRVYGRVYIATNGRGIVYGEPASDEPV

>1XA3A

MHHHHHHGSTSLYKKAGSETLYIQGDHLPMPKFGPLAGLRVVFSGIEIAGPFAGQMFAEWGAEVIWIENVAWADTIRVQPNYPQLSRRNLHALSLNIFKDEGREAFLKLMETTDIFIEASKGPAFARRGITDEVLWQHNPKLVIAHLSGFGQYGTEEYTNLPAYNTIAQAFSGYLIQNGDVDQPMPAFPYTADYFSGLTATTAALAALHKVRETGKGESIDIAMYEVMLRMGQYFMMDYFNGGEMCPRMSKGKDPYYAGCGLYKCADGYIVMELVGITQIEECFKDIGLAHLLGTPEIPEGTQLIHRIECPYGPLVEEKLDAWLATHTIAEVKERFAELNIACAKVLTVPELESNPQYVARESITQWQTMDGRTCKGPNIMPKFKNNPGQIWRGMPSHGMDTAAILKNIGYSENDIQELVSKGLAKVEDSTHHHHHH

>1GPIA

QQAGTNTAENHPQLQSQQCTTSGGCKPLSTKVVLDSNWRWVHSTSGYTNCYTGNEWDTSLCPDGKTCAANCALDGADYSGTYGITSTGTALTLKFVTGSNVGSRVYLMADDTHYQLLKLLNQEFTFDVDMSNLPCGLNGALYLSAMDADGGMSKYPGNKAGAKYGTGYCDSQCPKDIKFINGEANVGNWTETGSNTGTGSYGTCCSEMDIWEANNDAAAFTPHPCTTTGQTRCSGDDCARNTGLCDGDGCDFNSFRMGDKTFLGKGMTVDTSKPFTVVTQFLTNDNTSTGTLSEIRRIYIQNGKVIQNSVANIPGVDPVNSITDNFCAQQKTAFGDTNWFAQKGGLKQMGEALGNGMVLALSIWDDHAANMLWLDSDYPTDKDPSAPGVARGTCATTSGVPSDVESQVPNSQVVFSNIKFGDIGSTFSGTS

>1CMNA

ASGAPSFPFSRASGPEPPAEFAKLRATNPVSQVKLFDGSLAWLVTKHKDVCFVATSEKLSKVRTRQGFPELSASGKQAAKAKPTFVDMDPPEHMHQRSMVEPTFTPEAVKNLQPYIQRTVDDLLEQMKQKGCANGPVDLVKEFALPVPSYIIYTLLGVPFNDLEYLTQQNAIRTNGSSTAREASAANQELLDYLAILVEQRLVEPKDDIISKLCTEQVKPGNIDKSDAVQIAFLLLVAGNATMVNMIALGVATLAQHPDQLAQLKANPSLAPQFVEELCRYHTAVALAIKRTAKEDVMIGDKLVRANEGIIASNQSANRDEEVFENPDEFNMNRKWPPQDPLGFGFGDHRCIAEHLAKAELTTVFSTLYQKFPDLKVAVPLGKINYTPLNRDVGIVDLPVIF

>1LRIA

TACTASQQTAAYKTLVSILSDASFNQCSTDSGYSMLTAKALPTTAQYKLMCASTACNTMIKKIVTLNPPNCDLTVPTSGLVLNVYSYANGFSNKCSSL

>2QECA

GMSPTVLPATQADFPKIVDVLVEAFANDPTFLRWIPQPDPGSAKLRALFELQIEKQYAVAGNIDVARDSEGEIVGVALWDRPDGNHSAKDQAAMLPRLVSIFGIKAAQVAWTDLSSARFHPKFPHWYLYTVATSSSARGTGVGSALLNHGIARAGDEAIYLEATSTRAAQLYNRLGFVPLGYIPSDDDGTPELAMWKPPAMPTV

>1VAJA

MVFKIKDEWGEFLVRLARRAIEEYLKTGKEIEPPKDTPPELWEKMGVFVTLNRYNVPPQTALRGCIGFPTPIYPLVEATIKAAIYSAVDDPRFPPVKLEEMDNLVVEVSVLTPPELIEGPPEERPRKIKVGRDGLIVEKGIYSGLLLPQVPVEWGWDEEEFLAETCWKAGLPPDCWLDEDTKVYKFTAEIFEEEYPRGPIKRKPLVLEHHHHHH

>1D7EA

VAFGSEDIENTLAKMDDGQLDGLAFGAIQLDGDGNILQYNAAEGDITGRDPKQVIGKNFFKDVAPCTDSPEFYGKFKEGVASGNLNTMFEYTFDYQMTPTKVKVHMKKALSGDSYWVFVKRV

>1YNVX

DVSGTVCLSALPPEATDTLNLIASDGPFPYSQDGVVFQNRESVLPTQSYGYYHEYTVITPGARTRGTRRIITGEATQEDYYTGDHYATFSLIDKTC

>1XH9A

GNAAAAKKGSEQESVKEFLAKAKEDFLKKWENPAQNTAHLDQFERIKTLGTGSFGRVMLVKHMETGNHYAMKILDKQKVVKLKEIEHTLNEKRILQAVNFPFLVKLEFSFKDNSNLYMVMEYAPGGEMFSHLRRIGRFSEPHARFYAAQIVLTFEYLHSLDLIYRDLKPENLMIDQQGYIKVTDFGLAKRVKGRTWTLCGTPEYLAPEIILSKGYNKAVDWWALGVLIYEMAAGYPPFFADQPIQIYEKIVSGKVRFPSHFSSDLKDLLRNLLQVDLTKRFGNLKNGVNDIKNHKWFATTDWIAIYQRKVEAPFIPKFKGPGDTSNFDDYEEEEIRVSINEKCGKEFSEF

>3OIRA

SNADGLEGMDDPDATSKKVVPLGVEIYEINGPFFFGVADRLKGVLDVIEETPKVFILRMRRVPVIDATGMHALWEFQESCEKRGTILLLSGVSDRLYGALNRFGFIEALGEERVFDHIDKALAYAKLLVETAEER

>2V4CA

ADYDLKFGMNAGTSSNEYKAAEMFAKEVKEKSQGKIEISLYPSSQLGDDRAMLKQLKDGSLDFTFAESARFQLFYPEAAVFALPYVISNYNVAQKALFDTEFGKDLIKKMDKDLGVTLLSQAYNGTRQTTSNRAINSIADMKGLKLRVPNAATNLAYAKYVGASPTPMAFSEVYLALQTNAVDGQENPLAAVQAQKFYEVQKFLAMTNHILNDQLYLVSNETYKELPEDLQKVVKDAAENAAKYHTKLFVDGEKDLVTFFEKQGVKITHPDLVPFKESMKPYYAEFVKQTGQKGESALKQIEAINPHHH

>1JNRA

MVYYPKKYELYKADEVPTEVVETDILIIGGGFSGCGAAYEAAYWAKLGGLKVTLVEKAAVERSGAVAQGLSAINTYIDLTGRSERQNTLEDYVRYVTLDMMGLAREDLVADYARHVDGTVHLFEKWGLPIWKTPDGKYVREGQWQIMIHGESYKPIIAEAAKMAVGEENIYERVFIFELLKDNNDPNAVAGAVGFSVREPKFYVFKAKAVILATGGATLLFRPRSTGEAAGRTWYAIFDTGSGYYMGLKAGAMLTQFEHRFIPFRFKDGYGPVGAWFLFFKCKAKNAYGEEYIKTRAAELEKYKPYGAAQPIPTPLRNHQVMLEIMDGNQPIYMHTEEALAELAGGDKKKLKHIYEEAFEDFLDMTVSQALLWACQNIDPQEQPSEAAPAEPYIMGSHSGEAGFWVCGPEDLMPEEYAKLFPLKYNRMTTVKGLFAIGDCAGANPHKFSSGSFTEGRIAAKAAVRFILEQKPNPEIDDAVVEELKKKAYAPMERFMQYKDLSTADDVNPEYILPWQGLVRLQKIMDEYAAGIATIYKTNEKMLQRALELLAFLKEDLEKLAARDLHELMRAWELVHRVWTAEAHVRHMLFRKETRWPGYYYRTDYPELNDEEWKCFVCSKYDAEKDEWTFEKVPYVQVIEWSF

>2QM0A

SNAMNTTVEKQQIITSNTEQWKMYSKLEGKEYQIHISKPKQPAPDSGYPVIYVLDGNAFFQTFHEAVKIQSVRAEKTGVSPAIIVGVGYPIEGAFSGEERCYDFTPSVISKDAPLKPDGKPWPKTGGAHNFFTFIEEELKPQIEKNFEIDKGKQTLFGHXLGGLFALHILFTNLNAFQNYFISSPSIWWNNKSVLEKEENLIIELNNAKFETGVFLTVGSLEREHMVVGANELSERLLQVNHDKLKFKFYEAEGENHASVVPTSLSKGLRFISYV

>2QM6A

MGSSHHHHHHSSGLVPRGSHMASAASYPPIKNTKVGLALSSHPLASEIGQKVLEEGGNAIDAAVAIGFALAVVHPAAGNIGGGGFAVIHLANGENVALDFREKAPLKATKNMFLDKQGNVVPKLSEDGYLAAGVPGTVAGMEAMLKKYGTKKLSQLIDPAIKLAENGYAISQRQAETLKEARERFLKYSSSKKYFFKKGHLDYQEGDLFVQKDLAKTLNQIKTLGAKGFYQGQVAELIEKDMKKNGGIITKEDLASYNVKWRKPVVGSYRGYKIISMSPPSSGGTHLIQILNVMENADLSALGYGASKNIHIAAEAMRQAYADRSVYMGDADFVSVPVDKLINKAYAKKIFDTIQPDTVTPSSQIKPGMGQLHEGSN

>3GHJA

MGSSHHHHHHSSGRENLYFQGVPMNIKGLFEVAVKVKNLEKSSQFYTEILGFEAGLLDSARRWNFLWVSGRAGMVVLQEEKENWQQQHFSFRVEKSEIEPLKKALESKGVSVHGPVNQEWMQAVSLYFADPNGHALEFTAL

>2Z58B

NTIRVIVSVDKAKFNPHEVLGIGGHIVYQFKLIPAVVVDVPANAVGKLKKMPWVEKVEFDHQAVLL

>1CUHA

LPTSNPAQELEARQLGRTTRDDLINGNSASCADVIFIYARGSTETGNLGTLGPSIASNLESAFGKDGVWIQGVGGAYRATLGDNALPRGTSSAAIREMLGLFQQANTKCPDATLIAGGYSQGAALAAASIEDLDSAIRDKIAGTVLFGYTKNLQNRGRIPNYPADRTKVFCNTGDLVCTGSLIVAAPHLAYGPDAEGPAPEFLIEKVRAVRGSA

>1OJQA

AETKNFTDLVEATKWGNSLIKSAKYSSKDKMAIYNYTKNSSPINTPLRSANGDVNKLSENIQEQVRQLDSTISKSVTPDSVYVYRLLNLDYLSSITGFTREDLHMLQQTNNGQYNEALVSKLNNLMNSRIYRENGYSSTQLVSGAALAGRPIELKLELPKGTKAAYIDSKELTAYPGQQEVLLPRGTEYAVGSVKLSDNKRKIIITAVVFKK

>1NIJA

MNPIAVTLLTGFLGAGKTTLLRHILNEQHGYKIAVIENEFGEVSVDDQLIGDRATQIKTLTNGCICCSRSNELEDALLDLLDNLDKGNIQFDRLVIECTGMADPGPIIQTFFSHEVLCQRYLLDGVIALVDAVHADEQMNQFTIAQSQVGYADRILLTKTDVAGEAEKLHERLARINARAPVYTVTHGDIDLGLLFNTNGFMLEENVVSTKPRFHFIADKQNDISSIVVELDYPVDISEVSRVMENLLLESADKLLRYKGMLWIDGEPNRLLFQGVQRLYSADWDRPWGDEKPHSTMVFIGIQLPEEEIRAAFAGLRK

>2O2GA

GMDRTLTHQPQEYAVSVSVGEVKLKGNLVIPNGATGIVLFAHGSGSSRYSPRNRYVAEVLQQAGLATLLIDLLTQEEEEIDLRTRHLRFDIGLLASRLVGATDWLTHNPDTQHLKVGYFGASTGGGAALVAAAERPETVQAVVSRGGRPDLAPSALPHVKAPTLLIVGGYDLPVIAMNEDALEQLQTSKRLVIIPRASHLFEEPGALTAVAQLASEWFMHYLR

>1O2DA

MGSDKIHHHHHHVWEFYMPTDVFFGEKILEKRGNIIDLLGKRALVVTGKSSSKKNGSLDDLKKLLDETEISYEIFDEVEENPSFDNVMKAVERYRNDSFDFVVGLGGGSPMDFAKAVAVLLKEKDLSVEDLYDREKVKHWLPVVEIPTTAGTGSEVTPYSILTDPEGNKRGCTLMFPVYAFLDPRYTYSMSDELTLSTGVDALSHAVEGYLSRKSTPPSDALAIEAMKIIHRNLPKAIEGNREARKKMFVASCLAGMVIAQTGTTLAHALGYPLTTEKGIKHGKATGMVLPFVMEVMKEEIPEKVDTVNHIFGGSLLKFLKELGLYEKVAVSSEELEKWVEKGSRAKHLKNTPGTFTPEKIRNIYREALGV

>3MR1A

GPGSMIKIHTEKDFIKMRAAGKLAAETLDFITDHVKPNVTTNSLNDLCHNFITSHNAIPAPLNYKGFPKSICTSINHVVCHGIPNDKPLKNGDIVNIDVTVILDGWYGDTSRMYYVGDVAIKPKRLIQVTYDAMMKGIEVVRPGAKLGDIGYAIQSYAEKHNYSVVRDYTGHGIGRVFHDKPSILNYGRNGTGLTLKEGMFFTVEPMINAGNYDTILSKLDGWTVTTRDKSLSAQFEHTIGVTKDGFEIFTL

>3NJCA

MGHHHHHHSHMKSKFEASIDNLKEIEMNAYAYELIREIVLPDMLGQDYSSMMYWAGKHLARKFPLESWEEFPAFFEEAGWGTLTNVSAKKQELEFELEGPIISNRLKHQKEPCFQLEAGFIAEQIQLMNDQIAESYEQVKKRADKVVLTVKWDMKDPV

>3R0NA

MQDVRVQVLPEVRGQLGGTVELPCHLLPPVPGLYISLVTWQRPDAPANHQNVAAFHPKMGPSFPSPKPGSERLSFVSAKQSTGQDTEAELQDATLALHGLTVEDEGNYTCEFATFPKGSVRGMTWLRV

>1FPZA

MKPPSSIQTSEFDSSDEEPIEDEQTPIHISWLSLSRVNCSQFLGLCALPGCKFKDVRRNVQKDTEELKSCGIQDIFVFCTRGELSKYRVPNLLDLYQQCGIITHHHPIADGGTPDIASCCEIMEELTTCLKNYRKTLIHSYGGLGRSCLVAACLLLYLSDTISPEQAIDSLRDLRGSGAIQTIKQYNYLHEFRDKLAAHLSSRDSQSRSVSR

>3LX4A

MGSSHHHHHHSQDPNSAAPAAEAPLSHVQQALAELAKPKDDPTRKHVCVQVAPAVRVAIAETLGLAPGATTPKQLAEGLRRLGFDEVFDTLFGADLTIMEEGSELLHRLTEHLEAHPHSDEPLPMFTSCCPGWIAMLEKSYPDLIPYVSSCKSPQMMLAAMVKSYLAEKKGIAPKDMVMVSIMPCTRKQSEADRDWFCVDADPTLRQLDHVITTVELGNIFKERGINLAELPEGEWDNPMGVGSGAGVLFGTTGGVMEAALRTAYELFTGTPLPRLSLSEVRGMDGIKETNITMVPAPGSKFEELLKHRAAARAEAAAHGTPGPLAWDGGAGFTSEDGRGGITLRVAVANGLGNAKKLITKMQAGEAKYDFVEIMACPAGCVGGGGQPRSTDKAITQKRQAALYNLDEKSTLRRSHENPSIRELYDTYLGEPLGHKAHELLHTHYVAGGVEEKDEKK

>1SCTB

SKVAELANAVVSNADQKDLLRMSWGVLSVDMEGTGLMLMANLFKTSPSAKGKFARLGDVSAGKDNSKLRGHSITLMYALQNFVDALDDVERLKCVVEKFAVNHINRQISADEFGEIVGPLRQTLKARMGNYFDEDTVAAWASLVAVVQASL

>3M1MA

SSNFSSERIRYAKWFLEHGFNIIPIDPESKKPVLKEWQKYSHEMPSDEEKQRFLKMIEEGYNYAIPGGQKGLVILDFESKEKLKAWIGESALEELCRKTLCTNTVHGGIHIYVLSNDIPPHKINPLFEENGKGIIDLQSYNSYVLGLGSCVNHLHCTTDKCPWKEQNYTTCYTLYNELKEISKVDLKSLLRFLAEKGKRLGITLSKTAKEWLEGKKEEEDTVVEFEELRKELVKRDSGKPVEKIKEEICTKSPPKLIKEIICENKTYADVNIDRSRGDWHVILYLMKHGVTDPDKILELLPRDSKAKENEKWNTQKYFVITLSKAWSVVKKYLEA

>3M12A

STHFDVIVVGAGSMGMAAGYQLAKQGVKTLLVDAFDPPHTNGSHHGDTRIIRHAYGEGREYVPLALRSQELWYELEKETHHKIFTKTGVLVFGPKGESAFVAETMEAAKEHSLTVDLLEGDEINKRWPGITVPENYNAIFEPNSGVLFSENCIRAYRELAEARGAKVLTHTRVEDFDISPDSVKIETANGSYTADKLIVSMGAWNSKLLSKLNLDIPLQPYRQVVGFFESDESKYSNDIDFPGFMVEVPNGIYYGFPSFGGCGLRLGYHTFGQKIDPDTINREFGVYPEDESNLRAFLEEYMPGANGELKRGAVCMYTKTLDEHFIIDLHPEHSNVVIAAGFSGHGFKFSSGVGEVLSQLALTGKTEHDISIFSINRPALKESLQKTTI

>3BDZA

TSLFTTADHYHTPLGPDGTPHAFFEALRDEAETTPIGWSEAYGGHWVVAGYKEIQAVIQNTKAFSNKGVTFPRYETGEFELMMAGQDDPVHKKYRQLVAKPFSPEATDLFTEQLRQSTNDLIDARIELGEGDAATWLANEIPARLTAILLGLPPEDGDTYRRWVWAITHVENPEEGAEIFAELVAHARTLIAERRTNPGNDIMSRVIMSKIDGESLSEDDLIGFFTILLLGGIDATARFLSSVFWRLAWDIELRRRLIAHPELIPNAVDELLRFYGPAMVGRLVTQEVTVGDITMKPGQTAMLWFPIASRDRSAFDSPDNIVIERTPNRHLSLGHGIHRCLGAHLIRVEARVAITEFLKRIPEFSLDPNKECEWLMGQVAGMLHVPIIFPKGKRLSE

>1FY3A

IVGGRKARPRQFPFLASIQNQGRHFCGGALIHARFVMTAASCFQSQNPGVSTVVLGAYDLRRRERQSRQTFSISSMSENGYDPQQNLNDLMLLQLDREANLTSSVTILPLPLQNATVEAGTRCQVAGWGSQRSGGRLSRFPRFVNVTVTPEDQCRPNNVCTGVLTRRGGICNGDQGTPLVCEGLAHGVASFSLGPCGRGPDFFTRVALFRDWIDGVLNNPGPGPA

>2VR3A

MRGSHHHHHHGSGTDITNQLTNVTVGIDSGTTVYPHQAGYVKLNYGFSVPNSAVKGDTFKITVPKELNLNGVTSTAKVPPIMAGDQVLANGVIDSDGNVIYTFTDYVNTKCDVKATLTMPAYIDPENVKKTGNVTLATGIGSTTANKTVLVDYEKYGKFYNLSIKGTIDQIDKTNNTYRQTIYVNPSGDNVIAPVLTGNLKPNTDSNALIDQQNTSIKVYKVDNAADLSESYFVNPENFEDVTNSVNITFPNPNQYKVEFNTPDDQITTPYIVVVNGHIDPNSKGDLALRSTLYGYNSNIIWRSMSWDNEVAFNNGSGSGDGIDCPVVP

>1R8ME

LEANEGSKTLQRNRKMAMGRKKFNMDPKKGIQFLVENELLQNTPEEIARFLYKGEGLNKTAIGDYLGEREELNLAVLHAFVDLHEFTDLNLVQALRQFLWSFRLPGEAQKIDRMMEAFAQRYCLCNPGVFQSTDTCYVLSYSVIMLNTDLHNPNVRDKMGLERFVAMNRGINEGGDLPEELLRNLYDSIRNEPFKIPEDDGND

>1BYPA

AEVLLGSSDGGLAFVPSDLSIASGEKITFKNNAGFPHNDLFDKKEVPAGVDVTKISMPEEDLLNAPGEEYSVTLTEKGTYKFYCAPHAGAGMVGKVTVN

>3D0KA

SNAMKPADLTNADRIALELGHAGRNAIPYLDDDRNADRPFTLNTYRPYGYTPDRPVVVVQHGVLRNGADYRDFWIPAADRHKLLIVAPTFSDEIWPGVESYNNGRAFTAAGNPRHVDGWTYALVARVLANIRAAEIADCEQVYLFGHSAGGQFVHRLMSSQPHAPFHAVTAANPGWYTLPTFEHRFPEGLDGVGLTEDHLARLLAYPMTILAGDQDIATDDPNLPSEPAALRQGPHRYARARHYYEAGQRAAAQRGLPFGWQLQVVPGIGHDGQAMSQVCASLWFDGRMPDAAELARLAGSQSA

>1ZHVA

APRIKLKILNGSYGIARLSASEAIPAWADGGGFVSITRTDDELSIVCLIDRIPQDVRVDPGWSCFKFQGPFAFDETGIVLSVISPLSTNGIGIFVVSTFDGDHLLVRSNDLEKTADLLANAGHSLLLEHHHHHH

>3H3JA

MNKFKGNKVVLIGNGAVGSSYAFSLVNQSIVDELVIIDLDTEKVRGDVMDLKHATPYSPTTVRVKAGEYSDCHDADLVVICAGARQKPGETRLDLVSKNLKIFKSIVGEVMASKFDGIFLVATNPVDILAYATWKFSGLPKERVIGSGTILDSARFRLLLSEAFDVAPRSVDAQIIGEHGDTELPVWSHANIAGQPLKTLLEQRPEGKAQIEQIFVQTRDAAYDIIQAKGATYYGVAMGLARITEAIFRNEDAVLTVSALLEGEYEEEDVYIGVPAVINRNGIRNVVEIPLNDEEQSKFAHSAKTLKDIMAEAEELK

>3H36A

SNAVELLQVDADLQAEIVGKYNADLQKAVQIEEKKASEIATEAVKEHVTAEYEERYAEHEEHDRIMRDVAEILEQMEHAEVRRLITEDKVRPD

>1Z3EB

MEKEKVLEMTIEELDLSVRSYNCLKRAGINTVQELANKTEEDMMKVRNLGRKSLEEVKAKLEELGLGLRKDDG

>3KFFA

EEATSKGQNLNVEKINGEWFSILLASDKREKIEEHGSMRVFVEHIHVLENSLAFKFHTVIDGECSEIFLVADKTEKAGEYSVMYDGFNTFTILKTDYDNYIMFHLINEKDGKTFQLMELYGRKADLNSDIKEKFVKLCEEHGIIKENIIDLTKTNRCLKARE

>2RB8A

MRLDAPSQIEVKDVTDTTALITWMPPSQPVDGFELTYGIKDVPGDRTTIDLTEDENQYSIGNLKPDTEYEVSLISRRGDMSSNPAKETFTTGLAAALEHHHHHH

>2RBDA

GMGILSGNPQDEPLHYGEVFSTWTYLSTNNGLINGYRSFINHTGDEDLKNLIDEAIQAMQDENHQLEELLRSNGVGLPPAPPDRPAARLDDIPVGARFNDPEISATISMDVAKGLVTCSQIIGQSIREDVALMFSQFHMAKVQFGGKMLKLNKNKGWLIPPPLHSDRPIKE

>2W1VA

MSTFRLALIQLQVSSIKSDNLTRACSLVREAAKQGANIVSLPECFNSPYGTTYFPDYAEKIPGESTQKLSEVAKESSIYLIGGSIPEEDAGKLYNTCSVFGPDGSLLVKHRKIHLFDIDVPGKITFQESKTLSPGDSFSTFDTPYCKVGLGICYDMRFAELAQIYAQRGCQLLVYPGAFNLTTGPAHWELLQRARAVDNQVYVATASPARDDKASYVAWGHSTVVDPWGQVLTKAGTEETILYSDIDLKKLAEIRQQIPILKQKRADLYTVESKKP

>3OBIA

GMPHHQYVLTLSCPDRAGIVSAVSTFLFENGQNILDAQQYNDTESGHFFMRVVFNAAAKVIPLASLRTGFGVIAAKFTMGWHMRDRETRRKVMLLVSQSDHCLADILYRWRVGDLHMIPTAIVSNHPRETFSGFDFGDIPFYHFPVNKDTRRQQEAAITALIAQTHTDLVVLARYMQILSDEMSARLAGRCINIHHSFLPGFKGAKPYHQAFDRGVKLIGATAHYVTSALDEGPIIDQDVERISHRDTPADLVRKGRDIERRVLSRALHYHLDDRVILNGRKTVVFTD

>2BZUA

RIAISNSNRTRSVPSLTTIWSISPTPNCSIYETQDANLFLCLTKNGAHVLGTITIKGLKGALREMHDNALSLKLPFDNQGNLLNCALESSTWRYQETNAVASNALTFMPNSTVYPRNKTAHPGNMLIQISPNITFSVVYNEINSGYAFTFKWSAEPGKPFHPPTAVFCYITEQGSHHHHHH

>1Q16A

MSKFLDRFRYFKQKGETFADGHGQLLNTNRDWEDGYRQRWQHDKIVRSTHGVNCTGSCSWKIYVKNGLVTWETQQTDYPRTRPDLPNHEPRGCPRGASYSWYLYSANRLKYPMMRKRLMKMWREAKALHSDPVEAWASIIEDADKAKSFKQARGRGGFVRSSWQEVNELIAASNVYTIKNYGPDRVAGFSPIPAMSMVSYASGARYLSLIGGTCLSFYDWYCDLPPASPQTWGEQTDVPESADWYNSSYIIAWGSNVPQTRTPDAHFFTEVRYKGTKTVAVTPDYAEIAKLCDLWLAPKQGTDAAMALAMGHVMLREFHLDNPSQYFTDYVRRYTDMPMLVMLEERDGYYAAGRMLRAADLVDALGQENNPEWKTVAFNTNGEMVAPNGSIGFRWGEKGKWNLEQRDGKTGEETELQLSLLGSQDEIAEVGFPYFGGDGTEHFNKVELENVLLHKLPVKRLQLADGSTALVTTVYDLTLANYGLERGLNDVNCATSYDDVKAYTPAWAEQITGVSRSQIIRIAREFADNADKTHGRSMIIVGAGLNHWYHLDMNYRGLINMLIFCGCVGQSGGGWAHYVGQEKLRPQTGWQPLAFALDWQRPARHMNSTSYFYNHSSQWRYETVTAEELLSPMADKSRYTGHLIDFNVRAERMGWLPSAPQLGTNPLTIAGEAEKAGMNPVDYTVKSLKEGSIRFAAEQPENGKNHPRNLFIWRSNLLGSSGKGHEFMLKYLLGTEHGIQGKDLGQQGGVKPEEVDWQDNGLEGKLDLVVTLDFRLSSTCLYSDIILPTATWYEKDDMNTSDMHPFIHPLSAAVDPAWEAKSDWEIYKAIAKKFSEVCVGHLGKETDIVTLPIQHDSAAELAQPLDVKDWKKGECDLIPGKTAPHIMVVERDYPATYERFTSIGPLMEKIGNGGKGIAWNTQSEMDLLRKLNYTKAEGPAKGQPMLNTAIDAAEMILTLAPETNGQVAVKAWAALSEFTGRDHTHLALNKEDEKIRFRDIQAQPRKIISSPTWSGLEDEHVSYNAGYTNVHELIPWRTLSGRQQLYQDHQWMRDFGESLLVYRPPIDTRSVKEVIGQKSNGNQEKALNFLTPHQKWGIHSTYSDNLLMLTLGRGGPVVWLSEADAKDLGIADNDWIEVFNSNGALTARAVVSQRVPAGMTMMYHAQERIVNLPGSEITQQRGGIHNSVTRITPKPTHMIGGYAHLAYGFNYYGTVGSNRDEFVVVRKMKNIDWLDGEGNDQVQESVK

>3CX5C

MAFRKSNVYLSLVNSYIIDSPQPSSINYWWNMGSLLGLCLVIQIVTGIFMAMHYSSNIELAFSSVEHIMRDVHNGYILRYLHANGASFFFMVMFMHMAKGLYYGSYRSPRVTLWNVGVIIFILTIATAFLGYCCVYGQMSHWGATVITNLFSAIPFVGNDIVSWLWGGFSVSNPTIQRFFALHYLVPFIIAAMVIMHLMALHIHGSSNPLGITGNLDRIPMHSYFIFKDLVTVFLFMLILALFVFYSPNTLGHPDNYIPGNPLVTPASIVPEWYLLPFYAILRSIPDKLLGVITMFAAILVLLVLPFTDRSVVRGNTFKVLSKFFFFIFVFNFVLLGQIGACHVEVPYVLMGQIATFIYFAYFLIIVPVISTIENVLFYIGRVNK

>3I7QA

MFTGSIVAIVTPMDEKGNVCRASLKKLIDYHVASGTSAIVSVGTTGESATLNHDEHADVVMMTLDLADGRIPVIAGTGANATAEAISLTQRFNDSGIVGCLTVTPYYNRPSQEGLYQHFKAIAEHTDLPQILYNVPSRTGCDLLPETVGRLAKVKNIIGIREATGNLTRVNQIKELVSDDFVLLSGDDASALDFMQLGGHGVISVTANVAARDMAQMCKLAAEGHFAEARVINQRLMPLHNKLFVEPNPIPVKWACKELGLVATDTLRLPMTPITDSGRETVRAALKHAGLL

>3P1GA

GSILAETHGTRPDLTDQPIPDADYTWYTDGSSFLQEGQRRAGAAVTTETEVIWARALPAGTSAQRAELIALTQALKMAEGKKLNVYTDSRYAFATAHVHSEGREIKNKNEILALLKALFLPKRLSIIHCPGHQKGNSAEARGNRMADQAAREAAMKAVLETSTLL

>3EA6A

QGDIGIDNLRNFYTKKDFVDLKDVKDNDTPIANQLQFSNESYDLISESKDFNKFSNFKGKKLDVFGISYNGQCNTKYIYGGVTATNEYLDKSRNIPINIWINGNHKTISTNKVSTNKKFVTAQEIDVKLRKYLQEEYNIYGHNGTKKGEEYGHKSKFYSGFNIGKVTFHLNNNDTFSYDLFYTGDDGLPKSFLKIYEDNKTVESEKFHLDVDISYKETI

>1EZ6A

ATSTKKLHKEPATLIKAIDGDTVKLMYKGQPMVFRLLLVDIPETKHPKKGVEKYGPEAAAFTKKMVENAKKIEVEFDKGQRTDKYGRGLAYIYADGKMVNEALVRQGLAKVAYVYKGNNTHEQLLRKAEAQAKKEKLNIWSEDNADSGQ

>3GXBA

MVLDVAFVLEGSDKIGEADFNRSKEFMEEVIQRMDVGQDSIHVTVLQYSYMVTVEYPFSEAQSKGDILQRVREIRYQGGNRTNTGLALRYLSDHSFLVSQGDREQAPNLVYMVTGNPASDEIKRLPGDIQVVPIGVGPNANVQELERIGWPNAPILIQDFETLPREAPDLVLQRCCSPHHHHHH

>1LG7A

VDEMDTHDPHQLRYEKFFFTVKMTVRSNRPFRTYSDVAAAVSHWDHMYIGMAGKRPFYKILAFLGSSNLKATPAVLADQGQPEYHAHCEGRAYLPHRMGKTPPMLNVPEHFRRPFNIGLYKGTVELTMTIYDDESLEAAPMIWDHFNSSKFSDFREKALMFGLIVEKKASGAWVLDSVSHFK

>2Y0GA

MAHHHHHHGHHHQLVSKGEELFTGVVPILVELDGDVNGHKFSVSGEGEGDATYGKLTLKFICTTGKLPVPWPTLVTTLXVQCFSRYPDHMKQHDFFKSAMPEGYVQERTIFFKDDGNYKTRAEVKFEGDTLVNRIELKGIDFKEDGNILGHKLEYNYNSHNVYIMADKQKNGIKVNFKIRHNIEDGSVQLADHYQQNTPIGDGPVLLPDNHYLSTQSALSKDPNEKRDHMVLLEFVTAAGITLGMDELYK

>1K3UB

TTLLNPYFGEFGGMYVPQILMPALNQLEEAFVSAQKDPEFQAQFADLLKNYAGRPTALTKCQNITAGTRTTLYLKREDLLHGGAHKTNQVLGQALLAKRMGKSEIIAETGAGQHGVASALASALLGLKCRIYMGAKDVERQSPNVFRMRLMGAEVIPVHSGSATLKDACNEALRDWSGSYETAHYMLGTAAGPHPYPTIVREFQRMIGEETKAQILDKEGRLPDAVIACVGGGSNAIGMFADFINDTSVGLIGVEPGGHGIETGEHGAPLKHGRVGIYFGMKAPMMQTADGQIEESYSISAGLDFPSVGPQHAYLNSIGRADYVSITDDEALEAFKTLCRHEGIIPALESSHALAHALKMMREQPEKEQLLVVNLSGRGDKDIFTVHDILKARGEI

>3NYSA

MIEFIDLKNQQARIKDKIDAGIQRVLRHGQYILGPEVTELEDRLADFVGAKYCISCANGTDALQIVQMALGVGPGDEVITPGFTYVATAETVALLGAKPVYVDIDPRTYNLDPQLLEAAITPRTKAIIPVSLYGQCADFDAINAIASKYGIPVIEDAAQSFGASYKGKRSCNLSTVACTSFFPSAPLGCYGDGGAIFTNDDELATAIRQIARHGQDRRYHHIRVGVNSRLDTLQAAILLPKLEIFEEEIALRQKVAAEYDLSLKQVGIGTPFIEVNNISVYAQYTVRMDNRESVQASLKAAGVPTAVHYPIPLNKQPAVADEKAKLPVGDKAATQVMSLPMHPYLDTASIKIICAALTNLEHHHHHH

>2A6ZA

GSDASKLSSDYSLPDLINTRKVPNNWQTGEQASLEEGRIVLTSNQNSKGSLWLKQGFDLKDSFTMEWTFRSVGYSGQTDGGISFWFVQDSNIPRDKQLYNGPVNYDGLQLLVDNNGPLGPTLRGQLNDGQKPVDKTKIYDQSFASCLMGYQDSSVPSTIRVTYDLEDDNLLKVQVDNKVCFQTRKVRFPSGSYRIGVTAQNGAVNNNAESFEIFKMQFFNGV

>1G72B

YDGQNCKEPGNCWENKPGYPEKIAGSKYDPKHDPVELNKQEESIKAMDARNAKRIANAKSSGNFVFDVK

>1A8P

SNLNVERVLSVHHWNDTLFSFKTTRNPSLRFENGQFVMIGLEVDGRPLMRAYSIASPNYEEHLEFFSIKVQNGPLTSRLQHLKEGDELMVSRKPTGTLVTSDLLPGKHLYMLSTGTGLAPFMSLIQDPEVYERFEKVVLIHGVRQVNELAYQQFITEHLPQSEYFGEAVKEKLIYYPTVTRESFHNQGRLTDLMRSGKLFEDIGLPPINPQDDRAMICGSPSMLDESCEVLDGFGLKISPRMGEPGDYLIERAFVEK

>1A8Y

GLDFPEYDGVDRVINVNAKNYKNVFKKYEVLALLYHEPPEDDKASQRQFEMEELILELAAQVLEDKGVGFGLVDSEKDAAVAKKLGLTEEDSIYVFKEDEVIEYDGEFSADTLVEFLLDVLEDPVELIEGERELQAFENIEDEIKLIGYFKNKDSEHYKAFKEAAEEFHPYIPFFATFDSKVAKKLTLKLNEIDFYEAFMEEPVTIPDKPNSEEEIVNFVEEHRRSTLRKLKPESMYETWEDDMDGIHIVAFAEEADPDGYEFLEILKSVAQDNTDNPDLSIIWIDPDDFPLLVPYWEKTFDIDLSAPQIGVVNVTDADSVWMEPSAEELEDWLEDVL

>1ABE

NLKLGFLVKQPEEPWFQTEWKFADKAGKDLGFEVIKIAVPDGEKTLNAIDSLAASGAKGFVICTPDPKLGSAIVAKARGYDMKVIAVDDQFVNAKGKPMDTVPLVMMAATKIGERQGQELYKEMQKRGWDVKESAVMAITANELDTARRRTTGSMDALKAAGFPEKQIYQVPTKSNDIPGAFDAANSMLVQHPEVKHWLIVGMNDSTVLGGVRATEGQGFKAADIIGIGINGVDAVSELSKAQATGFYGSLLPSPDVHGYKSSEMLYNWVAKDVEPPKFTEVTDVVLITRDNFKEELEKKGLGGK

>1AIR

ATDTGGYAATAGGNVTGAVSKTATSMQDIVNIIDAARLDANGKKVKGGAYPLVITYTGNEDSLINAAAANICGQWSKDPRGVEIKEFTKGITIIGANGSSANFGIWIKKSSDVVVQNMRIGYLPGGAKDGDMIRVDDSPNVWVDHNELFAANHECDGTPDNDTTFESAVDIKGASNTVTVSYNYIHGVKKVGLDGSSSSDTGRNITYHHNYYNDVNARLPLQRGGLVHAYNNLYTNITGSGLNVRQNGQALIENNWFEKAINPVTSRYDGKNFGTWVLKGNNITKPADFSTYSITWTADTKPYVNADSWTSTGTFPTVAYNYSPVSAQCVKDKLPGYAGVGKNLATLTSTAC

>1AL3

TWPDKGSLYVATTHTQARYALPGVIKGFIERYPRVSLHMHQGSPTQIAEAVSKGNADFAIATEALHLYDDLVMLPCYHWNRSIVVTPEHPLATKGSVSIEELAQYPLVTYTFGFTGRSELDTAFNRAGLTPRIVFTATDADVIKTYVRLGLGVGVIASMAVDPVSDPDLVKLDANGIFSHSTTKIGFRRSTFLRSYMYDFIQRFAPHLTRDVVDTAVALRSNEDIEAMFKDIKLPEK

>1ALHA

MPVLENRAAQGDITAPGGARRLTGDQTAALRDSLSDKPAKNIILLIGDGMGDSEITAARNYAEGAGGFFKGIDALPLTGQYTHYALNKKTGKPDYVTDSAASATAWSTGVKTYNGALGVDIHEKDHPTILEMAKAAGLATGNVSTAELQDATPAALVAHVTSRKCYGPSATSEKCPGNALEKGGKGSITEQLLNARADVTLGGGAKTFAETATAGEWQGKTLREQAEARGYQLVSDAASLNSVTEANQQKPLLGLFADGNMPVRWLGPKATYHGNIDKPAVTCTPNPQRNDSVPTLAQMTDKAIELLSKNEKGFFLQVEGASIDKQDHAANPCGQIGETVDLDEAVQRALEFAKKEGNTLVIVTANHAHASQIVAPDTKAPGLTQALNTKDGAVMVMSYGNSEEDSQEHTGSQLRIAAYGPHAANVVGLTDQTDLFYTMKAALGLK

>1AMF

GKITVFAAASLTNAMQDIATQFKKEKGVDVVSSFASSSTLARQIEAGAPADLFISADQKWMDYAVDKKAIDTATRQTLLGNSLVVVAPKASVQKDFTIDSKTNWTSLLNGGRLAVGDPEHVPAGIYAKEALQKLGAWDTLSPKLAPAEDVRGALALVERNEAPLGIVYGSDAVASKGVKVVATFPEDSHKKVEYPVAVVEGHNNATVKAFYDYLKGPQAAEIFKRYGFTIK

>1AMK

SAKPQPIAAANWKCNGTTASIEKLVQVFNEHTISHDVQCVVAPTFVHIPLVQAKLRNPKYVISAENAIAKSGAFTGEVSMPILKDIGVHWVILGHSERRTYYGETDEIVAQKVSEACKQGFMVIACIGETLQQREANQTAKVVLSQTSAIAAKLTKDAWNQVVLAYEPVWAIGTGKVATPEQAQEVHLLLRKWVSENIGTDVAAKLRILYGGSVNAANAATLYAKPDINGFLVGGASLKPEFRDIIDATR

>1AMX

TSSVFYYKTGDMLPEDTTHVRWFLNINNEKSYVSKDITIKDQIQGGQQLDLSTLNINVTGTHSNYYSGQSAITDFEKAFPGSKITVDNTKNTIDVTIPQGYGSYNSFSINYKTKITNEQQKEFVNNSQAWYQEHGKEEVNGKSFNHTVHN

>1ARB

GVSGSCNIDVVCPEGDGRRDIIRAVGAYSKSGTLACTGSLVNNTANDRKMYFLTAHHCGMGTASTAASIVVYWNYQNSTCRAPNTPASGANGDGSMSQTQSGSTVKATYATSDFTLLELNNAANPAFNLFWAGWDRRDQNYPGAIAIHHPNVAEKRISNSTSPTSFVAWGGGAGTTHLNVQWQPSGGVTEPGSSGSPIYSPEKRVLGQLHGGPSSCSATGTNRSDQYGRVFTSWTGGGAAASRLSDWLDPASTGAQFIDGLDS

>1ARU

SVTCPGGQSTSNSQCCVWFDVLDDLQTNFYQGSKCESPVRKILRIVFHDAIGFSPALTAAGQFGGGGADGSIIAHSNIELAFPANGGLTDTIEALRAVGINHGVSFGDLIQFATAVGMSNCPGSPRLEFLTGRSNSSQPSPPSLIPGPGNTVTAILDRMGDAGFSPDEVVDLLAAHSLASQEGLNSAIFRSPLDSTPQVFDTQFYIETLLKGTTQPGPSLGFAEELSPFPGEFRMRSDALLARDSRTACRWQSMTSSNEVMGQRYRAAMAKMSVLGFDRNALTDCSDVIPSAVSNNAAPVIPGGLTVDDIEVSCPSEPFPEIATASGPLPSLAPAP

>1AT0

CFTPESTALLESGVRKPLGELSIGDRVLSTANGQAVYSEVILFDRNLEQQNFVQLHTDGGAVLTVTPAHLVSVWQPESQKLTFVFADRIEEKNQVLVRDVETGELRPQRVVKVGSVRSKGVVAPLTREGTIVVNSVAASCYA

>1AV4

ASPFRLASAGEISEVQGILRTAGLLGPEKRIAYLGVLDPARGAGSEAEDRRFRVFIHDVSGARPQEVTVSVTNGTVISAVELDTAATGELPVLEEEFEVVEQLLATDERWLKALAARNLDVSKVRVAPLSAGVFEYAEERGRRILRGLAFVQDFPEDSAWAHPVDGLVAYVDVVSKEVTRVIDTGVFPVPAEHGNYTDPELTGPLRTTQKPISITQPEGPSFTVTGGNHIEWEKWSLDVGFDVREGVVLHNIAFRDGDRLRPIINRASIAEMVVPYGDPSPIRSWQNYFDTGEYLVGQYANSLELGCDCLGDITYLSPVISDAFGNPREIRNGICMHEEDWGILAKHSDLWSGINYTRRNRRMVISFFTTIGNDYGFYWYLYLDGTIEFEAKATGVVFTSAFPEGGSDNISQLAPGLGAPFHQHIFSARLDMAIDGFTNRVEEEDVVRQTMGPGNERGNAFSRKRTVLTRESEAVREADARTGRTWIISNPESKNRLNEPVGYKLHAHNQPTLLADPGSSIARRAAFATKDLWVTRYADDERYPTGDFVNQHSGGAGLPSYIAQDRDIDGQDIVVWHTFGLTHFPRVEDWPIMPVDTVGFKLRPEGFFDRSPVLDVPAN

>1AYL

MRVNNGLTPQELEAYGISDVHDIVYNPSYDLLYQEELDPSLTGYERGVLTNLGAVAVDTGIFTGRSPKDKYIVRDDTTRDTFWWADKGKGKNDNKPLSPETWQHLKGLVTRQLSGKRLFVVDAFCGANPDTRLSVRFITEVAWQAHFVKNMFIRPSDEELAGFKPDFIVMNGAKCTNPQWKEQGLNSENFVAFNLTERMQLIGGTWYGGEMKKGMFSMMNYLLPLKGIASMHCSANVGEKGDVAVFFGLSGTGKTTLSTDPKRRLIGDDEHGWDDDGVFNFEGGCYAKTIKLSKEAEPEIYNAIRRDALLENVTVREDGTIDFDDGSKTENTRVSYPIYHIDNIVKPVSKAGHATKVIFLTADAFGVLPPVSRLTADQTQYHFLSGFTAKLAPTPTFSACFGAAFLSLHPTQYAEVLVKRMQAAGAQAYLVNTGWNGTGKRISIKDTRAIIDAILNGSLDNAETFTLPMFNLAIPTELPGVDTKILDPRNTYASPEQWQEKAETLAKLFIDNFDKYTDTPAGAALVAAGPKL

>1B51A

ADVPAGVQLADKQTLVRNNGSEVQSLDPHKIEGVPESNVSRDLFEGLLISDVEGHPSPGVAEKWENKDFKVWTFHLRENAKWSDGTPVTAHDFVYSWQRLADPNTASPYASYLQYGHIANIDDIIAGKKPATDLGVKALDDHTFEVTLSEPVPYFYKLLVHPSVSPVPKSAVEKFGDKWTQPANIVTNGAYKLKNWVVNERIVLERNPQYWDNAKTVINQVTYLPISSEVTDVNRYRSGEIDMTYNNMPIELFQKLKKEIPNEVRVDPYLCTYYYEINNQKAPFNDVRVRTALKLALDRDIIVNKVKNQGDLPAYSYTPPYTDGAKLVEPEWFKWSQQKRNEEAKKLLAEAGFTADKPLTFDLLYNTSDLHKKLAIAVASIWKKNLGVNVNLENQEWKTFLDTRHQGTFDVARAGWCADYNEPTSFLNTMLSDSSNNTAHYKSPAFDKLIADTLKVADDTQRSELYAKAEQQLDKDSAIVPVYYYVNARLVKPWVGGYTGKDPLDNIYVKNLYIIKH

>1B6A

KVQTDPPSVPICDLYPNGVFPKGQECEYPEEKKALDQASEEIWNDFREAAEAHRQVRKYVMSWIKPGMTMIEICEKLEDCSRKLIKENGLNAGLAFPTGCSLNNCAAHYTPNAGDTTVLQYDDICKIDFGTHISGRIIDCAFTVTFNPKYDTLLKAVKDATNTGIKCAGIDVRLCDVGEAIQEVMESYEVEIDGKTYQVKPIRNLNGHSIGQYRIHAGKTVPIVKGGEATRMEEGEVYAIETFGSTGKGVVHDDMECSHYMKNFDVGHVPIRLPRTKHLLNVINENFGTLAFCRRWLDRLGESKYLMALKNLCDLGIVDPYPPLCDIKGSYTAQFEHTILLRPTCKEVVSRGDDY

>1BB9

TTGRLDLPPGFMFKVQAQHDYTATDTDELQLKAGDVVLVIPFQNPEEQDEGWLMGVKESDWNQHKELEKCRGVFPENFTERVQ

>1BDB

MKLKGEAVLITGGASGLGRALVDRFVAEGAKVAVLDKSAERLAELETDHGDNVLGIVGDVRSLEDQKQAASRCVARFGKIDTLIPNAGIWDYSTALVDLPEESLDAAFDEVFHINVKGYIHAVKACLPALVASRGNVIFTISNAGFYPNGGGPLYTAAKHAIVGLVRELAFELAPYVRVNGVGSGGINSDLRGPSSLGPLADMLKSVLPIGRMPEVEEYTGAYVFFATRGDAAPATGALLNYDGGLGVRGFFSGAGGNDLLEQLNIH

>1BFD

ASVHGTTYELLRRQGIDTVFGNPGSNELPFLKDFPEDFRYILALQEACVVGIADGYAQASRKPAFINLHSAAGTGNAMGALSNAWNSHSPLIVTAGQQTRAMIGVEALLTNVDAANLPRPLVKWSYEPASAAEVPHAMSRAIHMASMAPQGPVYLSVPYDDWDKDADPQSHHLFDRHVSSSVRLNDQDLDILVKALNSASNPAIVLGPDVDAANANADCVMLAERLKAPVWVAPSAPRCPFPTRHPCFRGLMPAGIAAISQLLEGHDVVLVIGAPVFRYHQYDPGQYLKPGTRLISVTCDPLEAARAPMGDAIVADIGAMASALANLVEESSRQLPTAAPEPAKVDQDAGRLHPETVFDTLNDMAPENAIYLNESTSTTAQMWQRLNMRNPGSYYFCAAGGLGFALPAAIGVQLAEPERQVIAVIGDGSANYSISALWTAAQYNIPTIFVIMNNGTYGALRWFAGVLEAENVPGLDVPGIDFRALAKGYGVQALKADNLEQLKGSLQEALSAKGPVLIEVSTV

>1BG2

DLAECNIKVMCRFRPLNESEVNRGDKYIAKFQGEDTVVIASKPYAFDRVFQSSTSQEQVYNDCAKKIVKDVLEGYNGTIFAYGQTSSGKTHTMEGKLHDPEGMGIIPRIVQDIFNYIYSMDENLEFHIKVSYFEIYLDKIRDLLDVSKTNLSVHEDKNRVPYVKGCTERFVCSPDEVMDTIDEGKSNRHVAVTNMNEHSSRSHSIFLINVKQENTQTEQKLSGKLYLVDLAGSEKVSKTGAEGAVLDEAKNINKSLSALGNVISALAEGSTYVPYRDSKMTRILQDSLGGNCRTTIVICCSPSSYNESETKSTLLFGQRAKTI

>1BG6

SKTYAVLGLGNGGHAFAAYLALKGQSVLAWDIDAQRIKEIQDRGAIIAEGPGLAGTAHPDLLTSDIGLAVKDADVILIVVPAIHHASIAANIASYISEGQLIILNPGATGGALEFRKILRENGAPEVTIGETSSMLFTCRSERPGQVTVNAIKGAMDFACLPAAKAGWALEQIGSVLPQYVAVENVLHTSLTNVNAVMHPLPTLLNAARCESGTPFQYYLEGITPSVGSLAEKVDAERIAIAKAFDLNVPSVCEWYPATIYEAVQGNPAYRGIAGPINLNTRYFFEDVSTGLVPLSELGRAVNVPTPLIDAVLDLISSLIDTDFRKEGRTLEKLGLSGLTAAGIRSAVE

>1CHD

LLSSEKLIAIGASTGGTEAIRHVLQPLPLSSPAVIITQHMPPGFTRSFAERLNKLCQISVKEAEDGERVLPGHAYIAPGDKHMELARSGANYQIKIHDGPPVNRHRPSVDVLFHSVAKHAGRNAVGVILTGMGNDGAAGMLAMYQAGAWTIAQNEASCVVFGMPREAINMGGVSEVVDLSQVSQQMLAKISAGQAIRI

>1CIY

YTPIDISLSLTQFLLSEFVPGAGFVLGLVDIIWGIFGPSQWDAFLVQIEQLINQRIEEFARNQAISRLEGLSNLYQIYAESFREWEADPTNPALREEMRIQFNDMNSALTTAIPLLAVQNYQVPLLSVYVQAANLHLSVLRDVSVFGQRWGFDAATINSRYNDLTRLIGNYTDYAVRWYNTGLERVWGPDSRDWVRYNQFRRELTLTVLDIVALFSNYDSRRYPIRTVSQLTREIYTNPVLENFDGSFRGMAQRIEQNIRQPHLMDILNSITIYTDVHRGFNYWSGHQITASPVGFSGPEFAFPLFGNAGNAAPPVLVSLTGLGIFRTLSSPLYRRIILGSGPNNQELFVLDGTEFSFASLTTNLPSTIYRQRGTVDSLDVIPPQDNSVPPRAGFSHRLSHVTMLSQAAGAVYTLRAPTFSWQHRSAEFNNIIPSSQITQIPLTKSTNLGSGTSVVKGPGFTGGDILRRTSPGQISTLRVNITAPLSQRYRVRIRYASTTNLQFHTSIDGRPINQGNFSATMSSGSNLQSGSFRTVGFTTPFNFSNGSSVFTLSAHVFNSGNEVYIDRIEFVPAEVT

>1CLC

IETKVSAAKITENYQFDSRIRLNSIGFIPNHSKKATIAANCSTFYVVKEDGTIVYTGTATSMFDNDTKETVYIADFSSVNEEGTYYLAVPGVGKSVNFKIAMNVYEDAFKTAMLGMYLLRCGTSVSATYNGIHYSHGPCHTNDAYLDYINGQHTKKDSTKGWHDAGDYNKYVVNAGITVGSMFLAWEHFKDQLEPVALEIPEKNNSIPDFLDELKYEIDWILTMQYPDGSGRVAHKVSTRNFGGFIMPENEHDERFFVPWSSAATADFVAMTAMAARIFRPYDPQYAEKCINAAKVSYEFLKNNPANVFANQSGFSTGEYATVSDADDRLWAAAEMWETLGDEEYLRDFENRAAQFSKKIEADFDWDNVANLGMFTYLLSERPGKNPALVQSIKDSLLSTADSIVRTSQNHGYGRTLGTTYYWGCNGTVVRQTMILQVANKISPNNDYVNAALDAISHVFGRNYYNRSYVTGLGINPPMNPHDRRSGADGIWEPWPGYLVGGGWPGPKDWVDIQDSYQTNEIAINWNAALIYALAGFVNYN

>1CNV

DISSTEIAVYWGQREDGLLRDTCKTNNYKIVFISFLDKFGCEIRKPELELEGVCGPSVGNPCSFLESQIKECQRMGVKVFLALGGPKGTYSACSADYAKDLAEYLHTYFLSERREGPLGKVALDGIHFDIQKPVDELNWDNLLEELYQIKDVYQSTFLLSAAPGCLSPDEYLDNAIQTRHFDYIFVRFYNDRSCQYSTGNIQRIRNAWLSWTKSVYPRDKNLFLELPASQATAPGGGYIPPSALIGQVLPYLPDLQTRYAGIALWNRQADKETGYSTNIIRYL

>1COT

DGDAAKGEKEFNKCKACHMIQAPDGTDIIKGGKTGPNLYGVVGRKIASEEGFKYGEGILEVAEKNPDLTWTEADLIEYVTDPKPWLVKMTDDKGAKTKMTFKMGKNQADVVAFLAQNSPDA

>1CPO

EPGSGIGYPYDNNTLPYVAPGPTDSRAPCPALNALANHGYIPHDGRAISRETLQNAFLNHMGIANSVIELALTNAFVVCEYVTGSDCGDSLVNLTLLAEPHAFEHDHSFSRKDYKQGVANSNDFIDNRNFDAETFQTSLDVVAGKTHFDYADMNEIRLQRESLSNELDFPGWFTESKPIQNVESGFIFALVSDFNLPDNDENPLVRIDWWKYWFTNESFPYHLGWHPPSPAREIEFVTSASSAVLAASVTSTPSSLPSGAIGPGAEAVPLSFASTMTPFLLATNAPYYAQDPTLGPND

>1CV8

NEQYVNKLENFKIRETQGNNGWCAGYTMSALLNATYNTNKYHAEAVMRFLHPNLQGQQFQFTGLTPREMIYFGQTQGRSPQLLNRMTTYNEVDNLTKNNKGIAILGSRVESRNGMHAGHAMAVVGNAKLNNGQEVIIIWNPWDNGFMTQDAKNNVIPVSNGDHYQWYSSIYGY

>1CVL

ADTYAATRYPVILVHGLAGTDKFANVVDYWYGIQSDLQSHGAKVYVANLSGFQSDDGPNGRGEQLLAYVKQVLAATGATKVNLIGHSQGGLTSRYVAAVAPQLVASVTTIGTPHRGSEFADFVQDVLKTDPTGLSSTVIAAFVNVFGTLVSSSHNTDQDALAALRTLTTAQTATYNRNFPSAGLGAPGSCQTGAATETVGGSQHLLYSWGGTAIQPTSTVTGATDTSTGTLDVANVTDPSTLALLATGAVMINRASGQNDGLVSRCSSLFGQVISTSYHWNHLDEINQLLGVRGANAEDPVAVIRTHVNRLKLQGV

>1CYO

SKAVKYYTLEEIQKHNNSKSTWLILHYKVYDLTKFLEEHPGGEEVLREQAGGDATENFEDVGHSTDARELSKTFIIGELHPDDRSKIT

>4FHZA

MHHHHHHSSGLVPRGSGMKETAAAKFERQHMDSPDLGTDDDDKAMADIMTRKLTFGRRGAAPGEATSLVVFLHGYGADGADLLGLAEPLAPHLPGTAFVAPDAPEPCRANGFGFQWFPIPWLDGSSETAAAEGMAAAARDLDAFLDERLAEEGLPPEALALVGFSQGTMMALHVAPRRAEEIAGIVGFSGRLLAPERLAEEARSKPPVLLVHGDADPVVPFADMSLAGEALAEAGFTTYGHVMKGTGHGIAPDGLSVALAFLKERLPDACGRTRAPPPPPLRSGC

>4FHRA

GTKFSKEQLRTFQMIHENFGRALSTYLSGRLRTFVDVEISIDQLTYEEFIRSVMIPSFIVIFTGDVFEGSAIFEMRLDLFYTMLDIIMGGPGENPPNRPPTEIETSIMRKEVTNMLTLLAQAWSDFQYFIPSIENVETNPQFVQIVPPNEIVLLVTASVSWGEFTSFINVCWPFSLLEPLLEKLSDR

>4FGZA

MTLIENLNSDKTFLENNQYTDEGVKVYEFIFGENYISSGGLEATKKILSDIELNENSKVLDIGSGLGGGCMYINEKYGAHTHGIDICSNIVNMANERVSGNNKIIFEANDILTKEFPENNFDLIYSRDAILHLSLENKNKLFQKCYKWLKPTGTLLITDYCATEKENWDDEFKEYVKQRKYTLITVEEYADILTACNFKNVVSKDLSDYWNQLLEVEHKYLHENKEEFLKLFSEKKFISLDDGWSRKIKDSKRKMQRWGYFKATKN

>4FFXA

GSHMAAGGDHGSPDSYRSPLASRYASPEMCFVFSDRYKFRTWRQLWLWLAEAEQTLGLPITDEQIREMKSNLENIDFKMAAEEEKRLRHDVMAHVHTFGHCCPKAAGIIHLGATSCYVGDNTDLIILRNALDLLLPKLARVISRLADFAKERASLPTLGFTHFQPAQLTTVGKRCCLWIQDLCMDLQNLKRVRDDLRFRGVKGTTGTQASFLQLFEGDDHKVEQLDKMVTEKAGFKRAFIITGQTYTRKVDIEVLSVLASLGASVHKICTDIRLLANLKEMEEPFEKQQIGSSAMPYKRNPMRSERCCSLARHLMTLVMDPLQTASVQWFERTLDDSANRRICLAEAFLTADTILNTLQNISEGLVVYPKVIERRIRQELPFMATENIIMAMVKAGGSRQDCHEKIRVLSQQAASVVKQEGGDNDLIERIQVDAYFSPIHSQLDHLLDPSSFTGRASQQVQRFLEEEVYPLLKPYESVMKVKAELCL

>4AVXA

SMGRGSGTFERLLDKATSQLLLETDWESILQICDLIRQGDTQAKYAVNSIKKKVNDKNPHVALYALEVMESVVKNCGQTVHDEVANKQTMEELKDLLKRQVEVNVRNKILYLIQAWAHAFRNEPKYKVVQDTYQIMKVEGHVFPEFKESDAMFAAERAPDWVDAEECHRCRVQFGVMTRKHHCRACGQIFCGKCSSKYSTIPKFGIEKEVRVCEPCYEQLNRKAEG

>4FEIA

QGGPWTPAADWRDAGTHLDLLLDVPGVDAGTLALAEDGGQLTVSGERPGTEHLLRSERPSGRFVRELAFPEPVRPASGVASLAGGVLTVRFEKLRPTIDVTA

>4FF1A

MGGSHHHHHHRSESTVTEELKEGIDAVYPSLVGTADSKAEGIKNYFKLSFTLPEEQKSRTVGSEAPLKDVAQALSSRARYELFTEKETANPAFNGEVIKRYKELMEHGEGIADILRSRLAKFLNTKDVGKRFAQGTEANRWVGGKLLNIVEQDGDTFKYNEQLLQTAVLAGLQWRLTATSNTAIKDAKDVAAITGIDQALLPEGLVEQFDTGMTLTEAVSSLAQKIESYWGLSRNPNAPLGYTKGIPTAMAAEILAAFVESTDVVENIVDMSEIDPDNKKTIGLYTITELDSFDPINSFPTAIEEAVLVNPTEKMFFGDDIPPVANTQLRNPAVRNTPEQKAALKAEQATEFYVHTPMVQFYETLGKDRILELMGAGTLNKELLNDNHAKSLEGKNRSVEDSYNQLFSVIEQVRAQSEDISTVPIHYAYNMTRVGRMQMLGKYNPQSAKLVREAILPTKATLDLSNQNNEDFSAFQLGLAQALDIKVHTMTREVMSDELTKLLEGNLKPAIDMMVEFNTTGSLPENAVDVLNTALGDRKSFVALMALMEYSRYLVAEDKSAFVTPLYVEADGVTNGPINAMMLMTGGLFTPDWIRNIAKGGLFIGSPNKTMNEHRSTADNNDLYQASTNALMESLGKLRSNYASNMPIQSQIDSLLSLMDLFLPDINLGENGALELKRGIAKNPLTITIYGSGARGIAGKLVSSVTDAIYERMSDVLKARAKDPNISAAMAMFGKQAASEAHAEELLARFLKDMETLTSTVPVKRKGVLELQSTGTGAKGKINPKTYTIKGEQLKALQENMLHFFVEPLRNGITQTVGESLVYSTEQLQKATQIQSVVLEDMFKQRVQEKLAEKAKDPTWKKGDFLTQKELNDIQASLNNLAPMIETGSQTFYIAGSENAEVANQVLATNLDDRMRVPMSIYAPAQAGVAGIPFMTIGTGDGMMMQTLSTMKGAPKNTLKIFDGMNIGLNDITDASRKANEAVYTSWQGNPIKNVYESYAKFMKNVDFSKLSPEALEAIGKSALEYDQRENATVDDIANAASLIERNLRNIALGVDIRHKVLDKVNLSIDQMAAVGAPYQNNGKIDLSNMTPEQQADELNKLFREELEARKQKVAKAR

>4FE1A

MTISPPEREPKVRVVVDNDPVPTSFEKWAKPGHFDRTLARGPQTTTWIWNLHALAHDFDTHTSDLEDISRKIFSAHFGHLAVVFIWLSGMYFHGAKFSNYEAWLADPTGIKPSAQVVWPIVGQGILNGDVGGGFHGIQITSGLFQLWRASGITNEFQLYCTAIGGLVMAGLMLFAGWFHYHKRAPKLEWFQNVESMLNHHLAGLLGLGSLAWAGHQIHVSLPINKLLDAGVAAKDIPLPHEFILNPSLMAELYPKVDWGFFSGVIPFFTFNWAAYSDFLTFNGGLNPVTGGLWLSDTAHHHLAIAVLFIIAGHMYRTNWGIGHSLKEILEAHKGPFTGAGHKGLYEVLTTSWHAQLAINLAMMGSLSIIVAQHMYAMPPYPYLATDYPTQLSLFTHHMWIGGFLVVGGAAHGAIFMVRDYDPAMNQNNVLDRVLRHRDAIISHLNWVCIFLGFHSFGLYVHNDTMRAFGRPQDMFSDTGIQLQPVFAQWVQNLHTLAPGGTAPNAAATASVAFGGDVVAVGGKVAMMPIVLGTADFMVHHIHAFTIHVTVLILLKGVLFARSSRLIPDKANLGFRFPCDGPGRGGTCQVSGWDHVFLGLFWMYNCISVVIFHFSWKMQSDVWGTVAPDGTVSHITGGNFAQSAITINGWLRDFLWAQASQVIGSYGSALSAYGLLFLGAHFIWAFSLMFLFSGRGYWQELIESIVWAHNKLKVAPAIQPRALSIIQGRAVGVAHYLLGGIATTWAFFLARIISVG

>4AVPA

SMGPTSQRRGSLQLWQFLVALLDDPSNSHFIAWTGRGMEFKLIEPEEVARRWGIQKNRPAMNYDKLSRSLRYYYEKGIMQKVAGERYVYKFVCDPEALFSMAFSDN

>4FD5A

MLDSKLNNIRFETISSKYYDDVIEHLRQTFFADEPLNKAVNLTRPGQGHPLLEQHSLSTLKDNVSIMAISNDGDIAGVALNGILYGNTDIEKSREKLNEIQDESFKKIFKLLYEQNLKINLFKQFDVDKIFEIRILSVDSRFRGKGLAKKLIEKSEELALDRGFQVMKTDATGAFSQRVVSSLGFITKCEINYTDYLDENGEQIFVVDPPHEKLKIMCKVIN

>4FCIA

MSAKSRTIGIIGAPFSKGQPRGGVEEGPTVLRKAGLLEKLKEQECDVKDYGDLPFADIPNDSPFQIVKNPRSVGKASEQLAGKVAEVKKNGRISLVLGGDHSLAIGSISGHARVHPDLGVIWVDAHTDINTPLTTTSGNLHGQPVSFLLKELKGKIPDVPGFSWVTPCISAKDIVYIGLRDVDPGEHYILKTLGIKYFSMTEVDRLGIGKVMEETLSYLLGRKKRPIHLSFDVDGLDPSFTPATGTPVVGGLTYREGLYITEEIYKTGLLSGLDIMEVNPSLGKTPEEVTRTVNTAVAITLACFGLAREGNHKPIDYLNPPK

>4F9KA

MSGLNDIFEAQKIEWHEHHHHHHENLYFQSHMEDESLKGCELYVQLHGIQQVLKDCIVHLCISKPERPMKFLREHFEKLEKEENRQILARQKSNS

>4F99B

GPGTRTGRLKKPFVKVEDMSQLYRPFYLQLTNMPFINYSIQKPCSPFDVDKPSSMQKQTQVKLRIQTDGDKYGGTSIQLQLKEKKKKGYCECCLQKYEDLETHLLSEQHRNFAQSNQYQVVDDIVSKLVFDFVEYEKDTPKKKR

>4F8CA

MAHHHHHHSSGLEVLFQGPPVSHSINNPSIQHVQDFATLSARSLRANVLLNSDDHSVPIHAKNPSELLEAIDNNISQTAQDWGVSIQEVEVILGSSKRIIEPVAGVTANTIMKLFLDNDIFSYSFEKGQSLSLSQLQERLASLPAHKNFILRVNDGGLGHAYVIDFPATTNPSRDAFLYQSDLGEGVTREVRFEDWMTQKASHPISLDDINTHFIGIAQDQIDLAHIAKLFDVDGNVKMLRADHLISHKTSEFNFQLFEYDLKNLENNMSIIKTH

>4F84A

MGSSHHHHHHSSGLVPRGSHMAAASAPVPGPGGASSTARGRIPAPATPYQEDIARYWNNEARPVNLRLGDVDGLYHHHYGIGAVDHAALGDPGDGGYEARLIAELHRLESAQAEFLLDHLGPVGPGDTLVDAGCGRGGSMVMAHQRFGCKVEGVTLSAAQAEFGNRRARELGIDDHVRSRVCNMLDTPFEKGTVAASWNNESSMYVDLHDVFAEHSRFLRVGGRYVTVTGCWNPRYGQPSKWVSQINAHFECNIHSRREYLRAMADNRLVPQTVVDLTPETLPYWELRATSSLVTGIEEAFIESYRDGSFQYVLIAADRV

>4F6OA

EQAKAQLSNGYNNPNVNASNMYGPPQNMSLPPPQTQTIQGTDQPYQYSQCTGRRKALIIGINYIGSKNQLRGCINDAHNIFNFLTNGYGYSSDDIVILTDDQNDLVRVPTRANMIRAMQWLVKDAQPNDSLFLHYSGHGGQTEDLDGDEEDGMDDVIYPVDFETQGPIIDDEMHDIMVKPLQQGVRLTALFDSCHSGTVLDLPYTYSTKGIIKEPNIWKDVGQDGLQAAISYATGNRAALIGSLGSIFKTVKGGMGNNVDRERVRQIKFSAADVVMLSGSKDNQTSADAVEDGQNTGAMSHAFIKVMTLQPQQSYLSLLQNMRKELAGKYSQKPQLSSSHPIDVNLQFIM

>2LT5A

RPCKYKLKKSTNKFCVTCENQAPVHFVGVGSCGSGGSGIFLETSLSAGSDWLTFQKKHITNTRDVDCDNIMSTNLFHCKDKNTFIYSRPEPVKAICKGIIASKNVLTTSEFYLSDCNVTS

>4F52B

GSMDVDTPSGTNSGAGKKRFEVKKWNAVALWAWDIVVDNCAICRNHIMDLCIECQANQASATSEECTVAWGVCNHAFHFHCISRWLKTRQVCPLDNREWEFQKYGH

>4F52E

GSMAVEELQSIIKRCQILEEQDFKEEDFGLFQLAGQRCIEEGHTDQLLEIIQNEKNKVIIKNMGWNLVGPVVRCLLCKDKEDSKRKVYFLIFDLLVKLCNPKELLLGLLELIEEPSGKQISQSILLLLQPLQTVIQKLHNKAYSIGLALSTLWNQLSLLPVPYSKEQIQMDDYGLCQCCKALIEFTKPFVEEVIDNKENSLENEKLKDELLKFCFKSLKCPLLTAQFFEQSEEGGNDPFRYFASEIIGFLSAIGHPFPKMIFNHGRKKRTWNYLEFEEEENKQLADSMASLAYLVFVQGIHIDQLPMVLSPLYLLQFNMGHIEVFLQRTEESVISKGLELLENSLLRIEDNSLLYQYLEIKSFLTVPQGLVKVMTLCPIETLRKKSLAMLQLYINKLDSQGKYTLFRCLLNTSNHSGVEAFIIQNIKNQIDMSLKRTRNNKWFTGPQLISLLDLVLFLPEGAETDLLQNSDRIMASLNLLRYLVIKDNENDNQTGLWTELGNIENNFLKPLHIGLNMSKAHYEAEIKNSQEAQKSKDLCSITVSGEEIPNMPPEMQLKVLHSALFTFDLIESVLARVEELIEIKTKSTSEENIGIK

>4F3WA

GPGSMPDIDWKQLRDKATQVAAGAYAPYSRFPVGAAALVDDGRVVTGCNVENVSYGLALCAECGVVCALHATGGGRLVALACVDGRGAPLMPCGRCRQLLFEHGGPELLVDHLAGPRRLGDLLPEPFHADLTGEP

>4F2ME

YPYDVPDYAGAQPARSPGLVPRGSRTANLNNGFYPVSSSEVGLVNKSVVLLPSFYTHTIVNITIDLGMKRSGYGQPIASTLSNITLPMQDNNTDVYCIRSDQFSVYVHSTCKSSLWDNIFKRNCTDVLDATAVIKTGTCPFSFDKLNNYLTFNKFCLSLSPVGANCKFDVAARTRTNEQVVRSLYVIYEEGDNIVLVPRGSDYKDDDDK

>4F2ZA

MSSQVEHPAGGYKKLFETVEELSSPLTAHVTGRIPLWLTGSLLRCGPGLFEVGSEPFYHLFDGQALLHKFDFKEGHVTYHRRFIRTDAYVRAMTEKRIVITEFGTCAFPDPCKNIFSRFFSYFRGVEVTDNALVNIYPVGEDYYACTETNFITKVNPETLETIKQVDLCNYVSVNGATAHPHIENDGTVYNIGNCFGKNFSIAYNIVKIPPLQADKEDPISKSEIVVQFPCSDRFKPSYVHSFGLTPNYIVFVETPVKINLFKFLSSWSLWGANYMDCFESNETMGVWLHIADKKRKKYINNKYRTSPFNLFHHINTYEDHEFLIVDLCCWKGFEFVYNYLYLANLRENWEEVKKNARKAPQPEVRRYVLPLNIDKADTGKNLVTLPNTTATAILCSDETIWLEPEVLFSGPRQAFEFPQINYQKYGGKPYTYAYGLGLNHFVPDRLCKLNVKTKETWVWQEPDSYPSEPIFVSHPDALEEDDGVVLSVVVSPGAGQKPAYLLILNAKDLSEVARAEVEINIPVTFHGLFKKS

>4F0VA

MGSSHHHHHHSSGENLYFEGSHMASMTGGQQMGRMDSLDQCIVNACKNSWDKSYLAGTPNKDNCSGFVQSVAAELGVPMPRGNANAMVDGLEQSWTKLASGAEAAQKAAQGFLVIAGLKGRTYGHVAVVISGPLYRQKYPMCWCGSIAGAVGQSQGLKSVGQVWNRTDRDRLNYYVYSLASCSLPRAS

>4F02A

GPLGSMNPSAPSYPMASLYVGDLHPDVTEAMLYEKFSPAGPILSIRVCRDMITRRSLGYAYVNFQQPADAERALDTMNFDVIKGKPVRIMWSQRDPSLRKSGVGNIFIKNLDKSIDNKALYDTFSAFGNILSCKVVCDENGSKGYGFVHFETQEAAERAIEKMNGMLLNDRKVFVGRFKSRKEREAELGARAKEFYPYDVPDYAGSSGRIVTD

>4ASUH

AEAAAAQAPAAGPGQMSFTFASPTQVFFNSANVRQVDVPTQTGAFGILAAHVPTLQVLRPGLVVVHAEDGTTSKYFVSSGSVTVNADSSVQLLAEEAVTLDMLDLGAAKANLEKAQSELLGAADEATRAEIQIRIEANEALVKALE

>4EY0A

HSNEKWFHGKLGAGRDGRHIAERLLTEYCIETGAPDGSFLVRESETFVGDYTLSFWRNGKVQHCRIHSRQDAGTPKFFLTDNLVFDSLYDLITHYQQVPLRCNEFEMRLSEPVPQTNAHESKEWYHASLTRAQAEHMLMRVPRDGAFLVRKRNEPNSYAISFRAEGKIKHCRVQQEGQTVMLGNSEFDSLVDLISYYEKHPLYRKMKLRYPINEEALEKIGTAEPDFGALFEGRNPGFYVEANPMP

>4ARZB

MSLEATDSKAMVLLMGVRRCGKSSICKVVFHNMQPLDTLYLESTSNPSLEHFSTLIDLAVMELPGQLNYFEPSYDSERLFKSVGALVYVIDSQDEYINAITNLAMIIEYAYKVNPSINIEVLIHKVDGLSEDFKVDAQRDIMQRTGEELLELGLDGVQVSFYLTSIFDHSIYEAFSRIVQKLIPELSFLENMLDNLIQHSKIEKAFLFDVNSKIYVSTDSNPVDIQMYEVCSEFIDVTIDLFDLYKAPVLRNSQKSSDKDNVINPRNELQNVSQLANGVIIYLRQMIRGLALVAIIRPNGTDMESCLTVADYNIDIFKKGLEDIWANARASQAKNSIEDDV

>4EVWA

MIVIPMAGMSSRFFKAGYTQPKYMLEAHGQTLFEHSVNSFAAYFASTPFLFIVRNVYDTAVFVREKATQLGIKQFYIAELHTETRGQAETVTLGLEELAKQGVDYQGSITVFNIDTFRPNFVFPDISQHSDGYLEVFQGGGDNWSFAKPEHAGSTKVIQTAEKNPISDLCSTGLYHFNRKEDYLEAYREYVARPSQEWERGELYIAPLYNELIQKGLNIHYHLIARHEVIFCGVPDEYTDFLRQPQPLEHHHHHH

>4EVFA

MPKVTDIANELKQAIDAKDEVQIAFIASEYSAESREKIAKAYVASYGKELPDDIKKALKGGSEESLLMDLFSDRHEVRAQHIRDALSGRNDHMAFFDTVILCTPEDWHETVAAYTRMFKKPLVEDFMKDVGRKEDWCLLMEKWMAHERVSRPGSPEDEAQRLDQAFDQKNTAYLIDFFGTVPSAEYRPIAEAFKAQNGKSIEQAIATIYTKTDYYTFYCAHFALLGMHRLAAYLINCACNDKGDEKRMRRITGMMVDKCLGAKHAYKIYGDMGTDIERCFDKRMAPILRTLWRVK

>4EUWA

MAHHHHHHVDDDDKMSENLYFQSSKNKPHVKRPMNAFMVWAQAARRKLADQYPHLHNAELSKTLGKLWRLLNESEKRPFVEEAERLRVQHKKDHPDYKYQPRRRKS

>4ETYA

SMQEGSLPDITIFPNSSLMISQGTFVTVVCSYSDKHDLYNMVRLEKDGSTFMEKSTEPYKTEDEFEIGPVNETITGHYSCIYSKGITWSERSKTLELKVIKENVIQTPAPGPTSEHLG

>4ETPB

GASEIAALEKEIAALEKEIAALEKEISKQEKFYNDTYNTVCKELLRSRRLENSIIEQKGTMRVYAYVMEQNLPENLLFDYENGVITQGLSEHVYKFNRVIPHLKVSEDCFFTQEYSVYHDMALNQKKNFNLISLSTTPHGSLRESLIKFLAEKDTIYQKQYVITLQFVFLSDDEFSQDMLLDYSHNDKDSIKLKFEKHSISLDSKLVIIENGLEDLPLNFSADEHPNLPHSGMGIIKVQFFPRDSKSDGNNDPVPVDFYFIELNNLKSIEQFDKSIFKKESAETPIALVLKKLISDTKSFFLLNLNDSKNVNKLLTISEEVQTQLAKRKKKLT

>4ESVA

MSELFSERIPPQSIEAEQAVLGAVFLDPAALVPASEILIPEDFYRAAHQKIFHAMLRVADRGEPVDLVTVTAELAASEQLEEIGGVSYLSELADAVPTAANVEYYARIVEEKSVLRRLIRTATSIAQDGYTREDEIDVLLDEADRKIMEVSQRKHSGAFKNIKDILVQTYDNIEMLHNRDGEITGIPTGFTELDRMTSGFQRSDLIIVAARPSVGKTAFALNIAQNVATKTNENVAIFSLEMSAQQLVMRMLCAEGNINAQNLRTGKLTPEDWGKLTMAMGSLSNAGIYIDDTPSIRVSDIRAKCRRLKQESGLGMIVIDYLQLIQGSGRSKENRQQEVSEISRSLKALARELEVPVIALSQLSRSVEQRQDKRPMMSDIRESGSIEQDADIVAFLYRDDYYNKDSENKNIIEIIIAKQRNGPVGTVQLAFIKEYNKFVNLERRFDEAQIPPGA

>4ESEA

SNAMSKVLVLKSSILATSSQSNQLADFFVEQWQAAHAGDQITVRDLAAQPIPVLDGELVGALRPSGTALTPRQQEALALSDELIAELQANDVIVIAAPMYNFNIPTQLKNYFDMIARAGVTFRYTEKGPEGLVTGKRAIILTSRGGIHKDTPTDLVVPYLRLFLGFIGITDVEFVFAEGIAYGPEVATKAQADAKTLLAQVVAA

>2LS8A

MVCPIDWRAFQSNCYFPLTDNKTWAESERNCSGMGAHLMTISTEAEQNFIIQFLDRRLSYFLGLRDENAKGQWRWVDQTPFNPRRVFWHKNEPDNSQGENCVVLVYNQDKWAWNDVPCNFEASRICKIPGTTLNAENLYFQSHHHHHHWSHPQFEK

>4ERRA

MGQIFTVQELKERAKVFAKPIGASYQGILDQLDLVHQAKGRDQIAASFELNKKINDYIAEHPTSGRNQALTQLKEQVTSALGLEHHHHHH

>4EPAA

GAMGQTSQQDESTLVVTASKQSSRSASANNVSSTVVSAPELSDAGVTASDKLPRVLPGLNIENSGNMLFSTISLRGVSSAQDFYNPAVTLYVDGVPQLSTNTIQALTDVQSVELLRGPQGTLYGKSAQGGIINIVTQQPDSTPRGYIEGGVSSRDSYRSKFNLSGPIQDGLLYGSVTLLRQVDDGDMINPATGSDDLGGTRASIGNVKLRLAPDDQPWEMGFAASRECTRATQDAYVGWNDIKGRKLSISDGSPDPYMRRCTDSQTLSGKYTTDDWVFNLISAWQQQHYSRTFPSGSLIVNMPQRWNQDVQELRAATLGDARTVDMVFGLYRQNTREKLNSAYDMPTMPYLSSTGYTTAETLAAYSDLTWHLTDRFDIGGGVRFSHDKSSTQYHGSMLGNPFGDQGKSNDDQVLGQLSAGYMLTDDWRVYTRVAQGYKPSGYNIVPTAGLDAKPFVAEKSINYELGTRYETADVTLQAATFYTHTKDMQLYSGPVRMQTLSNAGKADATGVELEAKWRFAPGWSWDINGNVIRSEFTNDSELYHGNRVPFVPRYGAGSSVNGVIDTRYGALMPRLAVNLVGPHYFDGDNQLRQGTYATLDSSLGWQATERMNISVYVDNLFDRRYRTYGYMNGSSAVAQVNMGRTVGINTRIDFF

>4EPCA

GSTTSTKPSQPSKPSGGTNNKLTVSANRGVAQIKPTNNGLYTTVYDSKGHKTDQVQKTLSVTKTATLGNNKFYLVEDYNSGKKYGWVKQGDVVYNTAKAPVKVNQTYNVKAGSTLYTVPWGTPKQVASKVSGTGNQTFKATKQQQIDKATYLYGTVNGKSGWISKYYLTTASKPSNPTKPSTNNQLTVTNNSGVAQINAKNSGLYTTVYDTKGKTTNQIQRTLSVTKAATLGDKKFYLVGDYNTGTNYGWVKQDEVIYNTAKSPVKINQTYNVKPGVKLHTVPWGTYNQVAGTVSGKGDQTFKATKQQQIDKATYLYGTVNGKSGWISKYYLTA

>4EOZA

GSNMVKVPECRLADELGGLWENSRFTDCCLCVAGQEFQAHKAILAARSPVFSAMFEHEMEESKKNRVEINDVEPEVFKEMMCFIYTGKAPNLDKMADDLLAAADKYALERLKVMCEDALCSNLSVENAAEILILADLHSADQLKT

>4EODA

MAVTDLSLTNSSLMPTLNPMIQQLALAIAASWQSLPLKPYQLPEDLGYVEGRLEGEKLVIENRCYQTPQFRKMELELAKVGKGLDILHCVMFPEPLYGLPLFGCDIVAGPGGVSAAIADLSPTQSDRQLPAAYQKSLAELGQPEFEQQRELPPWGEIFSEYCLFIRPSNVTEEERFVQRVVDFLQIHCHQSIVAEPLSEAQTLEHRQGQIHYCQQQQKNDKTRRVLEKAFGEAWAERYMSQVLFDVIQ

>4EO1A

PSEQTPEEICEAKPPIDGVFNNVFKGDEGGFYINYNGCEYEATGVTVCQNDGTVCSSSAWKPTGYVPESG

>4EN6B

QTILPYPNGLYVINKGDGYMRTNDKDLIGTLLIESSTSGSIIQPRLRNTTRPLFNTSNPTIFSQEYTEARLNDAFNIQLFNTSTTLFKFVEEAPTNKNISMKVYNTYEKYELINYQNGNIDDKAEYYLPSLGKCEVSDAPSPQAPVVETPVDQDGFIQTGPNENIIVGVINPSENIEEISTPIPDDYTYNIPTSIQNNACYVLFKVNTTGVYKITTKNNLPPLIIYEAIGSSNRNMNSNNLSNDNIKAIKYITGLNRSDAKSYLIVSLFKDKNYYIRIPQISSSTTSQLIFKRELGNISDLADSTVNILDNLNTSGTHYYTRQSPDVGNYISYQLTIPGDFNNIASSIFSFRTRNNQGIGTLYRLTESINGYNLITINNYSDLLNNVEPISLLNGATYIFRVKVTELNNYNIIFDAYRNS

>4EMOA

GSMAPPAGGAAAAASDLGSAAVLMAVHAAVRPLGAGPDAEAQLRRLQLSADPERPGRFRLELLGAGPGAVNLEWPLESVSYTIRGPTQHELQPPPGGPGTLSMHFLNPQEAQRWAVLVRGATVEGQNGS

>4AQ1A

MASFTDVAPQYKDAIDFLVSTGATKGKTETKFGVYDEITRLDAAVILARVLKLDVGNAKDAGFTDVPKDRAKYVNALVEAGVLNGKAPGKFGAYDPLTRVEMAKIIANAHKLKADDVKLPFTDVNDTWAPYVKALYKYEVTKGKTPTSFGAYQNITRGDFAQFVYRAVNINAVPEIVEVTAVNSTTVKVTFNTQIADVDFTNFAIDNGLTVTKATLSRDKKSVEVVVNKPFTRNQEYTITATGIKNLKGETAKELTGKFVWSVQDAVTVALNNSSLKVGEESGLTVKDQDGKDVVGAKVELTSSNTNIVVVSSGEVSVSAAKVTAVKPGTADVTAKVTLPDGVVLTNTFKVTVTEVPVQVQNQGFTLVDNLSNAPQNTVAFNKAEKVTSMFAGETKTVAMYDTKNGDPETKPVDFKDATVRSLNPIIATAAINGSELLVTANAGQSGKASFEVTFKDNTKRTFTVDVKKEPVLQDIKVDATSVKLSDEAVGGGEVEGVNQKTIKVSAVDQYGKEIKFGTKGKVTVTTNTEGLVIKNVNSDNTIDFDSGNSATDQFVVVATKDKIVNGKVEVKYFKNASDTTPTSTKTITVNVVNVKADATPVGLDIVAPSEIDVNAPNTASTADVDFINFESVEIYTLDSNGNRLKKVTPTATTLVGTNDYVEVNGNVLQFKGNDELTLLTSSSTVNVDVTADGITKRIPVKYINSASVPASATVATSPVTVKLNSSDNDLTFEELIFGVIDPTQLVKDEDINEFIAVSKAAKNDGYLYNKPLVTVKDASGEVIPTGANVYGLNHDATNGNIWFDEEQAGLAKKFSDVHFDVDFSLANVVKTGSGTVSSSPSLSDAIQLTNSGDAVSFTLVIKSIYVKGADKDDNNLLAAPVSVNVTVTKGS

>4EMZB

MGGKWSKSSVIGWPAVRERMRRAEPAADGVGAVSRDLEKHGAITSSNTAANNAACAWLEAQEEEEVGFPVTPQVPLRPMTYKAAVDLSHFLKEKGGLEGLIHSQRRQDILDLWIYHTQGYFPDWQNYTPGPGVRYPLTFGWCYKLVPVEPDKVEEANKGENTSLLHPVSLHGMDDPEREVLEWRFDSRLAFHHVARELHPEYFKNC

>4EMKB

MDSSPNEFLNKVIGKKVLIRLSSGVDYKGILSCLDGYMNLALERTEEYVNGKKTNVYGDAFIRGNNVLYVSALDD

>4EMCC

QKKRFLPQSVLIKREDEIAFDDFHLDARKVLNDLSATSENPFSSSPNTKKIKSKGKTLEVVPKKKNKKII

>4ELLA

GEFNTIQQLMMILNSASDQPSENLISYFNNCTVNPKESILKRVKDIGYIFKEKFAKAVGQGCVEIGSQRYKLGVRLYYRVMESMLKSEEERLSIQNFSKLLNDNIFHMSLLACALEVVMATYSRSTSQNLDSGTDLSFPWILNVLNLKAFDFYKVIESFIKAEGNLTREMIKHLERCEHRIMESFAWLSDSPLFDLIKQSKDREGPTDHLESACPLNLPLQNNHTAADMYLEPVRAPKKKGSTTRVNSTANAETQATSAFQTQKPLKSTSLSLFYKKVYRLAYLRLNTLCERLLSEHPELEHIIWTLFQHTLQNEYELMRDRHLDQIMMCSMYGICKVKNIDLKFKIIVTAYKDLPHAVQETFKRVLIKEEEYDSIIVFYNSVFMQRLKTNILQYASTRPPTLAPIPHIPR

>3VRCA

ADLSPEEQIETRQAGYAFMAWNMGKIKANLEGEYNADQVRAAANVVAAIANSGMGALYGPGTDKNVGAVKTRAKPELFQNLEDVGKLARDLGTAANALAAAAATGEANAVKSAFADVGAACKACHQKYRAD

>3VR8A

MLRAVRALICRIGARRTLSVSSSRLDVSTSNIAQYKVIDHAYDVVIIGAGGAGLRAAMGLGEAGFKTAVVTKMFPTRSHTTAAQGGINAALGSMNPDDWKWHFYDTAKGSDWLGDQNAMHYLTRNAVEAVTELENFGMPFSRTPEGKIYQRSFGGQSNNYGKGGVAKRTCCVADRTGHSMLHTLYGNSLRCHCTFFIEYFALDLLMDKGRCVGVIALCLEDGTIHRFRSKRTIVATGGYGRAYFSCTTAHMNTGDGTALATRAGIALEDLEFIQFHPTGIYGVGCLITEGSRGEGGFLVNSEGERFMERYAPKAKDLASRDVVSRAETIEIMEGRGVGPEKDHIYLQLHHLPAEQLHQRLPGISETAKIFAGVDVTKEPIPVIPTVHYNMGGIPTNYKAQVIKYTKEGGDKIVPGLYACGECACHSVHGANRLGANSLLDAVVFGRACSINIKEELKPDEKIPELPEGAGEESIANLDAVRYANGDVPTAELRLTMQKTMQKHAGVFRRGDILAEGVKKMMDLSKELKRLKTTDRSLIWNSDLTESLELQNLMLNATQTIVAAENRKESRGAHARDDFPKREDEYDYSKPIEGQTKRPFEKHWRKHTLTKQDPRTGHITLDYRPVIDKTLDPAEVDWIPPIIRSY

>4EJOA

SNAMAYDDIVSSMVLELRRGTLVMLVLSQLREPAYGYALVKSLADHGIPIEANTLYPLMRRLESQGLLASEWDNGGSKPRKYYRTTDEGLRVLREVEAQWHVLCDGVGKLLETNGEDREHAER

>4EJ7A

MGSSHHHHHHSSGRENLYFQGMSHIQRETSCSRPRLNSNLDADLYGYRWARDNVGQSGATIYRLYGKPNAPELFLKHGKGSVANDVTDEMVRLNWLTAFMPLPTIKHFIRTPDDAWLLTTAIPGKTAFQVLEEYPDSGENIVDALAVFLRRLHSIPVCNCPFNSDRVFRLAQAQSRMNNGLVDASDFDDERNGWPVEQVWKEMHKLLPFSPDSVVTHGDFSLDNLIFDEGKLIGCIDVGRVGIADRYQDLAILWNCLGEFSPSLQKRLFQKYGIDNPDMNKLQFHLMLDEFF

>4EIYA

MKTIIALSYIFCLVFADYKDDDDGAPPIMGSSVYITVELAIAVLAILGNVLVCWAVWLNSNLQNVTNYFVVSLAAADIAVGVLAIPFAITISTGFCAACHGCLFIACFVLVLTQSSIFSLLAIAIDRYIAIRIPLRYNGLVTGTRAKGIIAICWVLSFAIGLTPMLGWNNCGQPKEGKNHSQGCGEGQVACLFEDVVPMNYMVYFNFFACVLVPLLLMLGVYLRIFLAARRQLADLEDNWETLNDNLKVIEKADNAAQVKDALTKMRAAALDAQKATPPKLEDKSPDSPEMKDFRHGFDILVGQIDDALKLANEGKVKEAQAAAEQLKTTRNAYIQKYLERARSTLQKEVHAAKSLAIIVGLFALCWLPLHIINCFTFFCPDCSHAPLWLMYLAIVLSHTNSVVNPFIYAYRIREFRQTFRKIIRSHVLRQQEPFKAHHHHHHHHHH

>2LRKA

AEELEEVVMGLIINSGQARSLAYAALKQAKQGDFAAAKAMMDQSRMALNEAHLVQTKLIEGDAGEGKMKVSLVLVEAQLHLMTSMLARELITELIELHEKLKA

>2LRKD

MFQQEVTITAPNGLHTRPAAQFVKEAKGFTSEITVTSNGKSASAKSLFKLQTLGLTQGTVVTISAEGEDEQKAVEHLVKLMAELE

>4EIVA

MHHHHHHENLYFQGGTIYKQFTSRTLLNFFEVAALTDGETNESVAAVCKIAAKDPAIVGVSVRPAFVRFIRQELVKSAPEVAGIKVCAAVNFPEGTGTPDTVSLEAVGALKDGADEIECLIDWRRMNENVADGESRIRLLVSEVKKVVGPKTLKVVLSGGELQGGDIISRAAVAALEGGADFLQTSSGLGATHATMFTVHLISIALREYMVRENERIRVEGINREGAAVRCIGIKIEVGDVHMAETADFLMQMIFENGPRSIVRDKFRVGGGFNLLKELRDCYESWDSVGVSPDTSP

>4APMA

GSAMGSTPKDIWGRYMAKFDLAKSHGSGIYVDLGGTERVGATQHRMPTGKCPVMGKVINLGNNADFLNRISAENPQDRGLAFPDTAVAVTRNSNARNRAAAEKTEIILSPVSAADLVRWGYDGNDVANCAEYAGNIIPASDTATKYRYPFVYDAKEEMCHILFTPMQYNRGSRYCDNDGSQDEGTSSLLCMEPMKSGIDAHLYYGSSRVDKKWEENCPMYPVKDAIFGRGANGSCVAIESAFEEFTRDAEECSALMFENAAADLEIDEEADNFDELKTLSDGLRNIKASKIAQALFSPIAKAGTSAKNSKGVGMNWANYDSNTGLCRVIEETPNCLIIDAGSFAMTAVGSPLEQDAVPFPCDIVTNGYIEPRPRSRHRNTTPIFEVTTALSREALKCSKYVHEKYSESCGTYYYCSEEKPSSWAFWRNLDAAALVPR

>2LRJA

GSSISHSGNLYTAGQCTWYVYDKVGGEIGSTWGNANNWAAAAQGAGFTVNHTPSKGAILQSSEGPFGHVAYVESVNSDGSVTISEMNYSGGPFSVSSRTISASEAGNYNYIHI

>4EFOA

GPLGSTSDILHRMVIHVFSLQQMTAHKIYIHSYNTATIFHELVYKQTKIISSNQELIYEGRRLVLEPGRLAQHFPKTTEENPIFVVSLERPHRD

>4EFZA

GPGSMTVEGFFDPATCTISYLLFDSGSGECALIDSVLDYDPKSGRTRTASADQLIARVAALGARVRWLLETHVHADHLSAAPYLKTRVGGEIAIGRHVTRVQDVFGKLFNAGPAFAHDGSQFDRLLDDGDTLALGALSIRAMHTPGHTPACMTYVVTEAHAAHDARDAAAFVGDTLFMPDYGTARCDFPGGDARSLYRSIRKVLSLPPATRLYMCHDYQPNGRAIQYASTVADELRENVHIREGVTEDDFVAMRTARDATLDMPVLMLPSVQVNMRAGRLPEPEDNGVRYLKIPLDAI

>4AOWA

MHHHHHHSSGVDLGTENLYFQSMMTEQMTLRGTLKGHNGWVTQIATTPQFPDMILSASRDKTIIMWKLTRDETNYGIPQRALRGHSHFVSDVVISSDGQFALSGSWDGTLRLWDLTTGTTTRRFVGHTKDVLSVAFSSDNRQIVSGSRDKTIKLWNTLGVCKYTVQDESHSEWVSCVRFSPNSSNPIIVSCGWDKLVKVWNLANCKLKTNHIGHTGYLNTVTVSPDGSLCASGGKDGQAMLWDLNEGKHLYTLDGGDIINALCFSPNRYWLCAATGPSIKIWDLEGKIIVDELKQEVISTSSKAEPPQCTSLAWSADGQTLFAGYTDNLVRVWQVTIGTR

>4EFIA

MSSPDFSAGRELRTQGARIAGVVSCVPSKQVDNDYFVERFDASAVRDVVKMIGVNRRRWADAQTSAGDLCRKAGEKLLAGLGWQADSIDALIFVSQTPNYRLPATAFVLQAELDLPASCLALDINLGCSGYPQALWLGMNLIQTGAAKRVLLAVGDTISKMIDPTDRSTSLLFGDAGTMTALETSNGDAAAHFIIGADGKGARNLIVPSGGFKPYDAAADERMAGKSPECLFMDGGEIFNFTLNAVPKLVSRTLDIAGRDKDSYDAFLFHQANLFMLKHLAKKAGLPAERVPVNIGEYGNTSCASIPLLITTELKDRLKEETLQLGMFGFGVGYSWASAALAVGPLNIVDTIET

>4EEEA

MECVKTRSVNIHVPVKETSKVVLECRGDSYFRHFSYVYWIIGKNKTVDQLPPNSGYRERIYLFKKPHRCENRPRADLILTNITDEMRNEKLTCVLIDPKDPLKESVILSKIWNCVYKI

>2LRDA

AMGKCSVLKKVACAAAIAGAVAACGGIDLPCVLAALKAAEGCASCFCEDHCHGVCKDLHLC

>4EEIA

MIKRYDVAEISKIWADENKYAKMLEVELAILEALEDRMVPKGTAAEIRARAQIRPERVDEIEKVTKHDIIAFCTSIAEQFTAETGKFFHFGVTSSDIIDSALSLQIRDSMSYVIKDLEALCDSLLTKAEETKEIITMGRSHGMFAEPMSFGQKFLGAYVEFKRRLKDLKDFQKDGLTVQFSGAVGNYCILTTEDEKKAADILGLPVEEVSTQVIPRDRIAKLISIHGLIASAIERLAVEIRHLHRSDVFEVYEGFSKGQKGSSTMPHKKNPISTENLTGMARMLRSHVSIALENCVLWHERDISHSSAERFYLPDNFGIMVYALRRMKNTIDNLVVQRDIIEDRVRSTSAYLSSFYLHFLVANTPFMREDCYKIVQQVAFDLKQGESFSKKLQKVMHDEHNIILDIPEMDFEGIKKTYLKEIDHVFDRSVKARGENLY

>4EDFA

MFEIKKICCIGAGYVGGPTCSVIAHMCPEIRVTVVDVNESRINAWNSPTLPIYEPGLKEVVESCRGKNLFFSTNIDDAIKEADLVFISVNTPTETYGMGKGRAADLKYIEACARRIVQNSNGYKIVTEKSTVPVRAAESIRRIFDANTKPNLNLQVLSNPEFLAEGTAIKDLKNPDRVLIGGDETPEGQRAVQALCAVYEHWVPREKILTTNTWSSELSKLAANAFLAQRISSINSISALCEATGADVEEVATAIGMDQRIGNKFLKASVGFGGSCFQKDVLNLVYLCEALNLPEVARYWQQVIDMNDYQRRRFASRIIDSLFNTVTDKKIAILGFAFKKDTGDTRESSSIYISKYLMDEGAHLHIYDPKVPREQIVVDLSHPGVSEDDQVSRLVTISKDPYEACDGAHAVVICTEWDMFKELDYERIHKKMLKPAFIFDGRRVLDGLHNELQTIGFQIETIGKKVSSKRIPYAPSGEIPKFSLQDPPNKKPKV

>3VQKA

MYYLGKELQKRSEELSRGFYELVYPPVDMYEEGGYLVVVADLAGFNKEKIKARVSGQNELIIEAEREITEPGVKYLTQRPKYVRKVIRLPYNVAKDAEISGKYENGVLTIRIPIAGTSVIKIE

>4EBBA

PDPGFQERFFQQRLDHFNFERFGNKTFPQRFLVSDRFWVRGEGPIFFYTGNEGDVWAFANNSAFVAELAAERGALLVFAEHRYYGKSLPFGAQSTQRGHTELLTVEQALADFAELLRALRRDLGAQDAPAIAFGGSYGGMLSAYLRMKYPHLVAGALAASAPVLAVAGLGDSNQFFRDVTADFEGQSPKCTQGVREAFRQIKDLFLQGAYDTVRWEFGTCQPLSDEKDLTQLFMFARNAFTVLAMMDYPYPTDFLGPLPANPVKVGCDRLLSEAQRITGLRALAGLVYNASGSEHCYDIYRLYHSCADPTGCGTGPDARAWDYQACTEINLTFASNNVTDMFPDLPFTDELRQRYCLDTWGVWPRPDWLLTSFWGGDLRAASNIIFSNGNLDPWAGGGIRRNLSASVIAVTIQGGAHHLDLRASHPEDPASVVEARKLEATIIGEWVKAARREQQPALRGGPRLSLENLYFQ

>4EAZA

DPNSMRSIASSKLWMLEFSAFLERQQDPDTYNKHLFVHISQSSPSYSDPYLETVDIRQIYDKFPEKKGGLKELFERGPSNAFFLVKFWADLNTNIDDEGSAFYGVSSQYESPENMIITCSTKVCSFGKQVVEKVETEYARYENGHYLYRIHRSPLCEYMINFIHKLKHLPEKYMMNSVLENFTILQVVTNRDTQETLLCIAYVFEVSASEHGAQHHIYRLVKE

>4EAGC

MESVAAESAPAPENEHSQETPESNSSVYTTFMKSHRCYDLIPTSSKLVVFDTSLQVKKAFFALVTNGVRAAPLWDSKKQSFVGMLTITDFINILHRYYKSALVQIYELEEHKIETWREVYLQDSFKPLVCISPNASLFDAVSSLIRNKIHRLPVIDPESGNTLYILTHKRILKFLKLFITEFPKPEFMSKSLEELQIGTYANIAMVRTTTPVYVALGIFVQHRVSALPVVDEKGRVVDIYSKFDVINLAAEKTYNNLDVSVTKALQHRSHYFEGVLKCYLHETLEAIINRLVEAEVHRLVVVDEHDVVKGIVSLSDILQALVLTGGEKKP

>4E8JA

MKNNNVTEKELFYILDLFEHMKVTYWLDGGWGVDVLTGKQQREHRDIDIDFDAQHTQKVIQKLEDIGYKIEVHWMPSRMELKHEEYGYLDIHPINLNDDGSITQANPEGGNYVFQNDWFSETNYKDRKIPCISKEAQLLFHSGYDLTETDHFDIKNLKSIT

>4E88A

MGGRALRVLVDMDGVLADVEGGLLRKFRARFPDQPFIALEDRRGYKACEQYGRLRPGLSEKARSIAESKNFFFELEPLPGAVEAVKEMASLQNTDVFICTSPHKMFKYCPYEKYAWVEKYFGPDFLEQIVLTRDKTVVSADLLIDDRPDITGAEPTPSWEHVLFTACHNQHLQLQPPRRRLHSWADDWKAILDSKRPCGSLEHHHHHH

>4AN6A

DYTVHDTDGKPVLNNAGQYYILPAKQGKGGGLGLSNDDDGNCPLTVSQTPIDLPIGLPVRFSSRARISHITTALSLNIEFTIAPACAPKPARWRIFNEQSSEKGYTPVKISDDFSSAAPFQIKKFEEDYKLVYCSKSESGERKCVDLGIKIDDEKNRRLVLKEGDPFKVKFKKVDEESSEEWSIV

>4E4TA

MAHHHHHHMGTLEAQTQGPGSMTATPDSVSPILPGAWLGMVGGGQLGRMFCFAAQSMGYRVAVLDPDPASPAGAVADRHLRAAYDDEAALAELAGLCEAVSTEFENVPAASLDFLARTTFVAPAGRCVAVAQDRIAEKRFIEASGVPVAPHVVIESAAALAALDDAALDAVLPGILKTARLGYDGKGQVRVSTAREARDAHAALGGVPCVLEKRLPLKYEVSALIARGADGRSAAFPLAQNVHHNGILALTIVPAPAADTARVEEAQQAAVRIADTLGYVGVLCVEFFVLEDGSFVANEMAPRPHNSGHYTVDACATSQFEQQVRAMTRMPLGNPRQHSPAAMLNILGDVWFPNGAAAGAVTPPWDTVAAMPAAHLHLYGKEEARVGRKMGHVNFTAEMRDDAVAAATACAQLLRVPLD

>4E51A

MAHHHHHHMGTLEAQTQGPGSMTEQKRKLEKLTGVKGMNDILPQDAGLWEFFEATVKSLLRAYGYQNIRTPIVEHTPLFTRGIGEVTDIVEKEMYSFVDALNGENLTLRPENTAAVVRAAIEHNMLYDGPKRLWYIGPMFRHERPQRGRYRQFHQVGVEALGFAGPDADAEIVMMCQRLWEDLGLTGIKLEINSLGLAEERAAHRVELIKYLEQHADKLDDDAQRRLYTNPLRVLDTKNPALQEIVRNAPKLIDFLGDVSRAHFEGLQRLLKANNVPFTINPRLVRGLDYYNLTVFEWVTDKLGAQGTVAAGGRYDPLIEQLGGKPTAACGWAMGIERILELLKEEHLVPEQEGVDVYVVHQGDAAREQAFIVAERLRDTGLDVILHCSADGAGASFKSQMKRADASGAAFAVIFGEDEVTNGTASVKPLRGTGDDGEKSVQQSVPVESLTEFLINAMVATAEDGDD

>2LQOA

MVTAALTIYTTSWCGYCLRLKTALTANRIAYDEVDIEHNRAAAEFVGSVNGGNRTVPTVKFADGSTLTNPSADEVKAKLVKIAGLEHHHHHH

>4E1YA

GSHPFDQAVVKDPTASYVDVKARRTFLQSGQLDDRLKAALPKEYDCTTEATPNPQQGEMVIPRRYLSGNHGPVNPDYEPVVTLYRDFEKISATLGNLYVATGKPVYATCLLNMLDKWAKADALLNYDPKSQSWYQVEWSAATAAFALSTMMAEPNVDTAQRERVVKWLNRVARHQTSFPGGDTSCCNNASYWRGQEATIIGVISKDDELFRWGLGRYVQAMGLINEDGSFVHEMTRHEQSLHYQNYAMLPLTMIAETASRQGIDLYAYKENGRDIHSARKFVFAAVKNPDLIKKYASEPQDTRAFKPGRGDLNWIEYQRARFGFADELGFMTVPIFDPRTGGSGTLLAYKPQG

>4E1JA

MHHHHHHSSGVDLGTENLYFQSMMGGYILAIDQGTTSTRAIVFDGNQKIAGVGQKEFKQHFPKSGWVEHDPEEIWQTVVSTVKEAIEKSGITANDIAAIGITNQRETVVVWDRETGKPIHNAIVWQDRRTAAFCDKLKKKGLEKTFVKKTGLLLDPYFSGTKLNWLLSNVKGAQVRAAKGELCFGTIDTFLIWRLTGGECFCTDATNASRTLLYNIAENAWDDELTEVLRVPKEMLPEVKDCAADFGVTDPSLFGAAIPILGVAGDQQAATIGQACFKPGMLKSTYGTGCFALLNTGKDMVRSKNRLLTTIAYRLDGETTYALEGSIFVAGAAVQWLRDGLKVIKAAPDTGSLAESADPSQEVYLVPAFTGLGAPHWDPDARGAIFGMTRNTGPAEFARAALEAVCYQTRDLLEAMHKDWRRNGNDTVLRVDGGMVASDWTMQRLSDLLDAPVDRPVILETTALGVAWLAGSRAGVWPNQEAFAKSWARDRRFEPHMDEATRKVKLKGWRSAVKRTLIAA

>4E0VA

MNVFFMFSKPGKLADDRNPLEECFRETDYEEFLEIAKNGLSTTSNPKRVVIVGAGMSGLSAAYVLANAGHQVTVLEASERAGGQVKTYRNEKEGWYANLGPMRLPEKHRIVREYIRKFGLQLNEFSQENENAWYFIKNIRKRVGEVNKDPGVLDYPVKPSEVGKSAGQLYEESLQKAVEELRRTNCSYMLNKYDTYSTKEYLLKEGNLSPGAVDMIGDLLNEDSGYYVSFIESLKHDDIFAYEKRFDEIVGGMDKLPTSMYQAIQEKVHLNARVIKIQQDVKEVTVTYQTSEKETLSVTADYVIVCTTSRAARRIKFEPPLPPKKAHALRSVHYRSGTKIFLTCTKKFWEDDGIHGGKSTTDLPSRFIYYPNHNFPNGVGVIIAYGIGDDANYFEALDFEDCGDIVINDLSLIHQLPKEEIQAICRPSMIQRWSLDKYAMGGITTFTPYQFQHFSEALTAPVDRIYFAGEYTAQAHGWIASTIKSGPEGLDVNRASE

>4E0IA

MKAIDKMTDNPPQEGLSGRKIIYDEDGKPSRSCNTLLDFQYVTGKISNGLKNLSSNGKLAGTGALTGEASELMPGSRTYRKVDPPDVEQLGRSSWTLLHSVAASYPAQPTDQQKGEMKQFLNIFSHIYPCNWSAKDFEKYIRENAPQVESREELGRWMCEAHNKVNKKLRKPKFDCNFWEKRWKDGWDE

>4DYLA

SMGFSSELCSPQGHGVLQQMQEAELRLLEGMRKWMAQRVKSDREYAGLLHHMSLQDSGGQSRAISPDSPISQSWAEITSQTEGLSRLLRQHAEDLNSGPLSKLSLLIRERQQLRKTYSEQWQQLQQELTKTHSQDIEKLKSQYRALARDSAQAKRKYQEASKDKDRDKAKDKYVRSLWKLFAHHNRYVLGVRAAQLHHQHHHQLLLPGLLRSLQDLHEEMACILKEILQEYLEISSLVQDEVVAIHREMAAAAARIQPEAEYQGFLRQYGSAPDVPPCVTFDESLLEEGEPLEPGELQLNELTVESVQHTLTSVTDELAVATEMVFRRQEMVTQLQQELRNEEENTHPRERVQLLGKRQVLQEALQGLQVALCSQAKLQAQQELLQTKLEHLGPGEPPPVLLLQDD

>4DXRA

GPGGSGGVTEEQVHHIVKQALQRYSEDRIGLADYALESGGASVISTRCSETYETKTALLSLFGIPLWYHSQSPRVILQPDVHPGNCWAFQGPQGFAVVRLSARIRPTAVTLEHVPKALSPNSTISSAPKDFAIFGFDEDLQQEGTLLGKFTYDQDGEPIQTFHFQAPTMATYQVVELRILTNWGHPEYTCIYRFRVHGEPAH

>3VP7A

INIFNATFKISHSGPFATINGLRLGSIPESVVPWKEINAALGQLILLLATINKNLKINLVDYELQPMGSFSKIKKRMVNSVEYNNSTTNAPGDWLILPVYYDENFNLGRIFRKETKFDKSLETTLEIISEITRQLSTIASSYSSQTLTTSQDESSMNNANDVENSTSILELPYIMNKDKINGLSVKLHGSSPNLEWTTAMKFLLTNVKWLLAFSSNLLSK

>4DXDA

MHHHHHHLEFEQGFNHLATLKVIGVGGGGNNAVNRMIDHGMNNVEFIAINTDGQALNLSKAESKIQIGEKLTRGLGAGANPEIGKKAAEESREQIEDAIQGADMVFVTSGMGGGTGTGAAPVVAKIAKEMGALTVGVVTRPFSFEGRKRQTQAAAGVEAMKAAVDTLIVIPNDRLLDIVDKSTPMMEAFKEADNVLRQGVQGISDLIAVSGEVNLDFADVKTIMSNQGSALMGIGVSSGENRAVEAAKKAISSPLLETSIVGAQGVLMNITGGESLSLFEAQEAADIVQDAADEDVNMIFGTVINPELQDEIVVTVIATGFDDKPTSHGRKSGSTGFGTSVNTSSNATSKDESFTSNSSNAQATDSVSERTHTTKEDDIPSFIRNREERRSRRTRR

>4DX1A

MFNRPILFDIVSRGSTADLDGLLPFLLTHKKRLTDEEFREPSTGKTCLPKALLNLSNGRNDTIPVLLDIAERTGNMREFINSPFRDIYYRGQTALHIAIERRCKHYVELLVAQGADVHAQARGRFFQPKDEGGYFYFGELPLSLAACTNQPHIVNYLTENPHKKADMRRQDSRGNTVLHALVAIADNTRENTKFVTKMYDLLLLKCARLFPDSNLEAVLNNDGLSPLMMAAKTGKIGIFQHIIRREVTDEAAAHHHHHH

>4AKKA

MRGSHHHHHHTDPHASSVPGRGSIEGRMNNMAGNTPEVVDWFARARRLQKQQLHQLAQQGTLAGQISALVHMLQCERGASNIWLCSGGRLYAAECRAGAALVDEQLTRFYAALEPARDAASSALCWRIACAVWYLPQLAALRKRVRDREIAAEEATGQFSRIIRHLLNIVPQLNDSIDDPQIAGRMVALYSFMQGKELAGQERALGALGFARGQFSDELRQQLVDRIDGQQPCFDSFQALAQPPQTALFAEQCQASLEIEQLRRVACTRQPPADEGETALRWFCAQTQRLEQLRGVEELLIVDLLNAADALLEGEEPEAQLPPADWQEDSIALRLDKQLLPLVRQQAHELQQLSGQLASLKDALEERKLIEKAKSVLMTYQGMQEEQAWQALRKMAMDKNQRMVEIARALLTVKALWRVTPKE

>4DW0A

GSSKKVGTLNRFTQALVIAYVIGYVFVYNKGYQDTDTVLSSVTTKVKGIALTKTSELGERIWDVADYIIPPQEDGSFFVLTNMIITTNQTQSKCAENPTPASTCTSHRDCKRGFNDARGDGVRTGRCVSYSASVKTCEVLSWCPLEKIVDPPNPPLLADAERFTVLIKNNIRYPKFNFNKRNILPNINSSYLTHCVFSRKTDPDCPIFRLGDIVGEAEEDFQIMAVRGGVMGVQIRWDCDLDMPQSWCVPRYTFRRLDNKDPDNNVAPGYNFRFAKYYKNSDGTETRTLIKGYGIRFDVMVFGQAGKFNIIPTLLNIGAGLALLGLVNVICDWIVLTFMK

>4DT4A

MGSSHHHHHHSSGLVPRGSHMSESVQSNSAVLVHFTLKLDDGTTAESTRNNGKPALFRLGDASLSEGLEQHLLGLKVGDKTTFSLEPDAAFGVPSPDLIQYFSRREFMDAGEPEIGAIMLFTAMDGSEMPGVIREINGDSITVDFNHPLAGQTVHFDIEVLEIDPALEA

>2LPIA

GSGNSHTTPWTNPGLAENFMNSFMQGLSSMPGFTASQLDDMSTIAQSMVQSIQSLAAQGRTSPNKLQALNMRFASSMAEIAASEEGGGSLSTKTSSIASAMSNAFLQTTGVVNQPFINEITQLVSMFAQAGMNDVSA

>4DPPA

MHHHHHHGLPIPNPLLGLDSTENLYFQGIDPFTAAVVPNFHLPMRSLEVKNRTNTDDIKALRVITAIKTPYLPDGRFDLEAYDDLVNIQIQNGAEGVIVGGTTGEGQLMSWDEHIMLIGHTVNCFGGSIKVIGNTGSNSTREAIHATEQGFAVGMHAALHINPYYGKTSIEGLIAHFQSVLHMGPTIIYNVPGRTGQDIPPRAIFKLSQNPNLAGVKECVGNKRVEEYTENGVVVWSGNDDECHDSRWDYGATGVISVTSNLVPGLMRKLMFEGRNSSLNSKLLPLMAWLFHEPNPIGINTALAQLGVSRPVFRLPYVPLPLSKRLEFVKLVKEIGREHFVGEKDVQALDDDDFILIGRY

>4DOTA

MRAPIPEPKPGDLIEIFRPFYRHWAIYVGDGYVVHLAPPSEVAGAGAASVMSALTDKAIVKKELLYDVAGSDKYQVNNKHDDKYSPLPCSKIIQRAEELVGQEVLYKLTSENCEHFVNELRYGVARSDQVRDLEHHHHHH

>4DOJA

LENPTNLEGKLADAEEEIILEGEDTQASLNWSVIVPALVIVLATVVWGIGFKDSFTNFASSALSAVVDNLGWAFILFGTVFVFFIVVIAASKFGTIRLGRIDEAPEFRTVSWISMMFAAGMGIDLMFYGTTEPLTFYRNGVPGHDEHNVGVAMSTTMFHWTLHPWAIYAIVGLAIAYSTFRVGRKQLLSSAFVPLIGEKGAEGWLGKLIDILAIIATVFGTACSLGLGALQIGAGLSAANIIEDPSDWTIVGIVSVLTLAFIFSAISGVGKGIQYLSNANMVLAALLAIFVFVVGPTVSILNLLPGSIGNYLSNFFQMAGRTAMSADGTAGEWLGSWTIFYWAWWISWSPFVGMFLARISRGRSIREFILGVLLVPAGVSTVWFSIFGGTAIVFEQNGESIWGDGAAEEQLFGLLHALPGGQIMGIIAMILLGTFFITSADSASTVMGTMSQHGQLEANKWVTAAWGVATAAIGLTLLLSGGDNALSNLQNVTIVAATPFLFVVIGLMFALVKDLSNDVIYLEYREQQRFNARLARERRVHNEHRKRELAAKRRRERKASGAGKRR

>4AIEA

MASASWWKNAVVYQVYPKSFQDSNGDGIGDLQGIISRLDYLEKLGIDAIWLSPVYQSPGVDNGYDISDYEAIDPQYGTMADMDELISKAKEHHIKIVMDLVVNHTSDQHKWFVEAKKGKDNQYRDYYIWRDPVDEHEPNDLKSAFSGSAWKYDERSGQYYLHFFADQQPDLNWQNTELRQKIYNMMNFWLDKGIGGFRMDVIELIGKDPDKNIRENGPMLHPYLQEMNKATFGKRDVMTVGETWNATPKIAEEYSDPDRHELSMVFQFENQSLDQQPGKEKWDLKPLDLGELKKVLVKWQTKIDFDHAWNSLFWENHDIPRVISRWGNDQEYRVQCAKMFAIILHMMHGTPYIFNGEEIGMTNCPVKNIDEVEDIESINMYNERLAEGYDEEELIHAINVKGRDNARRPMQWNDEKNAGFSEVDPWLSVNPNYKDINVENALADPNSIFYTYQKLIKLRHENPIVVDGDFSLVSNTQDAVLAYYRILNDKKWLVVANLSNEEQNFVSNDQIETILSNYPERNNVQNITLKPYEAFISKVIELEHHHHHH

>4DMUB

APDCSQPLDVILLLDGSSSFPASYFDEMKSFAKAFISKANIGPRLTQVSVLQYGSITTIDVPWNVVPEKAHLLSLVDVMQREGGPSQIGDALGFAVRYLTSEMHGARPGASKAVVILVTDVSVDSVDAAADAARSNRVTVFPIGIGDRYDAAQLRILAGPAGDSNVVKLQRIEDLPTMVTLGNSFLHKLCSG

>4DM3A

MSGADRSPNAGAAPDSAPGQAAVASAYQRFEPRAYLRNNYAPPRGDLCNPNGVGPWKLRCLAQTFATGEVSGRTLIDIGSGPTVYQLLSACSHFEDITMTDFLEVNRQELGRWLQEEPGAFNWSMYSQHACLIEGKGECWQDKERQLRARVKRVLPIDVHQPQPLGAGSPAPLPADALVSAFCLEAVSPDLASFQRALDHITTLLRPGGHLLLIGALEESWYLAGEARLTVVPVSEEEVREALVRSGYKVRDLRTYIMPAHLQTGVDDVKGVFFAWAQKVGLEHHHHHH

>4DLFA

MGALRIDSHQHFWRYRAADYPWIGAGMGVLARDYLPDALHPLMHAQALGASIAVQARAGRDETAFLLELACDEARIAAVVGWEDLRAPQLAERVAEWRGTKLRGFRHQLQDEADVRAFVDDADFARGVAWLQANDYVYDVLVFERQLPDVQAFCARHDAHWLVLDHAGKPALAEFDRDDTALARWRAALRELAALPHVVCKLSGLVTEADWRRGLRASDLRHIEQCLDAALDAFGPQRLMFGSDWPVCLLAASYDEVASLVERWAESRLSAAERSALWGGTAARCYALPEPADARLAENLYFQ

>4DJSA

LATRAIPELTKLLNDEDQVVVNKAAVMVHQLSKKEASRHAIMRSPQMVSAIVRTMQNTNDVETARCTAGTLHNLSHHREGLLAIFKSGGIPALVKMLGSPVDSVLFYAITTLHNLLLHQEGAKMAVRLAGGLQKMVALLNKTNVKFLAITTDCLQILAYGNQESKLIILASGGPQALVNIMRTYTYEKLLWTTSRVLKVLSVCSSNKPAIVEAGGMQALGLHLTDPSQRLVQNCLWTLRNLSDAATKQEGMEGLLGTLVQLLGSDDINVVTCAAGILSNLTCNNYKNKMMVCQVGGIEALVRTVLRAGDREDITEPAICALRHLTSRHQEAEMAQNAVRLHYGLPVVVKLLHPPSHWPLIKATVGLIRNLALCPANHAPLREQGAIPRLVQLLVRAHQDTQRRTSMGGTQQQFVEGVRMEEIVEGCTGALHILARDVHNRIVIRGLNTIPLFVQLLYSPIENIQRVAAGVLCELAQDKEAAEAIEAEGATAPLTELLHSRNEGVATYAAAVLFRMSED

>4DJTA

GPGSMERRELTYKICLIGDGGVGKTTYINRVLDGRFEKNYNATVGAVNHPVTFLDDQGNVIKFNVWDTAGQEKKAVLKDVYYIGASGAILFFDVTSRITCQNLARWVKEFQAVVGNEAPIVVCANKIDIKNRQKISKKLVMEVLKGKNYEYFEISAKTAHNFGLPFLHLARIFTGRPDLIFVSNVNLEPTEVNYDYHSPEESKYIDYMEQASKMAPEE

>4DJBA

MGSSHHHHHHSQDPMIRCLRLKVEGALEQIFTMAGLNIRDLLRDILRRWRDENYLGMVEGAGMFIEEIHPEGFSLYVHLDVRAVSLLEAIVQHLTEAIISSLAVEFDHATGGERVHLIDLHFEVLDNLLE

>4DIXA

GPSSSKSEENISLVYEIDGTEALGSCLRVRPCSNDAPDLSKCTIQWYRSSSDGSKKELISGATKSVYAPEPFDVGRVLHADIIYDGHSLSLSTVGKIDPAAGLGSYVEALVRKHDVDFNVVVTQMSGEDHTSESIHLFHVGKMRIKLCKGKTVIAKEYYSSAMQLCGVRGGGNAAAQALYWQAKKGVSFVIAFESERERNAAIMLARRFACDCNVTLAGPEDRTETGQSP

>4DIPA

YFQSMGALIPEPEVKIEVLQKPFICHRKTKGGDLMLVHYEGYLEKDGSLFHSTHKHNNGQPIWFTLGILEALKGWDQGLKGMCVGEKRKLIIPPALGYGKEGKGKIPPESTLIFNIDLLEIRNGP

>4DIQA

LRRRYTMASGPQVDNTGGEPAWDSPLRRVLAELNRIPSSRRRAARLFEWLIAPMPPDHFYRRLWEREAVLVRRQDHTYYQGLFSTADLDSMLRNEEVQFGQHLDAARYINGRRETLNPPGRALPAAAWSLYQAGCSLRLLCPQAFSTTVWQFLAVLQEQFGSMAGSNVYLTPPNSQGFAPHYDDIEAFVLQLEGRKLWRVYRPRAPTEELALTSSPNFSQDDLGEPVLQTVLEPGDLLYFPRGFIHQAECQDGVHSLHLTLSTYQRNTWGDFLEAILPLAVQAAMEENVEFRRGLPRDFMDYMGAQHSDSKDPRRTAFMEKVRVLVARLGHFAPVDAVADQRAKDFIHDSLPPVLTDRERALSVYGLPIRWEAGEPVNVGAQLTTETEVHMLQDGIARLVGEGGHLFLYYTVENSRVYHLEEPKCLEIYPQQADAMELLLGSYPEFVRVGDLPCDSVEDQLSLATTLYDKGLLLTKMPLALNAENLYFQ

>2LOYA

MLIYKDIFTDDELSSDSFPMKLVDDLVYEFKGKHVVRKEGEIVLAGSNPSAEEGAEDDGSDEHVERGIDIVLNHKLVEMNCYEDASMFKAYIKKFMKNVIDHMEKNNRDKADVDAFKKKIQGWVVSLLAKDRFKNLAFFIGERAAEGAENGQVAIIEYRDVDGTEVPTLMLVKEAIIEEKCLEHHHHHH

>2LORA

MVNLGLSRVDDAVAAKHPGLGEYAACQSHAFMKGVFTFVTGTGMAFGLQMFIQRKFPYPLQWSLLVAVVAGSVVSYGVTRVESEKCNNLWLFLETGQLPKDRSTDQRS

>2LONA

MSANRRWWVPPDDEDCVSEKLLRKTRESPLVPIGLGGCLVVAAYRIYRLRSRGSTKMSIHLIHTRVAAQACAVGAIMLGAVYTMYSDYVKRMAQDAGEK

>4DG8A

GHMDSFFRKKAIVRMSQNSLLDLYAHPTVVARFSEMAALHPHREAIRDRFGSVDYRQLLDSAEQLSDYLLEHYPQPGVCLGVYGEYSRESITCLLAILLSGHHYLYIDLKQPAAWNAELCRQVDCRLILDCSTTPTPANGLPCVPVRHLPAAPASVARPCFAADQIAYINFSSGTTGRPKAIACTHAGITRLCLGQSFLAFAPQMRFLVNSPLSFDAATLEIWGALLNGGCCVLNDLGPLDPGVLRQLIGERGADSAWLTASLFNTLVDLDPDCLGGLRQLLTGGDILSVPHVRRALLRHPRLHLVNGYGPTENTTFTCCHVVTDDDLEEDDIPIGKAIAGTAVLLLDEHGQEIAEPDRAGEIVAFGAGLAQGYRNDAARTRASFVELPYRGRLLRAYRTGDRARYDEQGRLRFIGRGDGQVKLNGYRLDLPALEQRFRRQPGILDCALLVRERNGVKQLLCAWTGKADASPQALLRQLPTWQRPHACVRVEALPLTAHGKLDRAALLRRLEEPLERCASALDPDQRGCAQLWSELLGCEVGAADQDFFLCGGNSLLALQLVALCQSAGAGANLGLADLQANSRLDQFSRLLRSHGLAPERLLERAATPEQPLVLSRSAA

>2LOEA

EKVKGCDFTTSESTIFSKGYSINEISNKSSNNQQDIVCTVKAHANDLIGFKCPSNYSVEPHDCFVSAFNLSGKNENLENKLKLTNIIMDHYNNTFYSRLPSLISDNWKFFCVCSKDNEKKLVFTVEA

>4DEPB

EPLEADKCKEREEKIILVSSANEIDVRPCPLNPNEHKGTITWYKDDSKTPVSTEQASRIHQHKEKLWFVPAKVEDSGHYYCVVRNSSYCLRIKISAKFVENEPNLCYNAQAIFKQKLPVAGDGGLVCPYMEFFKNENNELPKLQWYKDCKPLLLDNIHFSGVKDRLIVMNVAEKHRGNYTCHASYTYLGKQYPITRVIEFITLEENKPTRPVIVSPANETMEVDLGSQIQLICNVTGQLSDIAYWKWNGSVIDEDDPVLGEDYYSVENPANKRRSTLITVLNISEIESRFYKHPFTCFAKNTHGIDAAYIQLIYPVTNFQK

>2LOBA

HHHHHHHHHHSSGHIEGRHMENLYFQGIRKVLLLKEDHEGLGISITGGKEHGVPILISEIHPGQPADRCGGLHVGDAILAVNGVNLRDTKHKEAVTILSQQRGEIEFEVVYV

>4AFIA

GSPFYIKSSPSPQKRYQDTPGVEHIPVVQIDLSVPLKVPGLPMSDQYVKLEEAMAILFAVVARGTTILAKHAWCGGNFLEVTEQILAKIPSENNKLTYSHGNYLFHYICQDRIVYLCITDDDFERSRAFSFLNEVKKRFQTTYGSRAQTALPYAMNSEFSSVLAAQLKHHSEN

>4DCNC

GSRTVDLELELQIELLRETKRKYESVLQLGRALTAHLYSLLQTQHALGDAFADLSQKSPELQEEFGYNAETQKLLCKNGETLLGAVNFFVSSINTLVTKTMEDTLMTVKQYEAARLEYDAYRTDLEELSLGPRDAGTRGRLESAQATFQAHRDKYEKLRGDVAIKLKFLEENKIKVMHKQLLLFHNAVSAYFAGNQKQ

>3VO1A

MVSTTETAEAEPVKKLEKVSKKQEEGLVTNKYKPKEPYVGRCLLNTRITGDQAPGETWHMVFSTEGEVPYREGQSIGVIADGEDKNGKPHKLRLYSIASSALGDFGDSKTVSLCVKRLVYTNDQGEVVKGVCSNFLCDLKPGAEVKITGPVGKEMLMPKDPNATIIMLATGTGIAPFRSFLWKMFFEEHEDYKYTGLAWLFLGVPTSDTLLYKEELEKMKEMAPDNFRLDFAVSREQTNAAGEKMYIQTRMAEYKEELWELLKKDNTYVYMCGLKGMEKGIDDIMLDLAAKDGINWLDYKKQLKKSEQWNVEVY

>4DCXA

AAPDEITTAWPVNVGPLNPHLYTPNQMFAQSMVYEPLVKYQADGSVIPWLAKSWTHSEDGKTWTFTLRDDVKFSNGEPFDAEAAAENFRAVLDNRQRHAWLELANQIVDVKALSKTELQITLKSAYYPFLQELALPRPFRFIAPSQFKNHETMNGIKAPIGTGPWILQESKLNQYDVFVRNENYWGEKPAIKKITFNVIPDPTTRAVAFETGDIDLLYGNEGLLPLDTFARFSQNPAYHTQLSQPIETVMLALNTAKAPTNELAVREALNYAVNKKSLIDNALYGTQQVADTLFAPSVPYANLGLKPSQYDPQKAKALLEKAGWTLPAGKDIREKNGQPLRIELSFIGTDALSKSMAEIIQADMRQIGADVSLIGEEESSIYARQRDGRFGMIFHRTWGAPYDPHAFLSSMRVPSHADFQAQQGLADKPLIDKEIGEVLATHDETQRQALYRDILTRLHDEAVYLPISYISMMVVSKPELGNIPYAPIATEIPFEQIKPVKP

>4DCKC

MALLRKSYSEPQLKGIVTKLYSRQGYHLQLQADGTIDGTKDEDSTYTLFNLIPVGLRVVAIQGVQTKLYLAMNSEGYLYTSELFTPECKFKESVFENYYVTYSSMIYRQQQSGRGWYLGLNKEGEIMKGNHVKKNKPAAHFLPKPLKVAMYKEPSLHDLTEFSRSGSGTPTKSRSVSGVLNGGKSMSHNEST

>4DBLC

MSIVMQLQDVAESTRLGPLSGEVRAGEILHLVGPNGAGKSTLLARMAGMTSGKGSIQFAGQPLEAWSATKLALHRAYLSQQQTPPFATPVWHYLTLHQHDKTRTELLNDVAGALALDDKLGRSTNQLSGGEWQRVRLAAVVLQITPQANPAGQLLLLDQPMNSLDVAQQSALDKILSALSQQGLAIVMSSHDLNHTLRHAHRAWLLKGGKMLASGRREEVLTPPNLAQAYGMNFRRLDIEGHRMLISTI

>4D97A

MRGSHHHHHHGMASMPLHHLTRFPRLEFIGAPTPLEYLPRLSDYLGREIYIKRDDVTPIAMGGNKLRKLEFLVADALREGADTLITAGAIQSNHVRQTAAVAAKLGLHCVALLENPIGTTAENYLTNGNRLLLDLFNTQIEMCDALTDPDAQLQTLATRIEAQGFRPYVIPVGGSSALGAMGYVESALEIAQQCEEVVGLSSVVVASGSAGTHAGLAVGLEHLMPDVELIGVTVSRSVAEQKPKVIALQQAIAGQLALTATADIHLWDDYFAPGYGVPNDAGMEAVKLLASLEGVLLDPVYTGKAMAGLIDGISQKRFNDDGPILFIHTGGAPALFAYHPHVTYPE

>4D8QH

MSLRLPQNPNAGLFKQGYNSYSNADGQIIKSIAAIRELHQMCLTSMGPCGRNKIIVNHLGKIIITNDAATMLRELDIVHPAVKVLVMATEQQKIDMGDGTNLVMILAGELLNVSEKLISMGLSAVEIIQGYNMARKFTLKELDEMVVGEITDKNDKNELLKMIKPVISSKKYGSEDILSELVSEAVSHVLPVAQQAGEIPYFNVDSIRVVKIMGGSLSNSTVIKGMVFNREPEGHVKSLSEDKKHKVAVFTCPLDIANTETKGTVLLHNAQEMLDFSKGEEKQIDAMMKEIADMGVECIVAGAGVGELALHYLNRYGILVLKVPSKFELRRLCRVCGATPLPRLGAPTPEELGLVETVKTMEIGGDRVTVFKQEQGEISRTSTIILRGATQNNLDDIERAIDDGVAAVKGLMKPSGGKLLPGAGATEIELISRITKYGERTPGLLQLAIKQFAVAFEVVPRTLAETAGLDVNEVLPNLYAAHNVTEPGAVKTDHLYKGVDIDGESDEGVKDIREENIYDMLATKKFAINVATEAATTVLSIDQIIMAKKAGGPRAPQGPRPGNWDQED

>4D87A

MSNKYRVRKNVLHLTDTEKRDFVRTVLILKEKGIYDRYIAWHGAAGKFHTPPGSDRNAAHMSSAFLPWHREYLLRFERDLQSINPEVTLPYWEWETDAQMQDPSQSQIWSADFMGGNGNPIKDFIVDTGPFAAGRWTTIDEQGNPSGGLKRNFGATKEAPTLPTRDDVLNALKITQYDTPPWDMTSQNSFRNQLEGFINGPQLHNRVHRWVGGQMGVVPTAPNDPVFFLHHANVDRIWAVWQIIHRNQNYQPMKNGPFGQNFRDPMYPWNTTPEDVMNHRKLGYVYDIELRKSKRSSHHHHHH

>4D8KA

GGSNPPASPLQDNLVIALHSYEPSHDGDLGFEKGEQLRILEQSGEWWKAQSLTTGQEGFIPFNFVAKANSLEPEPWFFKNLSRKDAERQLLAPGNTHGSFLIRESESTAGSFSLSVRDFDQNQGEVVKHYKIRNLDNGGFYISPRITFPGLHELVRHYTNASDGLCTRLSRPCQT

>3VF0B

HMLDPEEIRKRLEHTERQFRNRRKILIRGLPGDVTNQEVHDLLSDYELKYCFVDKYKGTAFVTLLNGEQAEAAINAFHQSRLRERELSVQLQPTDALLCVANLPPSLTQQQFEELVRPFGSLERCFLVYSERTGQSKGYGFAEYMKKDSAARAKSDLLGKPLGPRTLYVHWTDAGQLTPALLHSRCLCVDRLPPGFNDVDALCRALSAVHSPTFCQLACGQDGQLKGFAVLEYETAEMAEEAQQQADGLSLGGSHLRVSFCAPGPPGRSMLAALIAAQATALNRG

>3VFDA

ESGAVPKRKDPLTHTSNSLPRSKTVMKTGSAGLSGHHRAPSYSGLSMVSGVKQGSGPAPTTHKGTPKTNRTNKPSTPTTATRKKKDLKNFRNVDSNLANLIMNEIVDNGTAVKFDDIAGQDLAKQALQEIVILPSLRPELFTGLRAPARGLLLFGPPGNGKTMLAKAVAAESNATFFNISAASLTSKYVGEGEKLVRALFAVARELQPSIIFIDQVDSLLCERREGEHDASRRLKTEFLIEFDGVQSAGDDRVLVMGATNRPQELDEAVLRRFIKRVYVSLPNEETRLLLLKNLLCKQGSPLTQKELAQLARMTDGYSGSDLTALAKDAALGPIRELKPEQVKNMSASEMRNIRLSDFTESLKKIKRSVSPQTLEAYIRWNKDFGDTTV

>2LO0A

SVDVAVSAGAGERASAEQKESYEPPKPAVGPSGESVVATEAFWDDLQGFLEQRLKDYDEANKLRVLFKEAWRSSF

>3VE0I

YPYDVPDYAIEGRGARSMPLGVVTNSTLEVTEIDQLVCKDHLASTDQLKSVGLNLEGSGVSTDIPSATKRWGFRSGVPPKVVSYEAGEWAENCYNLEIKKPDGSECLPPPPDGVRGFPRCRYVHKAQGTGPCPGDYAFHKDGAFFLYDRLASTVIYRGVNFAEGVIAFLILAKPKETFLQSPPIREAVNYTENTSSYYATSYLEYEIENFGAQHSTTLFKINNNTFVLLDRPHTPQFLFQLNDTIHLHQQLSNTTGKLIWTLDANINADIGEWAFWENKKNLSEQLRGEELSFETLSL

>4AE2A

ETGHHHHHHSADEPMDFKINTDEIMTSLKSVNGQIESLISPDGSRKNPARNCRDLKFCHPELKSGEYWVDPNQGCKLDAIKVFCNMETGETCISANPLNVPRKHWWTDSSAEKKHVWFGESMDGGFQFSYGNPELPEDVLDVQLAFLRLLSSRASQQITYHCKNSIAYMDQASGNVKKALKLMGSNEGEFKAEGNSKFTYTVLEDGCTKHTGEWSKTVFEYRTRKAVRLPIVDIAPYDIGGPDQEFGVDVGPVCFL

>3VDIA

MALFGTKDTTTAHSDYEIILEGGSSSWGQIKGRAKVNVPAALPLLPADCNIKIEAKPLDAQKGVVRFTSQIESIVDSTKNKLVVEVDIANETKDRRIAVGEGEVSVGDFSHKFSFEGSVVNMYYYRSDAVRRNVPNPVYMQGRQFHDIMMKVPLDNKDLIETWEGFQQSISGGGVNFGDWIREFWFIGPAYTAINEGGQRISPIQVNNFGVESGEKGPVGVSRWKFSHAGSGIVDSISRWAELFPVEQLNKPASIEGGFRSDSQGIEVKVDGNLPGVSRDAGGGLRRILNHPLIPLVHHGMVGKFNDFTVDTQLKVVLPKGYKIRYAAPQFRSQNLEEYRWSGGAYARWVEHVCKGGTGQFEVLYAQ

>3VDJA

YVEFEPSDKHIKEYLNKIQNSLSTEWSPCSVTCGNGIQVRIKPGSANKPKDELDYANDIEKKICKMEKCPHHHHHHA

>4ADZA

GSHMTTTEAGASAPSPAVDGAVNQTARQAEADGTDIVTDHDRGVHGYHKQKAEHLKRLRRIEGQIRGLQRMVDEDVYCIDILTQVSASTKALQSFALQLLEEHLRHCVADAALKGGTEIDAKVEEATKAIGRLLRT

>3VBAA

MRSIIKGRVWKFGNNVDTDAILPARYLVYTKPEELAQFVMTGADPDFPKKVKPGDIIVGGKNFGCGSSREHAPLGLKGAGISCVIAESFARIFYRNAINVGLPLIECKGISEKVNEGDELEVNLETGEIKNLTTGEVLKGQKLPEFMMEILEAGGLMPYLKKKMAESQLEHHHHHH

>3V9OA

GPGSMTGTTMFAALLHPRLADCRRLYLRNHEVYMNIGAFEHEKRGEQRVVINVDLFVPLALTTPVEDKLREVVDYDLMKQSVAQCVARGHIHLQETLCDAIAASLLAHDAVRAVRVSTEKPDAYPDCDAVGVEVFRIKDEERA

>3V9BA

IPRFGVKTEQEDVLAKELEDVNKWGLHVFRIAELSGNRPLTVIMHTIFQERDLLKTFKIPVDTLITYLMTLEDHYHADVAYHNNIHAADVVQSTHVLLSTPALEAVFTDLEILAAIFASAIHDVDHPGVSNQFLINTNSELALMYNDSSVLENHHLAVGFKLLQEENCDIFQNLTKKQRQSLRKMVIDIVLATDMSKHMNLLADLKTMVETKKVTSSGVLLLDNYSDRIQVLQNMVHCADLSNPTKPLQLYRQWTDRIMEEFFRQGDRERERGMEISPMCDKHNASVEKSQVGFIDYIVHPLWETWADLVHPDAQDILDTLEDNREWYQSTIPQSPSPAPDDPEEGRQGQTEKFQFELTL

>2LNAA

MGHHHHHHSHMKRSGREITWKDFVNNYLSKGVVDRLEVVNKRFVRVTFTPGKTPVDGQYVWFNIGSVDTFERNLETLQQELGIEGENRVPVVYIAESDG

>3V65B

TGEENCNVNNGGCAQKCQMIRGAVQCTCHTGYRLTEDGRTCQDVNECAEEGYCSQGCTNSEGAFQCWCEAGYELRPDRRSCKALGPEPVLLFANRIDIRQVLPHRSEYTLLLNNLENAIALDFHHRRELVFWSDVTLDRILRANLNGSNVEEVVSTGLESPGGLAVDWVHDKLYWTDSGTSRIEVANLDGAHRKVLLWQSLEKPRAIALHPMEGTIYWTDWGNTPRIEASSMDGSGRRIIADTHLFWPNGLTIDYAGRRMYWVDAKHHVIERANLDGSHRKAVISQGLPHPFAITVFEDSLYWTDWHTKSINSANKFTGKNQEIIRNKLHFPMDIHTLHPQRQPAGKNRCGDNNGGCTHLCLPSGQNYTCACPTGFRKINSHACAQ

>3V53A

MGHHHHHHMKRKHIKSLIEKIPTAKPELFAYPLDWSIVDSILMERRIRPWINKKIIEYIGEEEATLVDFVCSKVMAHSSPQSILDDVAMVLDEEAEVFIVKMWRLLIYETEAKKIGLVK

>3V57B

MLDAFSRVVVNSDAKAAYVGGSDLQALKSFIADGNKRLDAVNSIVSNASCMVSDAVSGMICENPGLISPGGNCYTNRRMAACLRDGEIILRYVSYALLAGDASVLEDRCLNGLKETYIALGVPTNSSIRAVSIMKAQAVAFITNTATERKMSFAAGDCTSLASEVASYFDRVGAAIS

>3V43A

HMEPIPICSFCLGTKEQNREKKPEELISCADCGNSGHPSCLKFSPELTVRVKALRWQCIECKTCSSCRDQGKNADNMLFCDSCDRGFHMECCDPPLTRMPKGMWICQICRPR

>4AC5C

CFEPPPATTTQTGFRGLSMGEVLHPATVKAKKERDAQYPPALAAVKAEGPPVSQVYKNVKVLGNLTEAEFLRTMTAITEWVSPQEGCTYCHDENNLASEAKYPYVVARRMLEMTRAINTNWTQHVAQTGVTCYTCHRGTPLPPYVRYLEPTLPLNNRETPTHVERVETRSGYVVRLAKYTAYSALNYDPFTMFLANDKRQVRVVPQTALPLVGVSRGKERRPLSDAYATFALMMSISDSLGTNCTFCHNAQTFESWGKKSTPQRAIAWWGIRMVRDLNMNYLAPLNASLPASRLGRQGEAPQADCRTCHQGVTKPLFGASRLKDYPELGPIKAAAK

>3V48A

GHMKLSLSPPPYADAPVVVLISGLGGSGSYWLPQLAVLEQEYQVVCYDQRGTGNNPDTLAEDYSIAQMAAELHQALVAAGIEHYAVVGHALGALVGMQLALDYPASVTVLISVNGWLRINAHTRRCFQVRERLLYSGGAQAWVEAQPLFLYPADWMAARAPRLEAEDALALAHFQGKNNLLRRLNALKRADFSHHADRIRCPVQIICASDDLLVPTACSSELHAALPDSQKMVMPYGGHACNVTDPETFNALLLNGLASLLHHREAAL

>3V2AR

MQSKVLLAVALWLCVETRAASVGLPSVSLDLPRLSIQKDILTIKANTTLQITCRGQRDLDWLWPNNQSGSEQRVEVTECSDGLFCKTLTIPKVIGNDTGAYKCFYRETDLASVIYVYVQDYRSPFIASVSDQHGVVYITENKNKTVVIPCLGSISNLNVSLCARYPEKRFVPDGNRISWDSKKGFTIPSYMISYAGMVFCEAKINDESYQSIMYIVVVVGYRIYDVVLSPSHGIELSVGEKLVLNCTARTELNVGIDFNWEYPSSKHQHKKLVNRDLKTQSGSEMKKFLSTLTIDGVTRSDQGLYTCAASSGLMTKKNSTFVRVHEKPFVAFGSGMESLVEATVGERVRIPAKYLGYPPPEIKWYKNGIPLESNHTIKAGHVLTIMEVSERDTGNYTVILTNPISKEKQSHVVSLVVYVPPQIGEKSLISPVDSYQYGTTQTLTCTVYAIPPPHHIHWYWQLEEECANEPSQAVSVTNPYPCEEWRSVEDFQGGNKIEVNKNQFALIEGKNKTVSTLVIQAANVSALYKCEAVNKVGRGERVISFHVTRGPEITLQPDMQPTEQESVSLWCTADRSTFENLTWYKLGPQPLPIHVGELPTPVCKNLDTLWKLNATMFSNSTNDILIMELKNASLQDQGDYVCLAQDRKTKKRHCVVRQLTVLERVAPTITGNLENQTTSIGESIEVSCTASGNPPPQIMWFKDNETLVEDSGIVLKDGNRNLTIRRVRKEDEGLYTCQACSVLGCAKVEAFFIIEGAQEKTNLERTHHHHHH

>3J16C

MKLNISYPVNGSQKTFEIDDEHRIRVFFDKRIGQEVDGEAVGDEFKGYVFKISGGNDKQGFPMKQGVLLPTRIKLLLTKNVSCYRPRRDGERKRKSVRGAIVGPDLAVLALVIVKKGEQELEGLTDTTVPKRLGPKRANNIRKFFGLSKEDDVRDFVIRREVTKGEKTYTKAPKIQRLVTPQRLQRKRHQRALKVRNAQAQREAAAEYAQLLAKRLSERKAEKAEIRKRRASSLKA

>3VMGA

MANVDEAILKRVKGWAPYVDAKLGFRNHWYPVMFSKEINEGEPKTLKLLGENLLVNRIDGKLYCLKDRCLHRGVQLSVKVECKTKSTITCWYHAWTYRWEDGVLCDILTNPTSAQIGRQKLKTYPVQEAKGCVFIYLGDGDPPPLARDTPPNFLDDDMEILGKNQIIKSNWRLAVENGFDPSHIYIHKDSILVKDNDLALPLGFAPGGDRKQQTRVVDDDVVGRKGVYDLIGEHGVPVFEGTIGGEVVREGAYGEKIVANDISIWLPGVLKVNPFPNPDMMQFEWYVPIDENTHYYFQTLGKPCANDEERKKYEQEFESKWKPMALEGFNNDDIWAREAMVDFYADDKGWVNEILFESDEAIVAWRKLASEHNQGIQTQAHVSGLEHHHHHH

>3V33A

GGGTPKAPNLEPPLPEEEKEGSDLRPVVIDGSNVAMSHGNKEVFSCRGILLAVNWFLERGHTDITVFVPSWRKEQPRPDVPITDQHILRELEKKKILVFTPSRRVGGKRVVCYDDRFIVKLAYESDGIVVSNDTYRDLQGERQEWKRFIEERLLMYSFVNDKFMPPDDPLGRHGPSLDNFLRKKPLTLEHRKQPCPYGRKCTYGIKCRFFHPERPSCPQRSVA

>3V22V

MKRQKRDRLERAHQRGYQAGIAGRSKEMCPYQTLNQRSQWLGGWREAMADRVVMAHHHHHH

>4ABRL

MVALPTINQLVRKGREKVRKKSKVPALKGAPFRRGVCTVVRTVTPKKPNSALRKVAKVRLTSGYEVTAYIPGEGHNLQEHSVVLIRGGRVKDLPGVRYHIVRGVYDAAGVKDRKKSRSKYGTKKPKEAAKTAAKK

>3VM6A

MNHKVHHHHHHIEGRHMAVVKEVLEIAEKIKNMEIRGAGKIARSAAYALQLQAEKSKATNVDEFWKEMKQAAKILFETRPTAVSLPNALRYVMHRGKIAYSSGADLEQLRFVIINAAKEFIHNSEKALERIGEFGAKRIEDGDVIMTHSHSKAAISVMKTAWEQGKDIKVIVTETRPKWQGKITAKELASYGIPVIYVVDSAARHYMKMTDKVVMGADSITVNGAVINKIGTALIALTAKEHRVWTMIAAETYKFHPETMLGQLVEIEMRDPTEVIPEDELKTWPKNIEVWNPAFDVTPPEYVDVIITERGIIPPYAAIDILREEFGWALKYTEPWED

>3J0TL

MKTFTAKPETVKRDWYVVDATGKTLGRLATELARRLRGKHKAEYTPHVDTGDYIIVLNADKVAVTGNKRTDKVYYHHTGHIGGIKQATFEEMIARRPERVIEIAVKGMLPKGPLGRAMFRKLKVYAGNEHNHAAQQPQVLDI

>3UXQD

MAVKKFKPYTPSRRFMTVADFSEITKTEPEKSLVKPLKKTGGRNNQGRITVRFRGGGHKRLYRIIDFKRWDKVGIPAKVAAIEYDPNRSARIALLHYVDGEKRYIIAPDGLQVGQQVVAGPDAPIQVGNALPLRFIPVGTVVHAVELEPKKGAKLARAAGTSAQIQGREGDYVILRLPSGELRKVHGECYATVGAVGNADHKNIVLGKAGRSRWLGRRPHVRGAAMNPVDHPHGGGEGRAPRGRPPASPWGWQTKGLKTRKRRKPSSRFIIARRKK

>3UX2A

SNARIMEEKALEVYDLIRTIRDPEKPNTLEELEVVSESCVEVQEINEEEYLVIIRFTPTVPHCSLATLIGLCLRVKLQRCLPFKHKLEIYISEGTHSTEEDINKQINDKERVAAAMENPNLREIVEQCVL

>3UW8A

MAETPNSDMSGATGGRSKRPKSNQDWWPSKLNLEILDQNARDVGPVEDDFDYAEEFQKLDLEAVKSDLEELMTSSQDWWPADYGHYGPLFIRMAWHSAGTYRTADGRGGAAGGRQRFAPINSWPDNANLDKARRLLLPIKQKYGQKISWADLMILAGNVAIESMGFKTFGYAGGREDAFEEDKAVNWGPEDEFETQERFDEPGEIQEGLGASVMGLIYVNPEGPDGNPDPEASAKNIRQTFDRMAMNDKETAALIAGGHTFGKVHGADDPEENLGPEPEAAPIEQQGLGWQNKNGNSKGGEMITTGIEGPWTQSPTEWDMGYINNLLDYEWEPEKGPGGAWQWAPKSEELKNSVPDAHDPDEKQTPMMLTTDIALKRDPDYREVMETFQENPMEFGMNFAKAWYKLTHRDMGPPERFLGPEVPDEEMIWQDPLPDADYDLIGDEEIAELKEEILDSDLSVSQLVKTAWASASTYRDSDKRGGANGARLRLEPQKNWEVNEPEQLETVLGTLENIQTEFNDSRSDGTQVSLADLIVLGGNAAVEQAAANAGYDVEIPFEPGRVDAGPEHTDAPSFDALKPKVDGVRNYIQDDITRPAEEVLVDNADLLNLTASELTALIGGMRSIGANYQDTDLGVFTDEPETLTNDFFVNLLDMGTEWEPAADSEHRYKGLDRDTGEVKWEATRIDLIFGSNDRLRAISEVYGSADAEKKLVHDFVDTWSKVMKLDRFDLEHHHHHH

>3UW2A

MAHHHHHHMGTLEAQTQGPGSMISQSIFKAYDIRGVIGKTLDADVARSIGRAFGSEVRAQGGDAVVVARDGRLSGPELVGALADGLRAAGVDVVDVGMVPTPVGYFAASVPLALSGGERRVDSCIVVTGSHNPPDYNGFKMVLRGAAIYGDQIQGLYKRIVDARFETGSGSYEQYDVADQYVERIVGDIKLTRPLKLVVDAGNGVAGPLATRLFKALGCELVELFTDIDGNFPNHHPDPAHPENLQDVIAKLKATDAEIGFAFDGDGDRLGVVTKDGQIIYPDRQLMLFAEEVLSRNPGAQIIYDVKCTRNLARWVREKGGEPLMWKTGHSLVKAKLRETGAPLAGEMSGHVFFKDRWYGFDDGLYTGARLLEILARVADPSALLNGLPNAVSTPELQLKLEEGENVKLIDKLRADAKFDGADEVVTIDGLRVEYPDGFGLARSSNTTPVVVLRFEATSDAALARIQDDFRRALKAAKPGANLPF

>3UV1A

DPIHYDKITEEINKAIDDAIAAIEQSETIDPMKVPDHADKFERHVGILDFKGELAMRNIEARGLKQMKRQGDANVKGEEGIVKAHLLIGVHDDIVSMEYDLAYKLGDLHPTTHVISDIQDFVVALSLEIPDEGNITMTSFEVRQFANVVNHIGGLSILDPIFGVLSDVLTAIFQDTVRKEMTKVLAPAFKRELEKN

>4A9WA

MDSVDVVVIGGGQSGLSAGYFLRRSGLSYVILDAEASPGGAWQHAWHSLHLFSPAGWSSIPGWPMPASQGPYPARAEVLAYLAQYEQKYALPVLRPIRVQRVSHFGERLRVVARDGRQWLARAVISATGTWGEAYTPEYQGLESFAGIQLHSAHYSTPAPFAGMRVAIIGGGNSGAQILAEVSTVAETTWITQHEPAFLADDVDGRVLFERATERWKAQQEGREPDLPPGGFGDIVMVPPVLDARARGVLAAVPPPARFSPTGMQWADGTERAFDAVIWCTGFRPALSHLKGLDLVTPQGQVEVDGSGLRALAVPSVWLLGYGDWNGMASATLIGVTRYAREAVRQVTAYCADHQDR

>3UV2A

SMQCQSTEDAMTVLTPLTEKDYEGLKRVLRSLQAHKMAWPFLEPVDPNDAPDYYGVIKEPMDLATMEERVQRRYYEKLTEFVADMTKIFDNCRYYNPSDSPFYQCAEVLESFFVQKLKGFKASRSH

>3UUMA

DMDLDSYQIALEEVLTWLLSAEDTFQEQDDISDDVEDVKEQFATHETFMMELSAHQSSVGSVLQAGNQLMTQGTLSDEEEFEIQEQMTLLNARWEALRVESMERQSRLHDALMELQKKQLQQL

>3UTNX

MGSSHHHHHHSSGLVPRGSHMASMPLFDLISPKAFVKLVASEKVHRIVPVDATWYLPSWKLDNKVDFLTKPRIPNSIFFDIDAISDKKSPYPHMFPTKKVFDDAMSNLGVQKDDILVVYDRVGNFSSPRCAWTLGVMGHPKVYLLNNFNQYREFKYPLDSSKVAAFSPYPKSHYESSESFQDKEIVDYEEMFQLVKSGELAKKFNAFDARSLGRFEGTEPEPRSDIPSGHIPGTQPLPYGSLLDPETKTYPEAGEAIHATLEKALKDFHCTLDPSKPTICSCGTGVSGVIIKTALELAGVPNVRLYDGSWTEWVLKSGPEWIAENRD

>4A9AA

MHHHHHHMSTTVEKIKAIEDEMARTQKNKATSFHLGQLKAKLAKLRRELLTSASSGSGGGAGIGFDVARTGVASVGFVGFPSVGKSTLLSKLTGTESEAAEYEFTTLVTVPGVIRYKGAKIQMLDLPGIIDGAKDGRGRGKQVIAVARTCNLLFIILDVNKPLHHKQIIEKELEGVGIRLNKTPPDILIKKKEKGGISITNTVPLTHLGNDEIRAVMSEYRINSAEIAFRCDATVDDLIDVLEASSRRYMPAIYVLNKIDSLSIEELELLYRIPNAVPISSGQDWNLDELLQVMWDRLNLVRIYTKPKGQIPDFTDPVVLRSDRCSVKDFCNQIHKSLVDDFRNALVYGSSVKHQPQYVGLSHILEDEDVVTILKK

>4A91A

MTDTQYIGRFAPSPSGELHFGSLIAALGSYLQARARQGRWLVRIEDIDPPREVPGAAETILRQLEHYGLHWDGDVLWQSQRHDAYREALAWLHEQGLSYYCTCTRARIQSIGGIYDGHCRVLHHGPDNAAVRIRQQHPVTQFTDQLRGIIHADEKLAREDFIIHRRDGLFAYNLAVVVDDHFQGVTEIVRGADLIEPTVRQISLYQLFGWKVPDYIHLPLALNPQGAKLSKQNHAPALPKGDPRPVLIAALQFLGQQAEAHWQDFSVEQILQSAVKNWRLTAVPESAIVNSTFSNASC

>3UR1C

SQIGETLENIRSIEKLIQNIMRIARETNILALNATIEAARAGEAGKGFMIVANEVQNLSNETNEVTKQIVEKAREILESSQRSLE

>4A8JC

MHHHHHHMGSVQRQDLVLFSDQSVLPAHFFQDSNSHNLFFITHQSCTQPLWMINALVETHVLGSPSSLNESSSSMLPSSTRSHAVLASFIHEQNYFTNSLNKLKIPSNNYNVLDFLSDFIVNNIHNKPRDKILSDVLAKFSAAIQNNPTDTIVIIEQPELLLSLVSGLTCSELNNKFITPLLRQCKVLIIVSNSDIFNIDEYDASVHSSNLQNFYKSSFIKSMINLNLNPLKTGFAKDVTGSLHVCRGGAPIATSNTSLHVVENEYLYLNEKESTKLFYR

>3UR1B

KEFEVLSFEIDEQALAFDVDNIEMVIEKSDITPVPKSRHFVEGVINLRGRIIPVVNLAKILGISFDEQKMKSIIVARTKDVEVGFLVDRVLGVLRITENQLDLTNVSDKFGKKSKGLVKTDGRLIIYLDIDKIIEEITV

>4A8JB

MASSSHNPVILLKRILSLTESSPFILCLDSIAQTSYKLIQEFVHQSKSKGNEYPIVYISFETVNKPSYCTQFIDATQMDFVHLVKQIISYLPAATATQAKKHMVIIDSLNYISTEYITRFLSEIASPHCTMVATYHKDIKDENRTVIPDWNNNYPDKLTLLQFMATTIVDIDVVLTGTLDTEEVSELLNEFRIPRGLNNDIFQLRLVNKRKSGRSLEYDFIVNSNTHEYELLSTTKQEEESSSNGLETPEMLQGLTTFNLGTSNKQKLAK

>3UPIA

SMSYTWTGALITPCAAEESKLPINPLSNSLLRHHNMVYATTSRSASLRQKKVTFDRLQVLDDHYRDVLKEMKAKASTVKAKLLSIEEACKLTPPHSAKSKFGYGAKDVRNLSSRAVNHIRSVWEDLLEDTETPIDTTIMAKSEVFCVQPEKGGRKPARLIVFPDLGVRVCEKMALYDVVSTLPQAVMGSSYGFQYSPKQRVEFLVNTWKSKKCPMGFSYDTRCFDSTVTESDIRVEESIYQCCDLAPEARQAIRSLTERLYIGGPLTNSKGQNCGYRRCRASGVLTTSCGNTLTCYLKATAACRAAKLQDCTMLVNGDDLVVICESAGTQEDAAALRAFTEAMTRYSAPPGDPPQPEYDLELITSCSSNVSVAHDASGKRVYYLTRDPTTPLARAAWETARHTPINSWLGNIIMYAPTLWARMILMTHFFSILLAQEQLGKALDCQIYGACYSIEPLDLPQIIERLHGLSAFTLHSYSPGEINRVASCLRKLGVPPLRTWRHRARSVRAKLLSQGGRAAICGRYLFNWAVRTKLKLTPIPAASQLDLSGWFVAGYSGGDIYHSLSRARPRENLYFQGLEHHHHHH

>3UOQG

MPRRRVIGQRKILPDPKFGSELLAKFVNILMVDGKKSTAESIVYSALETLAQRSGKSELEAFEVALENVRPTVEVKSRRVGGSTYQVPVEVRPVRRNALAMRWIVEAARKRGDKSMALRLANELSDAAENKGTAVKKREDVHRMAEANKAFAHYRWLSLRSFSHQAGASSKQPALGYLN

>3VKFA

SQKLDDVDPLVTTNFGKIRGIKKELNNEILGPVIQFLGVPYAAPPTGEHRFQPPEPPSPWSDIRNATQFAPVCPQNIIDGRLPEVMLPVWFTNNLDVVSSYVQDQSEDCLYLNIYVPTEDVKRISKECARKPGKKICRKGDIRDSGGPKPVMVYIHGGSYMEGTGNLYDGSVLASYGNVIVITVNYRLGVLGFLSTGDQAAKGNYGLLDLIQALRWTSENIGFFGGDPLRITVFGSGAGGSCVNLLTLSHYSEGLFQRAIAQSGTALSSWAVSFQPAKYARILATKVGCNVSDTVELVECLQKKPYKELVDQDVQPARYHIAFGPVIDGDVIPDDPQILMEQGEFLNYDIMLGVNQGEGLKFVENIVDSDDGVSASDFDFAVSNFVDNLYGYPEGKDVLRETIKFMYTDWADRHNPETRRKTLLALFTDHQWVAPAVATADLHSNFGSPTYFYAFYHHCQTDQVPAWADAAHGDEVPYVLGIPMIGPTELFPCNFSKNDVMLSAVVMTYWTNFAKTGDPNQPVPQDTKFIHTKPNRFEEVAWTRYSQKDQLYLHIGLKPRVKEHYRANKVNLWLELVPHLHNLND

>4A7FB

ELDRAQERLATALQKLEEAEKAADESERGMKVIESRAQKDEEKMEIQEIQLKEAKHIAEDADRKYEEVARKLVIIESDLERAEERAELSEGKCAELEEELKTVTNNLKSLEAQAEKYSQKEDKYEEEIKVLSDKLK

>4A7KA

MYSKVFLKPHCEPEQPAALPLFQPQLVQGGRPDGYWVEAFPFRSDSSKCPNIIGYGLGTYDMKSDIQMLVNPYATTNNQSSSWTPVPLAKLDFPVAMHYADITKNGFNDVIITDQYGSSMDDIWAYGGRVSWLENPGELRDNWTMRTIGHSPGMHRLKAGHFTRTDRVQVVAVPIVVASSDLTTPADVIIFTAPDDPRSEQLWQRDVVGTRHLVHEVAIVPAAETDGEMRFDQIILAGRDGVDCLWYDGARWQRHLVGTGLPEERGDPYWGAGSAAVGRVGDDYAGYICSAEAFHGNTVSVYTKPAGSPTGIVRAEWTRHVLDVFGPLNGKHTGSIHQVVCADIDGDGEDEFLVAMMGADPPDFQRTGVWCYKLVDRTNMKFSKTKVSSVSAGRIATANFHSQGSEVDIATISYSVPGYFESPNPSINVFLSTGILAERLDEEVMLRVVRAGSTRFKTEMEFLDVAGKKLTLVVLPPFARLDVERNVSGVKVMAGTVCWADENGKHERVPATRPFGCESMIVSADYLESGEEGAILVLYKPSSTSGRPPFRSMDELVAHNLFPAYVPDSVRAMKFPWVRCADRPWAHGRFKDLDFFNLIGFHVNFADDSAAVLAHVQLWTAGIGVSAGFHNHVEASFCEIHACIANGTGRGGMRWATVPDANFNPDSPNLEDTELIVVPDMHEHGPLWRTRPDGHPLLRMNDTIDYPWHAWLAGAGNPSPQAFDVWVAFEFPGFETFSTPPPPRVLEPGRYAIRFGDPHQTASLALQKNDATDGTPVLALLDLDGGPSPQAWNISHVPGTDMYEIAHAKTGSLVCARWPPVKNQRVAGTHSPAAMGLTSRWAVTKNTKGQITFRLPEAPDHGPLFLSVSAIRHQQEADAIPVIVQGDSIELSAWSLVPAN

>3UMFA

MGSSHHHHHHSSGLVPRGSHMTDQKLAKAKVIFVLGGPGSGKGTQCEKLVQKFHFNHLSSGDLLRAEVQSGSPKGKELKAMMERGELVPLEVVLALLKEAMIKLVDKNCHFLIDGYPRELDQGIKFEKEVCPCLCVINFDVSEEVMRKRLLKRAETSNRVDDNEETIVKRFRTFNELTKPVIEHYKQQNKVITIDASGTVDAIFDKVNHELQKFGVK

>3ULRB

GPLGSSDLGITAIALYDYQAAGDDEISFDPDDIITNIEMIDDGWWRGVCKGRYGLFPANYVELRQ

>2LLIA

KEAAPKCNNCSQRGHLKKDCPHIICSYCGATDDHYSRHCPKAIQCSKCDEVGHYRSQCPHKWKKVQCTLCKSKKHSKERCPSIWRAYILVDDNEKAKPKVLPFHTIYCYNCGGKGHFGDDCKEK

>4A6SA

AWKGEVLANNEAGQVTSIIYNPGDVITIVAAGWASYGPTQKWGPQGDREHPDQGLICHDAFCGALVMKIGNSGTIPVNTGLFRWVAPNNVQGAITLIYNDVPGTYGNNSGSFSVNIGKDQS

>2LLFA

PRLFECSNKTGRFLATEIVDFTQDDLDENDVYLLDTWDQIFFWIGKGANESEKEAAAETAQEYLRSHPGSRDLDTPIIVVKQGFEPPTFTGWFMAWDPLCWSDRKSY

>3UILA

EDPPACGSIVPRREWRALASECRERLTRPVRYVVVSHTAGSHCDTPASCAQQAQNVQSYHVRNLGWCDVGYNFLIGEDGLVYEGRGWNIKGAHAGPTWNPISIGISFMGNYMNRVPPPRALRAAQNLLACGVALGALRSNYEVKGHRDVQPTLSPGDRLYEIIQTWSHYRA

>3UGJA

GLVPRGSHMMEILRGSPALSAFRINKLLARFQAANLQVHNIYAEYVHFADLNAPLNDSEQAQLTRLLQYGPALSSHTPAGKLLLVTPRPGTISPWSSKATDIAHNCGLQQVDRLERGVAYYIEASTLTAEQWRQVAAELHDRMMETVFSSLTDAEKLFIHHQPAPVSSVDLLGEGRQALIDANLRLGLALAEDEIDYLQEAFTKLGRNPNDIELYMFAQANSEHCRHKIFNADWIIDGKPQPKSLFKMIKNTFETTPDYVLSAYKDNAAVMEGSAVGRYFADHNTGRYDFHQEPAHILMKVETHNHPTAISPWPGAATGSGGEIRDEGATGRGAKPKAGLVGFSVSNLRIPGFEQPWEEDFGKPERIVTALDIMTEGPLGGAAFNNEFGRPALTGYFRTYEEKVNSHNGEELRGYHKPIMLAGGIGNIRADHVQKGEIVVGAKLIVLGGPAMNIGLGGGAASSMASGQSDADLDFASVQRDNPEMERRCQEVIDRCWQLGDANPILFIHDVGAGGLSNAMPELVSDGGRGGKFELRDILSDEPGMSPLEIWCNESQERYVLAVAADQLPLFDELCKRERAPYAVIGDATEEQHLSLHDNHFDNQPIDLPLDVLLGKTPKMTRDVQTLKAKGDALNRADITIADAVKRVLHLPTVAEKTFLVTIGDRTVTGMVARDQMVGPWQVPVADCAVTTASLDSYYGEAMSIGERAPVALLDFAASARLAVGEALTNIAATQIGDIKRIKLSANWMAAAGHPGEDAGLYDAVKAVGEELCPQLGLTIPVGKDSMSMKTRWQEGNEQREMTSPLSLVISAFARVEDVRHTLTPQLSTEDNALLLIDLGKGHNALGATALAQVYRQLGDKPADVRDVAQLKGFYDAMQALVAARKLLAWHDRSDGGLLVTLAEMAFAGHCGVQVDIAALGDDHLAALFNEELGGVIQVRAEDRDAVEALLAQYGLADCVHYLGQALAGDRFVITANDQTVFSESRTTLRVWWAETTWQMQRLRDNPQCADQEHEAKANDTDPGLNVKLSFDINEDIAAPYIATGARPKVAVLREQGVNSHVEMAAAFHRAGFDAIDVHMSDLLGGRIGLGNFHALVACGGFSYGDVLGAGEGWAKSILFNHRVRDEFETFFHRPQTLALGVCNGCQMMSNLRELIPGSELWPRFVRNHSDRFEARFSLVEVTQSPSLLLQGMVGSQMPIAVSHGEGRVEVRDDAHLAALESKGLVALRYVDNFGKVTETYPANPNGSPNGITAVTTENGRVTIMMPHPERVFRTVANSWHPENWGEDSPWMRIFRNARKQLG

>3UGQA

MGSSHHHHHHSSGLVPRGSHMASATKNASSATPATMTSMVSQRQDLFMTDPLSPGSMFFLPNGAKIFNKLIEFMKLQQKFKFGFNEVVTPLIYKKTLWEKSGHWENYADDMFKVETTDEEKEEYGLKPMNCPGHCLIFGKKDRSYNELPLRFSDFSPLHRNEASGALSGLTRLRKFHQDDGHIFCTPSQVKSEIFNSLKLIDIVYNKIFPFVKGGSGAESNYFINFSTRPDHFIGDLKVWNHAEQVLKEILEESGKPWKLNPGDGAFYGPKLDIMVTDHLRKTHQVATIQLDFQLPERFDLKFKDQDNSYKRPIMIHRATFGSIERFMALLIDSNEGRWPFWLNPYQAVIIPVNTKNVQQLDMCTALQKKLRNELEADDMEPVPLNDWHFNVDLDIRNEPVGYRIKSAILKNYSYLIIVGDEEVQLQKYNIRERDNRKSFEKLTMSQIWEKFIELEKNYK

>4A69C

GAMRQLAVIPPMLYDADQQRIKFINMNGLMADPMKVYKDRQVMNMWSEQEKETFREKFMQHPKNFGLIASFLERKTVAECVLYYYLTKKNENYK

>4A6DA

MGSSEDQAYRLLNDYANGFMVSQVLFAACELGVFDLLAEAPGPLDVAAVAAGVRASAHGTELLLDICVSLKLLKVETRGGKAFYRNTELSSDYLTTVSPTSQCSMLKYMGRTSYRCWGHLADAVREGRNQYLETFGVPAEELFTAIYRSEGERLQFMQALQEVWSVNGRSVLTAFDLSVFPLMCDLGGGAGALAKECMSLYPGCKITVFDIPEVVWTAKQHFSFQEEEQIDFQEGDFFKDPLPEADLYILARVLHDWADGKCSHLLERIYHTCKPGGGILVIESLLDEDRRGPLLTQLYSLNMLVQTEGQERTPTHYHMLLSSAGFRDFQFKKTGAIYDAILARKGTHHHHHH

>3UEZE

GSHMEEVSEYCSHMIGSGHLQSLQRLIDSQMETSCQITFEFVDQEQLKDPVCYLKKAFLLVQDIMEDTMRFRDNTPNAIAIVQLQELSLRLKSCFTKDYEEHDKACVRTFYETPLQLLEKVKNVFNETKNLLDKDWNIFSKNCNNSFAECSSQ

>4A5VA

SSEPAKLDLSCVHSDNKGSRAPTIGEPVPDVSLEQCAAQCKAVDGCTHFTYNDDSKMCHVKEGKPDLYDLTGGKTASRSCDRSCFEQHVSYEGAPDVMTAMVTSQSADCQAACAADPSCEIFTYNEHDQKCTFKGRGFSAFKERGVLGVTSGPKQFCDEGG

>3UBBA

ERAGPVTWVMMIACVVVFIAMQILGDQEVMLWLAWPFDPTLKFEFWRYFTHALMHFSLMHILFNLLWWWYLGGAVEKRLGSGKLIVITLISALLSGYVQQKFSGPWFGGLSGVVYALMGYVWLRGERDPQSGIYLQRGLIIFALIWIVAGWFDLFGMSMANGAHIAGLAVGLAMAFVDSLNA

>3UBRA

SDKTEPRNEVYKDKFKNQYNSWHDTAKSEELVDALEQDPNMVILWAGYAFAKDYKAPRGHMYAVTDVRNTLRTGAPKNAEDGPLPMACWSCKSPDVPRLIEEQGEDGYFKGKWAKGGPEVTNTIGCSDCHEKGSPKLRISRPYVDRALDAIGTPFSKASKQDKESMVCAQCHVEYYFEKKEDKKGFVKFPWDMGVTVDQMEVYYDGIEFSDWTHALSKTPMLKAQHPEYETWKMGIHGKNNVSCVDCHMPKVTSPEGKKFTDHKVGNPFDRFEETCATCHSQTKEFLVGVTNERKAKVKEMKLKAEEQLVKAHFEAAKAWELGATEAEMKPILTDIRHAQWRWDLAIASHGVAAHAPEEALRVLGTSVNKAADARVKLAQLLAKKGLTDPVAIPDISTKAKAQAVLGMDMEKMNAEKEAFKKDMLPKWDAEAKKREATY

>4A53A

GAMGMSVADFYGSNVEVLLNNDSKARGVITNFDSSNSILQLRLANDSTKSIVTKDIKDLRILPKNEIMPKNGTKSPSTNSTKLKSAETYSSKNKWSMDCDEEFDFAANLEKFDKKQVFAEFREKD

>3UA0A

MGHHHHHHMRVKTFVILCCALQYVAYTNANINDFDEDYFGSDVTVQSSNTTDEIIRDASGAVIEEQITTKKMQRKNKNHGILGKNEKMIKTFVITTDSDGNESIVEEDVLMKTLSDGTVAQSYVAADAGAYSQS

>3U9GA

GPLGMADPGVCCFITKILCAHGGRMTLEELLGEIRLPEAQLYELLETAGPDRFVLLETGGQAGITRSVVATTRARVCRRKYCQRPCDSLHLCKLNLLGRCHYAQSQRNLCKYSHDVLSEQNFQILKNHELSGLNQEELACLLVQSDPFFLPEICKSYKGEGRKQTCGQPQPCERLHICEHFTRGNCSYLNCLRSHNLMDRKVLTIMREHGLSPDVVQNIQDICNNKHAR

>3U88C

SMDSRLQRIHAEIKNSLKIDNLDVNRCIEALDELASLQVTMQQAQKHTEMITTLKKIRRFKVSQVIMEKSTMLYNKFKNMFLVGEGDSV

>3U6WA

GAMTTSESPDAYTESFGAHTIVKPAGPPRVGQPSWNPQRASSMPVNRYRPFAEEVEPIRLRNRTWPDRVIDRAPLWCAVDLRDGNQALIDPMSPARKRRMFDLLVRMGYKEIEVGFPSASQTDFDFVREIIEQGAIPDDVTIQVLTQCRPELIERTFQACSGAPRAIVHFYNSTSILQRRVVFRANRAEVQAIATDGARKCVEQAAKYPGTQWRFEYSPESYTGTELEYAKQVCDAVGEVIAPTPERPIIFNLPATVEMTTPNVYADSIEWMSRNLANRESVILSLHPHNDRGTAVAAAELGFAAGADRIEGCLFGNGERTGNVCLVTLGLNLFSRGVDPQIDFSNIDEIRRTVEYCNQLPVHERHPYGGDLVYTAFSGSHQDAINKGLDAMKLDADAADCDVDDMLWQVPYLPIDPRDVGRTYEAV

>3U43A

MELKHSISDYTEAEFLEFVKKICRAEGATEEDDNKLVREFERLTEHPDGSDLIYYPRDDREDSPEGIVKEIKEWRAANGKSGFKQGLEHHHHHH

>3VIQB

MEKSQLESRVHLLEQQKEQLESSLQDALAKLKNRDAKQTVQKHIDLLHTYNEIRDIALGMIGKVAEHEKCTSVELFDRFGVNGSE

>3U3EA

MVLSEGEWQLVLHVWAKVEADVAGHGQDILIRLFKSHPETLEKFDRFKHLKTEAEMKASEDLKKHGVTVLTALGAILKKKGHHEAELKPLAQSHATKHKIPIKYLEFISEAIIHVLHSRHPGDFGADAQGAMNKALELFRKDIAAKYKELGYQG

>3U2GA

MGTYEIRGQVASGFGDQSWDASSFAGFYYDIDDNVSTETLTVSDLDGNVIPEGGLVYTTTIADVDFEYYNPDAGWDQYPVMGFFAEEYIPINPDKADKIAKLVLDSDDKYTIRTGEMLDLGEGYAIEAKQVDVDGEKVWLEFTKDGEFVDDEIISVSTADDEANTWDVELDDIEDEDDVVVLKVHVNQVFQGAVDSIAQIEGLWLIDYANAMTIESDDEFGNLDDVSIDGDTLKISNEDTFTLTRDSEEEIGEGMYFMIADTSSSDLRYYPYVEKTIGLEHHHHHH

>3U22A

GACDGILEGIYDSPAASDSNELGFIRTDPSTHSGTIYIDATDYRRWTFIDFHTQKVDSVNVTDSEQKEPEEWDIAVHRYDVKTNAGAVLETGFTGFSALRNADAMPEGAYVEDVWTTAKIAIDMSGMMDGNIVYMESYYNEELSKWLNVDKSNMPPTYTLSNKVYMVKLKDGTYAAVRLTNYMNASGVKGFMTIDYIYPFEL

>3U0CA

GSQAANDAANKLFSLTIADLTANQNINTTNAHSTSNILIPELKAPKSLNASSQLTLLIGNLIQILGEKSLTALTNKITAWKSQQQARQQKNLEFSDKINTLLSETEGLTRDYEKQINKLKNADSKIKDLENKINQIQTRLSELDPESPEKKKLSREEIQLTIKKDAAVKDRTLIEQKTLSIHSKLTDKSMQLEKEIDSFSA

>3U0OA

MSENSIRLTQYSHGAGCGCKISPKVLETILHSEQAKFVDPNLLVGNETRDDAAVYDLGNGTSVISTTDFFMPIVDNPFDFGRIAATNAISDIFAMGGKPIMAIAILGWPINKLSPEIAREVTEGGRYACRQAGIALAGGHSIDAPEPIFGLAVTGIVPTERVKKNSTAQAGCKLFLTKPLGIGVLTTAEKKSLLKPEHQGLATEVMCRMNIAGASFANIEGVKAMTDVTGFGLLGHLSEMCQGAGVQARVDYEAIPKLPGVEEYIKLGAVPGGTERNFASYGHLMGEMPREVRDLLCDPQTSGGLLLAVMPEAENEVKATAAEFGIELTAIGELVPARGGRAMVEIR

>4A2NB

MNENLWKICFIVMFIIWVFVRKVYGTRAMKNKSKKKVRPNFEKSLVFLNFIGMVFLPLTAVFSSYLDSFNINLPDSIRLFALIVTFLNIGLFTKIHKDLGNNWSAILEIKDGHKLVKEGIYKNIRHPMYAHLWLWVITQGIILSNWVVLIFGIVAWAILYFIRVPKEEELLIEEFGDEYIEYMGKTGRLFPKVV

>3VIAA

GSMAFVKSGWLLRQSTILKRWKKNWFDLWSDGHLIYYDDQTRQNIEDKVHMPMDCINIRTGQECRDTQPPDGKSKDCMLQIVCRDGKTISLCAESTDDCLAWKFTLQDSRTN

>4A2AA

MIDLSKTVFYTSIDIGSRYIKGLVLGKRDQEWEALAFSSVKSRGLDEGEIKDAIAFKESVNTLLKELEEQLQKSLRSDFVISFSSVSFEREDTVIERDFGEEKRSITLDILSEMQSEALEKLKENGKTPLHIFSKRYLLDDERIVFNPLDMKASKIAIEYTSIVVPLKVYEMFYNFLQDTVKSPFQLKSSLVSTAEGVLTTPEKDRGVVVVNLGYNFTGLIAYKNGVPIKISYVPVGMKHVIKDVSAVLDTSFEESERLIITHGNAVYNDLKEEEIQYRGLDGNTIKTTTAKKLSVIIHARLREIMSKSKKFFREVEAKIVEEGEIGIPGGVVLTGGGAKIPRINELATEVFKSPVRTGCYANSDRPSIINADEVANDPSFAAAFGNVFAVSENPYEETPVKSENPLKKIFRLFKELME

>4A25A

TTIHDVQTTGLTQDAVTGFDASSRLNAGLQEVLVDLTALHLQGKQAHWNIVGENWRDLHLQLDTLVEAARGFSDDVAERMRAVGGVPDARPQTVAASRIGDVGPDEIDTRACVEAIVALVRHTVDTIRRVHDPIDAEDPASADLLHAITLELEKQAWMIGSENRSPRRR

>3TWLA

MPELPEVEAARRAIEENCLGKKIKRVIIADDNKVIHGISPSDFQTSILGKTIISARRKGKNLWLELDSPPFPSFQFGMAGAIYIKGVAVTKYKRSAVKDSEEWPSKYSKFFVELDDGLELSFTDKRRFAKVRLLANPTSVSPISELGPDALLEPMTVDEFAESLAKKKITIKPLLLDQGYISGIGNWIADEVLYQARIHPLQTASSLSKEQCEALHTSIKEVIEKAVEVDADSSQFPSNWIFHNREKKPGKAFVDGKKIDFITAGGRTTAYVPELQKLYGKDAEKAAKVRPAKRGVKPKEDDGDHHHHHH

>3TVLA

MAQGLIEVERKFLPGPGTEERLQELGGTLEYRVTFRDTYYDTPELSLMQADHWLRRREDSGWELKCPGAAGVLGPHTEYKELTAEPTIVAQLCKVLRADGLGAGDVAAVLGPLGLQEVASFVTKRSAWKLVLLGADEEEPQLRVDLDTADFGYAVGEVEALVHEEAEVPTALEKIHRLSSMLGVPAQETAPAKLIVYLQRFRPQDYQRLLEVNSSRERPQETEDPDHCLG

>3TV0A

GSSSMAEKTQKSVKIAPGAVVCVESEIRGDVTIGPRTVIHPKARIIAEAGPIVIGEGNLIEEQALIINAYPDNITPDTEDPEPKPMIIGTNNVFEVGCYSQAMKMGDNNVIESKAYVGRNVILTSGCIIGACCNLNTFEVIPENTVIYGADCLRRVQTERPQPQTLQLDFLMKILPNYHHLKKTMKGSSTPVKN

>3TURA

RRYTLNATALGLGGAATRQLTFQTSSPAHLTMPYVMPGDGEVVGVGEPVAIRFDENIADRGAAEKAIKITTNPPVEGAFYWLNNREVRWRPEHFWKPGTAVDVAVNTYGVDLGEGMFGEDNVQTHFTIGDEVIATADDNTKILTVRVNGEVVKSMPTSMGKDSTPTANGIYIVGSRYKHIIMDSSTYGVPVNSPNGYRTDVDWATQISYSGVFVHSAPWSVGAQGHTNTSHGCLNVSPSNAQWFYDHVKRGDIVEVVNTVGGTLPGIDGLGDWNIPWDQWRAGNAKA

>3TUNA

GPGSMKVEKVFFVTSPIYYVNAAPHIGHVYSTLITDVIGRYHRVKGERVFALTGTDEHGQKVAEAAKQKQVSPYDFTTAVAGEFKKCFEQMDYSIDYFIRTTNEQHKAVVKELWTKLEQKGDIYLGRYEGWYSISDESFLTPQNITDGVDKDGNPCKVSLESGHVVTWVSEENYMFRLSAFRERLLEWYHANPGCIVPEFRRREVIRAVEKGLPDLSVSRARATLHNWAIPVPGNPDHCVYVWLDALTNYLTGSRLRVDESGKEVSLVDDFNELERFPADVHVIGKDILKFHAIYWPAFLLSAGLPLPKKIVAHGWWTKDRKKISKSLGNVFDPVEKAEEFGYDALKYFLLRESGFSDDGDYSDKNMIARLNGELADTLGNLVMRCTSAKINVNGEWPSPAAYTEEDESLIQLIKDLPGTADHYYLIPDIQKAIIAVFDVLRAINAYVTDMAPWKLVKTDPERLRTVLYITLEGVRVTTLLLSPILPRKSVVIFDMLGVPEVHRKGIENFEFGAVPPGTRLGPAVEGEVLFSKRSTENTKST

>3TU5A

MCDEDETTALVCDNGSGLVKAGFAGDDAPRAVFPSIVGRPRHQGVMVGMGQKDSYVGDEAQSKRGILTLKYPIEHGIITNWDDMEKIWHHTFYNELRVAPEEHPTLLTEAPLNPKANREKMTQIMFETFNVPAMYVAIQAVLSLYASGRTTGIVLDSGDGVTHNVPIYEGYALPHAIMRLDLAGRDLTDYLMKILTERGYSFVTTAEREIVRDIKEKLCYVALDFENEMATAASSSSLEKSYELPDGQVITIGNERFRCPETLFQPSFIGMESAGIHETTYNSIMKCDIDIRKDLYANNVMSGGTTMYPGIADRMQKEITALAPSTMKIKIIAPPERKYSVWIGGSILASLSTFQQMWITKQEYDEAGPSIVHRKCF

>3TU3A

MHHHHHHSSGVDLGTENLYFQSNAMIDTWLAQWGLRLPSSNDATLRLQPAEGPELVMERLEGGWLFVVELGLVPSGLPLGVILQLLQVNSPFSSLAPVKLAADDAGRLVLWAEARDGVDDVDALNRLHDRLREGHSRLVPLLEPTGELVPAQIQTSALVFV

>3TT1A

MEVKREHWATRLGLILAMAGNAVGLGNFLRFPVQAAENGGGAFMIPYIIAFLLVGIPLMWIEWAMGRYGGAQGHGTTPAIFYLLWRNRFAKILGVFGLWIPLVVAIYFVYIESWTLGFAIKFLVGLVPEPPPNATDPDSILRPFKEFLYSYIGVPKGDEPILKPSLFAYIVFLITMFINVSILIRGISKGIERFAKIAMPTLFILAVFLVIRVFLLETPNGTAADGLNFLWTPDFEKLKDPGVWIAAVGQIFFTLSLGFGAIITYASYVRKDQDIVLSGLTAATLNEAAEVILGGSISIPAAVAFFGVANAVAIAKAGAFNLGFITLPAIFSQTAGGTFLGFLWFFLLFFAGLTSSIAIMQPMIAFLEDELKLSRKHAVLWTAAIVFFSAHLVMFLNKSLDEMDFWAGTIGVVFFGLTELIIFFWIFGADKAWEEINRGGIIKVPRIYYYVMRYITPAFLAVLLVVWAREYIPKIMEETHWTVWITRFYIIGLFLFLTFLVFLAERRRNHESAGTLVPR

>4A0XA

MRGETLKLKKDKRREAIRQQIDSNPFITDHELSDLFQVSIQTIRLDRTYLNIPELRKRIKLVAEKNYDQISSIEEQEFIGDLIQVNPNVKAQSILDITSDSVFHKTGIARGHVLFAQANSLCVALIKQPTVLTHESSIQFIEKVKLNDTVRAEARVVNQTAKHYYVEVKSYVKHTLVFKGNFKMFYDKRG

>3TS9A

GHMDTRENPFKEKLLEIMASIQTYCQKSPMSDFGTQHYEQWAIQMEKKAAKDGNRKDRVCAEHLRKYNEALQINDTIRMIDAYSHLETFYTDEKEKKFAVLNDSKKSLKLDETDEFLMNLFFDNKKMLKKLAENPKYE

>3VHXB

GSLLFQPDQNAPPIRLRHRRSRSAGDRWVDHKPASNMQTETVMQPHVPHAITVSVANEKALAKCEKYMLTHQELASDGEIETKLIKGDIYKTRGGGQSVQFTDIETLKQESPNGSRKRRS

>3TRTA

GGSKPDCTAAMRDVRQQYESVAAKNLQEAEEWYKSKFADLSEAANRNNDALRQAKQESTEYRRQVQSLTMEVDALKG

>4A0EA

GGGSWVCRFYQGKHRGVEVELPHGRCVFGSDPLQSDIVLSDSEIAPVHLVLMVDEEGIRLTDSAEPLLQEGLPVPLGTLLRAGSCLEVGFLLWTFVAVGQPLPETLQVPTQRKEPTDRLPRSR

>4A03A

TMAHHHHHHVTNSTDGRADGRLRVVVLGSTGSIGTQALQVIADNPDRFEVVGLAAGGAHLDTLLRQRAQTGVTNIAVADEHAAQRVGDIPYHGSDAATRLVEQTEADVVLNALVGALGLRPTLAALKTGARLALANKESLVAGGSLVLRAARPGQIVPVDSEHSALAQCLRGGTPDEVAKLVLTASGGPFRGWSAADLEHVTPEQAGAHPTWSMGPMNTLNSASLVNKGLEVIETHLLFGIPYDRIDVVVHPQSIIHSMVTFIDGSTIAQASPPDMKLPISLALGWPRRVSGAAAACDFHTASSWEFEPLDTDVFPAVELARQAGVAGGCMTAVYNAANEEAAAAFLAGRIGFPAIVGIIADVLHAADQWAVEPATVDDVLDAQRWARERAQRAVSGM

>3TO8A

MGHHHHHHDSPVLMVYGLDQSKMNCDRVFNVFCLYGNVEKVKFMKSKPGAAMVEMADGYAVDRAITHLNNNFMFGQKLNVCVSKQPAIMPGQSYGLEDGSCSYKDFSESRNNRFSTPEQAAKNRIQHPSNVLHFFNAPLEVTEENFFEICDELGVKRPSSVKVFSGKSERSSSGLLEWESKSDALETLGFLNHYQMKNPNGPYPYTLKLCFSTAQHAS

>3TNXA

MHHHHHHSSGLVPRGSGMKETAAAKFERQHMDSPDLGTDDDDKMDFSIVGYSQNDLTSTERLIQLFESWMLKHNKIYKNIDEKIYRFEIFKDNLKYIDETNKKNNSYWLGLNVFADMSNDEFKEKYTGSIAGNYTTTELSYEEVLNDGDVNIPEYVDWRQKGAVTPVKNQGSCGSAWAFSAVSTIESIIKIRTGNLNEYSEQELLDCDRRSYGCNGGYPWSALQLVAQYGIHYRNTYPYEGVQRYCRSREKGPYAAKTDGVRQVQPYNEGALLYSIANQPVSVVLEAAGKDFQLYRGGIFVGPCGNKVDHAVAAVGYGPNYILIRNSWGTGWGENGYIRIKRGTGNSYGVCGLYTSSFYPVKN

>3ZZOA

ECCTSRELVEFKMDRGDCEAVRAIENYPNGCEVTICADGVAQLGAYCGQGPCNIFGCNCDGGCLSGDWSQEFVRRNQQYGIQIIKVTRLPFWRPL

>3TMUA

MRSLLILVLCFLPLAALGKVFGRCELAAAMKRHGLDNYRGYSLGNWVCAAKFESNFNTQATNRNTDGSTDYGILQINSRWWCNDGRTPGSRNLCNIPCSALLSSDITASVNCAKKIVSDGNGMNAWVAWRNRCKGTDVQAWIRGCRL

>3TKLB

GPLGSTSSTSQADKEIQKMLDEYEQAIKRAQENIKKGEELEKKLDKLERQGKDLEDKYKTYEENLEGFEKLLTDSEELSLSEINEKMKAFSKDSEKLTQLMEKHKGDEKTVQSLQREHHDIKAKLANLQVLHDAHTGKKSYVNEKGNPVSSLKDAHLAINKDQEVVEHKGQFYLLQKGQWDAIKNDPAALEKAQKDYSQSKHDLATIKMEALIHKLSLEMEKQLETINDLIMSTDPKENEEATKLLHKHNGLNLKLANLQDMLAVHR

>3TIKA

KGKLPPVYPVTVPILGHIIQFGKSPLGFMQECKRQLKSGIFTINIVGKRVTIVGDPHEHSRFFLPRNEVLSPREVYSFMVPVFGEGVAYAAPYPRMREQLNFLAEELTIAKFQNFVPAIQHEVRKFMAANWDKDEGEINLLEDCSTMIINTACQCLFGEDLRKRLDARRFAQLLAKMESSLIPAAVFLPILLKLPLPQSARCHEARTELQKILSEIIIARKEEEVNKDSSTSDLLSGLLSAVYRDGTPMSLHEVCGMIVAAMFAGQHTSSITTTWSMLHLMHPANVKHLEALRKEIEEFPAQLNYNNVMDEMPFAERCARESIRRDPPLLMLMRKVMADVKVGSYVVPKGDIIACSPLLSHHDEEAFPEPRRWDPERDEKVEGAFIGFGAGVHKCIGQKFGLLQVKTILATAFRSYDFQLLRDEVPDPDYHTMVVGPTASQCRVKYIRRKAAAA

>3TH0A

YDPDQYSIEADKKFKYSVKLSDYPTLQDAASAAVDGLLIDRDYNFYGGETVDFGGKVLTIECKAKFIGDGNLIFTKLGKGSRIAGVFMESTTTPWVIKPWTDDNQWLTDAAAVVATLKQSKTDGYQPTVSDYVKFPGIETLLPPNAKGQNITSTLEIRECIGVEVHRASGLMAGFLFRGCHFCKMVDANNPSGGKDGIITFENLSGDWGKGNYVIGGRTSYGSVSSAQFLRNNGGFERDGGVIGFTSYRAGESGVKTWQGTVGSTTSRNYNLQFRDSVVIYPVWDGFDLGADTDMNPELDRPGDYPITQYPLHQLPLNHLIDNLLVRGALGVGFGMDGKGMYVSNITVEDCAGSGAYLLTHESVFTNIAIIDTNTKDFQANQIYISGACRVNGLRLIGIRSTDGQSLTIDAPNSTVSGITGMVDPSRINVANLAEEGLGNIRANSFGYDSAAIKLRIHKLSKTLDSGALYSHINGGAGSGSAYTQLTAISGSTPDAVSLKVNHKDCRGAEIPFVPDIASDDFIKDSSCFLPYWENNSTSLKALVKKPNGELVRLTLATL

>3TGUB

SLKVAPKVAVSAAAERVKLCPGAEDLEITKLPNGLIIASLENFSPASRIGVFIKAGSRYETTANLGTAHLLRLASPLTTKGASSFRITRGIEAVGGSLSVYSTREKMTYCVECLRDHVDTVMEYLLNVTTAPEFRPWEVTDLQPQLKVDKAVAFQSPQVGVLENLHAAAYKTALANPLYCPDYRIGKITSEQLHHFVQNNFTSARMALVGIGVKHSDLKQVAEQFLNIRSGAGTSSAKATYWGGEIREQNGHSLVHAAVVTEGAAVGSAEANAFSVLQHVLGAGPLIKRGSSVTSKLYQGVAKATTQPFDASAFNVNYSDSGLFGFYTISQAAHAGEVIRAAMNQLKAAAQGGVTEEDVTKAKNQLKATYLMSVETAQGLLNEIGSEALLSGTHTAPSVVAQKIDSVTSADVVNAAKKFVSGKKSMAASGDLGSTPFLDEL

>3ZXUB

MDFTSSSGVLDSERNTGSNDSDEPSSHSDVIETEELKLIKLQEHKNNLLRQRSELLDQLSQTRVVEPRSVQLDDKLLLKLLRRNDNAVSDSSQSSNNPLPRVLPSLNIEQRKKYLDITLNDVTVTCEKDMILLRKGSFTASFRIAVENESIRSMAIDLNAFEVELQPIIQYAEDTQNVNVAMMAVVQFLRIKELHEQMISKIVEASKFIRASNNTITLNDLEVSFHCYWNLPSPYPETLILTNKVQKILDFLIYQYGIQLGVIKYGSTII

>2LHRA

SDDYVDEETYNLQKLLAPYHKAKTLERQVYELEKLQEKLPEKYKAEYKKKLDQTRVELADQVKSAVTEFENVTPTNDQ

>3TEEA

QDINAQLTTWFSQRLAGFSDEVVVTLRSSPNLLPSCEQPAFSMTGSAKLWGNVNVVARCANEKRYLQVNVQATGNYVAVAAPIARGGKLTPANVTLKRGRLDQLPPRTVLDIRQIQDAVSLRDLAPGQPVQLTMIRQAWRVKAGQRVQVIANGEGFSVNAEGQAMNNAAVAQNARVRMTSGQIVSGTVDSDGNILINLDPNSSSVDKLAAALEHHHHHH

>3TDOA

MGRAHKETLDKLTNAAINKINLLNTSKVKYLVSSAFAGLYVGIGILLIFTIGGLLTDAGSPMTKIVMGLSFAIALSLVIMTGTELFTGNNMVMSAGMLNKGVSIKDTSKIWAYSWVGNLIGALVLGIIFVGTGLVDKGPVAEFFANTAASKASMPFTALFFRGILCNILVCVSVLCSFRTNSDTAKIIMIFLCLFAFITSGFEHSVANMTIYSVSLFSPTISTVTIGGAIYNLVAVTLGNIVGGALFMGLGTYILGKEKLNAAAENLY

>3TCJA

MSQFTLYKNKDKSSAKTYPYFVDVQSDLLDNLNTRLVIPLTPIELLDKKAPSHLCPTIHIDEGDFIMLTQQMTSVPVKILSEPVNELSTFRNEIIAAIDFLITGI

>3ZXBA

FTCPECRPELCGDPGYCEYGTTKDACDCCPVCFQGPGGYCGGPEDVFGICADGFACVPLVGERDSQDPEIVGTCVKIP

>3VGBA

TFAYKIDGNEVIFTLWAPYQKSVKLKVLEKGLYEMERDEKGYFTITLNNVKVRDRYKYVLDDASEIPDPASRYQPEGVHGPSQIIQESKEFNNETFLKKEDLIIYEIHVGTFTPEGTFEGVIRKLDYLKDLGITAIEIMPIAQFPGKRDWGYDGVYLYAVQNSYGGPEGFRKLVDEAHKKGLGVILDVVYNHVGPEGNYMVKLGPYFSQKYKTPWGLTFNFDDAESDEVRKFILENVEYWIKEYNVDGFRLDAVHAIIDTSPKHILEEIADVVHKYNRIVIAESDLNDPRVVNPKEKCGYNIDAQWVDDFHHSIHAYLTGERQGYYTDFGNLDDIVKSYKDVFVYDGKYSNFRRKTHGEPVGELDGCNFVVYIQNHDQVGNRGKGERIIKLVDRESYKIAAALYLLSPYIPMIFMGEEYGEENPFYFFSDFSDSKLIQGVREGRKKENGQDTDPQDESTFNASKLSWKIDEEIFSFYKILIKMRKELSIACDRRVNVVNGENWLIIKGREYFSLYVFSKSSIEVKYSGTLLLSSNNSFPQHIEEGKYEFDKGFALYKL

>3ZX8A

MENDPRVRKFASDGAQWAIKWQKKGWSTLTSRQKQTARAAMGIKLSPVAQPVQKVTRLSAPVALAYREVSTQPRVSTARDGITRSGSELITTLKKNTDTEPKYTTAVLNPSEPGTFNQLIKEAAQYEKYRFTSLRFRYSPMSPSTTGGKVALAFDRDAAKPPPNDLASLYNIEGCVSSVPWTGFILTVPTDSTDRFVADGISDPKLVDFGKLIMATYGQGAAQLGEVRVEYTVQLKNRTGSTSAQIGDFAGVKDGPRLVSWSKTKGTAGWEHDCHFLGTGNFSLTLFYEKAPVSGLENADASDFSVLGEAAAGSVQWAGVKVAERGQGVKMVTTEEQPKGKWQALRI

>3TBLA

MFEARLVQGSILKKVLEALKDLINEACWDISSSGVNLQSMDSSHVSLVQLTLRSEGFDTYRCDRNLAMGVNLTSMSKILKCAGNEDIITLRAEDNADTLALVFEAPNQEKVSDYEMKLMDLDVEQLGIPEQEYSCVVKMPSGEFARICRDLSHIGDAVVISCAKDGVKFSASGELGNGNIKLSQTSNVDKEEEAVTIEMNEPVQLTFALRYLNFFTKATPLSSTVTLSMSADVPLVVEYKIADMGHLKYYLAPKIEDEEGS

>3TAYA

GSLLDGPYQPTTFNPPTSYWILLAPTVEGVVIQGTNNIDRWLATILIEPNVQTTNRIYNLFGQQVTLSVENTSQTQWKFIDVSKTTPTGNYTQHGSLFSTPKLYAVMKFSGRIYTYNGTTPNATTGYYSTTNYDTVNMTSFCDFYIIPRNQEEKCTEYINHGL

>3ZWSA

MATGDERFYAEHLMPTLQGLLDPESAHRLAVRFTSLGLLPRARFQDSDMLEVRVLGHKFRNPVGIAAGFDKHGEAVDGLYKMGFGFVEIGSVTPKPQEGNPRPRVFRLPEDQAVINRYGFNSHGLSVVEHRLRARQQKQAKLTEDGLPLGVNLGKNKTSVDAAEDYAEGVRVLGPLADYLVVNVSSPNTAGLRSLQGKAELRRLLTKVLQERDGLRRVHRPAVLVKIAPDLTSQDKEDIASVVKELGIDGLIVTNTTVSRPAGLQGALRSETGGLSGKPLRDLSTQTIREMYALTQGRVPIIGVGGVSSGQDALEKIRAGASLVQLYTALTFWGPPVVGKVKRELEALLKEQGFGGVTDAIGADHRR

>3T60A

MHLKIVCLSDEVREMYKNHKTHHEGDSGLDLFIVKDEVLKPKSTTFVKLGIKAIALQYKSNYYYKCEKSENKKKDDDKSNIVNTSFLLFPRSSISKTPLRLANSIGLIDAGYRGEIIAALDNTSDQEYHIKKNDKLVQLVSFTGEPLSFELVEELDETSRGEGGFGSTSNNKYLEHHHHHH

>3T5VA

GSPLPSDVRPPHILVKTLDYIVDNLLTTLPESEGFLWDRMRSIRQDFTYQNYSGPEAVDCNERIVRIHLLILHIMVKSNVEFSLQQELEQLHKSLITLSEIYDDVRSSGGTCPNEAEFRAYALLSKIRDPQYDENIQRLPKHIFQDKLVQMALCFRRVISNSAYTERGFVKTENCLNFYARFFQLMQSPSLPLLMGFFLQMHLTDIRFYALRALSHTLNKKHKPIPFIYLENMLLFNNRQEIIEFCNYYSIEIINGDAADLKTLQHYSHKLSETQPLKKTYLTCLERRLQKTTYKGLINGGEDNLASSVYVKDPKK

>3T63M

PAQDNSRFVIRDRNWHPKALTPDYKTSIARSPRQALVSIPQSISETTGPNFSHLGFGAHDHDLLLNFNNGGLPIGERIIVAGRVVDQYGKPVPNTLVEMWQANAGGRYRHKNDRYLAPLDPNFGGVGRCLTDSDGYYSFRTIKPGPAPWRNGPNDWRPAHIYFGISGPSIATKLITQLYFEGDPLIPMCPIVKSIANPEAVQQLIAKLDMNNANPMDCLAYRFDIVLRGQRKTHFENC

>3T6BA

MADTQYILPNDIGVSSLDCREAFRLLSPTERLYAYHLSRAAWYGGLAVLLQTSPEAPYIYALLSRLFRAQDPDQLRQHALAEGLTEEEYQAFLVYAAGVYSNMGNYKSFGDTKFVPNLPKEKLERVILGSEAAQQHPEEVRGLWQTCGELMFSLEPRLRHLGLGKEGITTYFSGNCTMEDAKLAQDFLDSQNLSAYNTRLFKEVDGEGKPYYEVRLASVLGSEPSLDSEVTSKLKSYEFRGSPFQVTRGDYAPILQKVVEQLEKAKAYAANSHQGQMLAQYIESFTQGSIEAHKRGSRFWIQDKGPIVESYIGFIESYRDPFGSRGEFEGFVAVVNKAMSAKFERLVASAEQLLKELPWPPTFEKDKFLTPDFTSLDVLTFAGSGIPAGINIPNYDDLRQTEGFKNVSLGNVLAVAYATQREKLTFLEEDDKDLYILWKGPSFDVQVGLHALLGHGSGKLFVQDEKGAFNFDQETVINPETGEQIQSWYRSGETWDSKFSTIASSYEECRAESVGLYLCLHPQVLEIFGFEGADAEDVIYVNWLNMVRAGLLALEFYTPEAFNWRQAHMQARFVILRVLLEAGEGLVTITPTTGSDGRPDARVRLDRSKIRSVGKPALERFLRRLQVLKSTGDVAGGRALYEGYATVTDAPPECFLTLRDTVLLRKESRKLIVQPNTRLEGSDVQLLEYEASAAGLIRSFSERFPEDGPELEEILTQLATADARFW

>3T5AA

MGSSHHHHHHSSGLVPRGSHMSVRSLPAALRACARLQPHDPAFTFMDYEQDWDGVAITLTWSQLYRRTLNVAQELSRCGSTGDRVVISAPQGLEYVVAFLGALQAGRIAVPLSVPQGGVTDERSDSVLSDSSPVAILTTSSAVDDVVQHVARRPGESPPSIIEVDLLDLDAPNGYTFKEDEYPSTAYLQYTSGSTRTPAGVVMSHQNVRVNFEQLMSGYFADTDGIPPPNSALVSWLPFYHDMGLVIGICAPILGGYPAVLTSPVSFLQRPARWMHLMASDFHAFSAAPNFAFELAARRTTDDDMAGRDLGNILTILSGSERVQAATIKRFADRFARFNLQERVIRPSYWLAEATVYVATSKPGQPPETVDFDTESLSAGHAKPCAGGGATSLISYMLPRSPIVRIVDSDTCIECPDGTVGEIWVHGDNVANGYWQKPDESERTFGGKIVTPSPGTPEGPWLRTGDSGFVTDGKMFIIGR

>2LGDA

GSMALPIIVKWGGQEYSVTTLSEDDTVLDLKQFLKTLTGVLPERQKLLGLKVKGKPAENDVKLGALKLKPNTKIMMMGTREES

>3T1HR

MSTKNAKPKKEAQRRPSRKAKVKATLGEFDLRDYRNVEVLKRFLSETGKILPRRRTGLSGKEQRILAKTIKRARILGLLPFTEKLVRK

>3T1HQ

MPKKVLTGVVVSDKMQKTVTVLVERQFPHPLYGKVIKRSKKYLAHDPEEKYKLGDVVEIIESRPISKRKRFRVLRLVESGRMDLVEKYLIRRQNYQSLSKRGGKA

>3ZUIA

DSESDCTGSEPVDAFQAFSEGKEAYVLVRSTDPKARDCLKGEPAGEKQDNTLPVMMTFKNGTDWASTDWTFTLDGAKVTATLGNLTQNREVVYDSQSHHCHVDKVEKEVPDYEMWMLDAGGLEVEVECCRQKLEELASGRNQMYPHLKDC

>3SX6A

MRGSAHVVILGAGTGGMPAAYEMKEALGSGHEVTLISANDYFQFVPSNPWVGVGWKERDDIAFPIRHYVERKGIHFIAQSAEQIDAEAQNITLADGNTVHYDYLMIATGPKLAFENVPGSDPHEGPVQSICTVDHAERAFAEYQALLREPGPIVIGAMAGASCFGPAYEYAMIVASDLKKRGMRDKIPSFTFITSEPYIGHLGIQGVGDSKGILTKGLKEEGIEAYTNCKVTKVEDNKMYVTQVDEKGETIKEMVLPVKFGMMIPAFKGVPAVAGVEGLCNPGGFVLVDEHQRSKKYANIFAAGIAIAIPPVETTPVPTGAPKTGYMIESMVSAAVHNIKADLEGRKGEQTMGTWNAVAFADMGDRGAAFIALPQLKPRKVDVFAYGRWVHLAKVAFEKYFIRKMKMGVSEPFYEKVLFKMMGITRLKEEDTHRKAS

>3SWMA

HHHHHHMGIQETDPLTQLSLPPGFRFYPTDEELMVQYLCRKAAGYDFSLQLIAEIDLYKFDPWVLPNKALFGEKEWYFFSPRDRKYPNGSRPNRVAGSGYWKATGTDKIISTEGQRVGIKKALVFYIGKAPKGTKTNWIMHEYRLIEPSRRNGSTKLDDWVLCRIYKKQSSAQK

>3STTA

GSMEKSMSPFVKKHFVLVHTAFHGAWCWYKIVALMRSSGHNVTALDLGASGINPKQALQIPNFSDYLSPLMEFMASLPANEKIILVGHALGGLAISKAMETFPEKISVAVFLSGLMPGPNIDATTVCTKAGSAVLGQLDNCVTYENGPTNPPTTLIAGPKFLATNVYHLSPIEDLALATALVRPLYLYLAEDISKEVVLSSKRYGSVKRVFIVATENDALKKEFLKLMIEKNPPDEVKEIEGSDHVTMMSKPQQLFTTLLSIANKYK

>3SS3A

GSHMVAAGDNKIKQGLLPSLEDLLFYTIAEGQEKIPVHKFITALKSTGLRTSDPRLKECMDMLRLTLQTTSDGVMLDKDLFKKCVQSNIVLLTQAFRRKFVIPDFMSFTSHIDELYESAKKQSGGKVADYIPQLAKFSPDLWGVSVCTVDGQRHSIGDTKVPFCLQSCVKPLKYAIAVNDLGTEYVHRYVGKEPSGLRFNKLFLNEDDKPHNPMVNAGAIVVTSLIKQGVNNAEKFDYVMQFLNKMAGNEYVGFSNATFQSERESGDRNFAIGYYLKEKKCFPEGTDMVGILDFYFQLCSIEVTCESASVMAATLANGGFCPITGERVLSPEAVRNTLSLMHSCGMYDFSGQFAFHVGLPAKSGVAGGILLVVPNVMGMMCWSPPLDKMGNSVKGIHFCHDLVSLCNFHNYDNLRHFAKKLDPRREGGDQRHSFGPLDYESLQQELALKDTVWKKVSPESSDDTSTTVVYRMESLGERS

>3SQRA

MKYFTVFTALTALFAQASASAIPAVRSTLTPRQNTTASCANSATSRSCWGEYSIDTNWYDVTPTGVTREYWLSVENSTITPDGYTRSAMTFNGTVPGPAIIADWGDNLIIHVTNNLEHNGTSIHWHGIRQLGSLEYDGVPGVTQCPIAPGDTLTYKFQVTQYGTTWYHSHFSLQYGDGLFGPLIINGPATADYDEDVGVIFLQDWAHESVFEIWDTARLGAPPALENTLMNGTNTFDCSASTDPNCVGGGKKFELTFVEGTKYRLRLINVGIDSHFEFAIDNHTLTVIANDLVPIVPYTTDTLLIGIGQRYDVIVEANAAADNYWIRGNWGTTCSTNNEAANATGILRYDSSSIANPTSVGTTPRGTCEDEPVASLVPHLALDVGGYSLVDEQVSSAFTNYFTWTINSSSLLLDWSSPTTLKIFNNETIFPTEYNVVALEQTNANEEWVVYVIEDLTGFGIWHPIHLHGHDFFIVAQETDVFNSDESPAKFNLVNPPRRDVAALPGNGYLAIAFKLDNPGSWLLHCHIAWHASEGLAMQFVESQSSIAVKMTDTAIFEDTCANWNAYTPTQLFAEDDSGI

>3SO6A

MEGMVFSLKYLGMTLVERPKGEELSAAAVKRIVATAKASGKKLQKVTLKVSPRGIILTDSLTSQLIENVSIYRISYCTADKMHDKVFAYIAQSQQNESLECHAFLCTKRKVAQAVTLTVAQAFKVAFEFWQVSLVPR

>3SMHA

SRNNPFYFPSRRFSTRYGNQNGRIRVLQRFDQRSRQFQNLQNHRIVQIEAKPNTLVLPKHADADNILVIQQGQATVTVANGNNRKSFNLDEGHALRIPSGFISYILNRHDNQNLRVAKISMPVNTPGQFEDFFPASSRDQSSYLQGFSRNTLEAAFNAEFNEIRRVLLEENAGGEQEERGQRRWSTRSSENNEGVIVKVSKEHVEELTKHAKSVSKKGSEEEGDITNPINLREGEPDLSNNFGKLFEVKPDKKNPQLQDLDMMLTCVEIKEGALVLPHFNSKAMVIVVVNKGTGNLELVAVRKEQQQRGRREEEEDEDEEEEGSNREVRRYTARLKEGDVFIMPAAHPVAINASSELHLLGFGINAENNHRIFLAGDKDNVIDQIEKQAKDLAFPGSGEQVEKLIKNQKESHFVSARP

>3B18A

MSHTDLTPCTRVLASSGTVPIAEELLARVLEPYSCKGCRYLIDAQYSATEDSVLAYGNFTIGESAYIRSTGHFNAVELILCFNQLAYSAFAPAVLNEEIRVLRGWSIDDYCQHQLSSMLIRKASSRFRKPLNPQKFSARLLCRDLQVIERTWRYLKVPCVIEFWDENGGAASGEIELAALNIP

>3ZSCA

SLNDKPVGFASVPTADLPEGTVGGLGGEIVFVRTAEELEKYTTAEGKYVIVVDGTIVFEPKREIKVLSDKTIVGINDAKIVGGGLVIKDAQNVIIRNIHFEGFYMEDDPRGKKYDFDYINVENSHHIWIDHITFVNGNDGAVDIKKYSNYITVSWNKFVDHDKVSLVGSSDKEDPEQAGQAYKVTYHHNYFKNLIQRMPRIRFGMAHVFNNFYSMGLRTGVSGNVFPIYGVASAMGAKVHVEGNYFMGYGAVMAEAGIAFLPTRIMGPVEGYLTLGEGDAKNEFYYCKEPEVRPVEEGKPALDPREYYDYTLDPVQDVPKIVVDGAGAGKLVFEELNTAQ

>2LENA

MQLKPMEINPEMLNKVLYRLGVAGQWRFVDVLGLEEESLGSVPAPACALLLLFPLTAQHENFRKKQIEELKGQEVSPKVYFMKQTIGNSCGTIGLIHAVANNQDKLGFEDGSVLKQFLSETEKMSPEDRAKCFEKNEAIQAAHDAVAQEGQCRVDDKVNFHFILFNNVDGHLYELDGRMPFPVNHGASSEDTLLKDAAKVCREFTEREQGEVRFSAVALCKAALEHHHHHH

>2LELA

VDMSNVVKTYDLQDGSKVHVFKDGKMGMENKFGKSMNMPEGKVMETRDGTKIIMKGNEIFRLDEALRKGHSEGG

>3SGZA

PLVCLADFKAHAQKQLSKTSWDFIEGEADDGITYSENIAAFKRIRLRPRYLRDMSKVDTRTTIQGQEISAPICISPTAFHSIAWPDGEKSTARAAQEANICYVISSYASYSLEDIVAAAPEGFRWFQLYMKSDWDFNKQMVQRAEALGFKALVITIDTPVLGNRRRDKRNQLNLEANILKAALRALKEEKPTQSVPVLFPKASFCWNDLSLLQSITRLPIILKGILTKEDAELAMKHNVQGIVVSNHGGRQLDEVSASIDALREVVAAVKGKIEVYMDGGVRTGTDVLKALALGARCIFLGRPILWGLACKGEDGVKEVLDILTAELHRCMTLSGCQSVAEISPDLIQFSRL

>3SGFY

MAAKIRRDDEVIVLTGKDKGKRGKVKNVLSSGKVIVEGINLVKKHQKPVPALNQPGGIVEKEAAIQVSNVAIFNAATGKADRVGFRFEDGKKVRFFKSNSETIK

>3ZQDA

GRKLLTYQVKQGDTLNSIAADFRISTAALLQANPSLQAGLTAGQSIVIPGLPDPYTIPYHIAVSIGAKTLTLSLNNRVMKTYPIAVGKILTQTPTGEFYIINRQRNPGGPFGAYWLSLSKQHYGIHGTNNPASIGKAVSKGCIRMHNKDVIELASIVPNGTRVTINRGSHHHHHH

>3SCIE

RVVPSGDVVRFPNITNLCPFGEVFNATKFPSVYAWERKKISNCVADYSVLYNSTFFSTFKCYGVSATKLNDLCFSNVYADSFVVKGDDVRQIAPGQTGVIADYNYKLPDDFMGCVLAWNTRNIDATSTGNYNYKYRFLRHGKLRPFERDISNVPFSPDGKPCTPPAFNCYWPLNDYGFYTTTGIGYQPYRVVVLSFELLNAPATVCGPKLSTDLIKNQCVNFHHHHHH

>2YMAA

GSIGSNSIDLITKYEPIFLGSGIYFLRPFNTDERDKLMVTDNAMSNWDEITETYYQKFGNAINKMLSLRLVSLPNGHILQPGDSCVWLAEVVDMKDRFQTTLSLNILNSQRAEIFFNKTFTFNEDNGNFLSYKIGDHGESTELGQITHSNKADINTAEIRS

>3SBSA

MNTVPFTSAPIEVTIGIDQYSFNVKENQPFHGIKDIPIGHVHVIHFQHADNSSMRYGYWFDCRMGNFYIQYDPKDGLYKMMEERDGAKFENIVHNFKERQMMVSYPKIDEDDTWYNLTEFVQMDKIRKIVRKDENQFSYVDSSMTTVQENELLKSSLQKAGSKMEAKNEDDPAHSLNYTVINFKSREAIRPGHEMEDFLDKSYYLNTVMLQGIFKNSSNYFGELQFAFLNAMFFGNYGSSLQWHAMIELICSSATVPKHMLDKLDEILYYQIKTLPEQYSDILLNERVWNICLYSSFQKNSLHNTEKIMENKYPELLGKDNEDDALIYGISDEERDDEDDEHNPTIVGGLYYQRPLEHHHHHH

>2YJGA

MANIEIPYGKSKLAFDLPDERIQGILRSKAGSYKVNMSEEDIVKRALENPIGTKRLQDLAEGKKNIVIITSDHTRPVPSRITLPLLLDEIRKKNKSANVKILIATGFHRGTTLQEMKAKFGEDLVENEQFVVHDSRNSENMELIGTLPSGGKLEINKLAVEADLLVAEGFIEPHFFAGFSGGRKSILPGIASVQCILANHCSEFIKNPYARTGVLENNPIHRDMIYAAKKANLAFILNVVIDSSHKIVNAFAGHSEKAHLKGCEFVSEIATVNAKPADIVITSNGGYPLDQNIYQSVKGMTAGEAACKDGGVIIIAAECADGHGGEGFYRWFKESKDPQDVMNKILSRGRDETLPDQWEAQILARILINHKVIMVTDSKNYEYVKDMFMTPAKDLGEALKIAESIVNNDSKINVIPDGVSVIVREKASWSHPQFEK

>3S4EA

ASQVGVIKPWLLLGSQDAAHDLDTLKKNKVTHILNVAYGVENAFLSDFTYKSISILDLPETNILSYFPECFEFIEEAKRKDGVVLVHSNAGVSRAAAIVIGFLMNSEQTSFTSAFSLVKNARPSICPNSGFMEQLRTYQEGKES

>3S44A

MKTITLYLDPASLPALNQLMDFTQNNEDKTHPRIFGLSRFKIPDNIITQYQNIHFVELKDNRPTEALFTILDQYPGNIELNIHLNIAHSVQLIRPILAYRFKHLDRVSIQQLNLYDDGSDEYVDLEKEENKDISAEIKQAEKQLSHYLLTGKIKFDNPTIARYVWQSAFPVKYHFLSTDYFEKAEFLQPLKEYLAENYQKMDWTAYQQLTPEQQAFYLTLVGFNDEVKQSLEVQQAKFIFTGTTTWEGNTDVREYYAQQQLNLLNHFTQAEGDLFIGDHYKIYFKGHPRGGEINDYILNNAKNITNIPANISFEVLMMTGLLPDKVGGVASSLYFSLPKEKISHIIFTSNKQVKSKEDALNNPYVKVMRRLGIIDESQVIFWDSLKQLGGGLEHHHHHH

>3S2SA

MGSSHHHHHHSSGLVPRGSHMASMTGGQQMGRGSMSKALISIDYTYDFVADDGKLTAGKPAQAISKAIAQVTQKAYDNGDYIFFTIDGHDEGDDFHPETKLFPPHNIKGTSGRDLYGALADFYQKHENDKRVFWMDKRHYSAFSGTDLDIRLRERRVDTVVLTGVLTDICVLHTAIDAYNLGYQIEVVQSAVASLSQENHQFALNHLQNVLGATIIE

>3S2QA

GSHMRKQQRMVVVRAEGGGGINPEIRKNEDKVVDSVVVTELSKNITPYCRCWRSGTFPLCDGSHVKHNKANGDNVGPLLLKKQ

>3S0PA

ATKKAVAVLKGNSNVEGVVTLSQDDDGPTTVNVRITGLAPGLHGFHLHEYGDTTNGCMSTGAHFNPNKLTHGAPGDEIRHAGDLGNIVANADGVAEVTLVDNQIPLTGPNSVVGRALVVHELEDDLGKGGHELSLTTGNAGGRLACGVVGLTPI

>3AYQA

FAGGTVSQRCLSCICKMESGCRNVGCKMDMGSLSCGYFQIKEAYWIDCGRPGSSWKSCAASSYCASLCVQNYMKRYAKWAGCPLRCEGFAREHNGGPRGCKKGSTIGYWNRLQKISGCHGVQ

>3RZIA

MNWTVDIPIDQLPSLPPLPTDLRTRLDAALAKPAAQQPTWPADQALAMRTVLESVPPVTVPSEIVRLQEQLAQVAKGEAFLLQGGDCAETFMDNTEPHIRGNVRALLQMAVVLTYGASMPVVKVARIAGQYAKPRSADIDALGLRSYRGDMINGFAPDAAAREHDPSRLVRAYANASAAMNLVRALTSSGLASLHLVHDWNREFVRTSPAGARYEALATEIDRGLRFMSACGVADRNLQTAEIYASHEALVLDYERAMLRLSDGDDGEPQLFDLSAHTVWIGERTRQIDGAHIAFAQVIANPVGVKLGPNMTPELAVEYVERLDPHNKPGRLTLVSRMGNHKVRDLLPPIVEKVQATGHQVIWQCDPMHGNTHESSTGFKTRHFDRIVDEVQGFFEVHRALGTHPGGIHVEITGENVTECLGGAQDISETDLAGRYETACDPRLNTQQSLELAFLVAEMLRD

>3RQOA

RAPAPATPHAPDHSPAPNSPTLTRPPEGPKFPRVKNWELGSITYDTLCAQSQQDGPCTPRRCLGSLVLPRKLQTRPSPGPPPAEQLLSQARDFINQYYSSIKRSGSQAHEERLQEVEAEVASTGTYHLRESELVFGAKQAWRNAPRCVGRIQWGKLQVFDARDCSSAQEMFTYICNHIKYATNRGNLRSAITVFPQRAPGRGDFRIWNSQLVRYAGYRQQDGSVRGDPANVEITELCIQHGWTPGNGRFDVLPLLLQAPDEAPELFVLPPELVLEVPLEHPTLEWFAALGLRWYALPAVSNMLLEIGGLEFSAAPFSGWYMSTEIGTRNLCDPHRYNILEDVAVCMDLDTRTTSSLWKDKAAVEINLAVLHSFQLAKVTIVDHHAATVSFMKHLDNEQKARGGCPADWAWIVPPISGSLTPVFHQEMVNYILSPAFRYQPDPW

>3RO9A

KVPVVGIVAALLPEMGIGFQGNLPWRLAKEMKYFREVTTLTNDNSKQNVVIMGRKTWESIPQKFRPLPKRINVVVSRSFDGELRKVEDGIYHSNSLRNCLTALQSSLANENKIERIYIIGGGEIYRQSMDLADHWLITKIMPLPETTIPQMDTFLQKQELEQRFYDNSDKLVDFLPSSIQLEGRLTSQEWNGELVKGLPVQEKGYQFYFTLYTKKLEHHHHHHHH

>3RMJA

GIDPFTMTQTNRVIIFDTTLRDGEQSPGAAMTKEEKIRVARQLEKLGVDIIEAGFAAASPGDFEAVNAIAKTITKSTVCSLSRAIERDIRQAGEAVAPAPKKRIHTFIATSPIHMEYKLKMKPKQVIEAAVKAVKIAREYTDDVEFSCEDALRSEIDFLAEICGAVIEAGATTINIPDTVGYSIPYKTEEFFRELIAKTPNGGKVVWSAHCHNDLGLAVANSLAALKGGARQVECTVNGLGERAGNASVEEIVMALKVRHDLFGLETGIDTTQIVPSSKLVSTITGYPVQPNKAIVGANAFSHESGIHQDGVLKHRETYEIMSAESVGWATNRLSLGKLSGRNAFKTKLADLGIELESEEALNAAFARFK

>3AXXA

MEGNTILKIVLICTILAGLFGQVVPVYAENTTYQTPTGIYYEVRGDTIYMINVTSGEETPIHLFGVNWFGFETPNHVVHGLWKRNWEDMLLQIKSLGFNAIRLPFCTESVKPGTQPIGIDYSKNPDLRGLDSLQIMEKIIKKAGDLGIFVLLDYHRIGCTHIEPLWYTEDFSEEDFINTWIEVAKRFGKYWNVIGADLKNEPHSVTSPPAAYTDGTGATWGMGNPATDWNLAAERIGKAILKVAPHWLIFVEGTQFTNPKTDSSYKWGYNAWWGGNLMAVKDYPVNLPRNKLVYSPHVYGPDVYNQPYFGPAKGFPDNLPDIWYHHFGYVKLELGYSVVIGEFGGKYGHGGDPRDVIWQNKLVDWMIENKFCDFFYWSWNPDSGDTGGILQDDWTTIWEDKYNNLKRLMDSCSKSSSSTQSVIRSTTPTKSNTSKKICGPAILIILAVFSLLLRRAPR

>3RKLA

MSKITINIKDNTIEYGHKEFVLSNLQEDIKNLAEIVYQLAKLIEKLSQYEEEVDTELYNLLHEYAIYLAGATSMFIDSENKHHHHHH

>3RIKA

ARPCIPKSFGYSSVVCVCNATYCDSFDPPTFPALGTFSRYESTRSGRRMELSMGPIQANHTGTGLLLTLQPEQKFQKVKGFGGAMTDAAALNILALSPPAQNLLLKSYFSEEGIGYNIIRVPMASCDFSIRTYTYADTPDDFQLHNFSLPEEDTKLKIPLIHRALQLAQRPVSLLASPWTSPTWLKTNGAVNGKGSLKGQPGDIYHQTWARYFVKFLDAYAEHKLQFWAVTAENEPSAGLLSGYPFQCLGFTPEHQRDFIARDLGPTLANSTHHNVRLLMLDDQRLLLPHWAKVVLTDPEAAKYVHGIAVHWYLDFLAPAKATLGETHRLFPNTMLFASEACVGSKFWEQSVRLGSWDRGMQYSHSIITNLLYHVVGWTDWNLALNPEGGPNWVRNFVDSPIIVDITKDTFYKQPMFYHLGHFSKFIPEGSQRVGLVASQKNDLDAVALMHPDGSAVVVVLNRSSKDVPLTIKDPAVGFLETISPGYSIHTYLWHRQ

>3RFYA

GSPGISGGGGGILLVANPVIPDVSVLISGPPIKDPEALLRYALPIDNKAIREVQKPLEDITDSLKIAGVKALDSVERNVRQASRTLQQGKSIIVAGFAESKKDHGNEMIEKLEAGMQDMLKIVEDRKRDAVAPKQKEILKYVGGIEEDMVDGFPYEVPEEYRNMPLLKGRASVDMKVKIKDNPNIEDCVFRIVLDGYNAPVTAGNFVDLVERHFYDGMEIQRSDGFVVQTGDPEGPAEGFIDPSTEKTRTVPLEIMVTGEKTPFYGSTLEELGLYKAQVVIPFNAFGTMAMAREEFENDSGSSQVFWLLKESELTPSNSNILDGRYAVFGYVTDNEDFLADLKVGDVIESIQVVSGLENLANPSYKIAG

>2YFKA

METFKEYIEKLDKLEFEKMYENDFFLTWEKTRDELEAVFTVADTLRYLRENNISTKIFDSGLGISLFRDNSTRTRFSFASACNLLGLEVQDLDEGKSQISHGETVRETANMISFMADIIGIRDDMYIGKGNAYMHEVSESVQEGYKDGVLEQRPTLVNLQCDIDHPTQAMADALHLIHEFGGIENLKGKKVAMTWAYSPSYGKPLSVPQGIVGLMTRLGMDVVLAHPEGYEIMPEVEEVAKKNAAEFGGNFTKTNSMAEAFKDADVVYPKSWAPFAAMEKRTELYGNGDQAGIDQLEQELLSQNKKHKDWECTEELMKTTKDGKALYMHCLPADITGVSCEEGEVEASVFDRYRVELYKEASYKPYVIAAMIFLSKVKNPQKTLTDLADKATPREVKDPNSSSVDKLAAALEHHHHHH

>2LBTA

GQAPPGPPASGPCADLQSAINAVTGGPIAFGNDGASLIPAAYEILNRVADKLKACPDARVTINGYTDNTGSEGINIPLSAQRAKIVADYLVARGVAGDHIATVGLGSVNPIASNATPEGRAKNRRVEIVVNHHHHHH

>3AXDA

MVSMKDFSGAELYTLEEYQYGKFEARMKMAAASGTVSSMFLYQNGSEIADGRPWVEVDIEVLGKSPGSFQSNIITGKAGAQKTSEKHHAVSPAADQAFHTYGLEWTPNYVRWTVDGQEVRKTEGGQVSNLTGTQGLRFNLWSSESAAWVGQFDESKLPLFQFINWVKVYKYTPGQGEGGSDFTLDWTDNFDTFDGSRWGKGDYTFDGNRVDLTDKNIYSRDGMLILALTRKGQESFNGQVPRDDEPAPL

>2YEVB

MQRSFAALGLWGLSLAQEAHRVAITHPGGSFNQEVAFLFPWVYFFSFLIFLVVAGSLAYVTWKFRARPEDQEEPPQIHGNDRLEVVWTLIPLAIVFVLFGLTAKALIQVNRPIPGAMKVEVTGYQFWWDFHYPELGLRNSNELVLPAGVPVELEITSKDVIHSFWVPGLAGKRDAIPGQTTRISFEPKEPGLYYGFCAELCGASHARMLFRVVVLPKEEFDRFVEAAKASPAPVADERGQQVFQQNCAACHGVARSMPPAVIGPELGLWGNRTSLGAGIVENTPENLKAWIRDPAGMKPGVKMPGFPQLSEEDLDALVRYLEGLKVEGFDFGALPKF

>2YEVA

MAITAKPKAGVWAVLWDLLTTVDHKKIGLMYTATAFFAFALAGVFSLLIRTQLAVPNNQFLTGEQYNQILTLHGATMLFFFIIQAGLTGFGNFVVPLMLGARDVALPRVNAFSYWAFLGAIVLALMSYFFPGGAPSVGWTFYYPFSAQSESGVDFYLAAILLLGFSSLLGNANFVATIYNLRAQGMSLWKMPIYVWSVFAASVLNLFSLAGLTAATLLVLLERKIGLSWFNPAVGGDPVLFQQFFWFYSHPTVYVMLLPYLGILAEVASTFARKPLFGYRQMVWAQMGIVVLGTMVWAHHMFTVGESTLFQIAFAFFTALIAVPTGVKLFNIIGTLWGGKLQMKTPLYWVLGFIFNFLLGGITGVMLSMTPLDYQFHDSYFVVAHFHNVLMAGSGFGAFAGLYYWWPKMTGRMYDERLGRLHFWLFLVGYLLTFLPQYALGYLGMPRRYYTYNADIAGWPELNLLSTIGAYILGLGGLVWIYTMWKSLRSGPKAPDNPWGGYTLEWLTASPPKAHNFDVKLPTEFPSERPLYDWKKKGVELKPEDPAHIHLPNSSFWPFYSAATLFAFFVAVAALPVPNVWMWVFLALFAYGLVRWALEDEYSHPVEHHTVTGKSNAWMGMAWFIVSEVGLFAILIAGYLYLRLSGAATPPEERPALWLALLNTFLLVSSSFTVHFAHHDLRRGRFNPFRFGLLVTIILGVLFFLVQSWEFYQFYHHSSWQENLWTAAFFTIVGLHGLHVVIGGFGLILAYLQALRGKITLHNHGTLEAASMYWHLVDAVWLVIVTIFYVW

>3RBUA

RSGLNDIFEAQKIEWHEGSGSGSENLYFQGRSKSSNEATNITPKHNMKAFLDELKAENIKKFLYNFTQIPHLAGTEQNFQLAKQIQSQWKEFGLDSVELAHYDVLLSYPNKTHPNYISIINEDGNEIFNTSLFEPPPPGYENVSDIVPPFSAFSPQGMPEGDLVYVNYARTEDFFKLERDMKINCSGKIVIARYGKVFRGNKVKNAQLAGAKGVILYSDPADYFAPGVKSYPDGWNLPGGGVQRGNILNLNGAGDPLTPGYPANEYAYRRGIAEAVGLPSIPVHPIGYYDAQKLLEKMGGSAPPDSSWRGSLKVPYNVGPGFTGNFSTQKVKMHIHSTNEVTRIYNVIGTLRGAVEPDRYVILGGHRDSWVFGGIDPQSGAAVVHEIVRSFGTLKKEGWRPRRTILFASWDAEEFGLLGSTEWAEENSRLLQERGVAYINADSSIEGNYTLRVDCTPLMYSLVHNLTKELKSPDEGFEGKSLYESWTKKSPSPEFSGMPRISKLGSGNDFEVFFQRLGIASGRARYTKNWETNKFSGYPLYHSVYETYELVEKFYDPMFKYHLTVAQVRGGMVFELANSIVLPFDCRDYAVVLRKYADKIYSISMKHPQEMKTYSVSFDSLFSAVKNFTEIASKFSERLQDFDKSNPIVLRMMNDQLMFLERAFIDPLGLPDRPFYRHVIYAPSSHNKYAGESFPGIYDALFDIESKVDPSKAWGEVKRQIYVAAFTVQAAAETLSEVA

>2YDQA

GSVGPKTGEENQVLVPNLNPTPENLEVVGDGFKITSSINLVGEEEADENAVNALREFLTANNIEINSENDPNSTTLIIGEVDDDIPELDEALNGTTAENLKEEGYALVSNDGKIAIEGKDGDGTFYGVQTFKQLVKESNIPEVNITDYPTVSARGIVEGFYGTPWTHQDRLDQIKFYGENKLNTYIYAPKDDPYHREKWREPYPESEMQRMQELINASAENKVDFVFGISPGIDIRFDGDAGEEDFNHLITKAESLYDMGVRSFAIYWDNIQDKSAAKHAQVLNRFNEEFVKAKGDVKPLITVPTEYDTGAMVSNGQPRAYTRIFAETVDPSIEVMWTGPGVVTNEIPLSDAQLISGIYDRNMAVWWNYPVTDYFKGKLALGPMHGLDKGLNQYVDFFTVNPMEHAELSKISIHTAADYSWNMDNYDYDKAWNRAIDMLYGDLAEDMKVFANHSTRMDNKTWAKSGREDAPELRAKMDELWNKLSSKEDASALIEELYGEFARMEEACNNLKANLPEVALEECSRQLDELITLAQGDKASLDMIVAQLNEDTEAYESAKEIAQNKLNTALSSFAVISEKVAQSFIQEALS

>3R6TA

MGDTKEQRILRYVQQNAKPGDPQSVLEAIDTYCTQKEWAMNVGDAKGQIMDAVIREYSPSLVLELGAYCGYSAVRMARLLQPGARLLTMEINPDCAAITQQMLNFAGLQDKVTILNGASQDLIPQLKKKYDVDTLDMVFLDHWKDRYLPDTLLLEKCGLLRKGTVLLADNVIVPGTPDFLAYVRGSSSFECTHYSSYLEYMKVVDGLEKAIYQGPSSPDKS

>3R3JA

LHNYGYTSTKSVDNQIEELREKVVSKNKNEPEFLQAFEEVLSCLKPVFKKDNVYIGVLENIAEPERVIQFRVPWINDKGEHKMNRGFRVQYNSVLGPYKGGLRFHPAVNLSVIKFLGFEQIFKNSLTTLPMGGGKGGSDFDPKGKSENEILKFCQSFMTNLFRYIGPNTDVPAGDIGVGGREIGYLFGQYKKLKNSFEGVLTGKNIKWGGSNIRAEATGYGVVYFAENVLKDLNDNLENKKCLVSGSGNVAQYLVEKLIEKGAIVLTMSDSNGYILEPNGFTKEQLNYIMDIKNNQRLRLKEYLKYSKTAKYFENQKPWNIPCDIAFPCATQNEINENDADLFIQNKCKMIVEGANMPTHIKALHKLKQNNIILCPSKAANAGGVAVSGLEMSQNSMRLQWTHQETDMKLQNIMKSIYEQCHNTSKIYLNESDLVAGANIAGFLKVADSFLEQGGL

>3R18A

APSYPEYTREEVGRHRSPEERVWVTHGTDVFDVTDFVELHPGGPDKILLAAGGALEPFWALYAVHGEPHVLELLQQYKVGELSPDEAPAAPDAQDPFAGDPPRHPGLRVNSQKPFNAEPPAELLAERFLTPNELFFTRNHLPVPAVEPSSYRLRVDGPGGGTLSLSLAELRSRFPKHEVTATLQCAGNRRSEMSRVRPVKGLPWDIGAISTARWGGARLRDVLLHAGFPEELQGEWHVCFEGLDADPGGAPYGASIPYGRALSPAADVLLAYEMNGTELPRDHGFPVRVVVPGVVGARSVKWLRRVAVSPDESPSHWQQNDNKGFSPCVDWDTVDYRTAPAIQELPVQSAVTQPRPGAAVPPGELTVKGYAWSGGGREVVRVDVSLDGGRTWKVARLMGDKAPPGRAWAWALWELTVPVEAGTELEIVCKAVDSSYNVQPDSVAPIWNLMGVLSTAWHRVRVSVQD

>3QVSA

MKVWLVGAYGIVSTTAMVGARAIERGIAPKIGLVSELPHFEGIEKYAPFSFEFGGHEIRLLSNAYEAAKEHWELNRHFDREILEAVKSDLEGIVARKGTALNCGSGIKELGDIKTLEGEGLSLAEMVSRIEEDIKSFADDETVVINVASTEPLPNYSEEYHGSLEGFERMIDEDRKEYASASMLYAYAALKLGLPYANFTPSPGSAIPALKELAEKKGVPHAGNDGKTGETLVKTTLAPMFAYRNMEVVGWMSYNILGDYDGKVLSARDNKESKVLSKDKVLEKMLGYSPYSITEIQYFPSLVDNKTAFDFVHFKGFLGKLMKFYFIWDAIDAIVAAPLILDIARFLLFAKKKGVKGVVKEMAFFFKSPMDTNVINTHEQFVVLKEWYSNLK

>3QSYA

MAWPKVQPEVNIGVVGHVDHGKTTLVQAITGIWTSKHSEELKRGMTIKLGYAETNIGVCESCKKPEAYVTEPSCKSCGSDDEPKFLRRISFIDAPGHEVLMATMLSGAALMDGAILVVAANEPFPQPQTREHFVALGIIGVKNLIIVQNKVDVVSKEEALSQYRQIKQFTKGTWAENVPIIPVSALHKINIDSLIEGIEEYIKTPYRDLSQKPVMLVIRSFDVNKPGTQFNELKGGVIGGSIIQGLFKVDQEIKVLPGLRVEKQGKVSYEPIFTKISSIRFGDEEFKEAKPGGLVAIGTYLDPSLTKADNLLGSIITLADAEVPVLWNIRIKYNLLERVVGAKEMLKVDPIRAKETLMLSVGSSTTLGIVTSVKKDEIEVELRRPVAVWSNNIRTVISRQIAGRWRMIGWGLVEI

>3AUKA

MAASRANDAPIVLLHGFTGWGREEMFGFKYWGGVRGDIEQWLNDNGYRTYTLAVGPLSSNWDRACEAYAQLVGGTVDYGAAHAAKHGHARFGRTYLGLLPELKRGGRIHIIAHSQGGQTARMLVSLLENGSQEEREYAKAHNVSLSPLFEGGHHFVLSVTTIATPHDGTTLVNMVDFTDRFFDLQKAVLEAAAVASNVPYTSQVYDFKLDQWGLRRQPGESFDHYFERLKRSPVWTSTDTARYDLSVSGAEKLNQWVQASPNTYYLSFATERTYRGALTGNYYPELGMNAFSAVVCAPFLGSYRNPTLGIDDRWLENDGIVNTVSMNGPKRGSSDRIVPYDGALKKGVWNDMGTYNVDHLEIIGVDPNPSFDIRAFYLRLAEQLASLQP

>3QMXA

MRGSHHHHHHGSAVSAKIEIYTWSTCPFCMRALALLKRKGVEFQEYCIDGDNEAREAMAARANGKRSLPQIFIDDQHIGGCDDIYALDGAGKLDPLLHS

>3QLIA

HHHHHHSSGLVPRGSMDIRALYDEKLTTPEEAVSSIASGSHLSMGMFAAEPPALLKALADRATRGDIGDLRVYYFETAKIAGDTILRYELNNRIKPYSMFVTAVERALIRRGIEDGGRKVVNYVPSNFHQAPRLLAEEIGIDTFMHTVSPMDCHGYFSLGVGNDYSSRIARSARRFIVEVNRYMPRVQGEAAAIHISEVDAIVENHVPLIEMPVRSAIPEYTSISHIIADLVPDGACLQMGVGALPNLVCGVLKDRNDLGIHTEVLNPGLVDLIRRGVVTNQRKTLDRGRSVFTFAMGQQEMYEYLNDHPAIFSRPVDYVNDPHIIAQNDNVVSINATLQIDLTGACNSEHMLGHQYSASGGQLDFVRGAYASKGGRSIIATPSTAAKGTVSRIIPRIDGPVTTPRIDTHYIVTEFGAVNLKGLSSTERALRIIELAHPDFRDELTQAAKKMHLI

>2L97A

MEGLGFAIPANDAINIIEQLEKNGKVTRPALGIQMVNLSNVSTSDIRRLNIPSNVTSGVIVRSVQSNMPANGHLEKYDVITKVDDKEIASSTDLQSALYNHSIGDTIKITYYRNGKEETTSIKLNKLEHHHHHH

>3QKGA

GPVPTPPDNIQVQENFNISRIYGKWYNLAIGSTSPWLKKIMDRMTVSTLVLGEGATEAEISMTSTRWRKGVCEETSGAYEKTDTDGKFLYHKSKWNITMESYVVHTNYDEYAIFLTKKFSRHHGPTITAKLYGRAPQLRETLLQDFRVVAQGVGIPEDSIFTMADRGECVPGEQEPEPILIPRSAWSHPQFEK

>3QJJA

HHHHMRIEVKLLPLKDNPILPFNYNYEVYSQILEKVNSIEPTIAKLLSSPHGFWTFSRIIVRKRKILPDKGIEILSDDVSLYISSSNEDIIRAIAEAVEKSPEFKIGELSFLVGDIKAIKVKELGKENVFSTLSPIVVRTVKFEGNKLRHWDLYPHDELFMDRLRKVMILRYSEVMGETPKDRDFTIEVLKFKPTRLMVGSSYIRGSLMVFRYAGSEEIARFGYENGFGEKTGLGFGMVKLIE

>2Y6YA

GAMTIGRAKVYATLSKIFYHLFYDEAIPKDCREIIEKFGEIDFNLRSVLVRELRGSVLIKDMPQSLAEVYESVMKDFYERYGFQASELHADHIAVELAFMSKLVEREISLAQQMKEEELYKIRAAQHRFIKAHLQPLVKNLPSAPLLNFVRDFVREDAKYLYSSLVGEKNEGADNN

>2L8LA

CLAEGTRIFDPVTGTTHRIEDVVDGRKPIHVVAAAKDGTLHARPVVSWFDQGTRDVIGLRIAGGAILWATPDHKVLTEYGWRAAGELRKGDRVAVRDVETGELRYSVIREVLPTRRARTFDLEVEELHTLVAEGVVVHN

>3QDDA

HMPEETQTQDQPMEEEEVETFAFQAEIAQLMSLIINTFYSNKEIFLRELISNSSDALDKIRYESLTDPSKLDSGKELHINLIPNKQDRTLTIVDTGIGMTKADLINNLGTIAKSGTKAFMEALQAGADISMIGQFGVGFYSAYLVAEKVTVITKHNDDEQYAWESSAGGSFTVRTDTGEPMGRGTKVILHLKEDQTEYLEERRIKEIVKKHSQFIGYPITLFVEKERDKEVSDDEAE

>3QBDA

HHHHHHMTDIGAPVTVQVAVDPPYPVVIGTGLLDELEDLLADRHKVAVVHQPGLAETAEEIRKRLAGKGVDAHRIEIPDAEAGKDLPVVGFIWEVLGRIGIGRKDALVSLGGGAATDVAGFAAATWLRGVSIVHLPTTLLGMVDAAVGGKTGINTDAGKNLVGAFHQPLAVLVDLATLQTLPRDEMICGMAEVVKAGFIADPVILDLIEADPQAALDPAGDVLPELIRRAITVKAEVVAADEKESELREILNYGHTLGHAIERRERYRWRHGAAVSVGLVFAAELARLAGRLDDATAQRHRTILSSLGLPVSYDPDALPQLLEIMAGDKKTRAGVLRFVVLDGLAKPGRMVGPDPGLLVTAYAGVCAP

>3QB4A

MKRQGKRPSKNLKARCSRKALHVNFKDMGWDDWIIAPLEYEAFHCEGLCEFPLASHLEPTNHAVIQTLMNSMDPESTPPTCCVPTRLSPISILFIDSANNVVYKQYEDMVVESCGCR

>2L8BA

TSGIHVLDELSVRALSRDIMKQNRVTVHPEKSVPRTAGYSDAVSVLAQDRPSLAIVSGQGGAAGQRERVAELVMMAREQGREVQIIAADRRSQMNMKQDERLSGELITGRRQLLEGMAFTPGSTVIVDQGEKLSLKETLTLLDGAARHNVQVLITDSGQRTGTGSALMAMKDAGVNTYRWQGGEQRPAT
